# Global Data Compilation Across Climate Gradients Supports the Use of Common Allometric Equations for Three Transatlantic Mangrove Species

**DOI:** 10.1002/ece3.70577

**Published:** 2024-11-20

**Authors:** Charles A. Price, Benjamin Branoff, Karen Cummins, Romain Glèlè Kakaï, Danielle Ogurcak, Monica Papeș, Michael Ross, Kevin R. T. Whelan, Todd A. Schroeder

**Affiliations:** ^1^ Ecology and Evolutionary Biology University of Tennessee Knoxville Tennessee USA; ^2^ USDA Forest Service, Southern Research Station Forest Inventory and Analysis Knoxville Tennessee USA; ^3^ Tall Timbers Research Station and Land Conservancy Tallahassee Florida USA; ^4^ Laboratory of Biomathematics and Forest Estimations University of Abomey‐Calavi Cotonou Benin; ^5^ Institute of Environment, Florida International University Miami Florida USA; ^6^ National Institute for Mathematical and Biological Synthesis, University of Tennessee Knoxville Tennessee USA; ^7^ National Park Service South Florida Caribbean Inventory and Monitoring Network Palmetto Bay Florida USA

**Keywords:** allometry, *Avicennia germinans*, biomass, DBH, height, *Laguncularia racemosa*, *mangrove*, *Rhizophora mangle*, root mass

## Abstract

Predicting the distribution, structure, and biomass of mangrove forests is an area of high research interest. Across the Atlantic East Pacific biogeographic region, three species are common and abundant members of local mangrove communities; *Rhizophora mangle*, *Avicennia germinans*, and *Laguncularia racemosa*. Biomass prediction for these species has relied on two approaches: site‐specific allometries based on the idea that environmental/climatic differences between sites drive growth differences, or the use of common allometric equations based on the idea that site driven differences are minimal. Meta‐analyses of global compilations of interspecific plot level data (e.g., mean canopy height, stand basal area) show trends in size and structure with climatic variables, however this has not been critically evaluated across these species using empirical allometric growth functions. We compared allometric equations derived from 590 individuals within and across nine broadly distributed sites at interspecific and intraspecific levels and explored the influence of climatic variables on allometric slopes and intercepts. Assessing variables that can be used to predict biomass in the field (height, diameter at breast height (DBH), canopy spread), we find interspecific root mean squared errors similar to or smaller than most intraspecific or site‐specific equations, particularly when examining sites with sample sizes above recommended values. We also find significant effects of several climatic variables on growth allometries with the strongest effects from minimum temperature followed by precipitation seasonality. Our results suggest that while climate has a clear influence on mangrove allometric growth, common equations may have utility in biomass prediction. Future methodological improvements, particularly larger sample sizes across the entire available size range, combined with data from a broader range of growth conditions will further inform which allometric relationships exhibit the most variability within and across sites and which variables best predict mangrove biomass.

## Graphical abstract

Our work indicates that the use of common equations to estimate above ground biomass may be justified in mangrove forests. We have also shown that temperature and to a lesser extent, precipitation, have a significant influence on the growth allometries of these species.

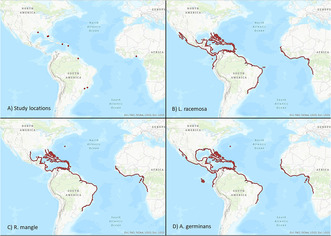

## 1 Introduction

In the intertidal zone where periodic saltwater inundation combined with evapotranspiration results in high soil salinity levels, a relatively small number of halophytic plant species have evolved to thrive (Wang et al. 2011). Known collectively as mangroves, approximately 70 species globally occupy this zone in two major biogeographic regions, the Indo‐West Pacific and Atlantic East Pacific (AEP) (Duke 2017; Lugo and Snedaker 1974; Rovai et al. 2021), and across a range of biophysical typologies (Lugo and Snedaker 1974; Worthington et al. 2020).

Mangrove forests are recognized as critical habitats and recruitment zones for an array of marine and terrestrial species (Laegdsgaard and Johnson 2001; Mumby et al. 2004). The soils in mangroves contain carbon levels that are among the highest found in the tropics (Donato et al. 2011; Komiyama, Ong, and Poungparn 2008). Mangroves have also been noted for their ability to buffer storm damage from cyclones or hurricanes (Alongi 2008; Zhang et al. 2012). With mean production levels of 2.5 g C m^−2^ day^−1^ (Jennerjahn and Ittekkot 2002) mangroves are also among the most productive ecosystems on Earth and their importance in blue carbon economics has been increasingly recognized (Bertram et al. 2021).

The array of natural and ecosystem services provided by mangroves highlights the need for methods to accurately quantify community and landscape level biomass of their forests, so that we might better manage and protect them. This is especially important in areas where mangroves are subject to intense periodic disturbance such as floods or hurricanes.

**FIGURE 1:**
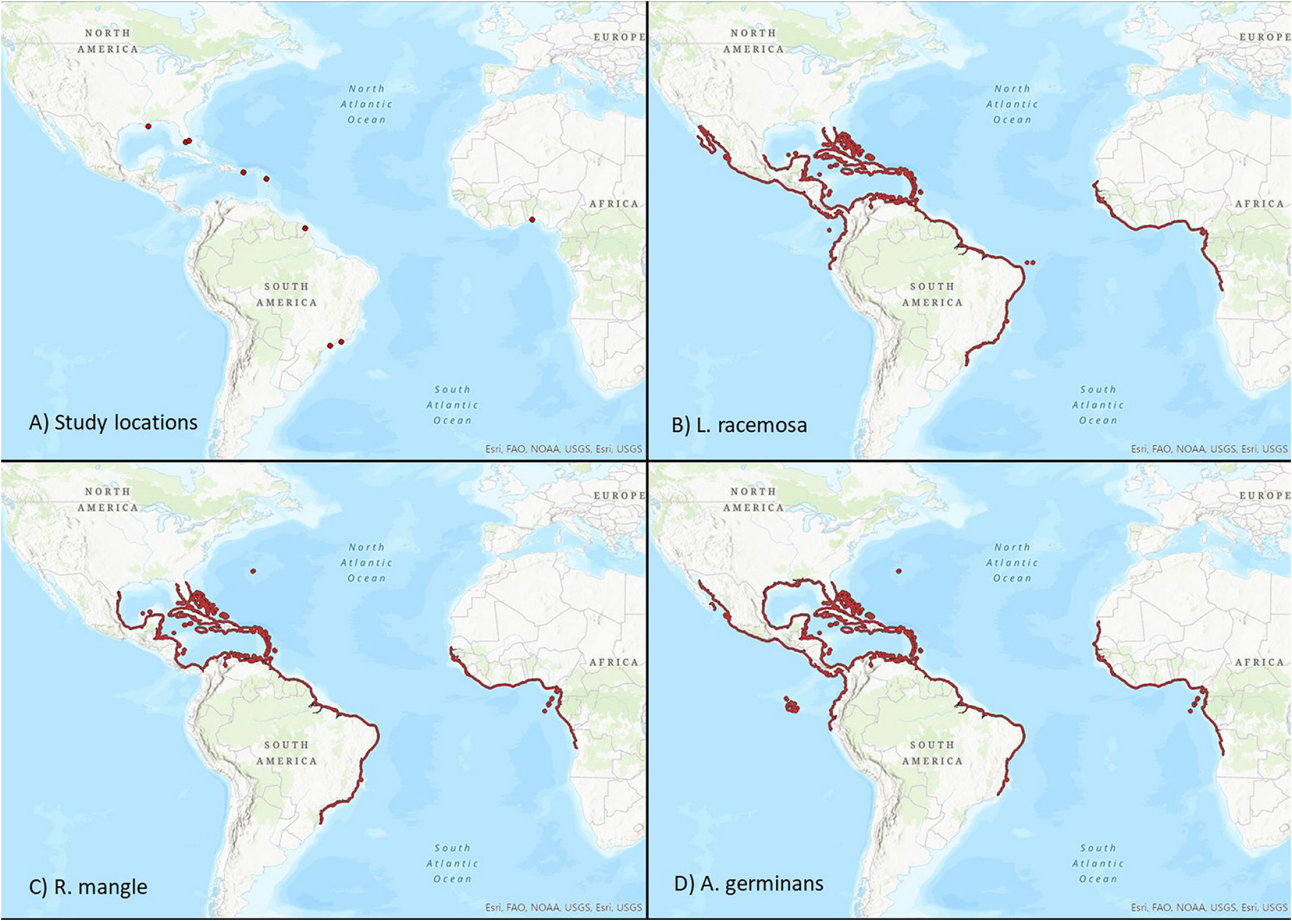
Site locations and distribution maps for the three species. The nine study locations from which allometric data were aggregated are shown in Panel (A). Panels (B–D) are the distribution maps for the three species which were modified from shapefiles obtained from IUCN (see 2 Methods). Recent work suggests that *R. mangle* may be found on the Pacific coast of the Americas (Montes‐Chaura et al. 2024).

Among the AEP mangroves, three species are frequently noted as prominent members of local flora across the region, *Avicennia germinans*, *Laguncularia racemosa*, and *Rhizophora mangle* (Day et al. 1987; Soares and Schaeffer‐Novelli 2005; Spalding, Kainuma, and Collins 2010; Zanvo et al. 2023). These three species are found throughout coastal areas of North, Central, and South America, the Caribbean, and along the west coast of Africa, typically range between 25 N and 25 S latitude (Figure 1) with ranges expanding (Osland et al. 2022). These species are also the only true mangroves found in the continental U.S. Of the 47 AEP countries listed as having mangroves in the World Atlas of Mangroves (Spalding, Kainuma, and Collins 2010), *A. germinans* has been reported in all 47, *L. racemosa* occurs in 42 of 47 countries, and *R. mangle* occurs in 41 of 47 countries.

Efforts to estimate mangrove biomass at the community and landscape scale typically rely on allometric relationships. Numerous studies have reported measurements for the above ground dimensions (height, DBH, crown diameter) and biomass (bole/stem, leaf) which are used to develop allometric equations (Fromard, Vega, and Proisy 2004; Ross et al. 2001; Smith and Whelan 2006; Soares and Schaeffer‐Novelli 2005) that can subsequently be applied to estimate biomass non‐destructively. Collecting allometric data for large terrestrial plants are challenging by itself, and the habitats in which mangroves are found make this inordinately difficult. Moreover, in many countries, mangroves are protected species making harvesting new individuals to develop empirical allometries less likely to occur.

Measures of mangrove structure, biomass, and stem density have been shown to vary with latitude, local soil, and water conditions (Lugo and Snedaker 1974; Rovai et al. 2021). Thus, many investigators operate on the assumption that local edaphic or climatic processes will have a strong influence on mangrove growth and sample trees locally to develop allometric equations for the estimation of biomass for that area (Golley, Howard, and Ronald 1962; Guerra‐Santos et al. 2014; Imbert and Rollet 1989; Njana et al. 2016; Osland et al. 2014; Sherman, Fahey, and Martinez 2003; Smith and Whelan 2006; Soares and Schaeffer‐Novelli 2005).

However, not all allometric relationships are equal, and allometric theory suggests that although local environmental processes might affect the mature size of trees and whether they invest more in increasing canopy height or spread (growing up or out), some allometric relationships will be more constrained than others. For example, the relationship between DBH and above ground mass represents the amount of biomechanical and hydraulic tissue required to support and supply the above ground mass (Price et al. 2022; West, Brown, and Enquist 1999). Similarly, the amount of leaf material (mass, number) will be strongly dependent on plant size and the amount of above ground stem mass as leaves are produced from carbohydrates stored within stems, thus one might expect these relationships to exhibit less variability across sites compared to measures of plant height or canopy spread.

The height of mangrove trees is known to vary latitudinally at geographic scales (Lugo and Snedaker 1974; Rovai et al. 2021) and can be influenced regionally by soil nutrient composition and surface hydrology (Cintron et al. 1978; Saintilan 1997; Sherman, Fahey, and Martinez 2003), salinity, humidity, and temperature (Devaney et al. 2021), and disturbance frequency and intensity (Krauss and Osland 2020; Simard et al. 2019). In regions where mangroves attain tall heights and high stem density, competition for light may be more intense and mangroves may invest more biomass in growing taller. In contrast, in areas where mangrove stature is limited, competition for light may be less intense and mangroves might invest more in crown spread. This broad trend can be influenced by disturbance, where tree damage or mortality may create gaps in canopies altering investment patterns. Overall, we expect tree height or crown spread to exhibit more variability than other measured variables, reflecting the influence of local processes, and thus allometric regressions involving height or spread will exhibit more variance than those based on DBH.

Our central research question can be cast as two alternative research hypotheses. H1: site/species specific allometric relationships reduce errors associated with biomass estimation due to the influence of local environmental factors, and H2: intraspecific (across sites) or interspecific general allometric equations reduce errors associated with biomass estimation. To investigate how site and associated climate influence allometric regressions, we aggregated allometric data for 590 individuals across the three aforementioned species from eight published studies comprising nine sites in the AEP (Figure 1, Table S1). This unique dataset allows us to address the following questions related to our core hypotheses:

1. Do intraspecific allometric regression equations differ across sites and if so by how much?
2. Which allometric relationships exhibit the least and most variability?
3. Which linear measure (height, crown, DBH) is the best predictor of above ground biomass?
4. Are species/site level regression slopes and intercepts influenced by climatic variables and if so which variables have the greatest influence?
5. How does sample size and collection methodology influence allometric relationships and are existing sample sizes adequate?

## 2 Materials and Methods

We utilized the Web of Science search engine in an attempt to find all studies reporting allometric data for the following three species: *Rhizophora mangle*, *Avicennia germinans*, and *Laguncularia racemosa*. This yielded a number of published reports (Fromard, Vega, and Proisy 2004; Golley, Howard, and Ronald 1962; Imbert and Rollet 1989; Osland et al. 2014; Ross et al. 2001; Smith and Whelan 2006; Soares and Schaeffer‐Novelli 2005). Several of these studies had allometric data published within their papers which we aggregated (Golley, Howard, and Ronald 1962; Imbert and Rollet 1989; Osland et al. 2014; Soares and Schaeffer‐Novelli 2005). For the remaining studies, we reached out to the corresponding authors and requested their data which several graciously provided (Ross et al. 2001; Smith and Whelan 2006; Zanvo et al. 2023). For the Fromard et al. (1998) study, we were able to extract data using the plot digitizer application (plotdigitizer.com), which has been shown to be highly reliable and accurate in test cases (Aydin and Yassikaya 2022). Several authors either did not respond, were unable to share data, or were unable to locate the data due to the long time passed since the studies were published (Day et al. 1987; Santos et al. 2017; Sherman, Fahey, and Martinez 2003; Yepes et al. 2016).

Site locations (Table S1) were obtained from each paper or estimated based on descriptions in the paper (Golley, Howard, and Ronald 1962; Imbert and Rollet 1989; Soares and Schaeffer‐Novelli 2005; Zanvo et al. 2023). For full site descriptions, refer to each paper individually. Not all of the studies we found measured the same parameters and sample sizes differed considerably between studies (Table S1).

Climate data for each site were obtained from an online database for gridded climate data (www.worldclim.org) containing 24 climatic variables related to temperature, precipitation and solar radiation (Fick and Hijmans 2017). The latitude and longitude locations for the Biscayne and Louisiana sites were recorded in WorldClim as being located over water (not surprising for mangroves) so the average of gridded cells within 1 km of those locations for each variable was used.

Species distribution maps were downloaded as shape files from the IUCN website (Ellison, Farnsworth, and Moore 2010a, 2010b, 2015) with the distribution data colored red in ArcGIS Pro (ESRI 2011) (Figure 1). Site locations were also loaded into ArcGIS Pro to create Figure 1.

Several of the datasets contained different bole and branch biomass categories which were combined with leaf mass data into a single estimate of above ground mass (Imbert and Rollet 1989; Soares and Schaeffer‐Novelli 2005). For brevity, we use the following terms to refer to the measured allometric variables: height (m)*—*height of the plant above ground level, mass (kg)—above ground mass of all plant parts (bole, stems, leaves), leaves (kg)—leaf mass, roots (kg)—*R. mangle* above ground prop root mass, DBH (cm)—diameter at breast height (130 cm above ground), and crown (m)—crown diameter.

Only one of the species had root measures for more than one site, so we have only analyzed allometric relationships with roots for *R. mangle*. Two of the papers had measurements for trunk diameter at 30 cm (D30) above ground (Osland et al. 2014; Ross et al. 2001). Ross et al. (2001) had both D30 or *DBH* measures in their study. D30 measures were not included in our standardized major axis (SMA) regressions because they are not analogous to *DBH* measures, but other measures (*height*, *leaves*, *mass*, *crown*) for those plants were included.

### 2.1 Replication Statement

**Table:** 

Scale of inference	Scale at which the factor of interest is applied	Number of replicates at the appropriate scale
Species	Species	9
Interspecies	Interspecies	1

Bivariate relationships among the six field measured variables (*height*, *DBH*, *leaves*, *roots*, *mass*, *crown*) were estimated using standardized major axis regression (SMA) (aka Reduced Major Axis (RMA), Cohen et al. 2003). Regression exponents were estimated using the software package Standardized Major Axis Tests and Routines (SMATR) (Falster, Warton, and Wright 2006; Warton et al. 2006). SMA regression is typically used in allometric studies where both variables have associated measurement error and one wants to test if slopes from different datasets are equal, as is the case here (Warton et al. 2006). All data were log transformed before regression fitting to meet the regression assumption of homogeneity of variance. Regression functions were fit (1) interspecifically across all sites, (2) intraspecifically across sites, and (3) to all site‐species combinations. For the intraspecific, within site regressions we tested whether each relationship (i.e., *mass‐DBH* or *crown‐height*) had a common slope across sites (Tables S3–S5). All data were tested at the *p* > 0.05 significance level.

To determine which above ground linear measure (*height*, *DBH*, *crown*) was the best predictor of total above ground biomass (*mass*) and to compare results between regression models at different grouping levels (interspecifically, intraspecifically across sites, intraspecifically within sites) we calculated the root mean squared error (RMSE) using standard formula, which represents the mean difference between the values predicted by the regression model and the actual measured values.

To examine whether climatic variables had any influence on allometric slopes across sites and within species, we used ordinary least squares regression (OLS). OLS is better suited than SMA when testing whether the relationship between two variables is different than zero which is indicated when the *p*‐value for the regression slope is < 0.05 (Warton et al. 2006). We limited our analysis to those relationships that had at least four data points (sites) and for each climatic variable, we calculated regression statistics for the relationship between the values for that climate variable, and the slopes and intercepts for each site.

## 3 Results

**FIGURE 2:**
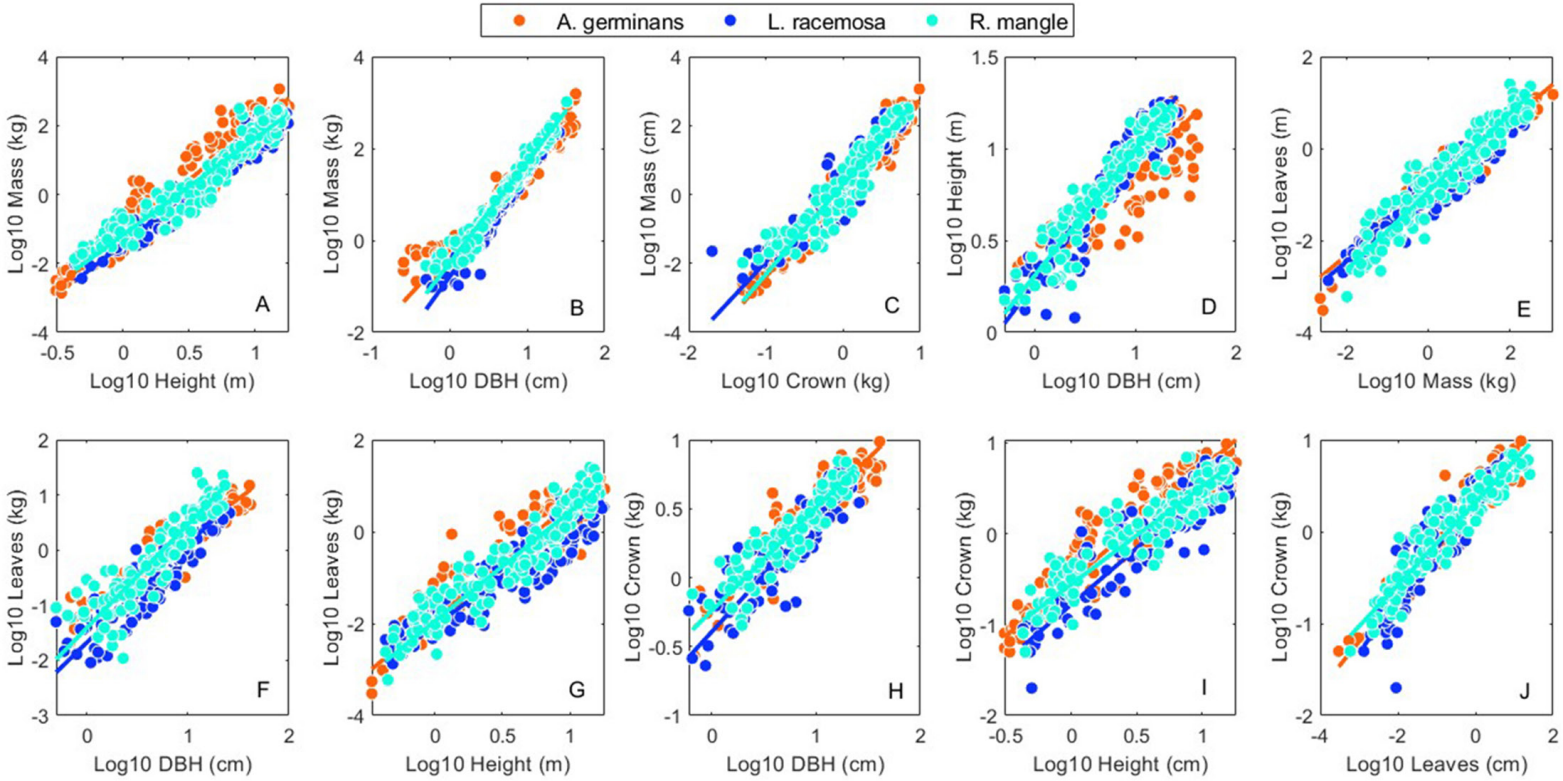
Bivariate relationships for *A. germinans*, *L. racemosa* and *R. mangle*. Panels (A–J) correspond to bivariate allometric relationships for *height*, *DBH*, *mass*, leaves and *crown* (see 2 Methods) across all sites. Symbol colors correspond to species as shown in the legend. Regression statistics for these 10 relationships are reported in Table 1. Note the relatively low variance around the *mass* to *DBH* (mean *R* ^2^ = 0.957) and the *height* to *mass* relationships (mean *R* ^2^ = 0.916).

**FIGURE 3:**
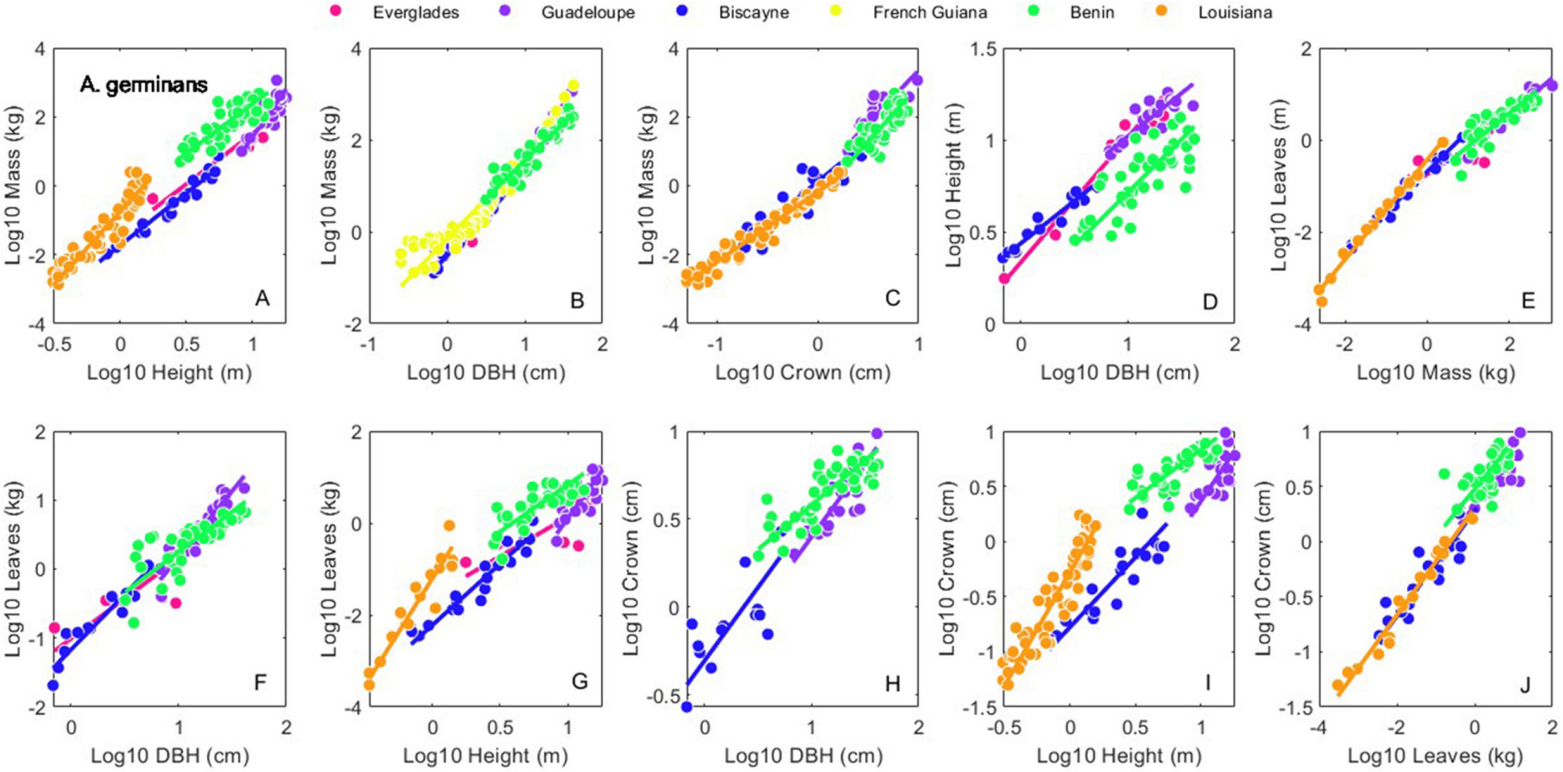
Site specific bivariate relationships for *A. germinans*. Allometric relationships for *height*, *DBH*, *mass*, leaves and *crown* (see 2 Methods) within all sites. Symbol colors correspond to sites as shown in the legend. Regression statistics for these 10 relationships are reported in Table S3.

**FIGURE 4:**
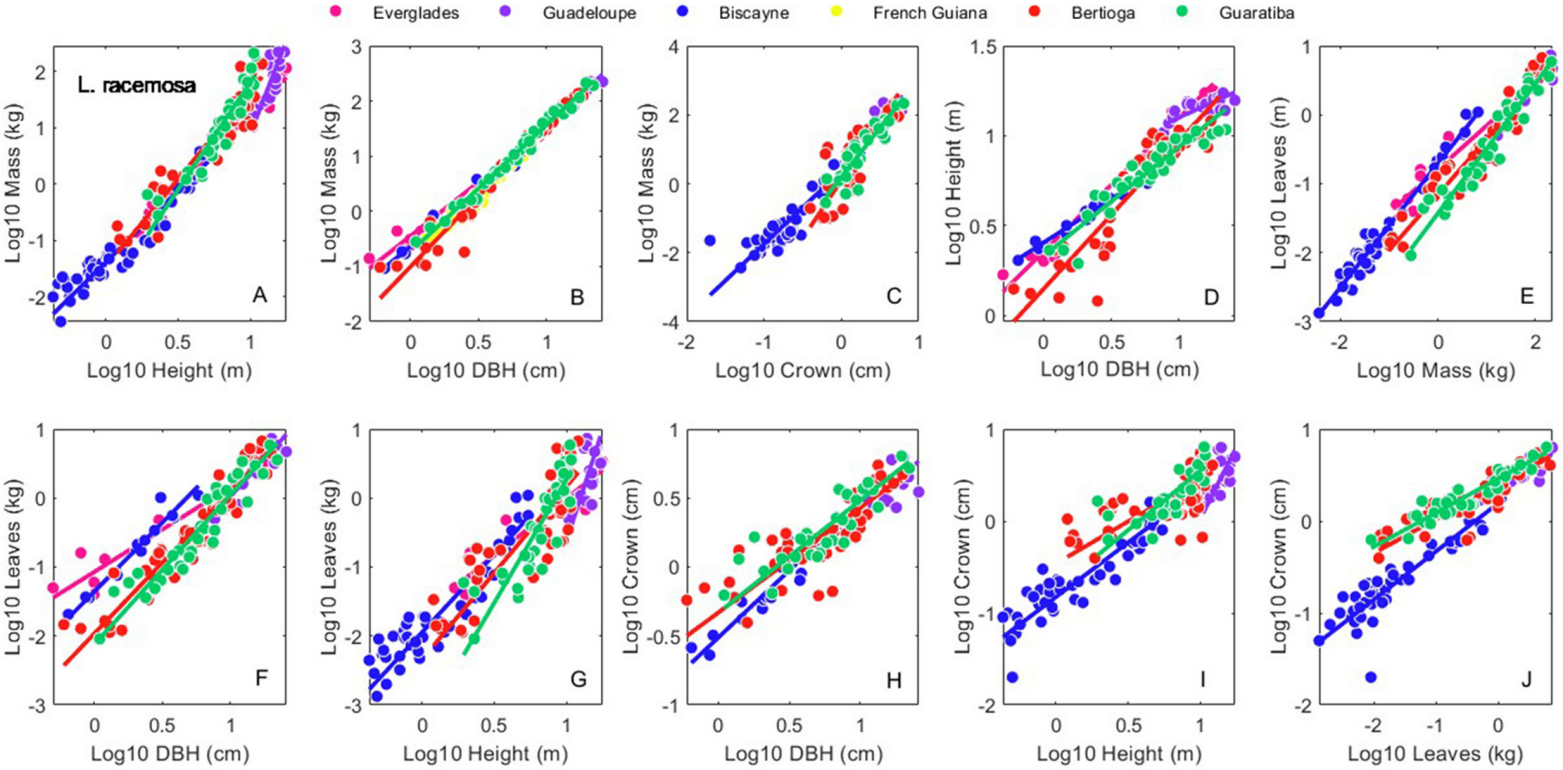
Site specific bivariate relationships for *L. racemosa*. Allometric relationships for *height*, *DBH*, *mass*, leaves and *crown* (see 2 Methods) within all sites. Symbol colors correspond to sites as shown in the legend. Regression statistics for these 10 relationships are reported in Table S4.

**FIGURE 5:**
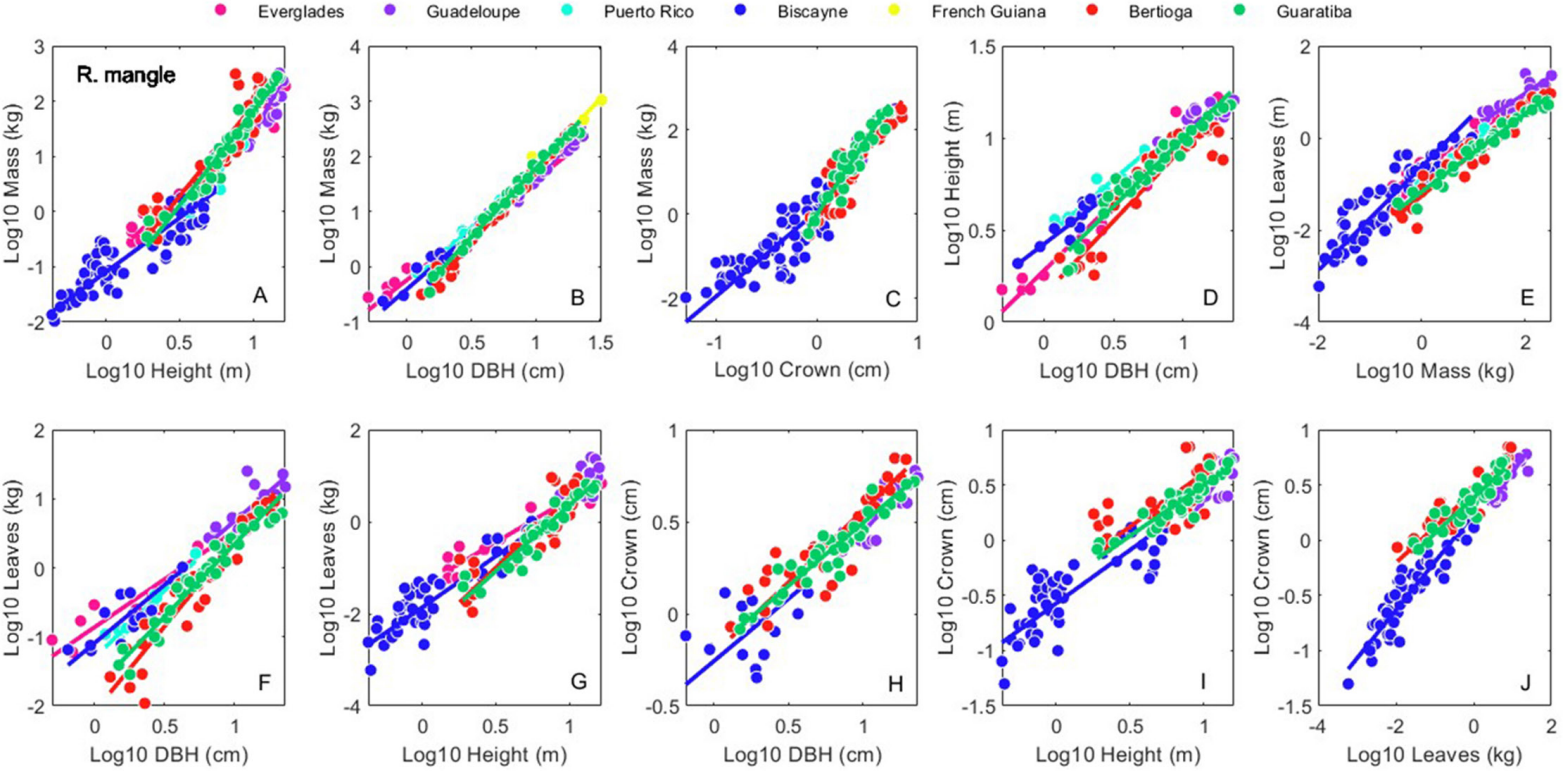
Site specific bivariate relationships for *R. mangle*. Allometric relationships for *height*, *DBH*, *mass*, leaves and *crown* (see 2 Methods) within all sites. Symbol colors correspond to sites as shown in the legend. Regression statistics for these 10 relationships are reported in Table S5.

We found eight allometric studies covering 590 individuals, six variables (*height*, *DBH*, *crown*, *mass*, *leaves*, *roots*) and nine sites across the AEP (Figure 1, Table S1). Across all sites there were 215 *A. germinans*, 200 *L. racemosa*, and 175 *R. mangle* individuals. The full combined allometric dataset is in Table S2. Intrasite regression statistical results from the SMATR program for *A. germinans*, *L. racemosa* and *R. mangle*, are available in Tables S3–S5, respectively. Results for the interspecific regressions, and the across site, intraspecific regressions are in Figures 2, 3, 4, 5 and S1, Table 1.

**TABLE 1:** Across site interspecific and intraspecific SMA regression results.

Log_10_ Y variable	Log_10_ X variable	Group	Figures	*n*	*R* ^2^	Slope	LowCI	UppCI	Interc	LowCI	UppCI	*A. germinans*	*L. racemosa*	*R. mangle*	*MeanSiteR*2	*Climate*
Mass (kg)	Height (m)	Interspecific	NA	492	0.903	2.9500	2.870	3.033	−1.2500	−1.308	−1.191	—	—	—	0.850	7
Mass (kg)	DBH (cm)	Interspecific	NA	418	0.931	2.0740	2.022	2.127	−0.4958	−0.540	−0.452	—	—	—	0.958	5
Mass (kg)	Crown (m)	Interspecific	NA	443	0.908	2.5320	2.461	2.604	0.3124	0.272	0.353	—	—		0.790	4
Height (m)	DBH (cm)	Interspecific	NA	320	0.781	0.6438	0.612	0.678	0.3010	0.271	0.331	—	—	—	0.839	5
Leaves (kg)	Mass (kg)	Interspecific	NA	452	0.922	0.7792	0.759	0.800	−0.9487	−0.977	−0.920	—	—	—	0.892	11
Leaves (kg)	DBH (cm)	Interspecific	NA	320	0.804	1.7260	1.644	1.812	−1.4750	−1.550	−1.399	—	—	—	0.832	0
Leaves (kg)	Height (m)	Interspecific	NA	452	0.843	2.3520	2.267	2.439	−1.9760	−2.040	−1.912	—	—	—	0.742	6
Crown (m)	DBH (cm)	Interspecific	NA	271	0.812	0.7887	0.749	0.831	−0.3122	−0.350	−0.274	—	—	—	0.671	6
Crown (m)	Height (m)	Interspecific	NA	443	0.801	1.1740	1.126	1.224	−0.6142	−0.649	−0.579	—	—	—	0.607	9
Crown (m)	Leaves (kg)	Interspecific	NA	403	0.876	0.5110	0.494	0.529	0.3542	0.333	0.376	—	—	—	0.794	11
Mass (kg)	Height (m)	*A. germinans*	2A	158	0.896	3.1250	2.970	3.288	−1.1410	−1.249	−1.033	1	0.13	0.002	0.845	0
Mass (kg)	Height (m)	*L. racemosa*	2A	163	0.946	2.9690	2.864	3.079	−1.4280	−1.509	−1.348	0.13	1	0.058	0.842	3
Mass (kg)	Height (m)	*R. mangle*	2A	171	0.906	2.7960	2.669	2.929	−1.1720	−1.265	−1.079	0.002	0.058	1	0.864	4
Mass (kg)	DBH (cm)	A. germinans	2B	138	0.935	1.8270	1.750	1.908	−0.2561	−0.330	−0.182	1	0.001	0.001	0.937	2
Mass (kg)	DBH (cm)	*L. racemosa*	2B	156	0.969	2.3320	2.267	2.398	−0.7996	−0.851	−0.748	0.001	1	0.01	0.973	3
Mass (kg)	DBH (cm)	*R. mangle*	2B	124	0.966	2.2080	2.137	2.282	−0.4958	−0.555	−0.437	0.001	0.01	1	0.964	0
Mass (kg)	Crown (m)	*A. germinans*	2C	150	0.951	2.5770	2.485	2.671	0.1893	0.131	0.248	CS: 2.539	—	—	0.798	2
Mass (kg)	Crown (m)	*L. racemosa*	2C	149	0.894	2.4030	2.278	2.534	0.4482	0.379	0.518	CS: 2.539	—	—	0.784	1
Mass (kg)	Crown (m)	*R. mangle*	2C	144	0.880	2.6050	2.459	2.759	0.2922	0.218	0.366	CS: 2.539	—	—	0.787	1
Height (m)	DBH (cm)	*A. germinans*	2D	81	0.629	0.566	0.494	0.648	0.292	0.208	0.377	1	0.004	0.01	0.813	0
Height (m)	DBH (cm)	*L. racemosa*	2D	119	0.868	0.721	0.675	0.771	0.266	0.225	0.306	0.004	1	0.28	0.830	1
Height (m)	DBH (cm)	*R. mangle*	2D	120	0.881	0.686	0.645	0.731	0.309	0.275	0.343	0.01	0.28	1	0.875	4
Leaves (kg)	Mass (kg)	*A. germinans*	2E	118	0.956	0.737	0.709	0.766	−0.858	−0.904	−0.812	1	0.751	0.001	0.869	4
Leaves (kg)	Mass (kg)	*L. racemosa*	2E	163	0.929	0.730	0.701	0.761	−1.007	−1.048	−0.967	0.751	1	0.001	0.927	6
Leaves (kg)	Mass (kg)	*R. mangle*	2E	171	0.906	0.852	0.813	0.893	−0.947	−0.998	−0.895	0.001	0.001	1	0.881	1
Leaves (kg)	DBH (cm)	*A. germinans*	2F	81	0.861	1.368	1.258	1.487	−1.123	−1.247	−0.999	1	0.001	0.001	0.786	0
Leaves (kg)	DBH (cm)	*L. racemosa*	2F	119	0.829	1.782	1.653	1.922	−1.681	−1.795	−1.568	0.001	1	0.311	0.894	0
Leaves (kg)	DBH (cm)	*R. mangle*	2F	120	0.822	1.883	1.744	2.034	−1.417	−1.533	−1.302	0.001	0.311	1	0.815	0
Leaves (kg)	Height (m)	*A. germinans*	2G	118	0.827	2.442	2.262	2.636	−1.846	−1.990	−1.703	1	0.021	0.589	0.734	6
Leaves (kg)	Height (m)	*L. racemosa*	2G	163	0.860	2.169	2.046	2.298	−2.051	−2.145	−1.956	0.021	1	0.02	0.710	0
Leaves (kg)	Height (m)	*R. mangle*	2G	171	0.894	2.383	2.267	2.504	−1.946	−2.030	−1.861	0.589	0.02	1	0.781	0
Crown (m)	DBH (cm)	*A. germinans*	2H	73	0.819	0.734	0.664	0.812	−0.238	−0.319	−0.157	CS: 0.7702	—	—	0.650	—
Crown (m)	DBH (cm)	*L. racemosa*	2H	105	0.796	0.826	0.757	0.903	−0.392	−0.454	−0.330	CS: 0.7702	—	—	0.742	4
Crown (m)	DBH (cm)	*R. mangle*	2H	93	0.833	0.748	0.687	0.814	−0.239	−0.293	−0.185	CS: 0.7702	—	—	0.623	2
Crown (m)	Height (m)	*A. germinans*	2I	150	0.819	1.230	1.148	1.317	−0.514	−0.570	−0.458	1	0.855	0.01	0.667	1
Crown (m)	Height (m)	*L. racemosa*	2I	149	0.836	1.241	1.162	1.326	−0.781	−0.841	−0.720	0.855	1	0.003	0.545	3
Crown (m)	Height (m)	*R. mangle*	2I	144	0.847	1.077	1.010	1.150	−0.558	−0.609	−0.508	0.01	0.003	1	0.609	5
Crown (m)	Leaves (kg)	*A. germinans*	2 J	110	0.928	0.519	0.493	0.546	0.360	0.329	0.390	1	0.03	0.001	0.725	0
Crown (m)	Leaves (kg)	*L racemosa*	2 J	149	0.838	0.569	0.533	0.608	0.392	0.346	0.438	0.03	1	0.001	0.816	9
Crown (m)	Leaves (kg)	*R mangle*	2 J	144	0.884	0.451	0.426	0.477	0.321	0.289	0.353	0.001	0.001	1	0.840	2
Roots (kg)	DBH (cm)	*R mangle*	S1A	106	0.843	2.679	2.480	2.893	−1.633	−1.806	−1.459	—	—	—	0.884	—
Roots (kg)	Mass (kg)	*R mangle*	S1B	106	0.924	1.209	1.146	1.276	−1.012	−1.105	−0.920	—	—	—	0.932	—
Roots (kg)	Height (m)	*R mangle*	S1C	106	0.813	3.709	3.410	4.034	−2.637	−2.903	−2.370	—	—	—	0.795	—
Roots (kg)	Leaves (kg)	*R mangle*	S1D	106	0.872	1.347	1.257	1.444	0.431	0.356	0.505	—	—	—	0.799	—
Roots (kg)	Crown (m)	*R mangle*	S1E	79	0.805	4.412	3.993	4.876	−1.207	−1.412	−1.003	—	—	—	0.788	—

*Note:* The Y and X variable are given in the first and second column, followed by the data grouping (3rd column), applicable figure (4th column), sample size (*n*, 5th column), regression results (columns 6–12), the results from slope similarity test (columns 13–15), the mean intrasite *R* ^2^ value (column 16) and the number of significant interactions with climate for that rows relationship. CS indicates that the three species had a common slope for that relationship, with the slope value following the colon. Note that the results from the slope similarity test are symmetric about the diagonal.

The mean interspecific *R* ^2^ across all 10 relationships is 0.858 (Table 1). The mean intraspecific *R* ^2^ across all relationships and sites is 0.870 (Table 1). Across all intraspecific, within site regressions, the mean intraspecific *R* ^2^ is 0.820. Across all intraspecific, within site regressions, the mean intraspecific *R* ^2^ *'s* for *A. germinans*, *L. racemosa*, and *R. mangle* are 0.791, 0.817, and 0.826, respectively (Tables S3–S5). The relationship with the highest mean, across site, intraspecific *R* ^2^ (0.957) is *mass‐DBH* (Table 1). The relationship with the lowest mean, across site, intraspecific *R* ^2^ (0.816) is *crown*‐*DBH* (Table 1).

In all, 46.5% (33/71) of the pairwise site comparisons for *A. germinans* were not different from each other; 37.9% (33/87) of the pairwise site comparisons for *L. racemosa* were not different from each other; and 58.1% (90/155) of the pairwise site comparisons for *R. mangle* were not different from each other. Across all species and sites, 49.8% (156/313) of the pairwise comparisons were not different from one another. Three of the relationships we examined had a common slope across sites, *crown* versus *leaves* for *A. germinans* (Table S3), *crown* versus *DBH* for *L. racemosa* (Table S4), and *crown* versus *DBH* for *R. mangle* (Table S5).

For relationships involving roots for *R. mangle* (the only species with data for multiple sites), across all sites 74.4% (32/43) of the relationships were not different from one another (Table S5). This was driven largely by the fact that 3 of the 5 allometric relationships had a common slope across sites (Table S5).

**FIGURE 6:**
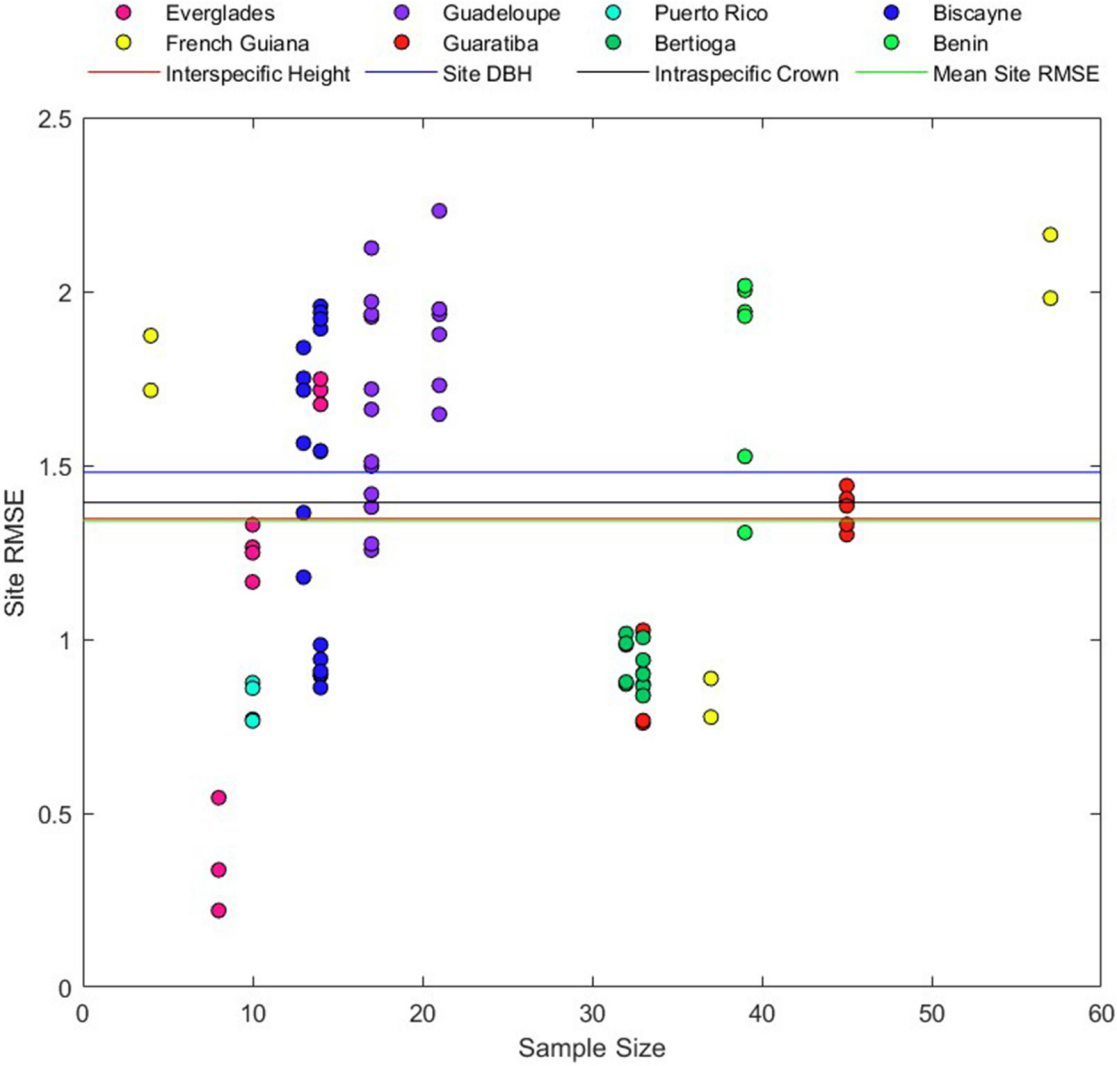
Site level RMSE values plotted as a function of sample size. Symbol colors correspond to the sites, and the four horizontal lines correspond to the lowest RMSE across sites for each allometric variable (DBH, Height, Crown, see Table 2), and the mean observed site specific RMSE (mean of all data points in plot). The values for each allometric relationship and whether they are inter or intraspecific within each site are given in Table S8. If we exclude values below recommended sample size thresholds (e.g., *n* = 30) only ¼ (22/88) of the sites have RMSE values below the best across‐site interspecific value (red line).

The results from the RMSE comparisons are provided in Tables 2 and S8, Figure 6, with the values for each individual tree predicted by each model in Table S2. Across all sites, using *height*, *DBH* and *crown* as predictor variables, the lowest RMSE differed for each variable and relationship. Using *height* as a predictor, the interspecific regression had the lowest RMSE. Using *DBH* as a predictor, the site level regressions had the lowest RMSE, and using *crown* as a predictor, intraspecific regression had the lowest RMSE. Across all relationships the lowest RMSE was for interspecific height.

**TABLE 2:** Root mean squared error estimates for the three predictor variables.

Predictor	Relationship	RMSE log scale (kg)	RMSE linear scale (kg)
**Height**	**Interspecific**	**1.347**	**22.214**
Height	Intraspecific	1.371	23.507
Height	Site	1.441	27.628
DBH	Interspecific	1.525	33.534
DBH	Intraspecific	1.492	31.054
**DBH**	**Site**	**1.480**	**30.175**
Crown	Interspecific	1.402	25.260
**Crown**	**Intraspecific**	**1.393**	**24.709**
Crown	Site	1.497	31.377

*Note:* The first column contains the predictor variable (*height*, *DBH* or *crown*), the second column is the relationship examined either interspecific (across all species and sites), intraspecific (within species and across all sites), or site (within species and site). The relationship with the lowest RMSE differs for each predictor variable (highlighted in bold).

RMSE values within sites exhibited high variance, ranging from 0.22 to 2.23, with a mean value of 1.34, close to the values observed across sites (Table 2). Within site RMSE values were clearly linked to sample size (Figure 6), with the lowest values found in sites with fewer than 10 individuals.

Of the 696 (9.2%) bivariate relationships between empirical allometric slopes and climatic variables, 64 had significant relationships (Table S6, Figures S2–S65). The average number of data points (sites) for the significant relationships was 4.79 (Table S6). Fifteen of the significant relationships were for *A. germinans*, 30 for *L. racemosa*, and 19 for *R. mangle*. The allometric relationships with the most significant interactions with climate were *leaves* versus *mass* (11 significant) followed by *crown* versus *leaves* (11 significant), and the relationship with the least was leaves versus *mass* with no significant interactions (Table S6). The highest number of significant relationships (of 30 possible) for any single climatic variable was 7 for minimum temperature followed by 6 for minimum temperature of the coldest month.

Of the 64 significant relationships between climatic variables and site allometric slopes, 27 involved *height*, 27 involved *mass*, 30 involved *crown*, 28 involved leaves, and 16 involved *DBH* (Table S6).

Of the 696 (11.8%) bivariate relationships between empirical allometric *intercepts* and climatic variables, 82 had significant non‐zero relationships (Table S7, Figures S63–S143). The climatic variable with the highest number of significant relationships was Solar Radiation with 11 followed by Minimum Temperature with 9.

## 4 Discussion

The three mangrove species we have considered here are prominent members of mangrove communities throughout the AEP. As shown in Figure 1, they have largely similar distributions, with *A. germinans* having the highest northern latitudinal range, and *R. mangle* and *L. racemosa* the highest southern latitudinal range. Their ranges otherwise overlap strongly with the exception that *R. mangle* is uncommon on the west coast of the Americas. As previously mentioned, they all tend to be abundant in local mangrove communities throughout their ranges, although any one of the three may be locally dominant, particularly *A. germinans* or *R. mangle*.

Within a site, they tend to occupy different positions across the intertidal zone. *R. mangle* is typically found furthest into the water because of its ability to withstand wave disturbance due to its interwoven mesh of prop roots. *L. racemosa* and *A. germinans* are typically found a bit more inland. These coarse trends are not always obeyed but are commonly observed.

### 4.1 Allometric Trends

Of the linear dimensions (*height*, *crown*, *DBH*) used to estimate biomass, mangrove height has probably been the most frequently utilized due to the ease with which it is estimated in the field or using remote sensing approaches (Feliciano et al. 2017; Navarro et al. 2020; Simard et al. 2006). Although this is understandable given the challenges in measuring other plant dimensions remotely (*DBH*, *crown*), our work shows that biomass regressions based on *DBH* have the highest correlation coefficients with a mean *R* ^2^ of 0.957 for intraspecific regression (Table 1) and a mean *R* ^2^ of 0.958 for intrasite regressions (Tables S3–S5). The relationship between *height* and *mass* has lower correlation coefficients, but *height* still explains considerable variability in *mass* with a mean *R* ^2^ of 0.916 for intraspecific regressions (Table 1) and a mean *R* ^2^ of 0.850 for intrasite regressions (Tables S3–S5).

The investment of a growing mangrove trees' mass in growing more vertically (up) or more horizontally (spreading out) will depend in part of the light environment it is exposed to. In tall closed canopy forests, growing trees will need to invest in reaching the upper canopy layer to receive sufficient light. In contrast in areas where maximum height is limited, or that are exposed to regular disturbance (Krauss and Osland 2020), light will be less of a limiting factor, and growing trees can invest more in spreading canopies to increase leaf light interception. Thus, it is not surprising that we see more variability in the scaling of plant height or canopy spread with other measures such as *mass* or *DBH* (Table 1). In contrast, the DBH is typically the point in the growing tree which represents the amount of biomechanical and conducting tissue (xylem, phloem) required to link above and below ground resource flow, and support the above ground tree mass. It is not surprising then, that the relationship between DBH and above ground mass is more tightly constrained.

Despite the higher *R* ^2^ values for *mass* to *DBH* regressions, across sites the RMSE value is lowest for *mass* as a function of tree *height* interspecifically (Table 2). Moreover, the RMSE values for *DBH* and *crown* as biomass predictors are only marginally higher for interspecific relationships compared to intraspecific or site‐specific relationships (Table 2).

Although some of the within site RMSE values we observed were indeed lower than those found across sites (Table S8, Figure S2), most of those were from sites with sample sizes lower than what has been recommended for studies attempting to use allometric models to predict biomass (Chave et al. 2004; Roxburgh et al. 2015), although in fairness some of these data were collected before these sample size recommendations were published. For example, Roxburgh et al. (2015) recommend collecting between 17 and 95 individuals to achieve biomass predictions with a standard deviation within 5% of the mean. Paul et al. (2018) recommend collecting at least 50 individuals. In addition, the mean site‐specific RMSE value (1.341) was almost identical to the RMSE for the interspecific‐across site relationship (1.347, Table 2).

Overall, our findings suggest that in the absence of quality site specific data (Njana et al. 2016), the use of interspecific or common equations, may have utility in predicting biomass across the AEP. This will be of particular interest to those using remote sensing approaches using UAV, airborne or satellite‐based approaches to estimate stand level biomass (Feliciano et al. 2017; Heumann 2011; Navarro et al. 2020; Yin and Wang 2019). While identifying individual trees from remote sensed data has its challenges, particularly for understory trees (Simard et al. 2006; Zhou et al. 2010), our results suggest that to the extent that individual trees can be identified, there is support for the use of common allometric equations to predict their mass.

Across all species, sites, and allometric relationships, about half of the pairwise relationships we examined were not different from each other (Tables S3–S5). *R. mangle* growth was the most similar across sites (58.1%), and *L. racemosa* the least (37.9%). Our ability to draw conclusions from these data are tempered by the fact that the range of the data differed between sites (Figures 3, 4, 5 and S1). For sites such as within dwarf mangrove forests (Ross et al. 2001), there are no individuals above a certain size available for collection, so the constrained data range is unavoidable. However, for sites with large individuals, if possible, the efforts should be made to include the small end of the size spectrum in data collection efforts.

### 4.2 Climatic Trends

It has long been understood that the growth and form of these species is strongly influenced by climate and soil conditions at local and global scales (Lugo and Snedaker 1974; Rovai et al. 2021). At a global scale, mangrove height and annual litterfall have been linked to latitude and temperature, with taller and more productive trees at lower latitudes (Saenger and Snedaker 1993). At local scales, increasing soil salinity is linked to increased root to shoot ratios (Saintilan 1997), decreased net primary productivity and growth efficiency (Sherman, Fahey, and Martinez 2003), and decreased basal area and decreasing height (Cintron et al. 1978).

We see clear and strong relationships between climate indices and the slopes and intercepts of the allometric relationships we examined (Tables 3, S6 and S7, Figures S2–S147). The variables that explain the most variance in allometric slopes and intercepts are those having to do with minimum temperature (including variables influenced by minimum temperature such as mean temperature, etc.), and to a lesser extent precipitation (Table 3). As a number of the sites in our data compilation come from areas toward the latitudinal limits of mangrove species distributions (Figure 1, Table S1), it is not surprising that we see an effect from temperature on allometric slopes and intercepts across sites. Moreover, this is consistent with several studies that have established temperature as the variable limiting mangrove growth and poleward expansion (Cavanaugh et al. 2014; Osland et al. 2016; Saintilan et al. 2014), or temperature and precipitation as limiting species ranges globally (Michael J. Osland et al. 2017).

**TABLE 3:** Variance explained by climatic variables.

BioClim parameter	Mean variance explained		Non‐zero	
	Slopes	Intercepts	Slopes	Intercepts
Min temperature of coldest month	0.430	0.463	6	6
Minimum temp	0.427	0.479	7	9
Precipitation seasonality	0.417	0.330	5	3
Average temp	0.402	0.460	1	8
Temperature annual range	0.395	0.370	4	4
Mean temperature of coldest quarter	0.392	0.431	4	4
Solar radiation	0.387	0.608	4	11
Annual mean temperature	0.386	0.427	1	6
Mean temperature of driest quarter	0.381	0.434	4	3
Temperature seasonality	0.375	0.369	5	6
Isothermality	0.367	0.413	4	3
Mean temperature of warmest quarter	0.351	0.418	3	4
Mean temperature of wettest quarter	0.326	0.293	3	2
Precipitation	0.306	0.275	2	1
Precipitation of driest month	0.303	0.236	3	2
Precipitation of driest quarter	0.287	0.213	2	2
Max temperature of warmest month	0.282	0.402	2	7
Annual precipitation	0.277	0.244	2	0
Maximum temp	0.261	0.169	1	0
Precipitation of warmest quarter	0.221	0.272	0	1
Precipitation of wettest quarter	0.181	0.149	0	0
Mean diurnal range	0.157	0.136	0	0
Precipitation of wettest month	0.155	0.164	1	0
Precipitation of coldest quarter	0.116	0.168	0	0

*Note:* The amount of variance explained, across all allometric relationships, by each of the 24 climatic variables we examined (derived from Tables S6 and S7), and the number of relationships with non‐zero OLS regression slopes. The first column is the list of WorldClim parameters. The second column is the amount of variance explained for slope differences, the third column is the amount of variance explained for intercept differences. The fourth and fifth columns are the number of non‐zero relationships for slopes and intercepts (see 2 Methods). The table has been sorted by the second column in descending order.

### 4.3 Moving Forward

The most accurate way to estimate biomass is through the development of allometric equations, as exemplified in the studies we have collated, and as we have shown here. Site specific equations can be developed that outperform common equations (Njana et al. 2016), but as our analysis demonstrates this is not always the case. Future efforts should prioritize collecting sample sizes within recommended ranges (Paul et al. 2018; Roxburgh et al. 2015) to increase the likelihood of developing improved site‐specific estimates. Unfortunately, such approaches require the destructive harvesting of individuals and mangrove species are appropriately protected in many of the countries in which they grow. As the emphasis on accurate blue carbon accounting grows, managers and governing agencies may need to weigh the costs of destructive harvesting of a relatively small number of individual trees against the benefits of improved estimates of standing biomass and carbon storage. There may be opportunities to take advantage of planned development within mangrove forests, i.e., identifying sites where development has been approved and trees are going to be cleared anyway, by coordinating with state and federal agencies responsible for approving such projects.

Some groups have explored the use of volume estimation using quantitative structural models derived from high resolution point clouds from terrestrial laser scanners (Feliciano, Wdowinski, and Potts 2014). While such approaches hold promise, the density at which mangroves grow, and the challenges of working in them make such approaches unlikely to yield large scale estimates in the near future. Mangrove stands, particularly those subject to frequent disturbance, have overlapping branches and leaves growing throughout their canopies which cause occlusion in lidar scans, rendering such approaches difficult. Methodological advances in scan acquisition, point density, and coverage may yield improved estimates in the future, but at present destructive harvesting probably remains the best approach for higher accuracy biomass estimates.

As shown in Figures 3, 4, 5 and S1, if allometric data are collected, gathering individuals across the entire size range present is advised. In areas where mangroves do not grow tall, particularly in dwarf mangrove forests and/or at latitudinal extremes, the upper end of the size spectrum may not be available for collection. However, the smaller end of the size spectrum should be available in most, if not all areas. The absence of data at the small end of allometric regressions can influence slope fitting, and thus complicate efforts to make inferences about the effect of site and climate on regression parameters.

Our work also illustrates the comparative lack of root data. Only one species, *R. mangle*, had root data for more than one site, obviating the need for interspecies comparisons. Future allometric work should prioritize the collection of below ground root mass to facilitate the types of analyses we have performed here.

## 5 Conclusions

We have shown that allometric relationships within and across three mangrove species tend to have high *R* ^2^ values with *mass* to *DBH* typically the highest. However, looking at RMSE we find that *mass* to *height* has the lowest value, and that the RMSE of other interspecific relationships are marginally higher than intraspecific relationships, across sites (Table 2). Within sites we find that the mean RSME is similar to that found looking across sites and while a handful of our sites with adequate sample sizes have lower RMSE values, larger sample size is not a guarantee of improved RMSE values (Figure 6). This suggests that the use of common equations to estimate above ground biomass may be justified depending on the question at hand and the investigators tolerance for error. We have also shown that temperature and to a lesser extent, precipitation, have a significant influence on the growth allometries of these species (Table 3). Future work with more and improved data will help illuminate the extent to which these patterns hold true.

## Author Contributions

**Charles A. Price:** conceptualization (lead), formal analysis (lead), investigation (lead), methodology (lead), project administration (lead), visualization (lead), writing – original draft (lead), writing – review and editing (lead). **Benjamin Branoff:** formal analysis (supporting), methodology (supporting), project administration (equal), writing – original draft (supporting), writing – review and editing (supporting). **Karen Cummins:** investigation (supporting), writing – original draft (supporting), writing – review and editing (supporting). **Romain Glèlè Kakaï:** data curation (lead), formal analysis (supporting), writing – original draft (supporting), writing – review and editing (supporting). **Danielle Ogurcak:** investigation (supporting), writing – original draft (supporting), writing – review and editing (lead). **Monica Papeș:** formal analysis (supporting), project administration (lead), writing – original draft (supporting), writing – review and editing (supporting). **Michael Ross:** data curation (lead), methodology (supporting), writing – original draft (supporting), writing – review and editing (supporting). **Kevin R. T. Whelan:** data curation (lead), methodology (supporting), writing – original draft (supporting), writing – review and editing (supporting). **Todd A. Schroeder:** investigation (supporting), project administration (lead), writing – original draft (supporting), writing – review and editing (supporting).

## Conflicts of Interest

The authors declare no conflicts of interest.

## Supporting information


Figures S1–S147.

Tables S1–S8.

## Data Availability

The data used in this manuscript are contained in Appendix Table S2.
