# Supplementary material for: Global Data Compilation Across Climate Gradients Supports the Use of Common Allometric Equations for Three Transatlantic Mangrove Species
Source: Ecol Evol. 2024 Nov 20;14(11):e70577. doi: 10.1002/ece3.70577 (PMC11578852; doi:10.1002/ece3.70577)
Supplement: Supplementary file 1 — Figures S1–S147. [file ECE3-14-e70577-s002.zip › Figs S2_S65 Slopes.pdf]

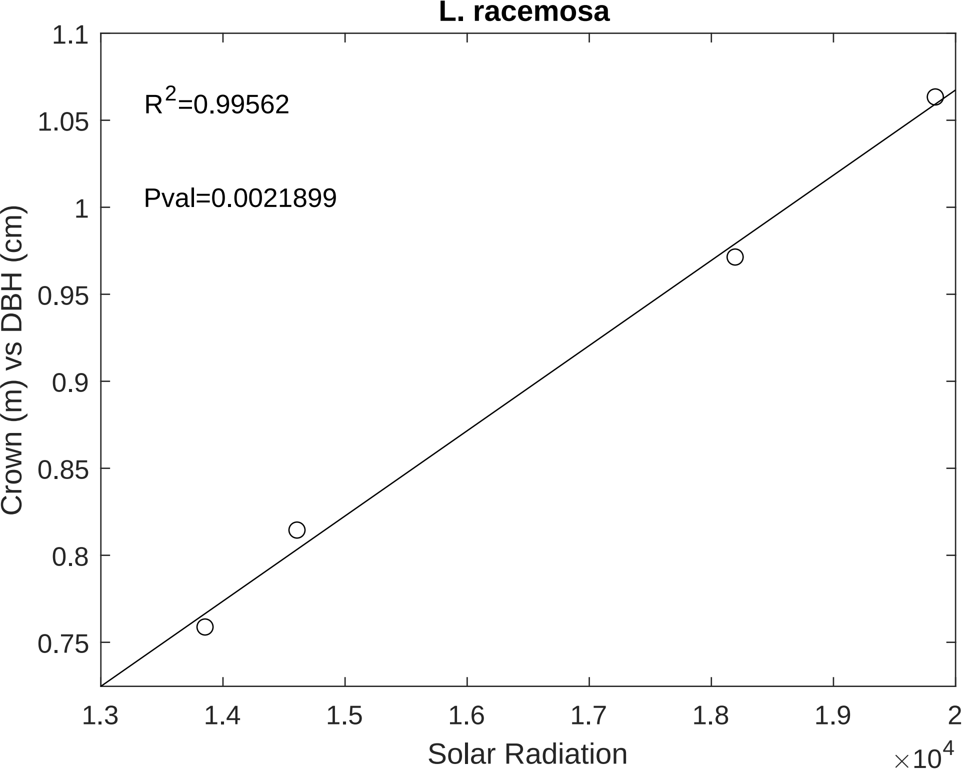
L. racemosa
R^2=0.99562
Pval=0.0021899
Crown (m) vs DBH (cm)
Solar Radiation
$\times 10^4$
1.1
1.05
1
0.95
0.9
0.85
0.8
0.75
1.3
1.4
1.5
1.6
1.7
1.8
1.9
2

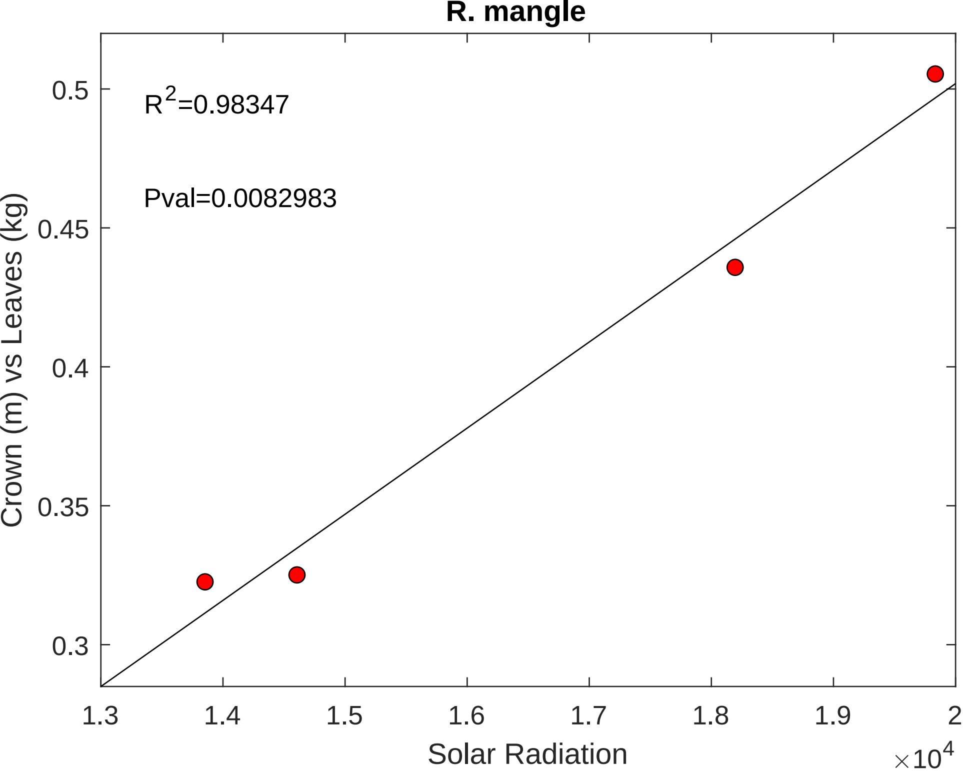

R. mangle
R^2=0.98347
Pval=0.0082983
Crown (m) vs Leaves (kg)
0.3
0.35
0.4
0.45
0.5
1.3
1.4
1.5
1.6
1.7
1.8
1.9
2
Solar Radiation
×10^4

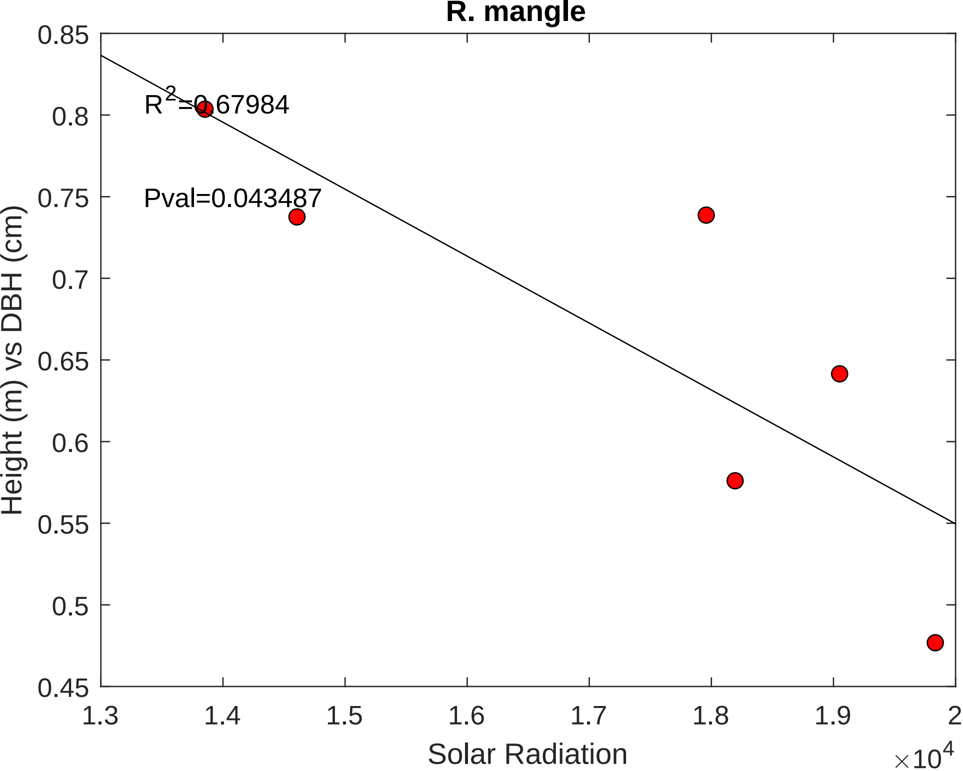
R. mangle
R2=0.67984
Pval=0.043487
Height (m) vs DBH (cm)
Solar Radiation
×10^4
0.85
0.8
0.75
0.7
0.65
0.6
0.55
0.5
0.45
1.3
1.4
1.5
1.6
1.7
1.8
1.9
2

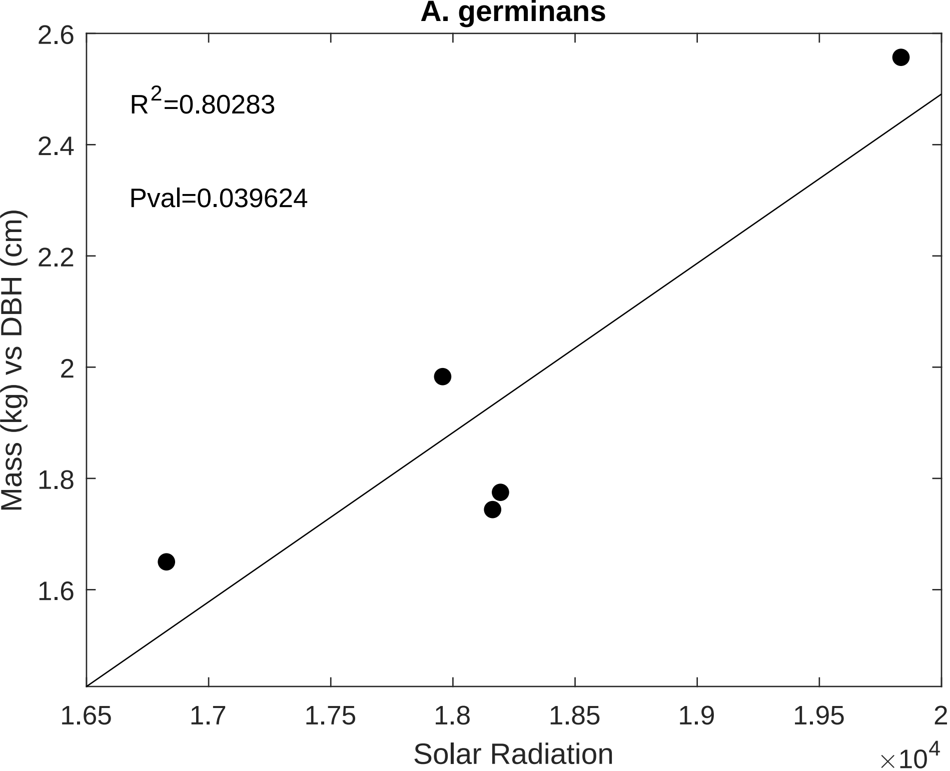
A. germinans
R2=0.80283
Pval=0.039624
Mass (kg) vs DBH (cm)
Solar Radiation
×10^4
2.6
2.4
2.2
2
1.8
1.6
1.65
1.7
1.75
1.8
1.85
1.9
1.95
2

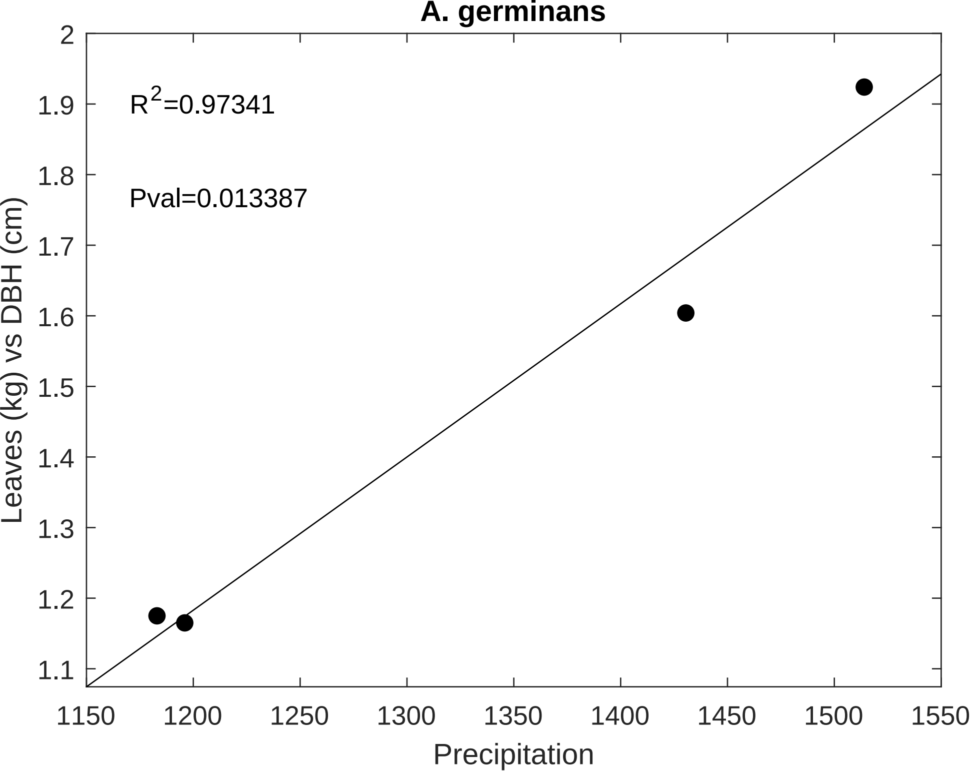

A. germinans
R^2=0.97341
Pval=0.013387
Leaves (kg) vs DBH (cm)
Precipitation
2
1.9
1.8
1.7
1.6
1.5
1.4
1.3
1.2
1.1
1150
1200
1250
1300
1350
1400
1450
1500
1550

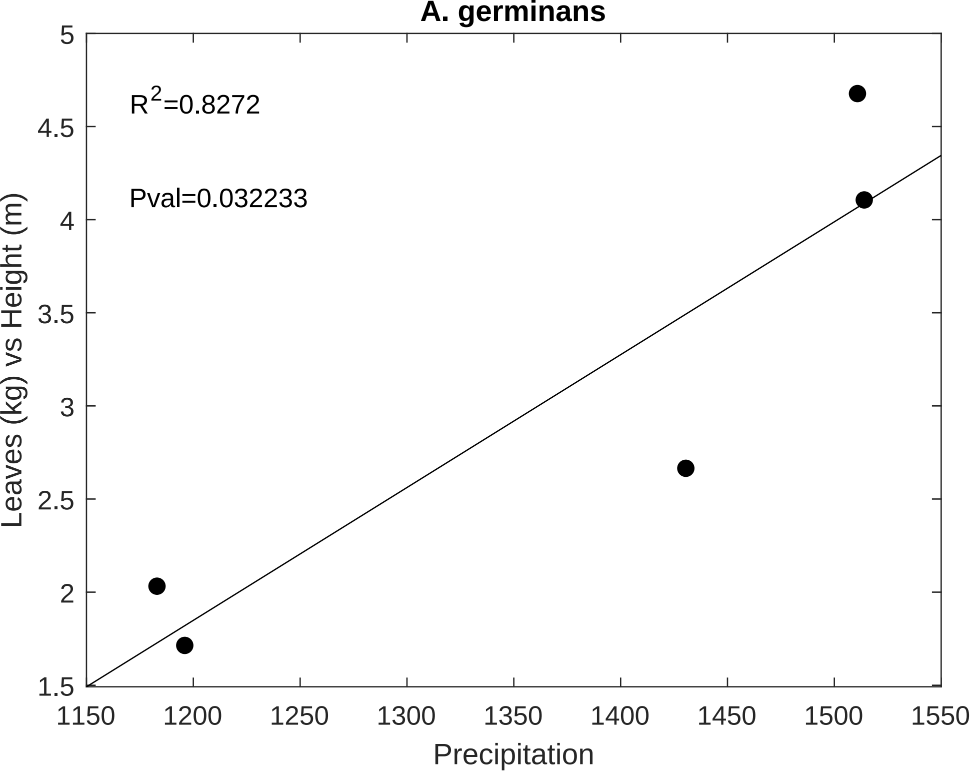
A. germinans
R2=0.8272
Pval=0.032233
Leaves (kg) vs Height (m)
Precipitation
5
4.5
4
3.5
3
2.5
2
1.5
1150
1200
1250
1300
1350
1400
1450
1500
1550

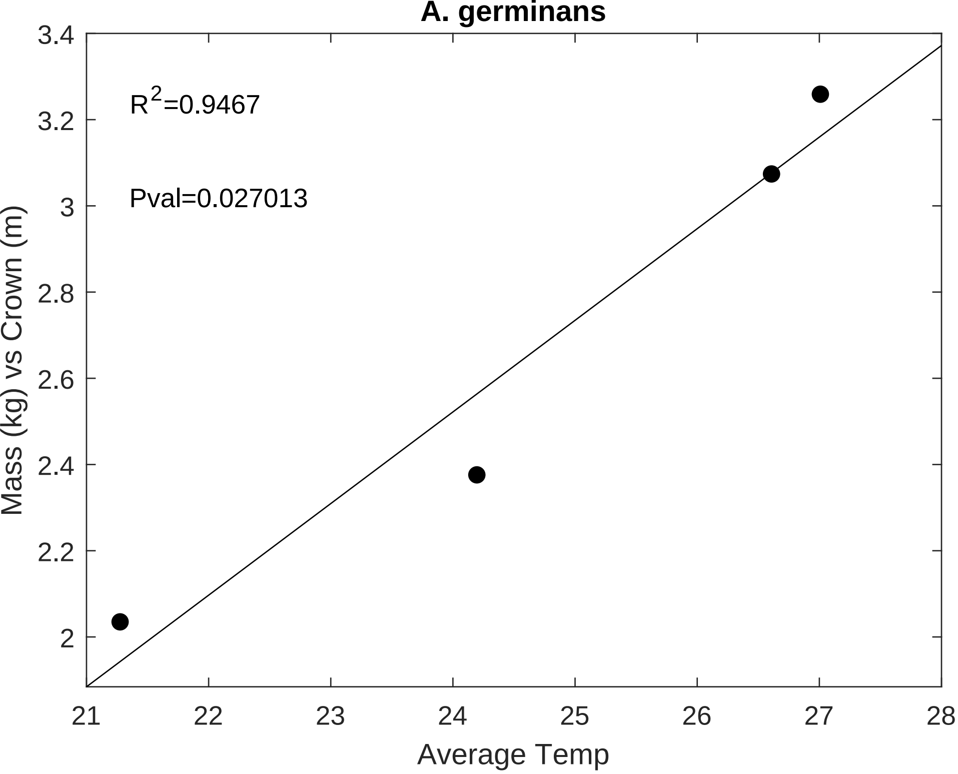
A. germinans
R^2=0.9467
Pval=0.027013
Mass (kg) vs Crown (m)
Average Temp
3.4
3.2
3
2.8
2.6
2.4
2.2
2
21
22
23
24
25
26
27
28

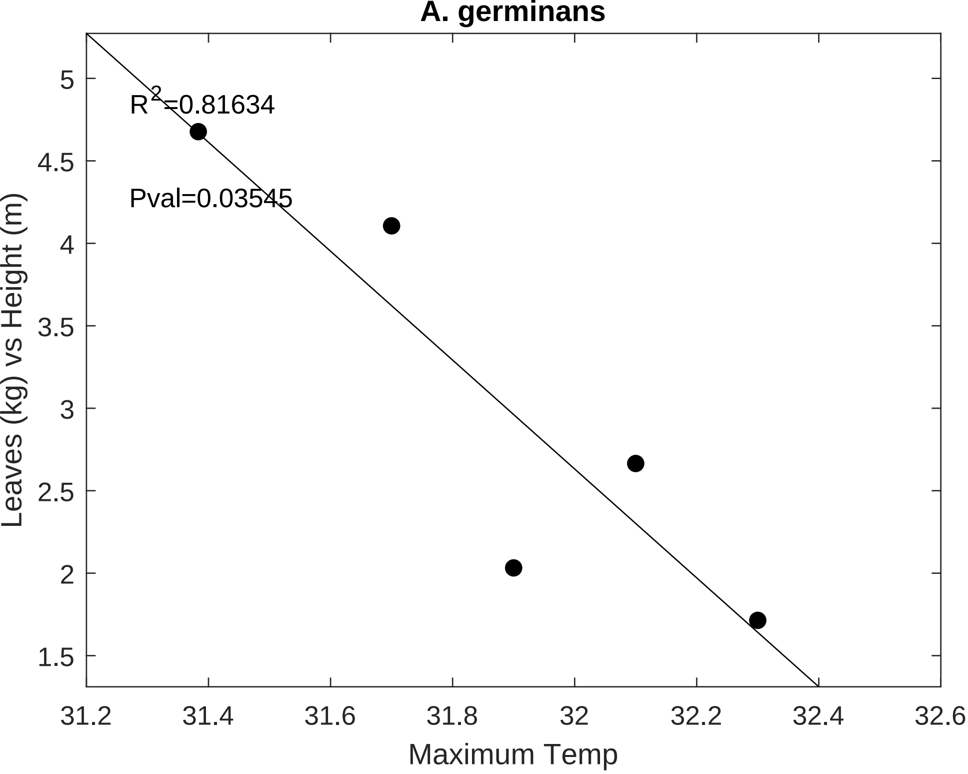
A. germinans
R2=0.81634
Pval=0.03545
5
4.5
4
3.5
3
2.5
2
1.5
Leaves (kg) vs Height (m)
31.2
31.4
31.6
31.8
32
32.2
32.4
32.6
Maximum Temp

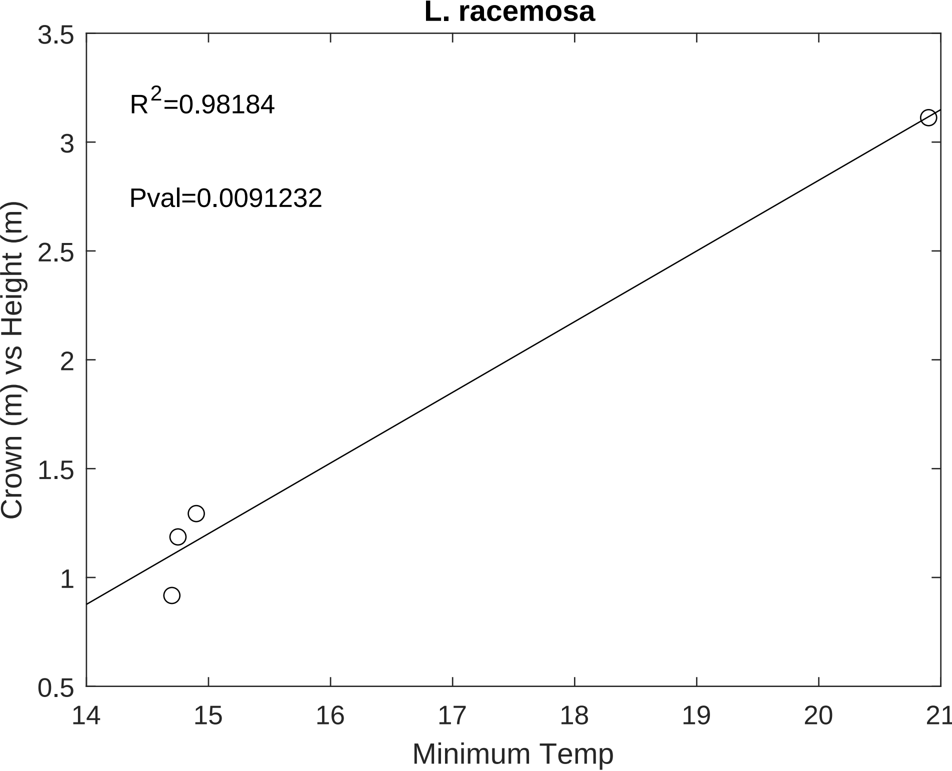
L. racemosa
R2=0.98184
Pval=0.0091232
Crown (m) vs Height (m)
Minimum Temp
3.5
3
2.5
2
1.5
1
0.5
14
15
16
17
18
19
20
21

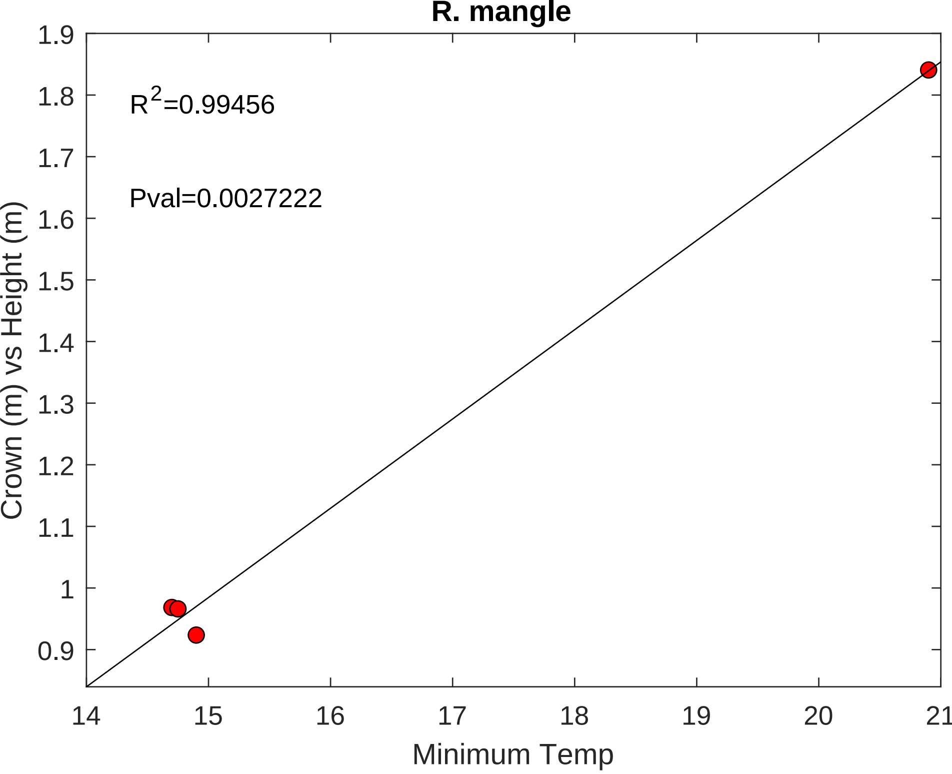

R. mangle
R^2=0.99456
Pval=0.0027222
1.9
1.8
1.7
1.6
1.5
1.4
1.3
1.2
1.1
1
0.9
Crown (m) vs Height (m)
14
15
16
17
18
19
20
21
Minimum Temp

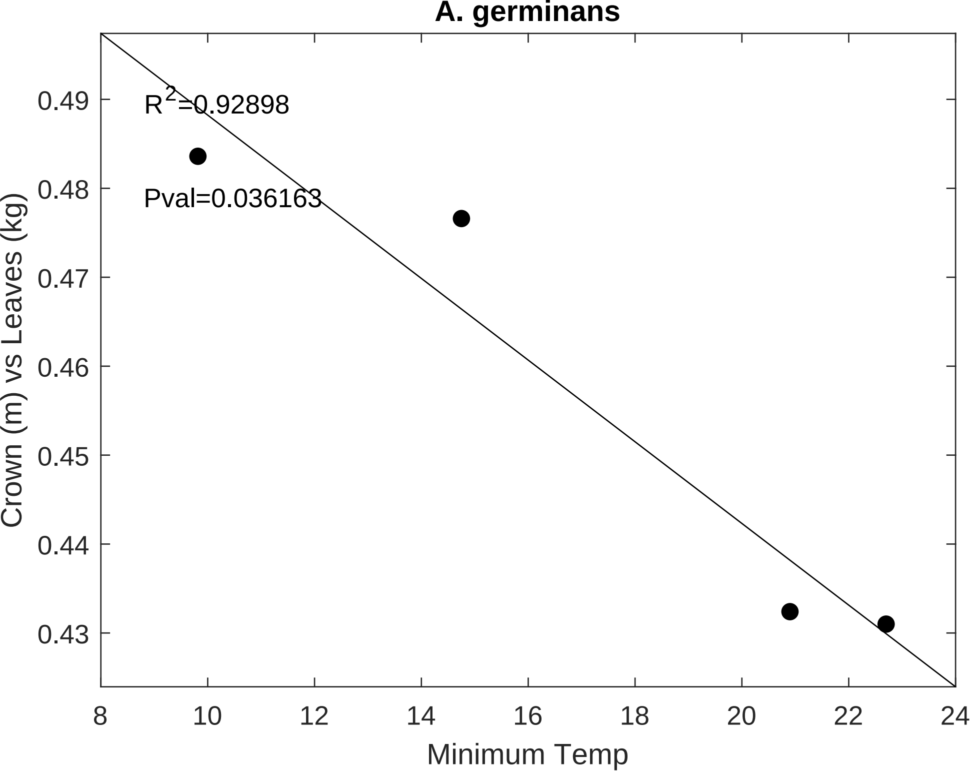
A. germinans
R^2=0.92898
Pval=0.036163
Crown (m) vs Leaves (kg)
0.49
0.48
0.47
0.46
0.45
0.44
0.43
8
10
12
14
16
18
20
22
24
Minimum Temp

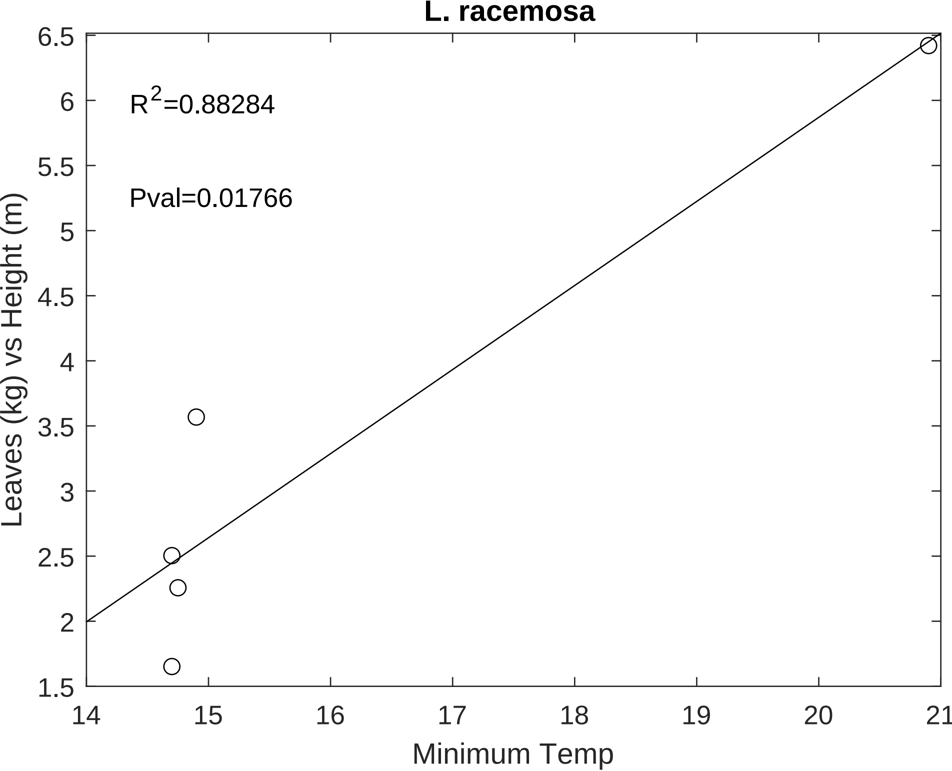
L. racemosa
R^2=0.88284
Pval=0.01766
Leaves (kg) vs Height (m)
Minimum Temp
6.5
6
5.5
5
4.5
4
3.5
3
2.5
2
1.5
14
15
16
17
18
19
20
21

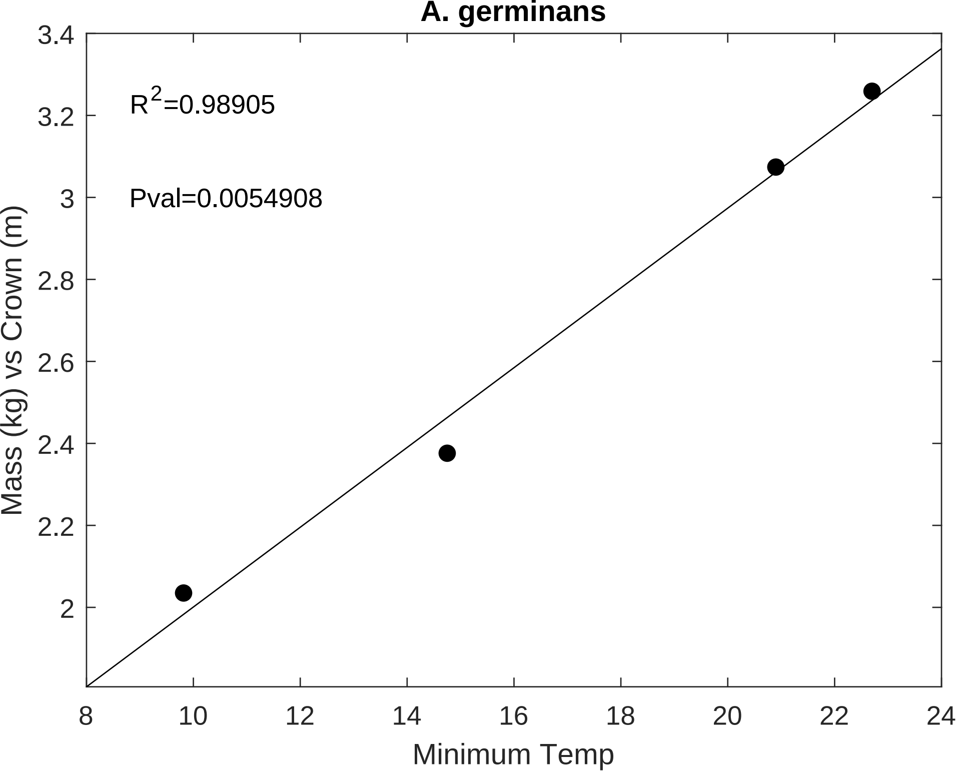
A. germinans
R2=0.98905
Pval=0.0054908
Mass (kg) vs Crown (m)
Minimum Temp
3.4
3.2
3
2.8
2.6
2.4
2.2
2
8
10
12
14
16
18
20
22
24

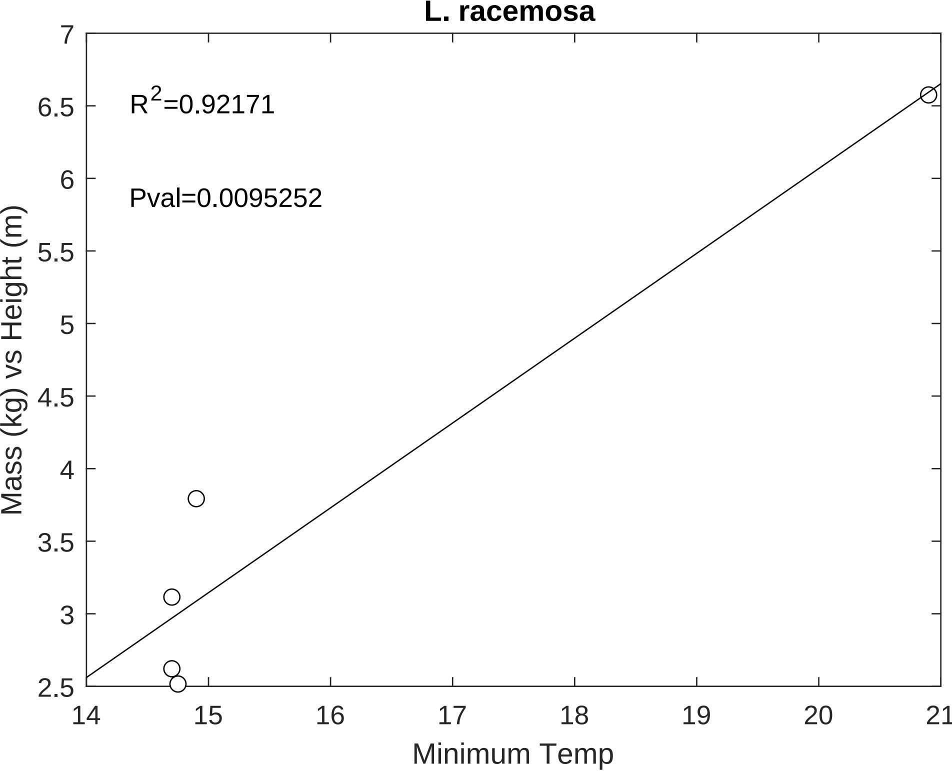
L. racemosa
R2=0.92171
Pval=0.0095252
Mass (kg) vs Height (m)
Minimum Temp
7
6.5
6
5.5
5
4.5
4
3.5
3
2.5
14
15
16
17
18
19
20
21

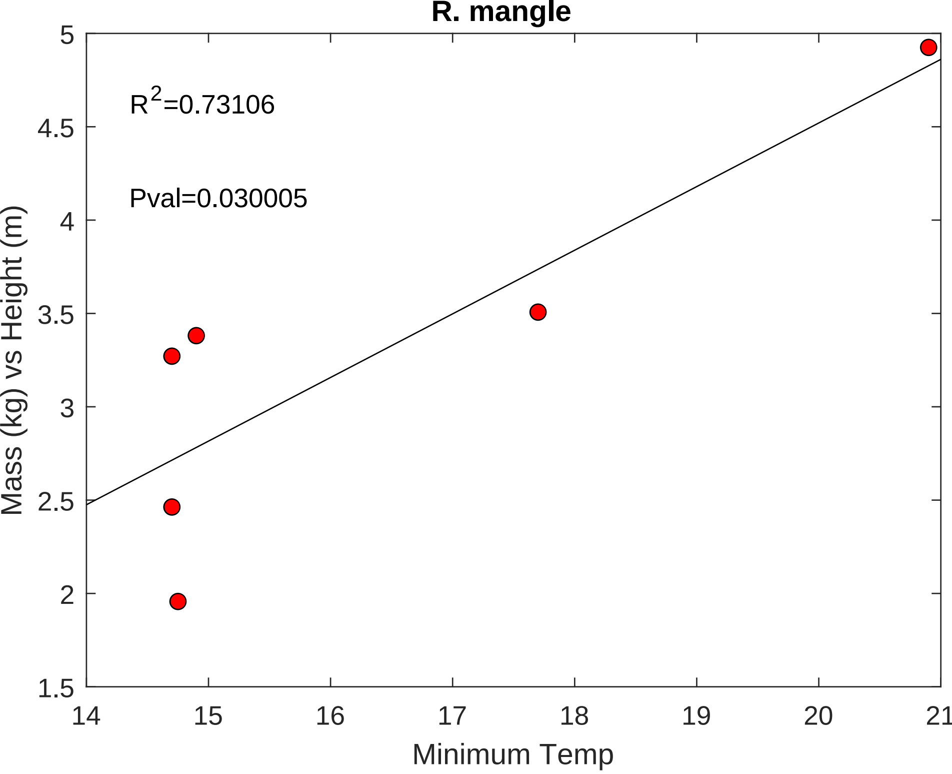
R. mangle
R2=0.73106
Pval=0.030005
Mass (kg) vs Height (m)
Minimum Temp
5
4.5
4
3.5
3
2.5
2
1.5
14
15
16
17
18
19
20
21

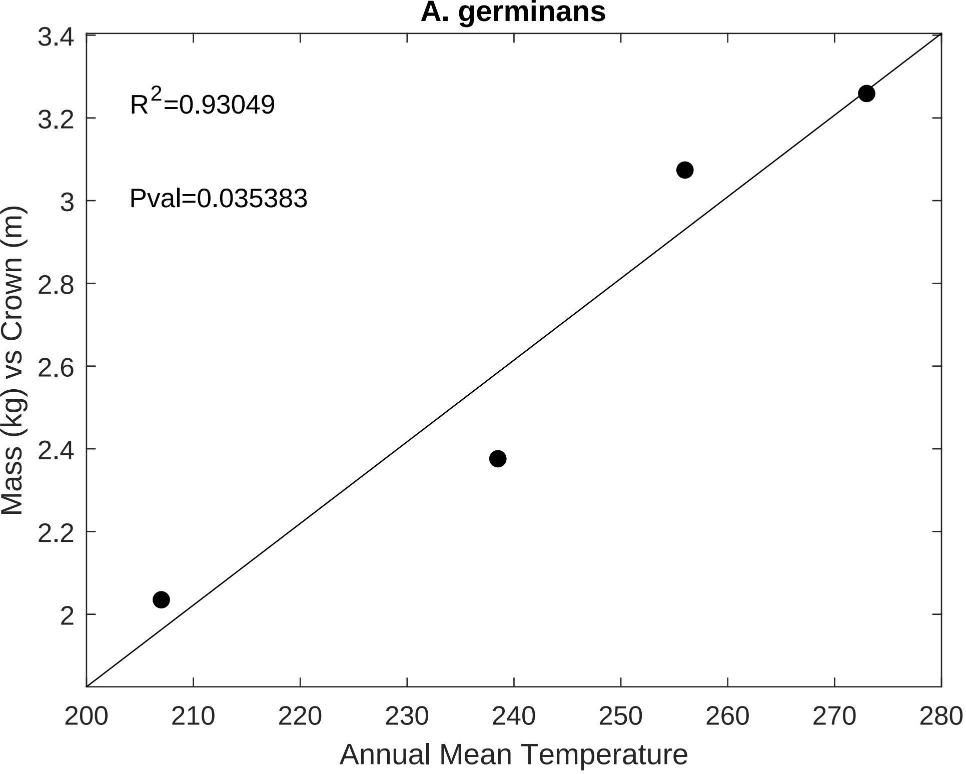
A. germinans
R^2=0.93049
Pval=0.035383
Mass (kg) vs Crown (m)
Annual Mean Temperature
3.4
3.2
3
2.8
2.6
2.4
2.2
2
200
210
220
230
240
250
260
270
280

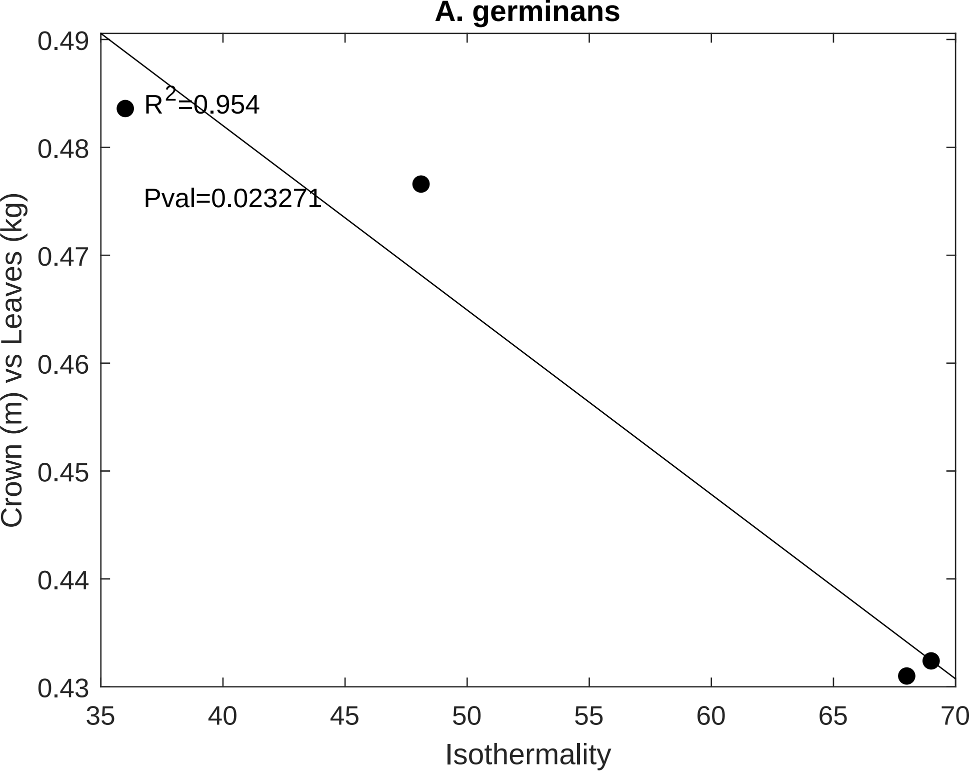
A. germinans
R^2=0.954
Pval=0.023271
Crown (m) vs Leaves (kg)
Isothermality
0.49
0.48
0.47
0.46
0.45
0.44
0.43
35
40
45
50
55
60
65
70

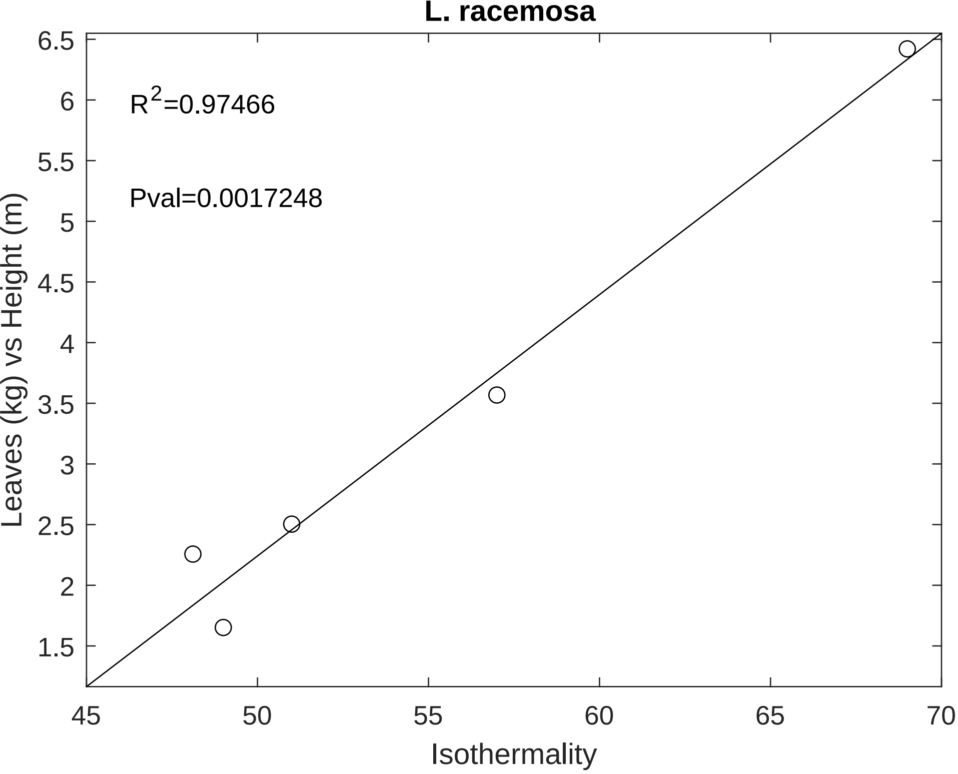
L. racemosa
R2=0.97466
Pval=0.0017248
Leaves (kg) vs Height (m)
Isothermality
6.5
6
5.5
5
4.5
4
3.5
3
2.5
2
1.5
45
50
55
60
65
70

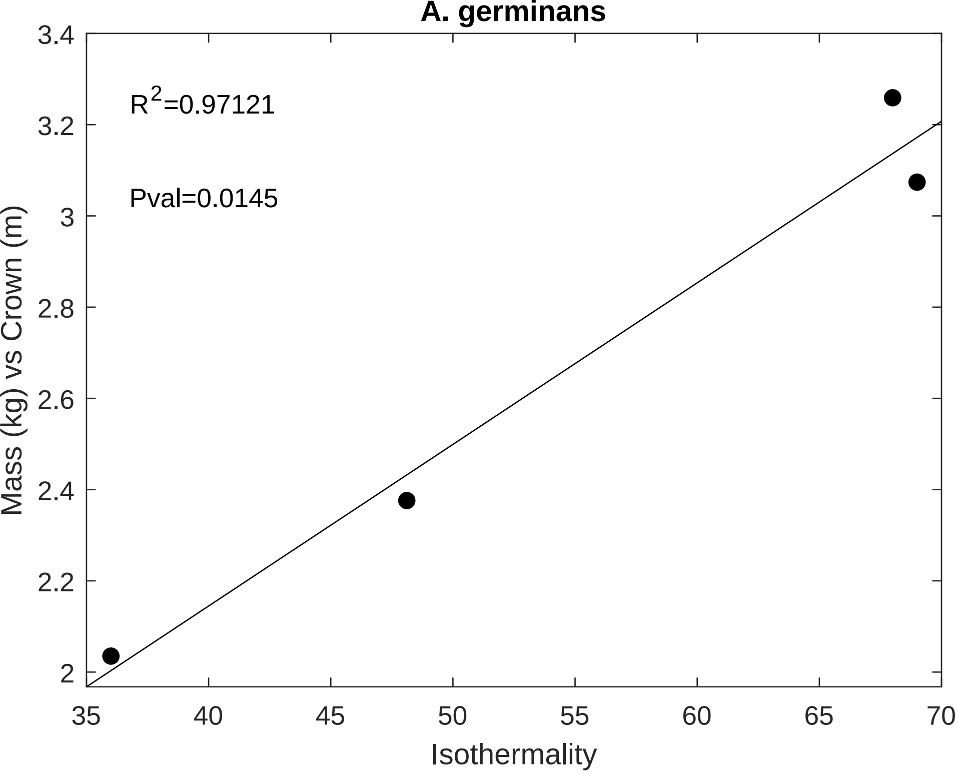
A. germinans
R^2=0.97121
Pval=0.0145
Mass (kg) vs Crown (m)
Isothermality
3.4
3.2
3
2.8
2.6
2.4
2.2
2
35
40
45
50
55
60
65
70

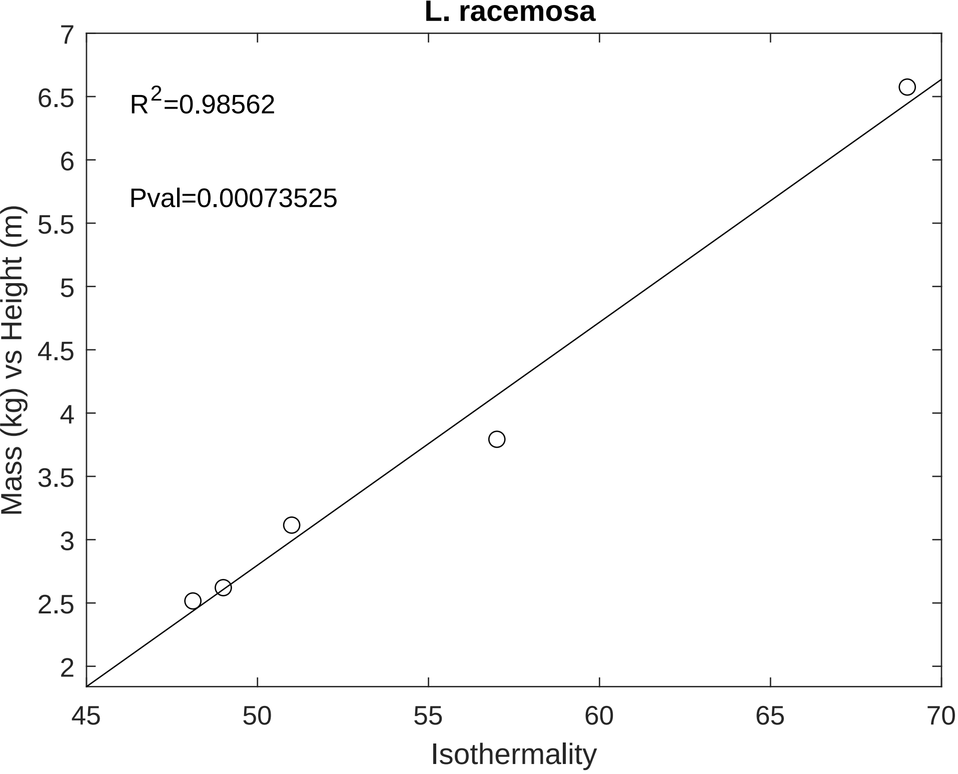

L. racemosa
R^2=0.98562
Pval=0.00073525
Mass (kg) vs Height (m)
Isothermality
7
6.5
6
5.5
5
4.5
4
3.5
3
2.5
2
45
50
55
60
65
70

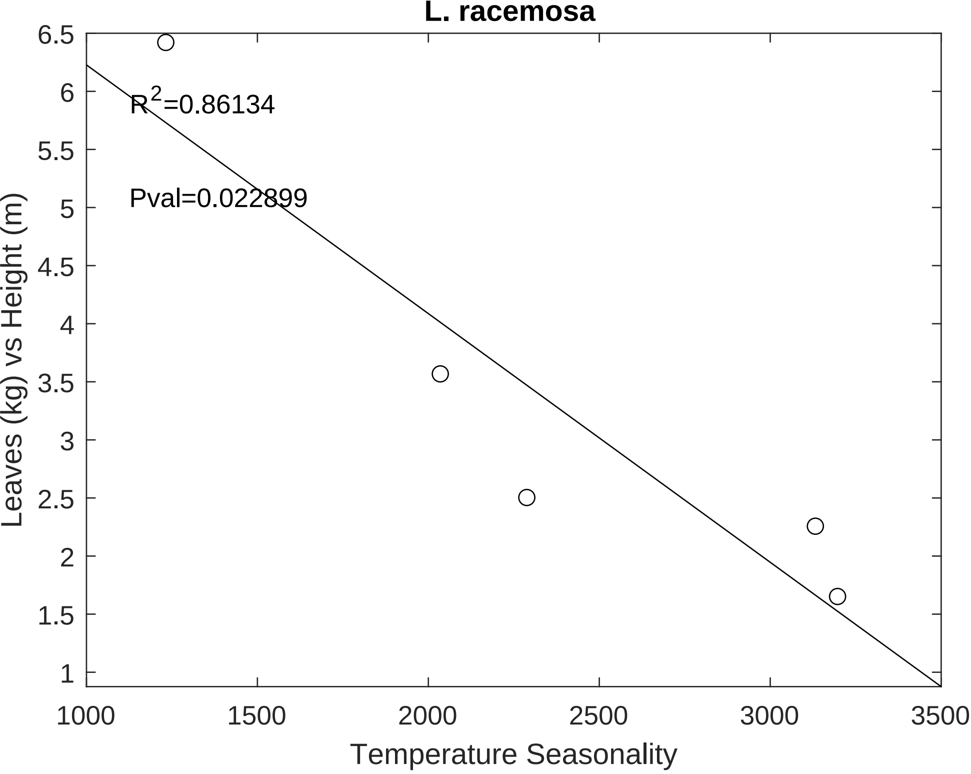
L. racemosa
R2=0.86134
Pval=0.022899
Leaves (kg) vs Height (m)
Temperature Seasonality
6.5
6
5.5
5
4.5
4
3.5
3
2.5
2
1.5
1
1000
1500
2000
2500
3000
3500

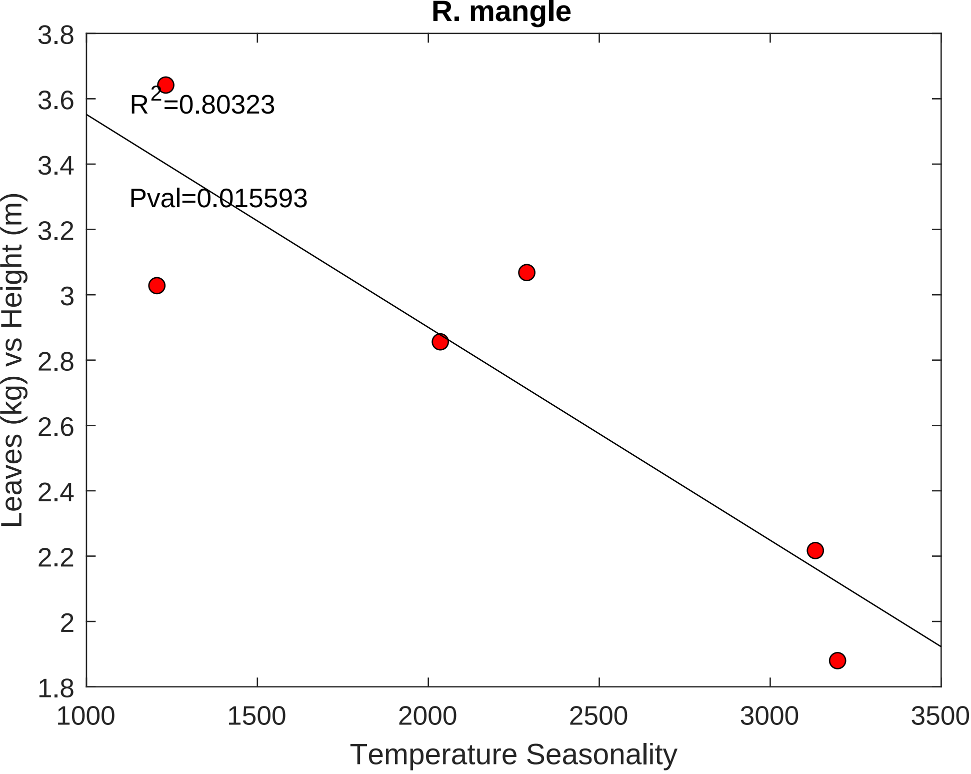

R. mangle
R2=0.80323
Pval=0.015593
Leaves (kg) vs Height (m)
Temperature Seasonality
3.8
3.6
3.4
3.2
3
2.8
2.6
2.4
2.2
2
1.8
1000
1500
2000
2500
3000
3500

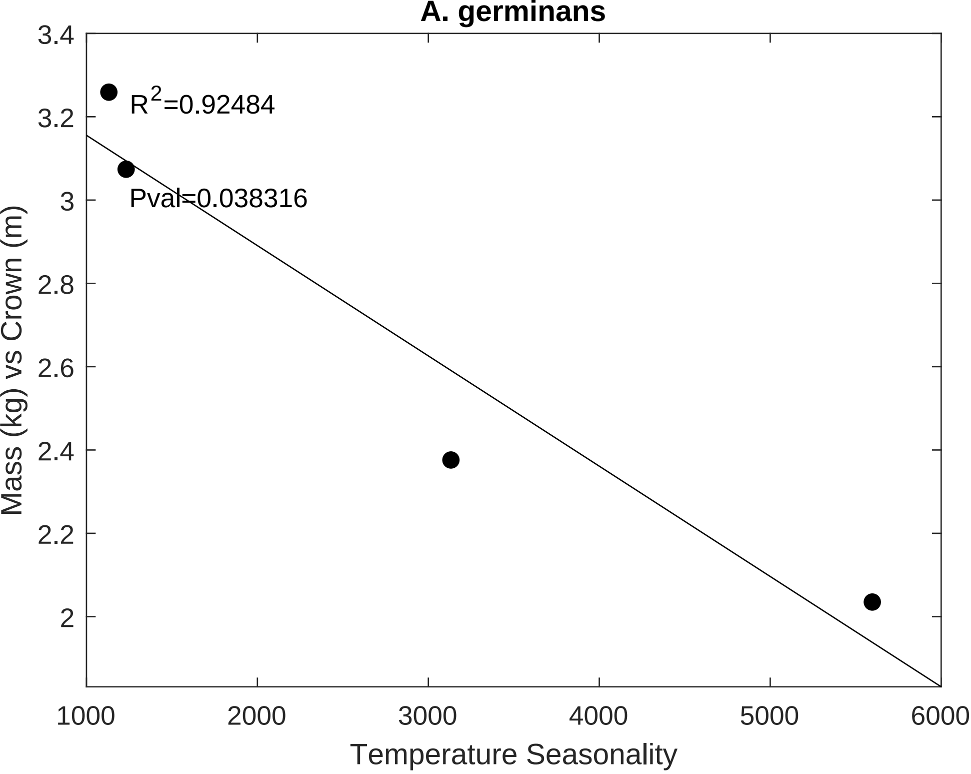
A. germinans
R2=0.92484
Pval=0.038316
Mass (kg) vs Crown (m)
Temperature Seasonality
3.4
3.2
3
2.8
2.6
2.4
2.2
2
1000
2000
3000
4000
5000
6000

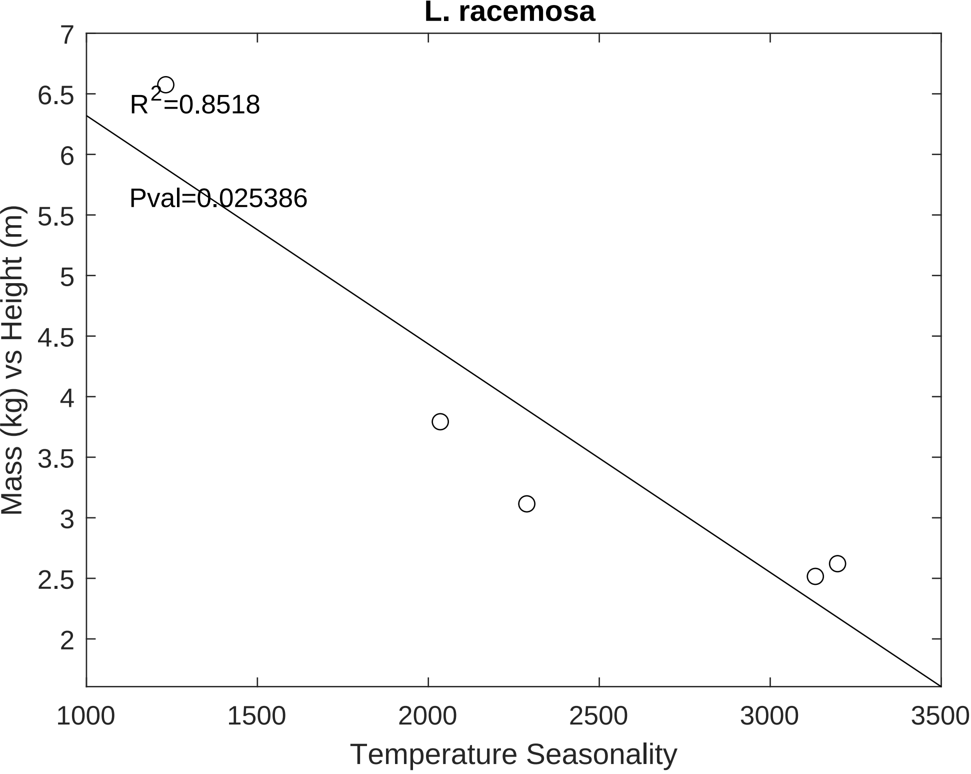

L. racemosa
R2=0.8518
Pval=0.025386
Mass (kg) vs Height (m)
Temperature Seasonality
7
6.5
6
5.5
5
4.5
4
3.5
3
2.5
2
1000
1500
2000
2500
3000
3500

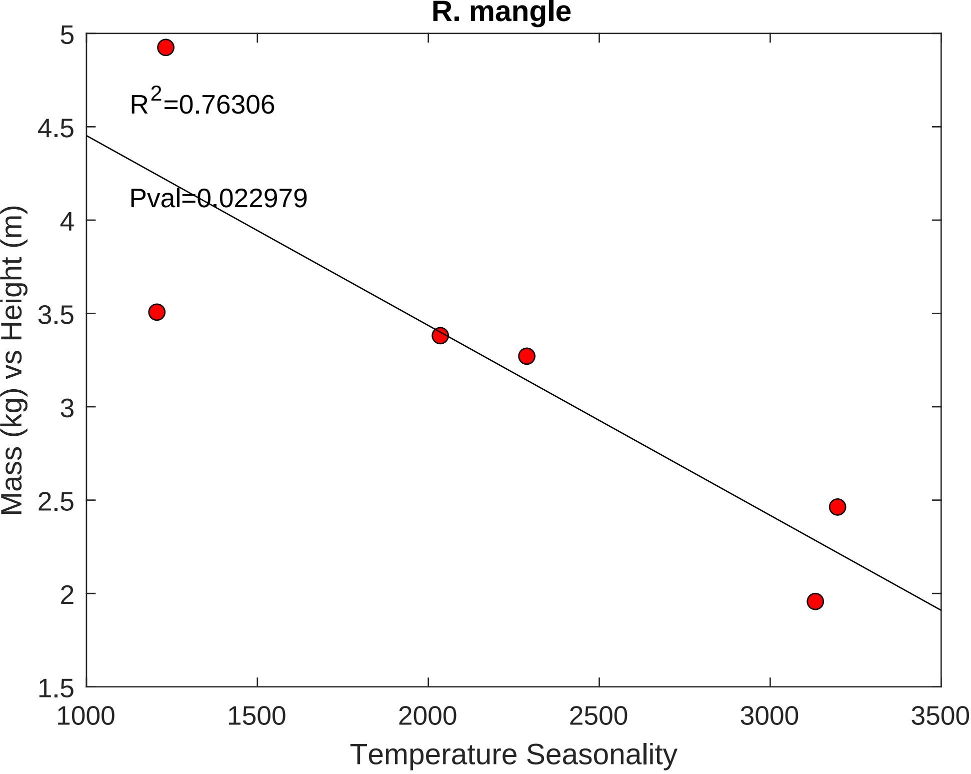
R. mangle
R^2=0.76306
Pval=0.022979
Mass (kg) vs Height (m)
Temperature Seasonality
5
4.5
4
3.5
3
2.5
2
1.5
1000
1500
2000
2500
3000
3500

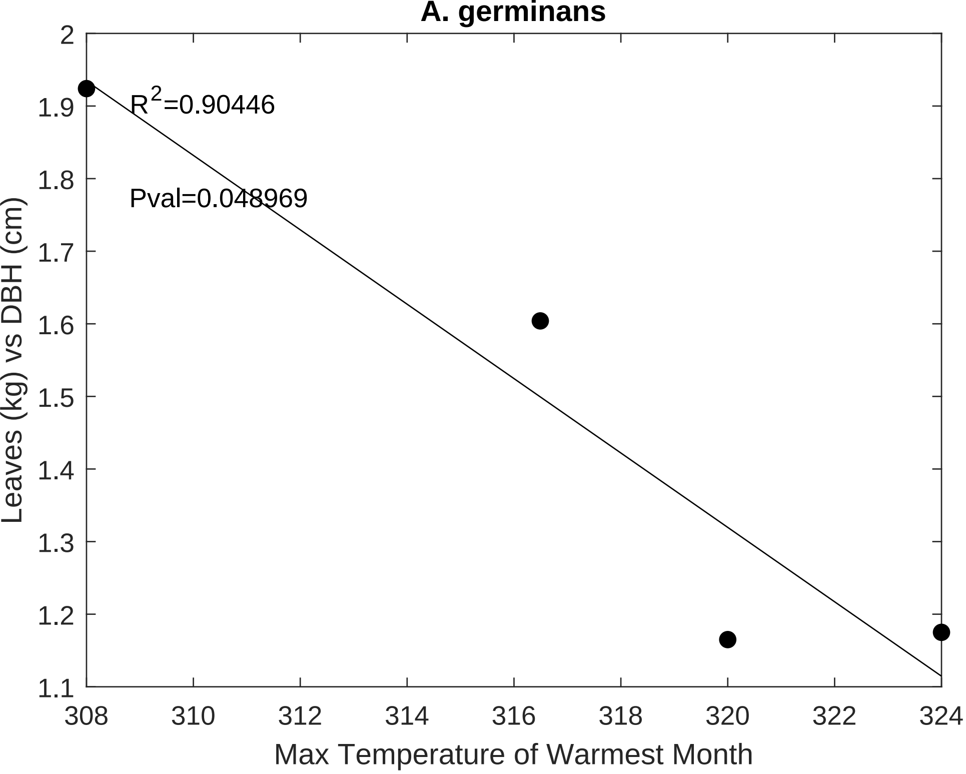

A. germinans
R2=0.90446
Pval=0.048969
Leaves (kg) vs DBH (cm)
Max Temperature of Warmest Month
2
1.9
1.8
1.7
1.6
1.5
1.4
1.3
1.2
1.1
308
310
312
314
316
318
320
322
324

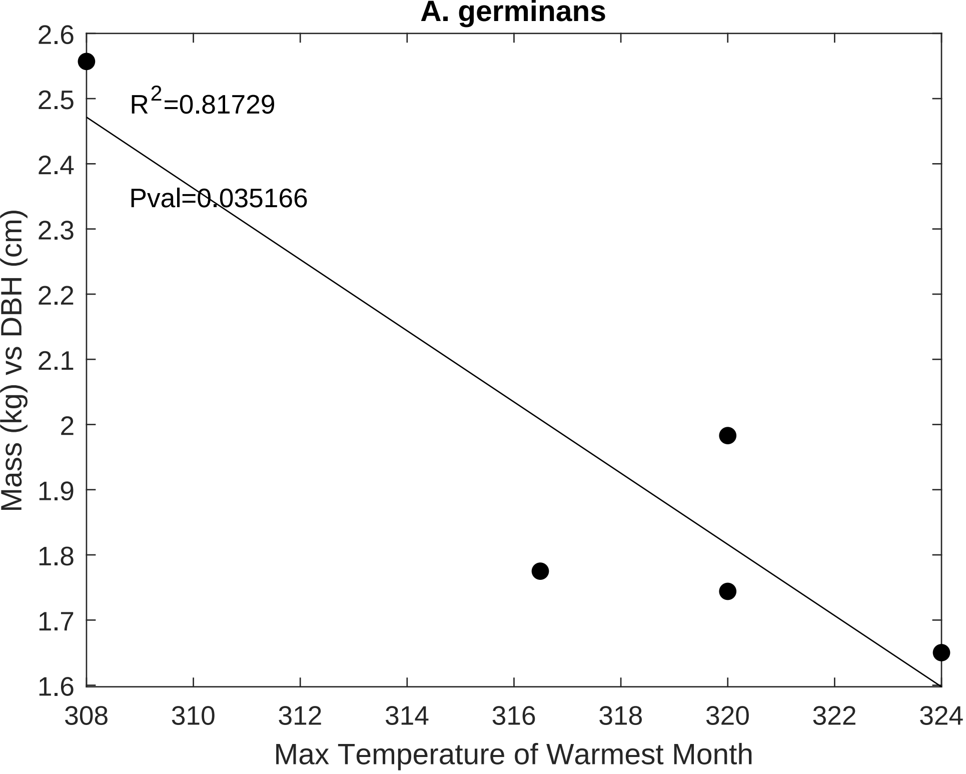
A. germinans
R^2=0.81729
Pval=0.035166
Mass (kg) vs DBH (cm)
Max Temperature of Warmest Month
2.6
2.5
2.4
2.3
2.2
2.1
2
1.9
1.8
1.7
1.6
308
310
312
314
316
318
320
322
324

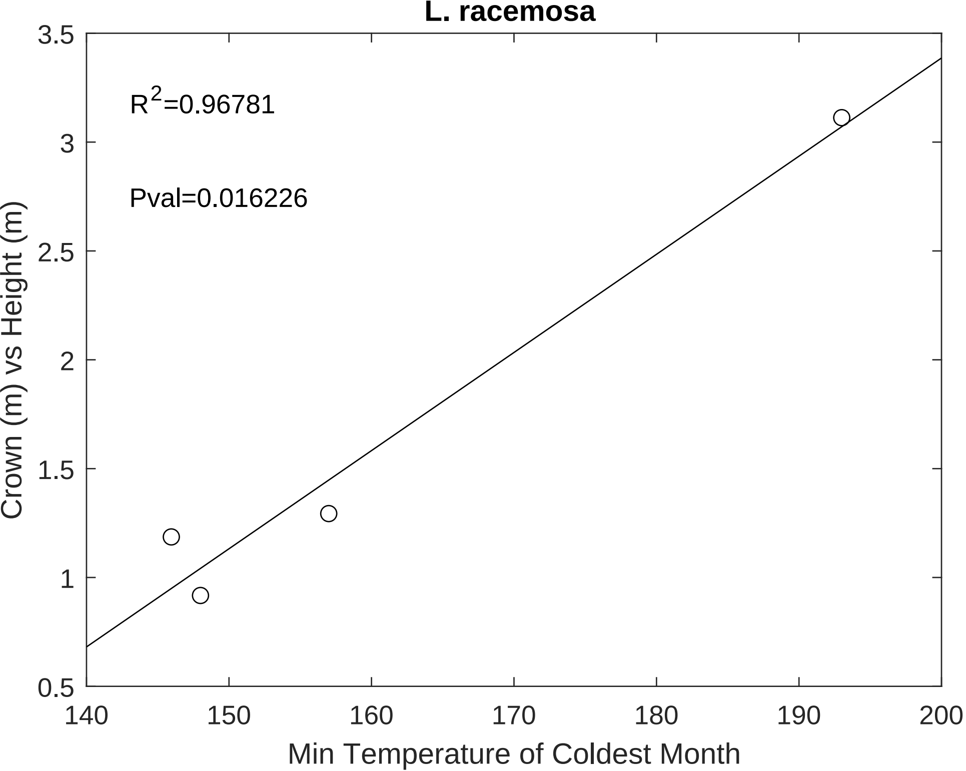
L. racemosa
R2=0.96781
Pval=0.016226
Crown (m) vs Height (m)
Min Temperature of Coldest Month
3.5
3
2.5
2
1.5
1
0.5
140
150
160
170
180
190
200

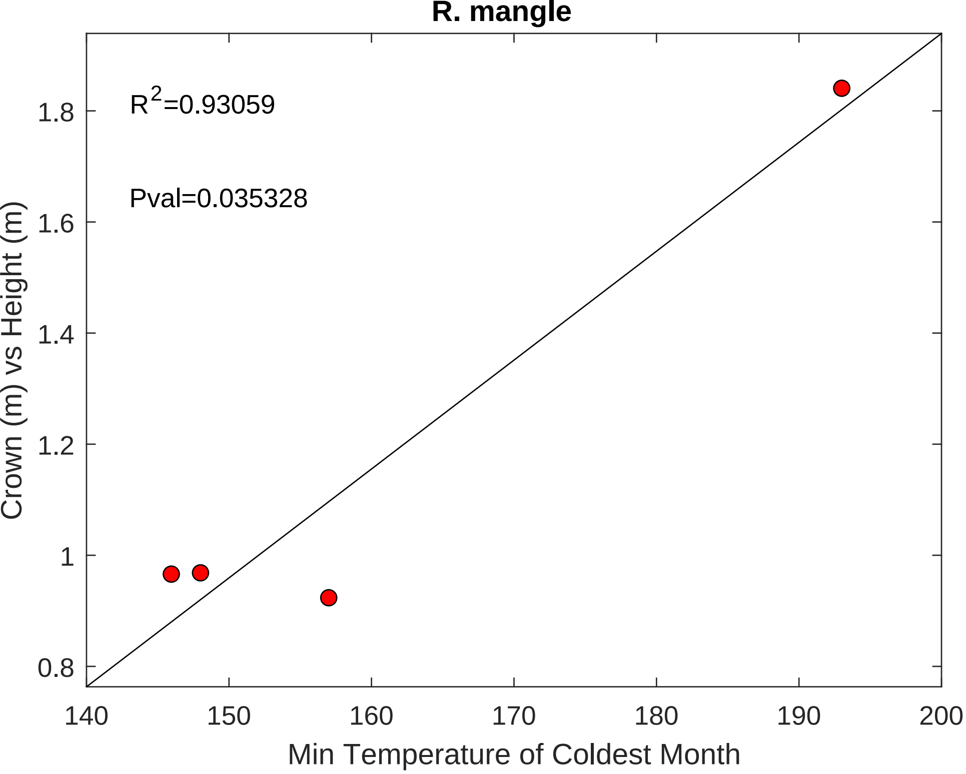

R. mangle
R2=0.93059
Pval=0.035328
Crown (m) vs Height (m)
0.8
1
1.2
1.4
1.6
1.8
140
150
160
170
180
190
200
Min Temperature of Coldest Month

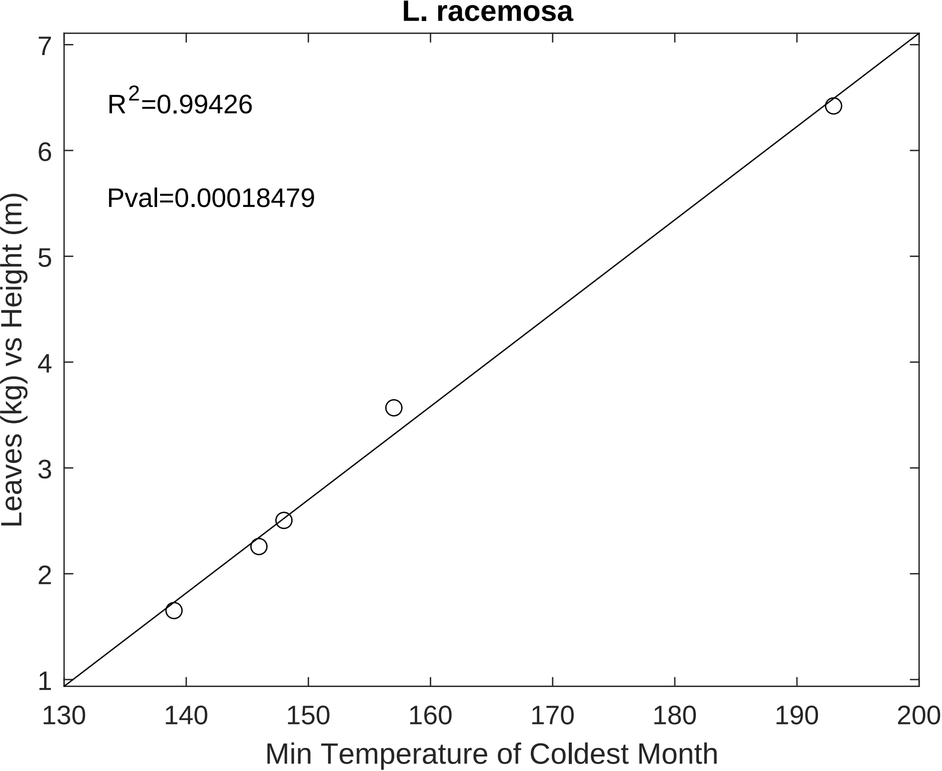
L. racemosa
R2=0.99426
Pval=0.00018479
Leaves (kg) vs Height (m)
1
2
3
4
5
6
7
130
140
150
160
170
180
190
200
Min Temperature of Coldest Month

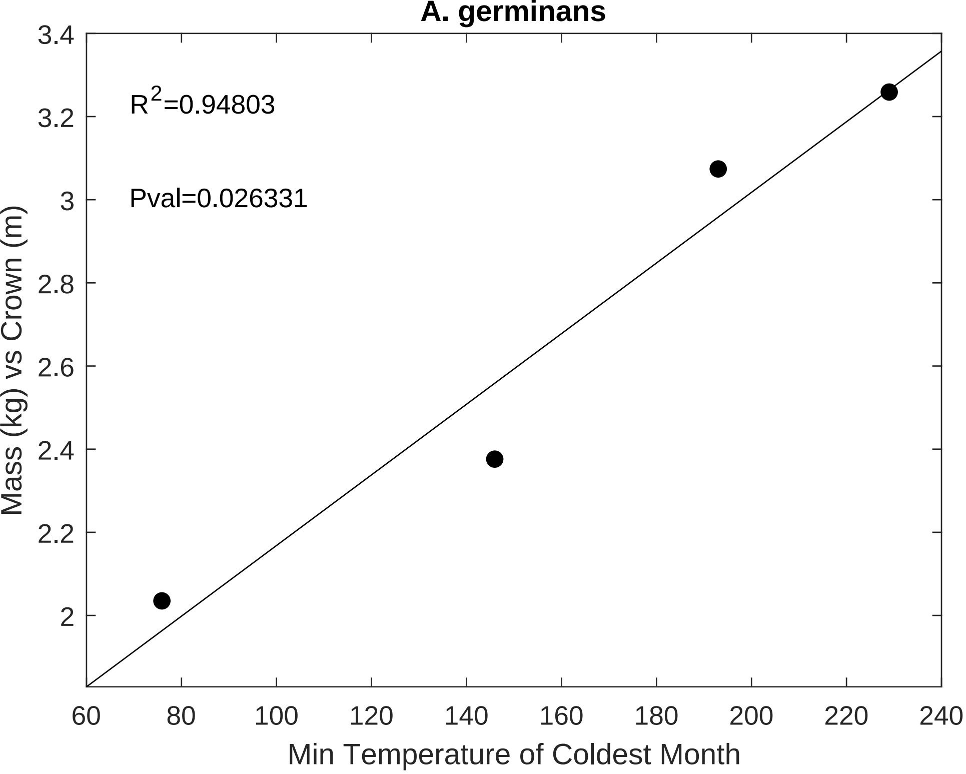
A. germinans
R^2=0.94803
Pval=0.026331
Mass (kg) vs Crown (m)
Min Temperature of Coldest Month
3.4
3.2
3
2.8
2.6
2.4
2.2
2
60
80
100
120
140
160
180
200
220
240

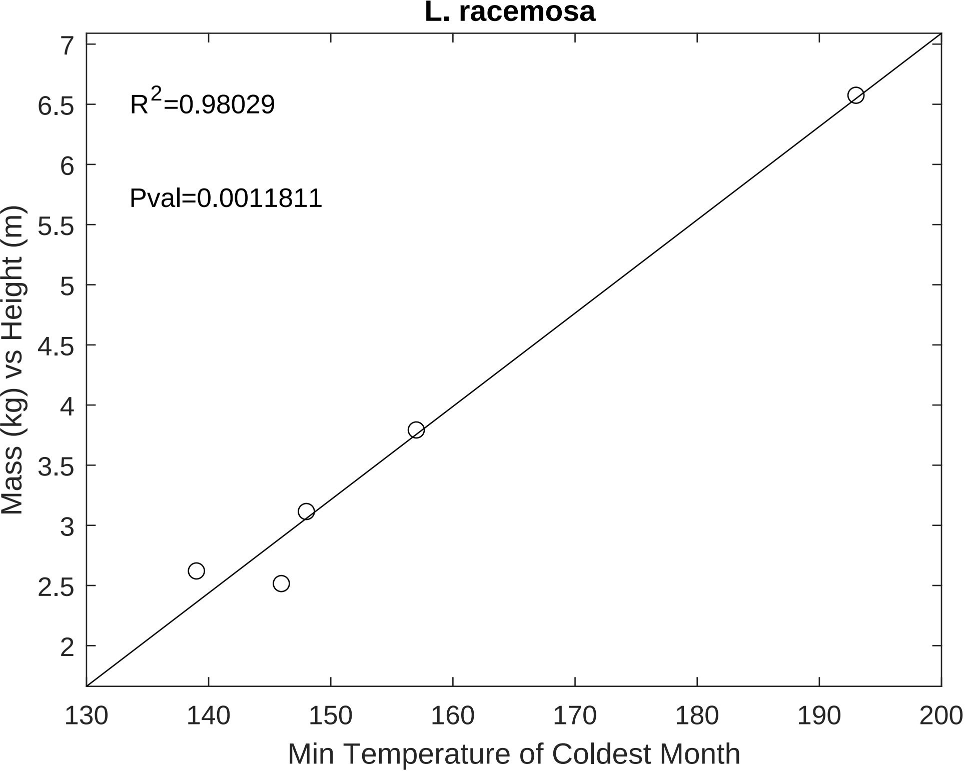

L. racemosa
R2=0.98029
Pval=0.0011811
Mass (kg) vs Height (m)
Min Temperature of Coldest Month
7
6.5
6
5.5
5
4.5
4
3.5
3
2.5
2
130
140
150
160
170
180
190
200

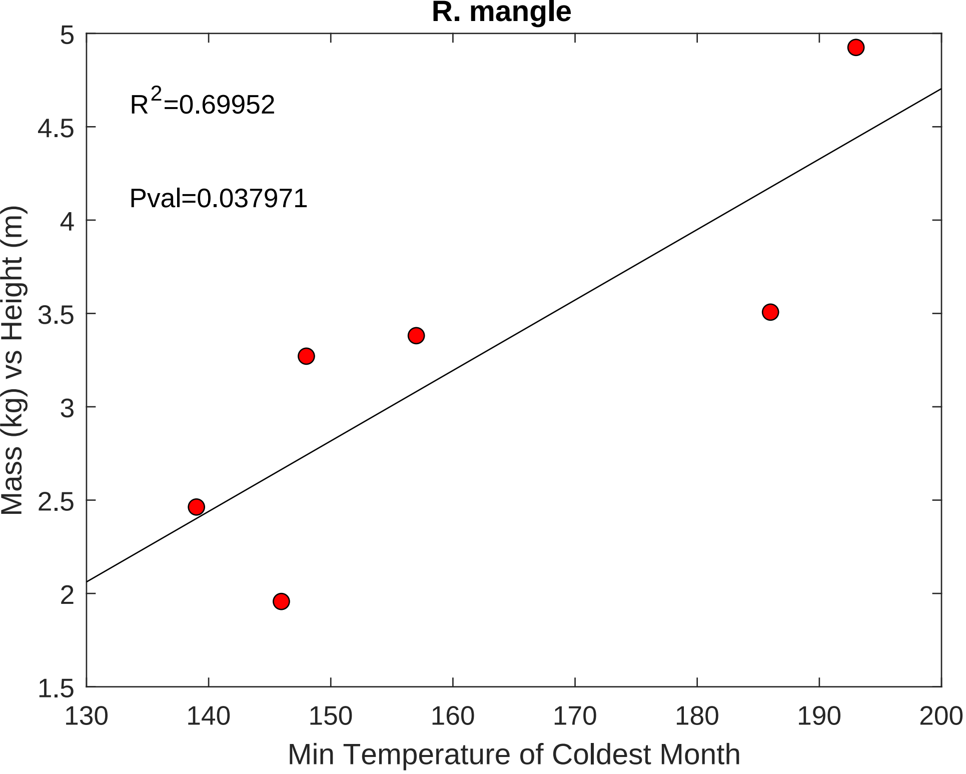
R. mangle
R^2=0.69952
Pval=0.037971
Mass (kg) vs Height (m)
Min Temperature of Coldest Month
5
4.5
4
3.5
3
2.5
2
1.5
130
140
150
160
170
180
190
200

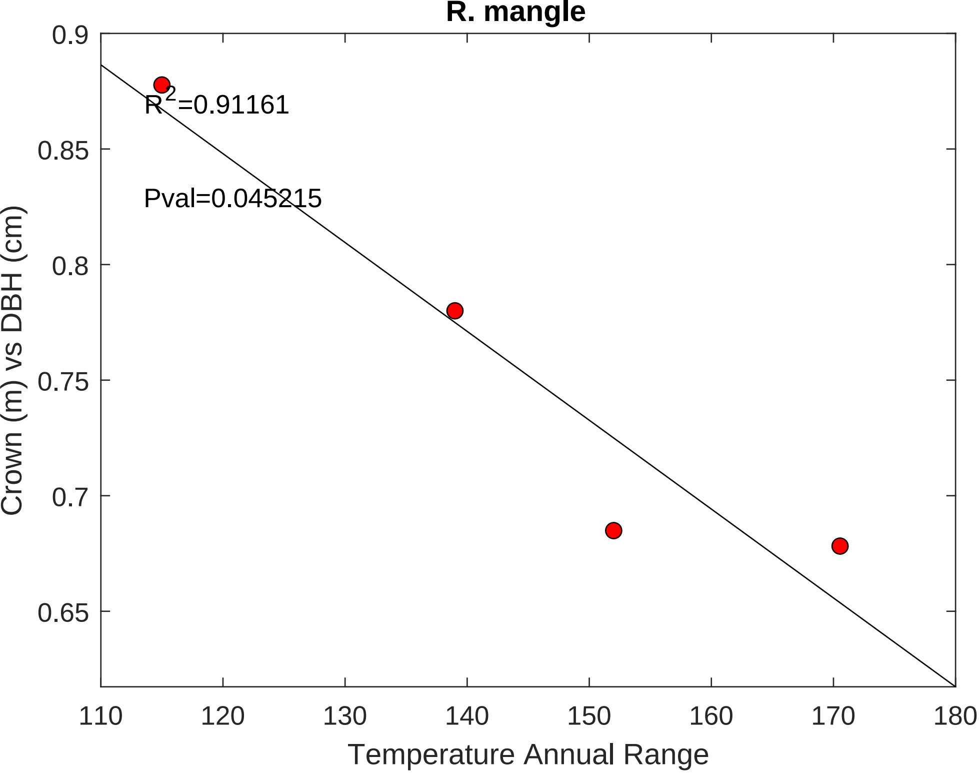
R. mangle
R^2=0.91161
Pval=0.045215
Crown (m) vs DBH (cm)
Temperature Annual Range
0.9
0.85
0.8
0.75
0.7
0.65
110
120
130
140
150
160
170
180

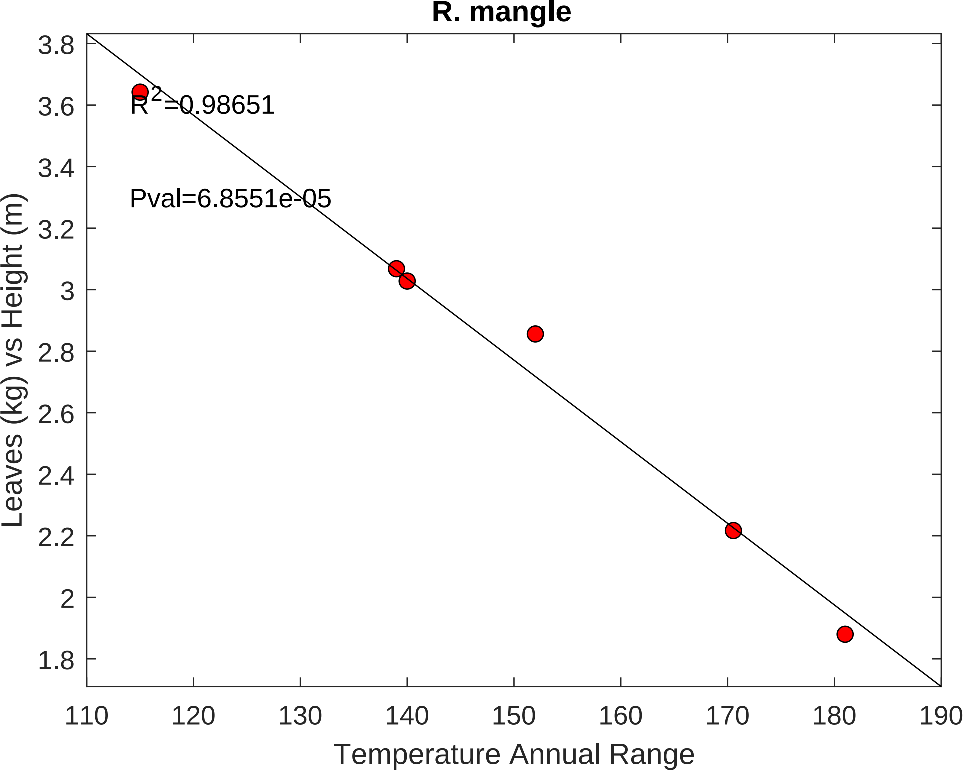
R. mangle
R²=0.98651
Pval=6.8551e-05
Leaves (kg) vs Height (m)
Temperature Annual Range
3.8
3.6
3.4
3.2
3
2.8
2.6
2.4
2.2
2
1.8
110
120
130
140
150
160
170
180
190

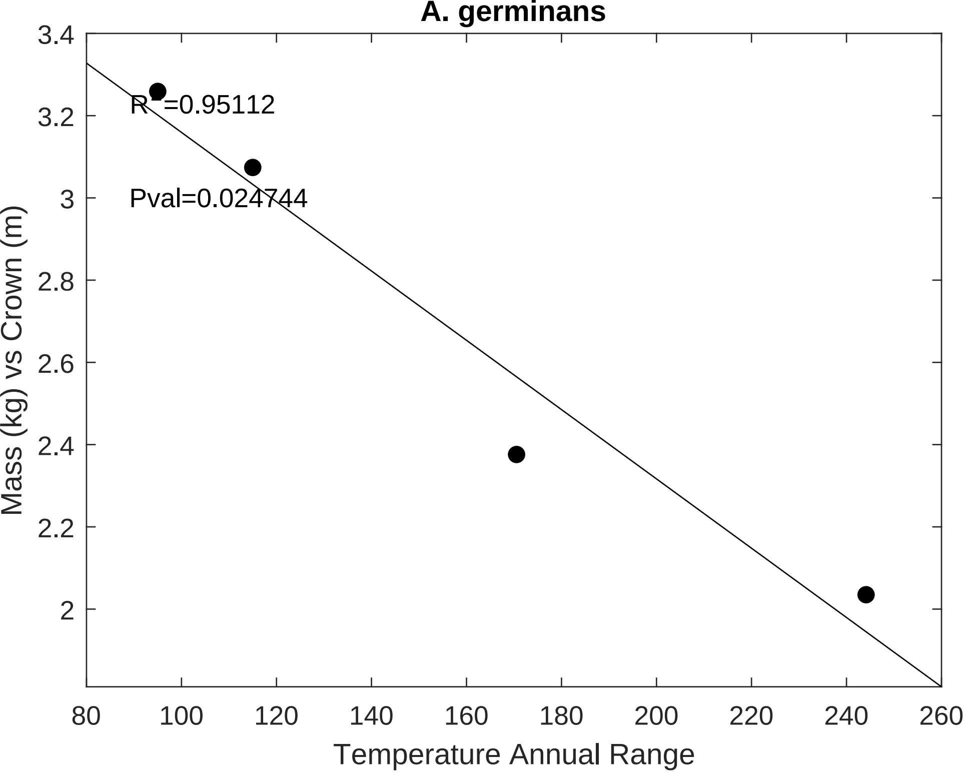
A. germinans
R² =0.95112
Pval=0.024744
Mass (kg) vs Crown (m)
Temperature Annual Range
3.4
3.2
3
2.8
2.6
2.4
2.2
2
80
100
120
140
160
180
200
220
240
260

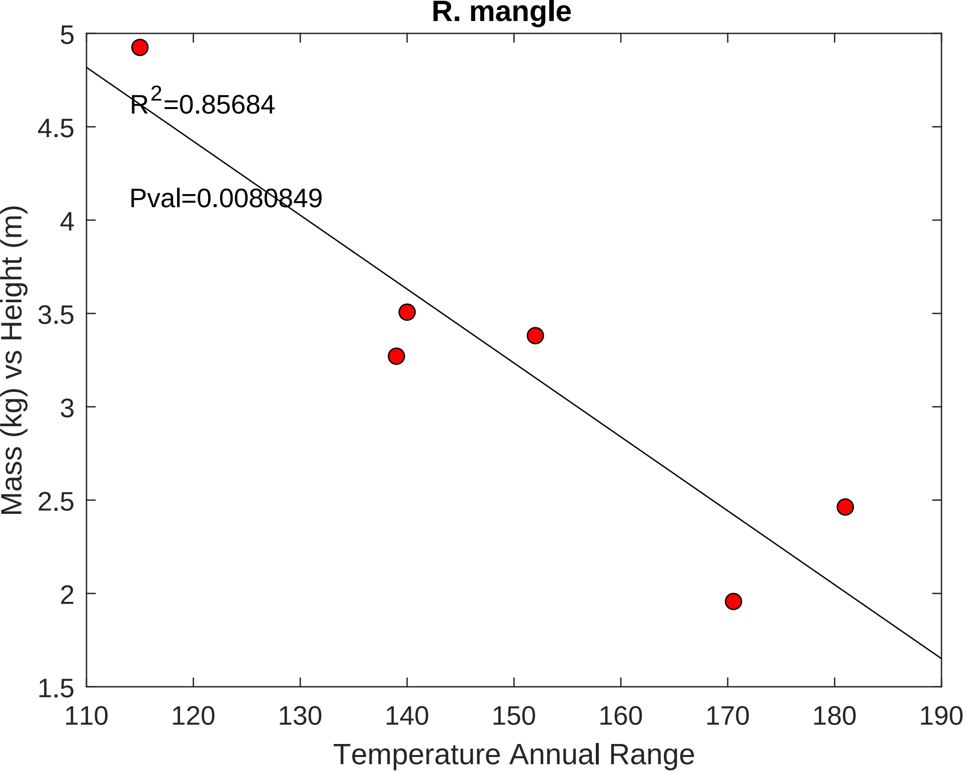

R. mangle
R^2=0.85684
Pval=0.0080849
Mass (kg) vs Height (m)
Temperature Annual Range
5
4.5
4
3.5
3
2.5
2
1.5
110
120
130
140
150
160
170
180
190

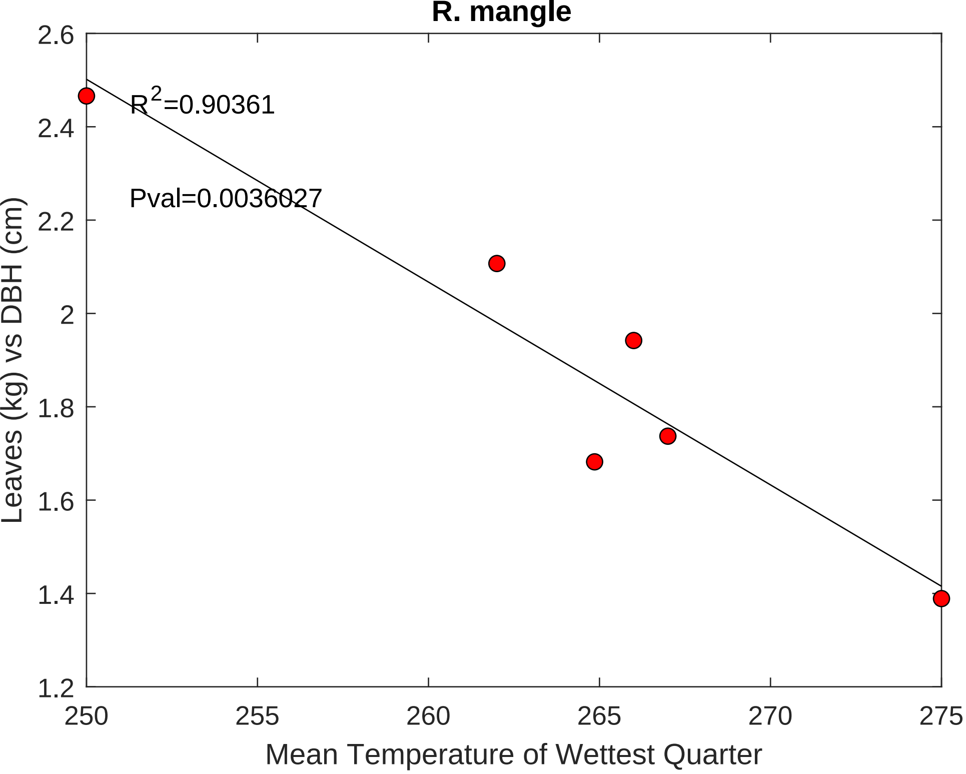
R. mangle
R^2=0.90361
Pval=0.0036027
Leaves (kg) vs DBH (cm)
Mean Temperature of Wettest Quarter
2.6
2.4
2.2
2
1.8
1.6
1.4
1.2
250
255
260
265
270
275

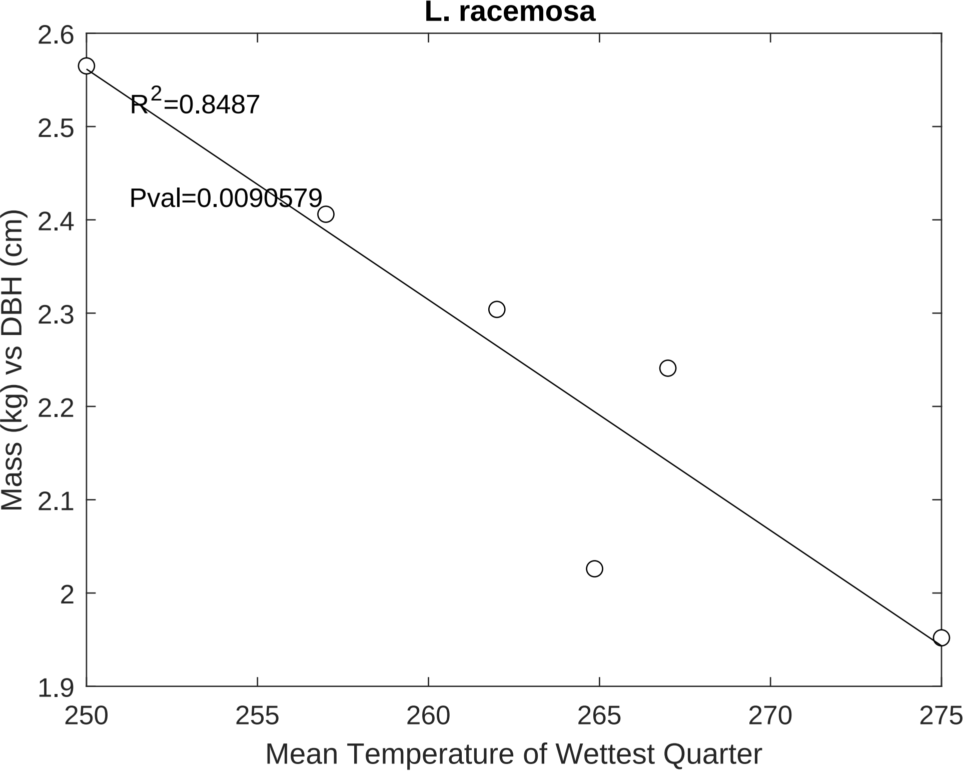

L. racemosa
R2=0.8487
Pval=0.0090579
Mass (kg) vs DBH (cm)
Mean Temperature of Wettest Quarter
2.6
2.5
2.4
2.3
2.2
2.1
2
1.9
250
255
260
265
270
275

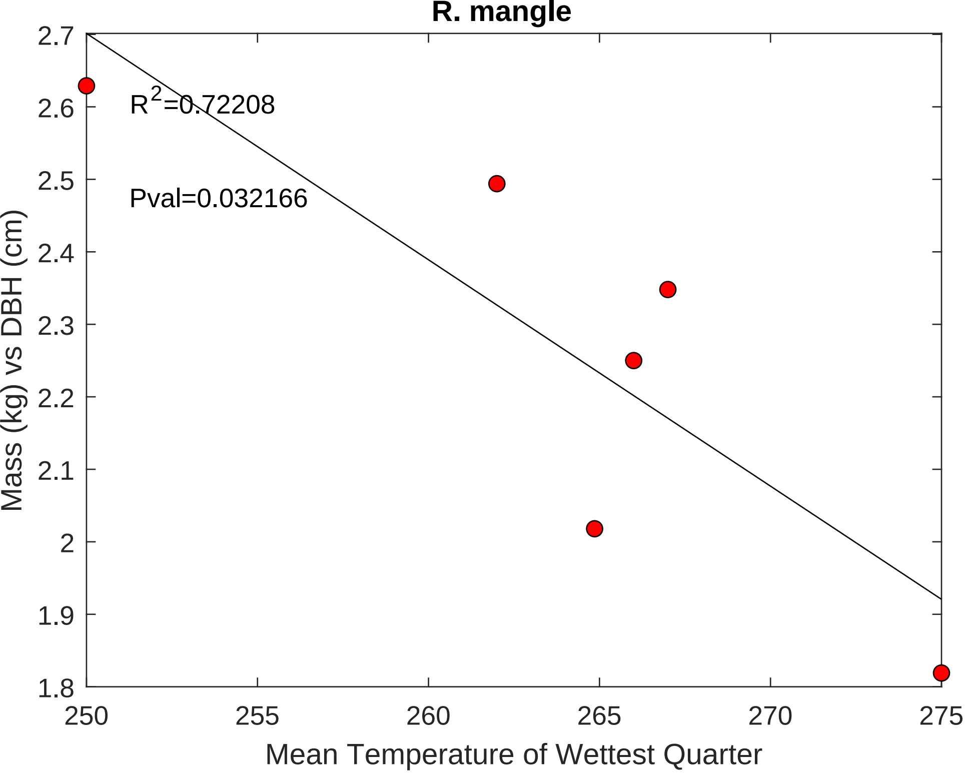
R. mangle
R2=0.72208
Pval=0.032166
Mass (kg) vs DBH (cm)
Mean Temperature of Wettest Quarter
2.7
2.6
2.5
2.4
2.3
2.2
2.1
2
1.9
1.8
250
255
260
265
270
275

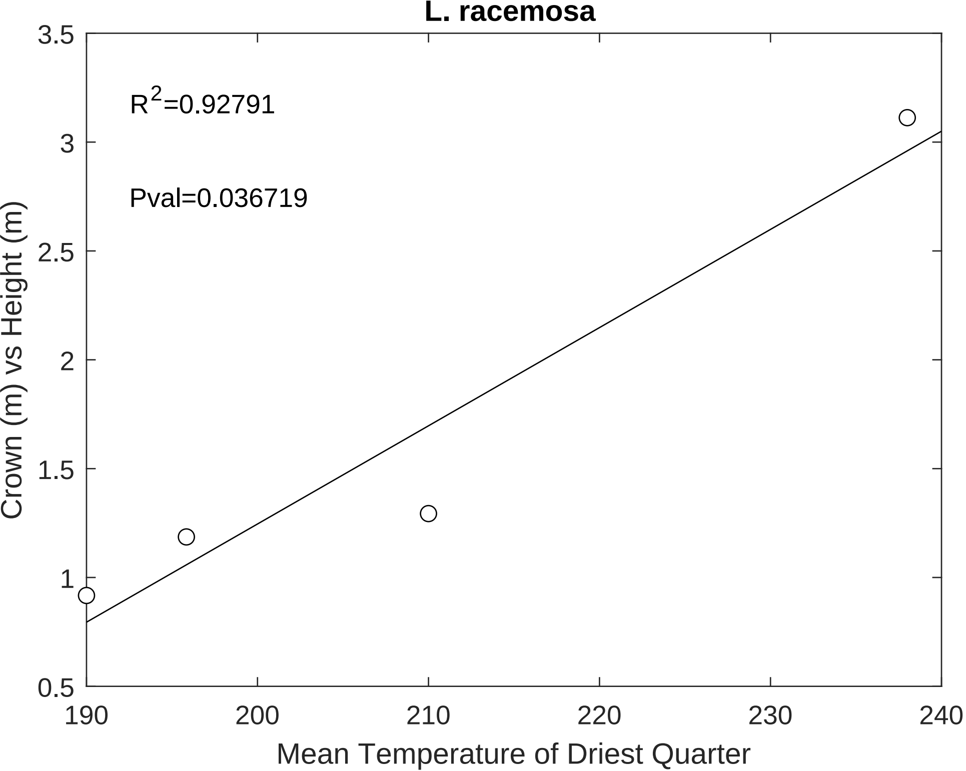
L. racemosa
R2=0.92791
Pval=0.036719
3.5
3
2.5
2
1.5
1
0.5
Crown (m) vs Height (m)
190
200
210
220
230
240
Mean Temperature of Driest Quarter

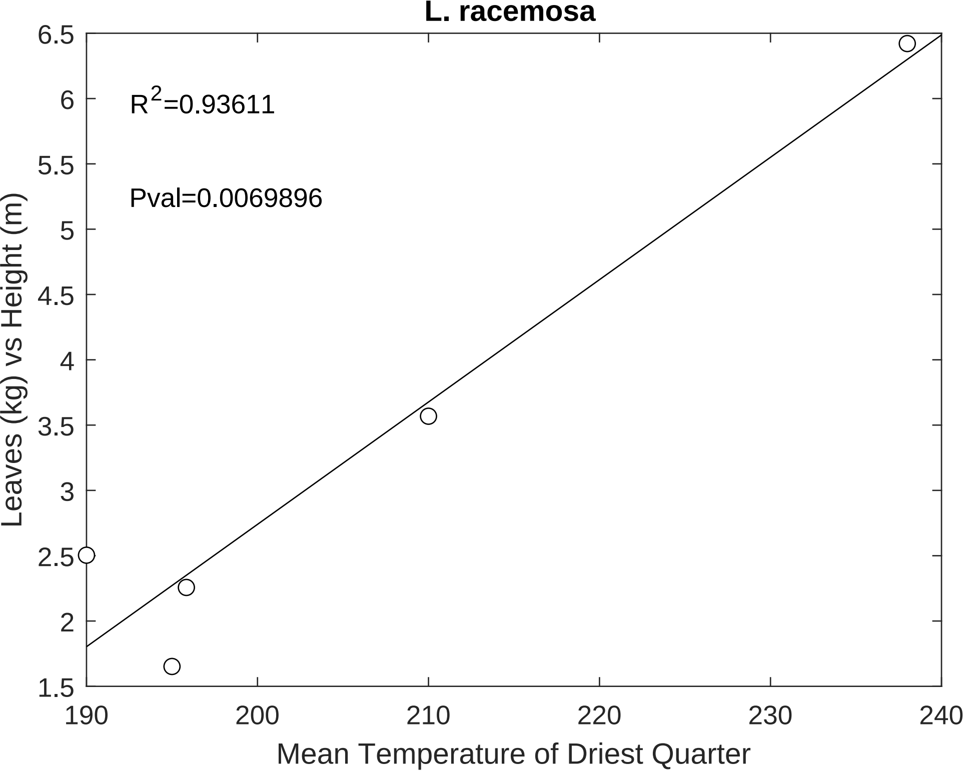
L. racemosa
R2=0.93611
Pval=0.0069896
Leaves (kg) vs Height (m)
Mean Temperature of Driest Quarter
6.5
6
5.5
5
4.5
4
3.5
3
2.5
2
1.5
190
200
210
220
230
240

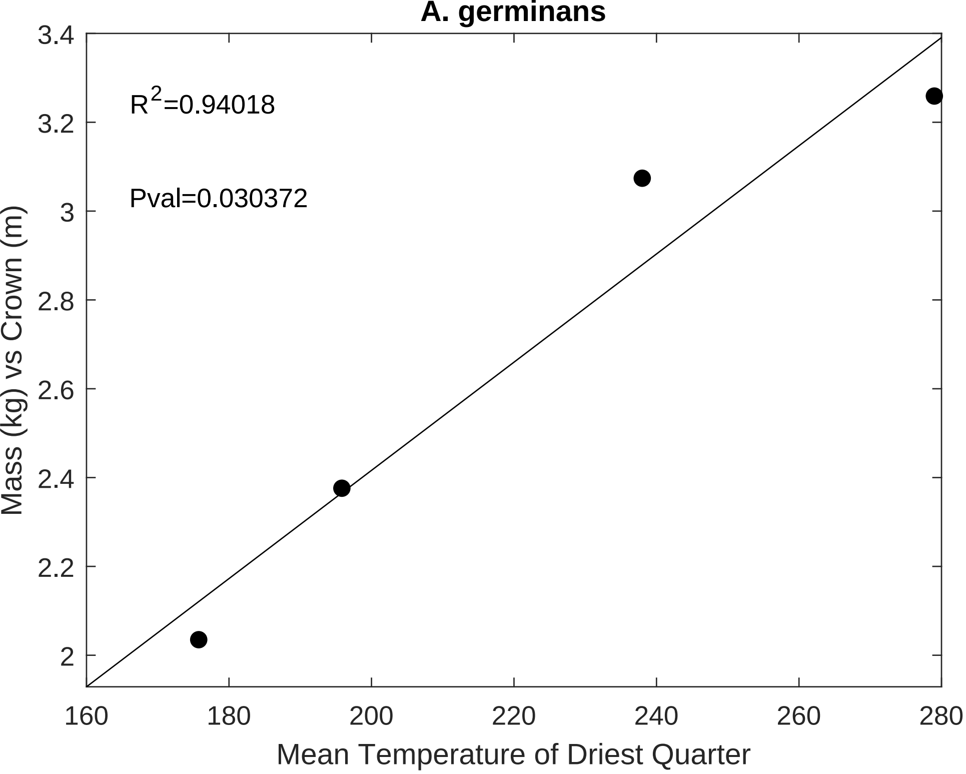
A. germinans
R2=0.94018
Pval=0.030372
Mass (kg) vs Crown (m)
Mean Temperature of Driest Quarter
3.4
3.2
3
2.8
2.6
2.4
2.2
2
160
180
200
220
240
260
280

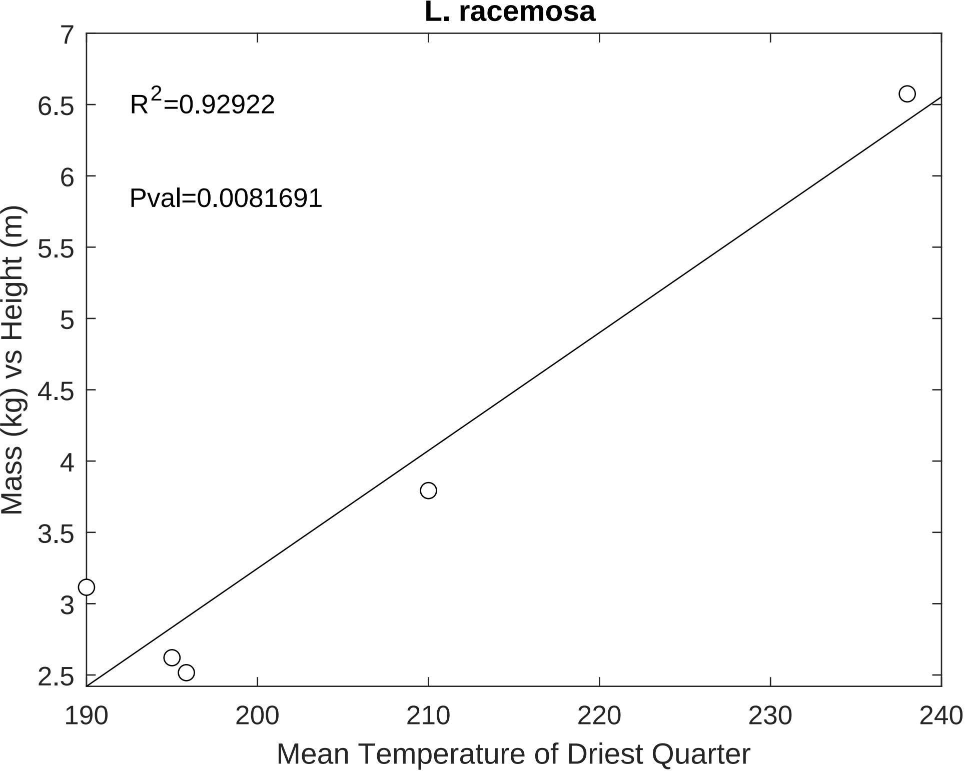
L. racemosa
R2=0.92922
Pval=0.0081691
Mass (kg) vs Height (m)
Mean Temperature of Driest Quarter
7
6.5
6
5.5
5
4.5
4
3.5
3
2.5
190
200
210
220
230
240

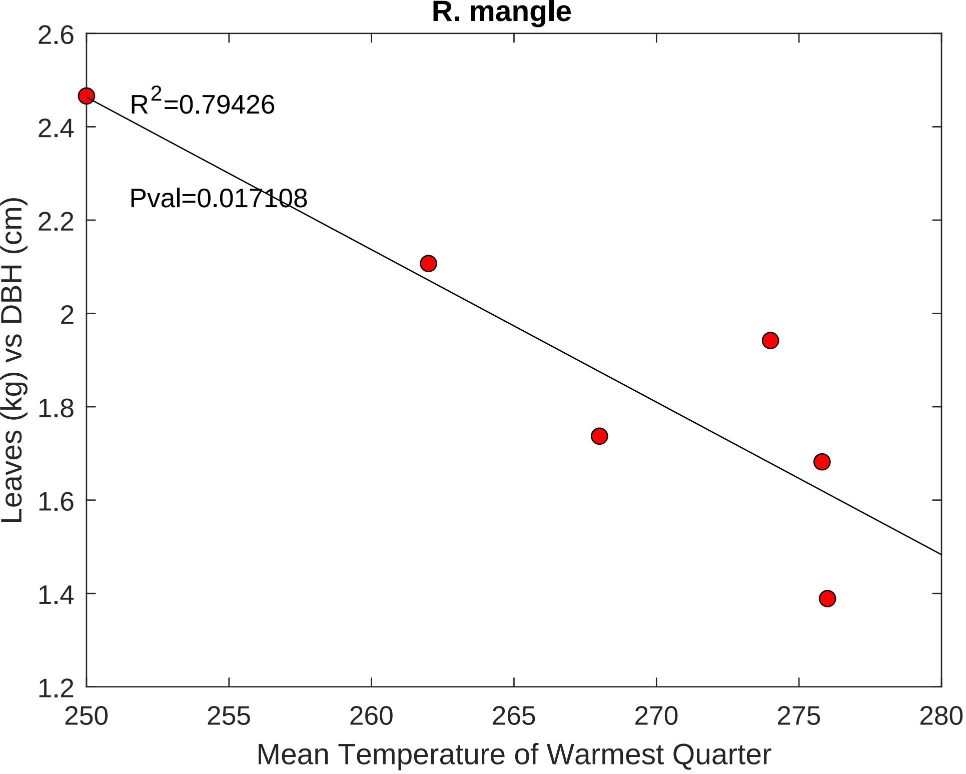

R. mangle
R^2=0.79426
Pval=0.017108
Leaves (kg) vs DBH (cm)
Mean Temperature of Warmest Quarter
2.6
2.4
2.2
2
1.8
1.6
1.4
1.2
250
255
260
265
270
275
280

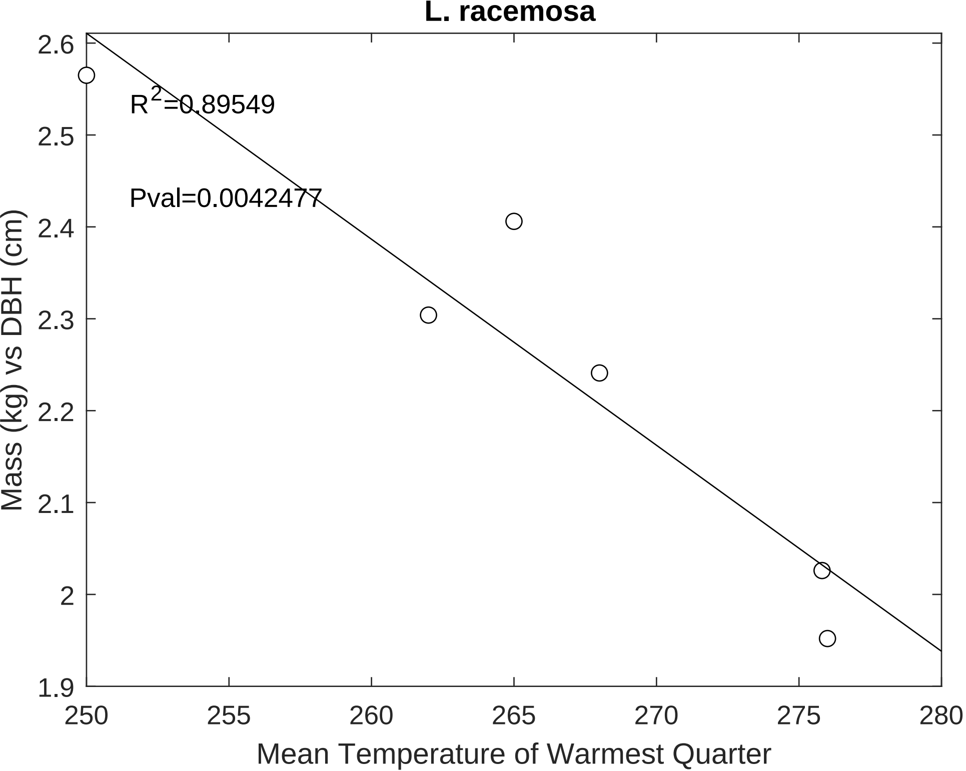

L. racemosa
R2=0.89549
Pval=0.0042477
Mass (kg) vs DBH (cm)
2.6
2.5
2.4
2.3
2.2
2.1
2
1.9
250
255
260
265
270
275
280
Mean Temperature of Warmest Quarter

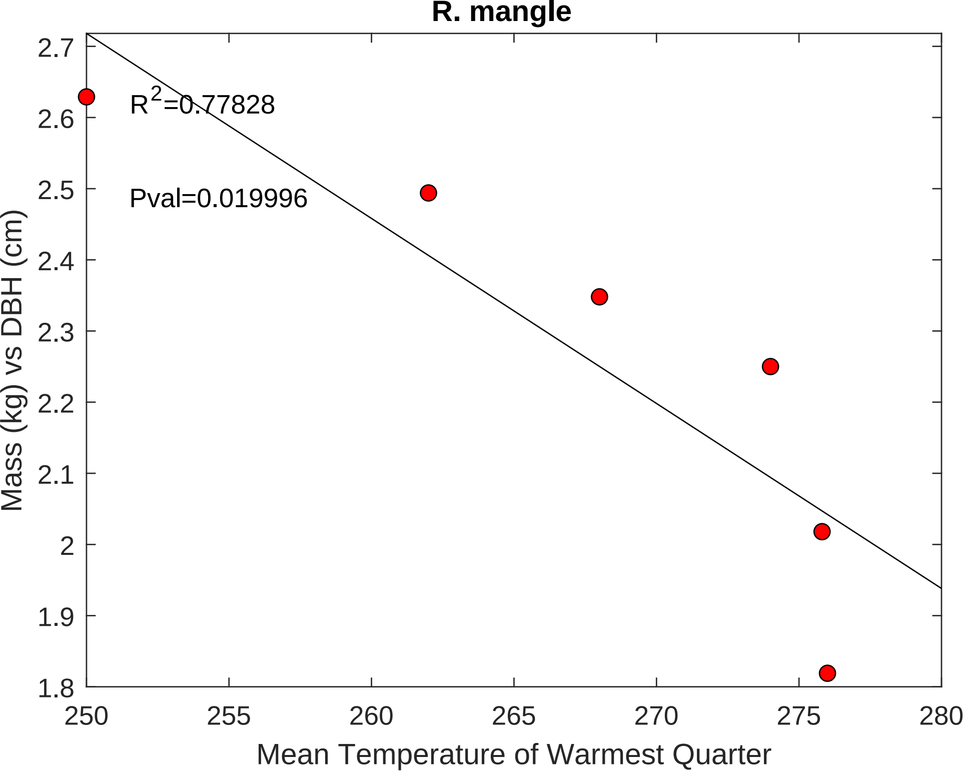
R. mangle
R^2=0.77828
Pval=0.019996
Mass (kg) vs DBH (cm)
Mean Temperature of Warmest Quarter
2.7
2.6
2.5
2.4
2.3
2.2
2.1
2
1.9
1.8
250
255
260
265
270
275
280

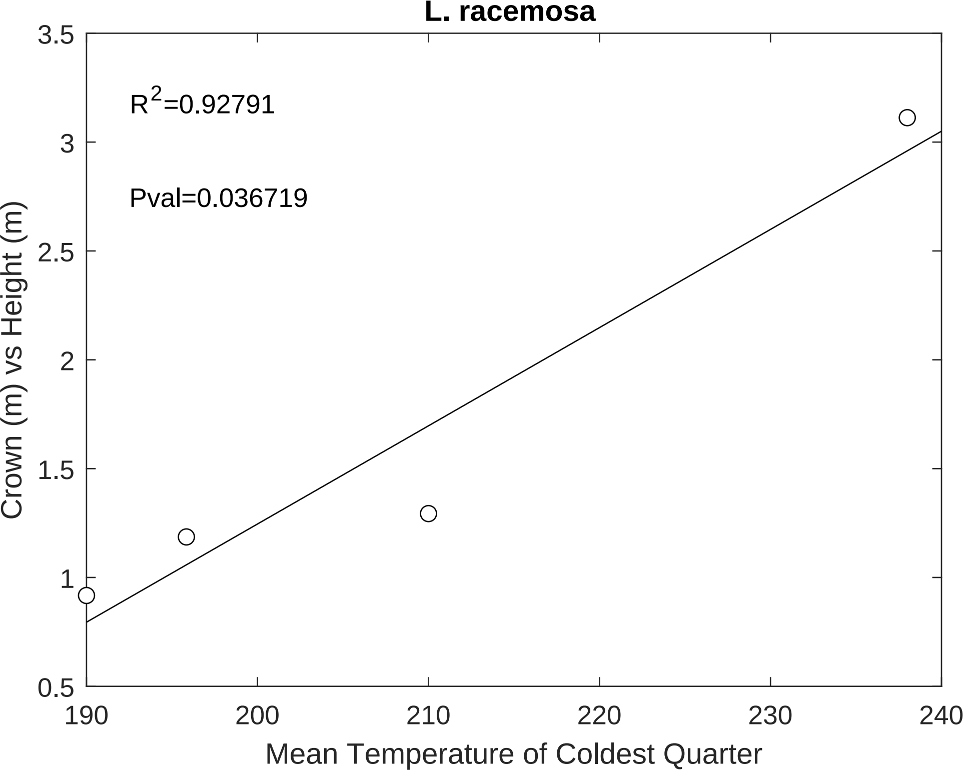
L. racemosa
R^2=0.92791
Pval=0.036719
Crown (m) vs Height (m)
Mean Temperature of Coldest Quarter
3.5
3
2.5
2
1.5
1
0.5
190
200
210
220
230
240

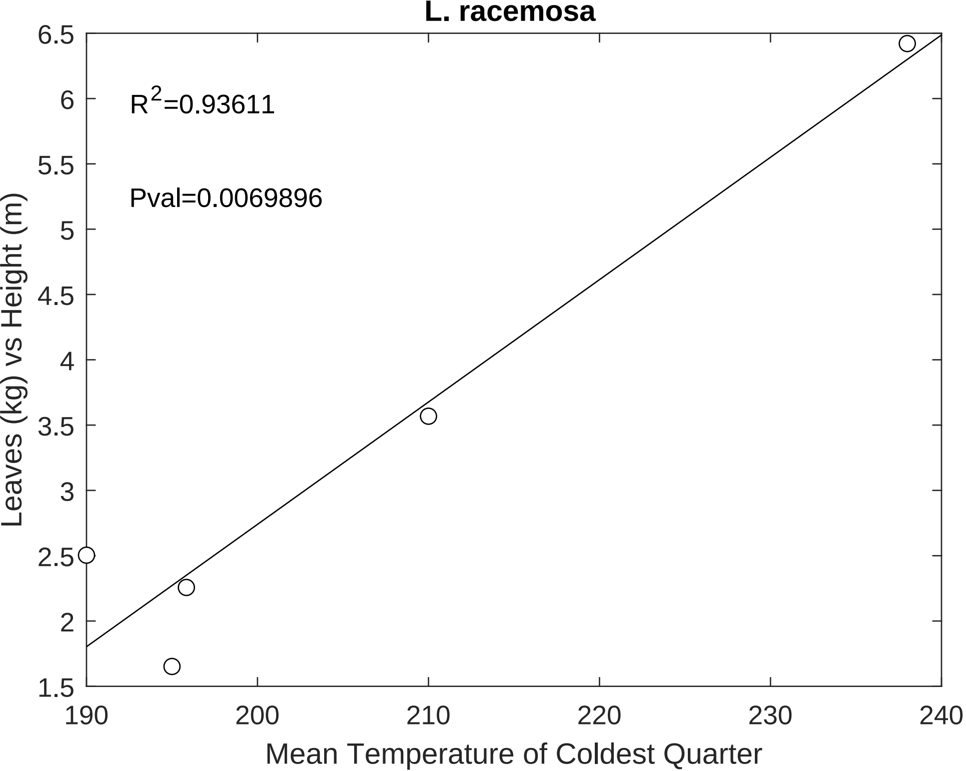
L. racemosa
R²=0.93611
Pval=0.0069896
Leaves (kg) vs Height (m)
Mean Temperature of Coldest Quarter
6.5
6
5.5
5
4.5
4
3.5
3
2.5
2
1.5
190
200
210
220
230
240

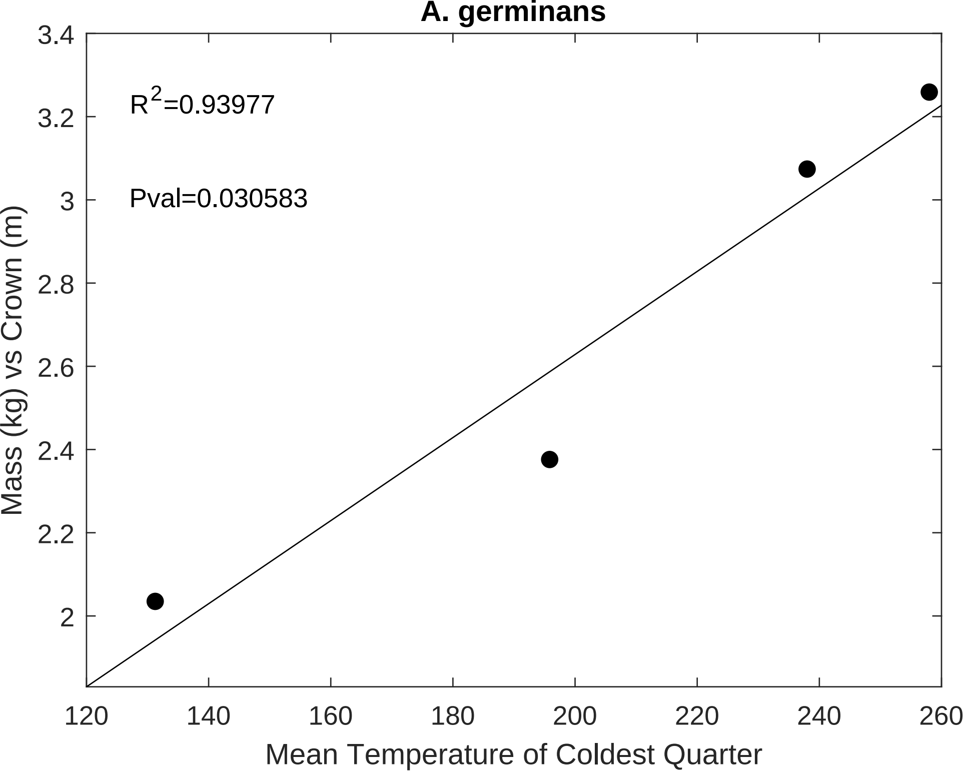
A. germinans
R^2=0.93977
Pval=0.030583
Mass (kg) vs Crown (m)
Mean Temperature of Coldest Quarter
3.4
3.2
3
2.8
2.6
2.4
2.2
2
120
140
160
180
200
220
240
260

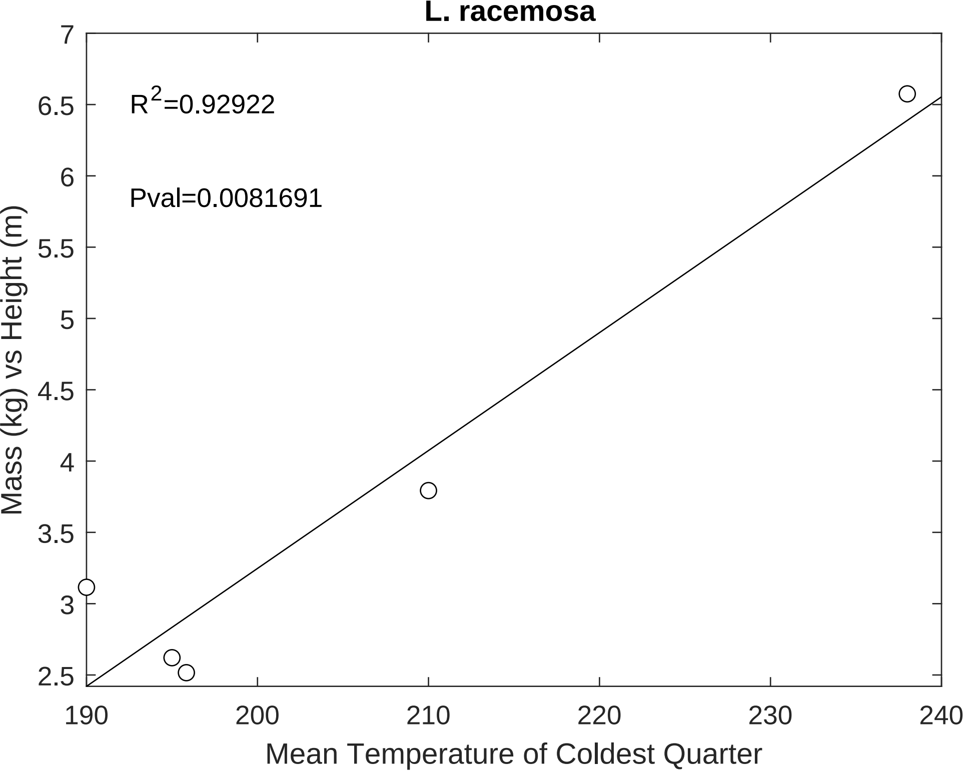
L. racemosa
R^2=0.92922
Pval=0.0081691
Mass (kg) vs Height (m)
Mean Temperature of Coldest Quarter
7
6.5
6
5.5
5
4.5
4
3.5
3
2.5
190
200
210
220
230
240

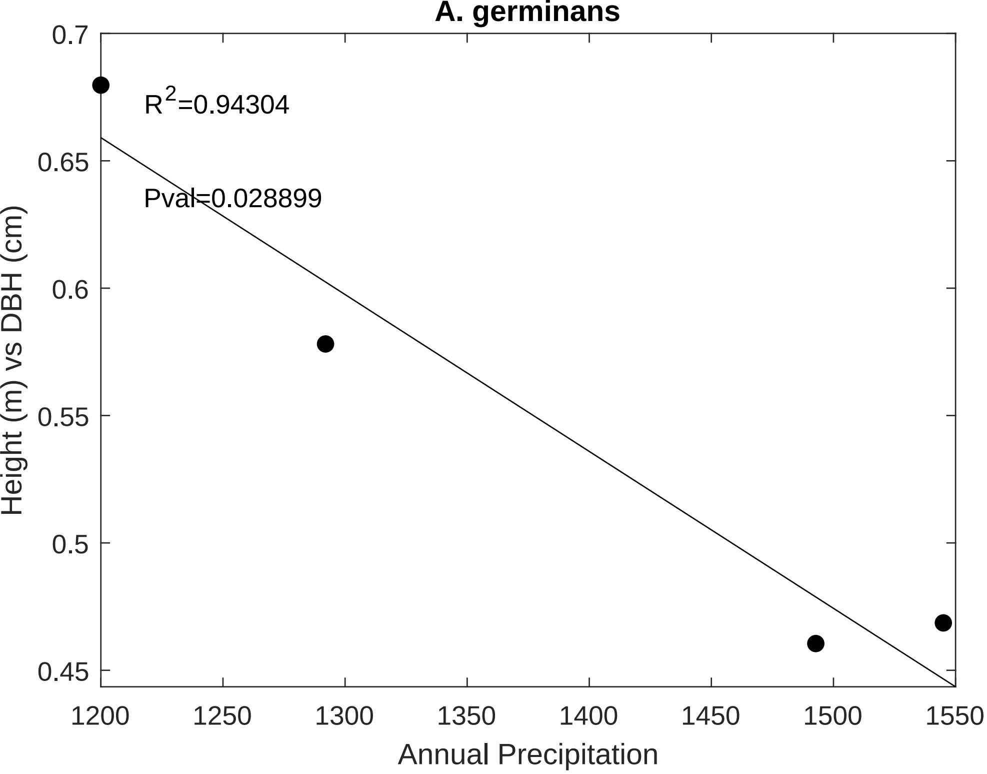

A. germinans
R^2=0.94304
Pval=0.028899
Height (m) vs DBH (cm)
Annual Precipitation
0.7
0.65
0.6
0.55
0.5
0.45
1200
1250
1300
1350
1400
1450
1500
1550

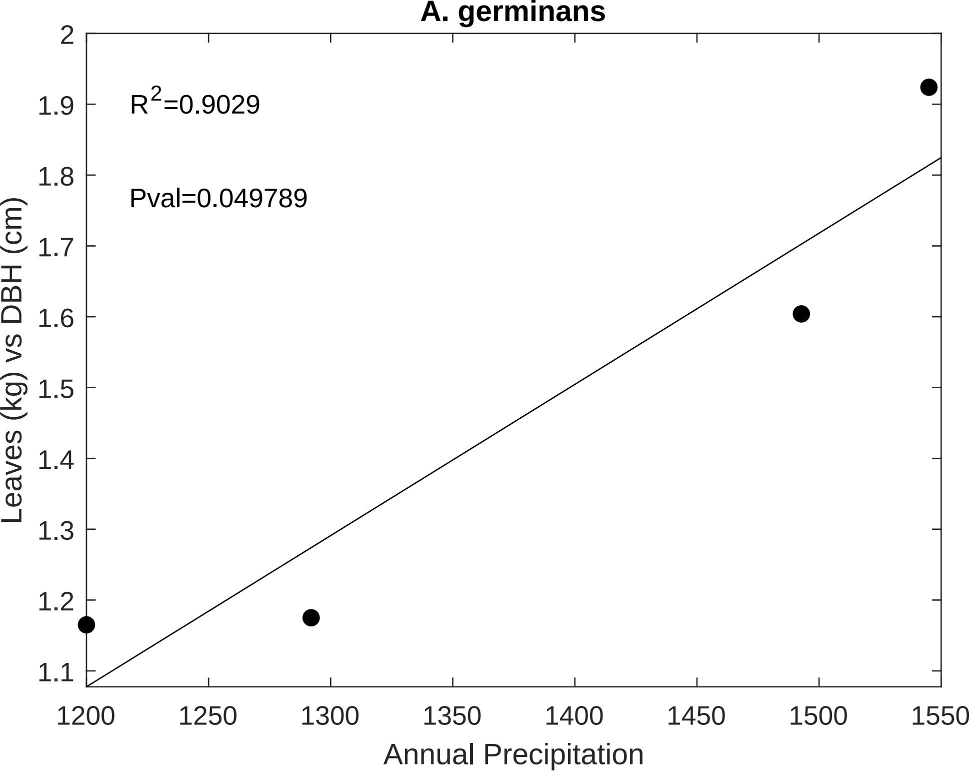

A. germinans
R2=0.9029
Pval=0.049789
Leaves (kg) vs DBH (cm)
Annual Precipitation
2
1.9
1.8
1.7
1.6
1.5
1.4
1.3
1.2
1.1
1200
1250
1300
1350
1400
1450
1500
1550

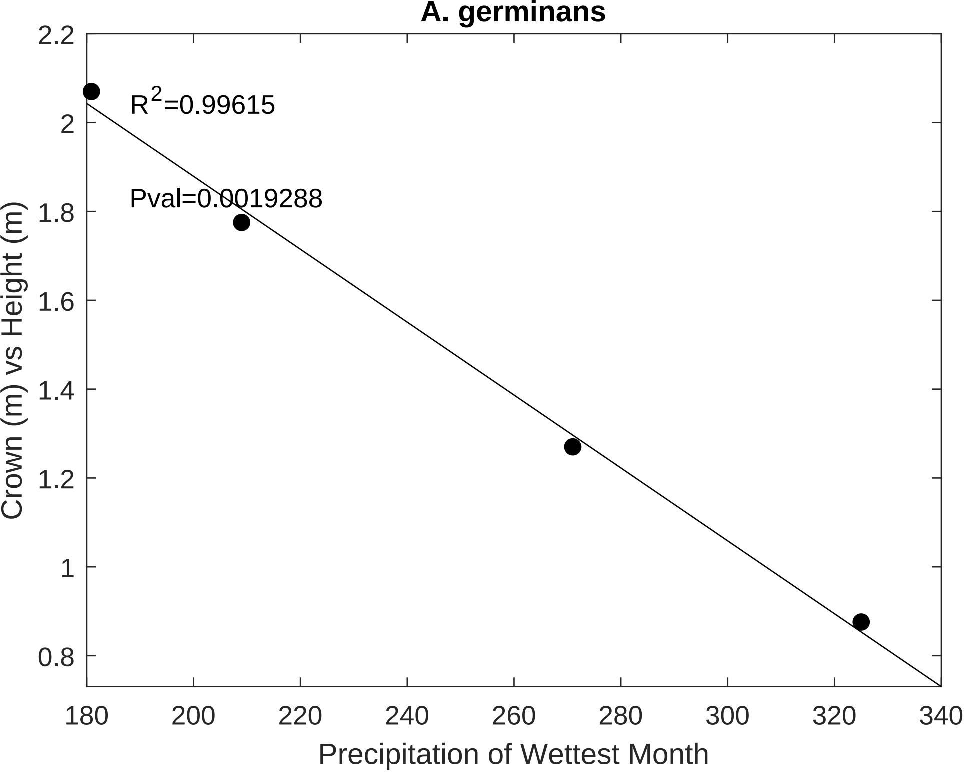

A. germinans
R2=0.99615
Pval=0.0019288
Crown (m) vs Height (m)
Precipitation of Wettest Month
2.2
2
1.8
1.6
1.4
1.2
1
0.8
180
200
220
240
260
280
300
320
340

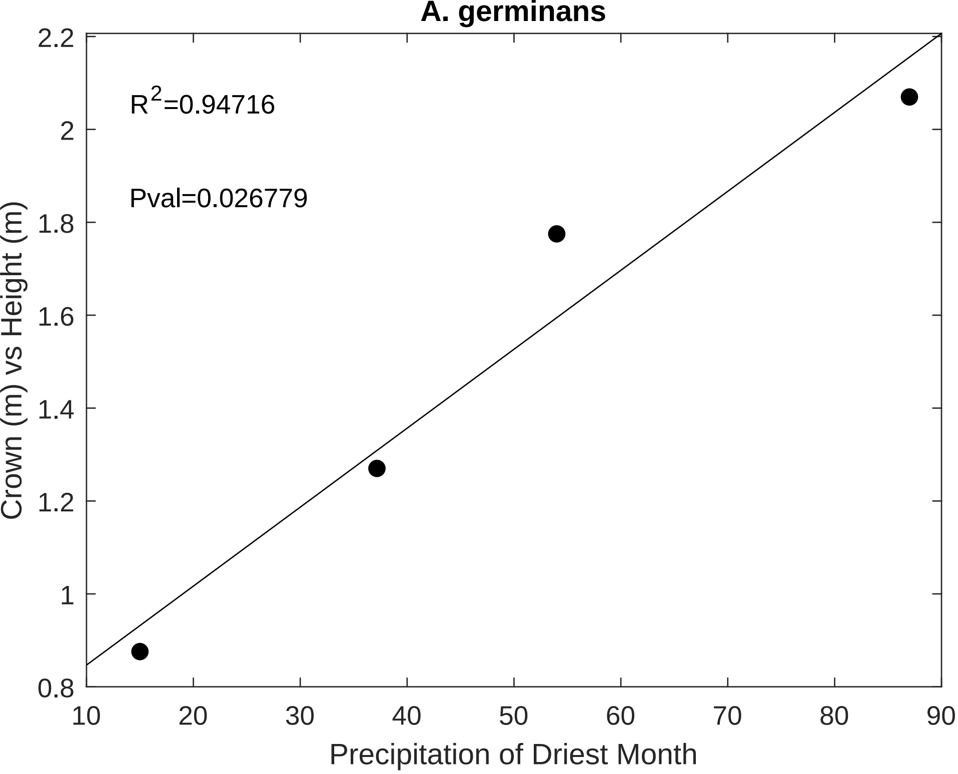
A. germinans
R^2=0.94716
Pval=0.026779
Crown (m) vs Height (m)
Precipitation of Driest Month
0.8
1
1.2
1.4
1.6
1.8
2
2.2
10
20
30
40
50
60
70
80
90

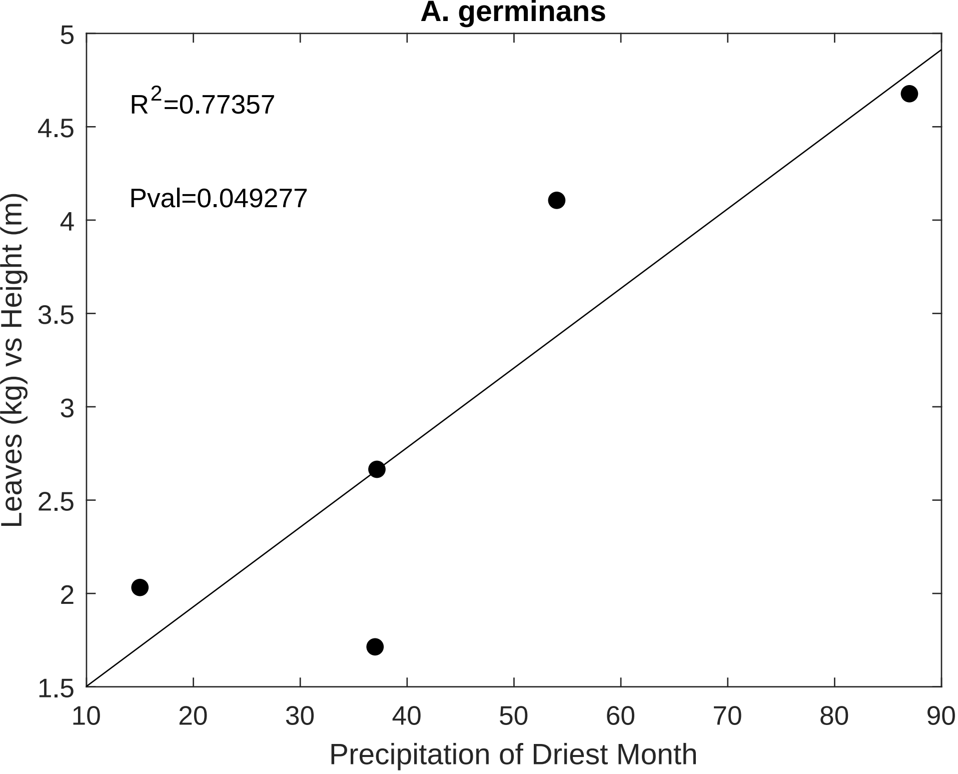
A. germinans
R^2=0.77357
Pval=0.049277
Leaves (kg) vs Height (m)
Precipitation of Driest Month
5
4.5
4
3.5
3
2.5
2
1.5
10
20
30
40
50
60
70
80
90

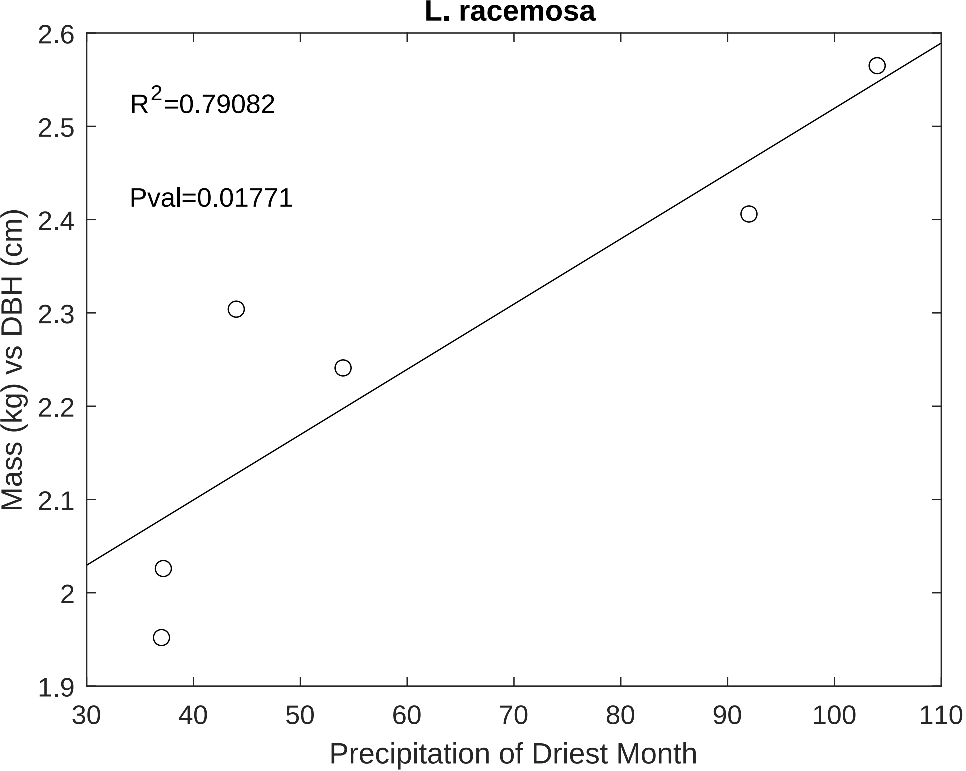
L. racemosa
R2=0.79082
Pval=0.01771
2.6
2.5
2.4
2.3
2.2
2.1
2
1.9
Mass (kg) vs DBH (cm)
30
40
50
60
70
80
90
100
110
Precipitation of Driest Month

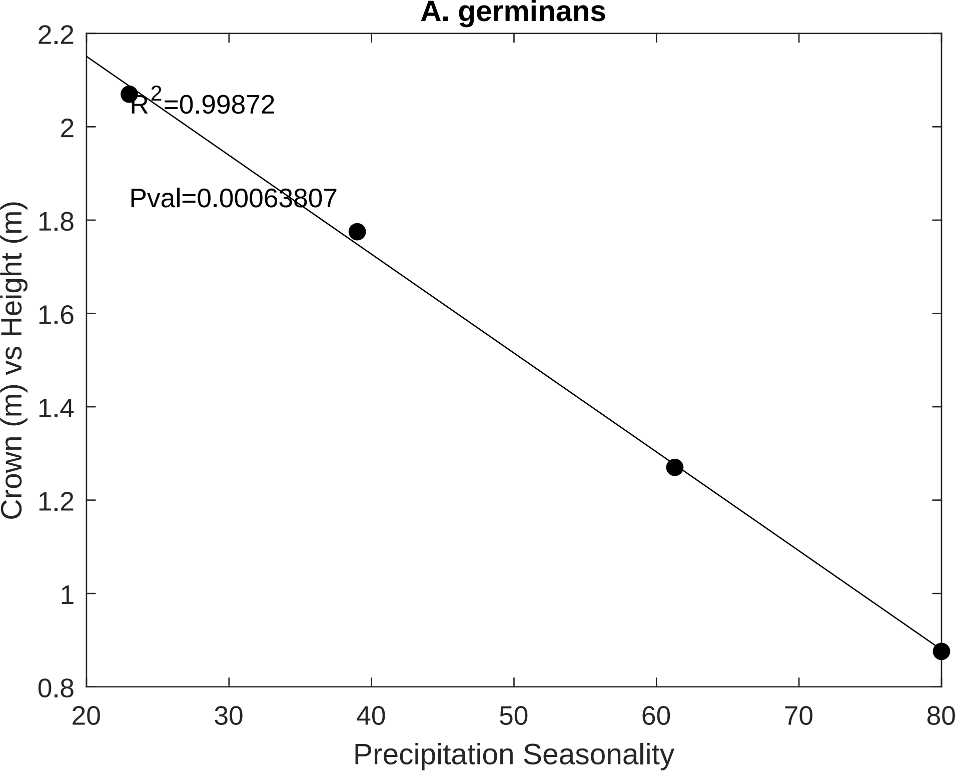

A. germinans
R^2=0.99872
Pval=0.00063807
Crown (m) vs Height (m)
Precipitation Seasonality
2.2
2
1.8
1.6
1.4
1.2
1
0.8
20
30
40
50
60
70
80

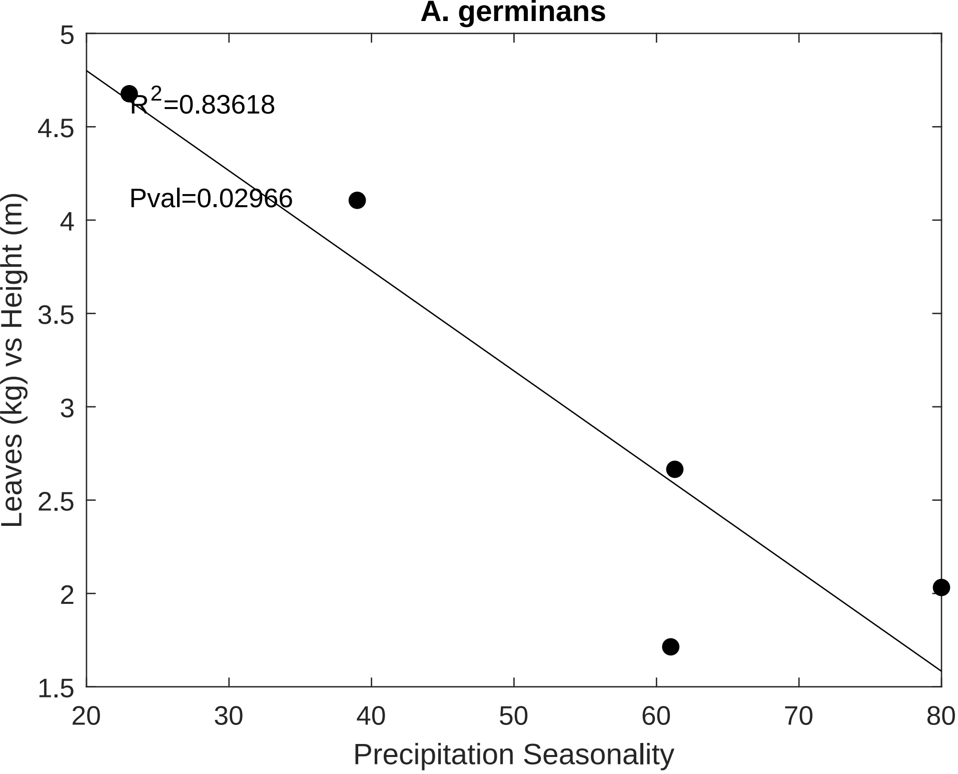
A. germinans
R2=0.83618
Pval=0.02966
Leaves (kg) vs Height (m)
Precipitation Seasonality
5
4.5
4
3.5
3
2.5
2
1.5
20
30
40
50
60
70
80

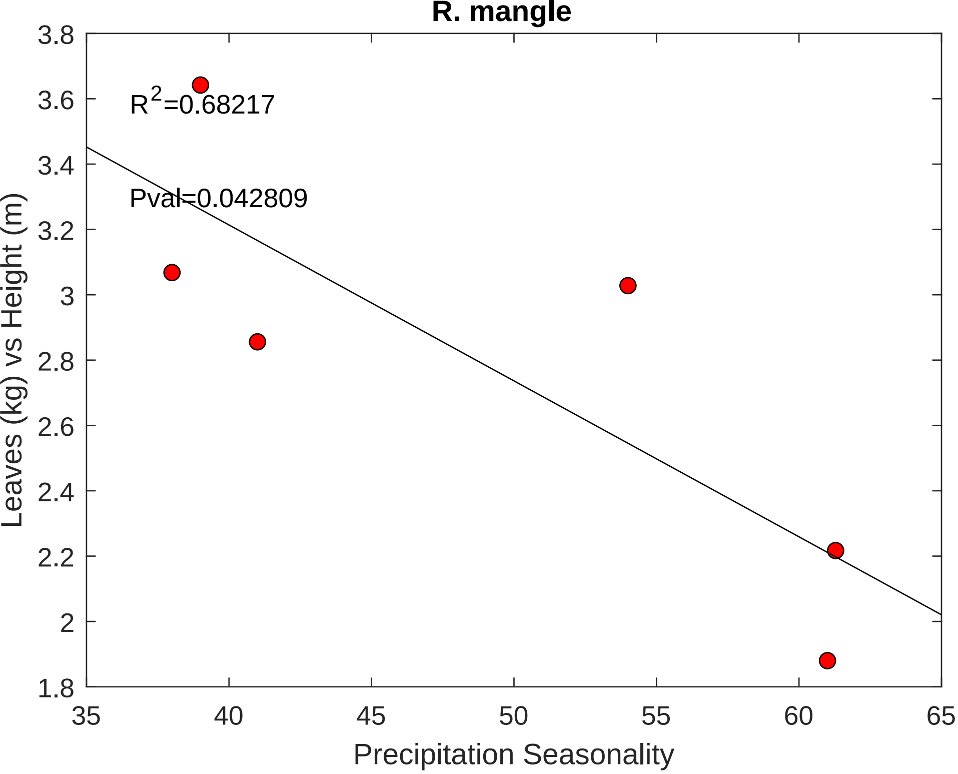
R. mangle
R2=0.68217
Pval=0.042809
Leaves (kg) vs Height (m)
Precipitation Seasonality
3.8
3.6
3.4
3.2
3
2.8
2.6
2.4
2.2
2
1.8
35
40
45
50
55
60
65

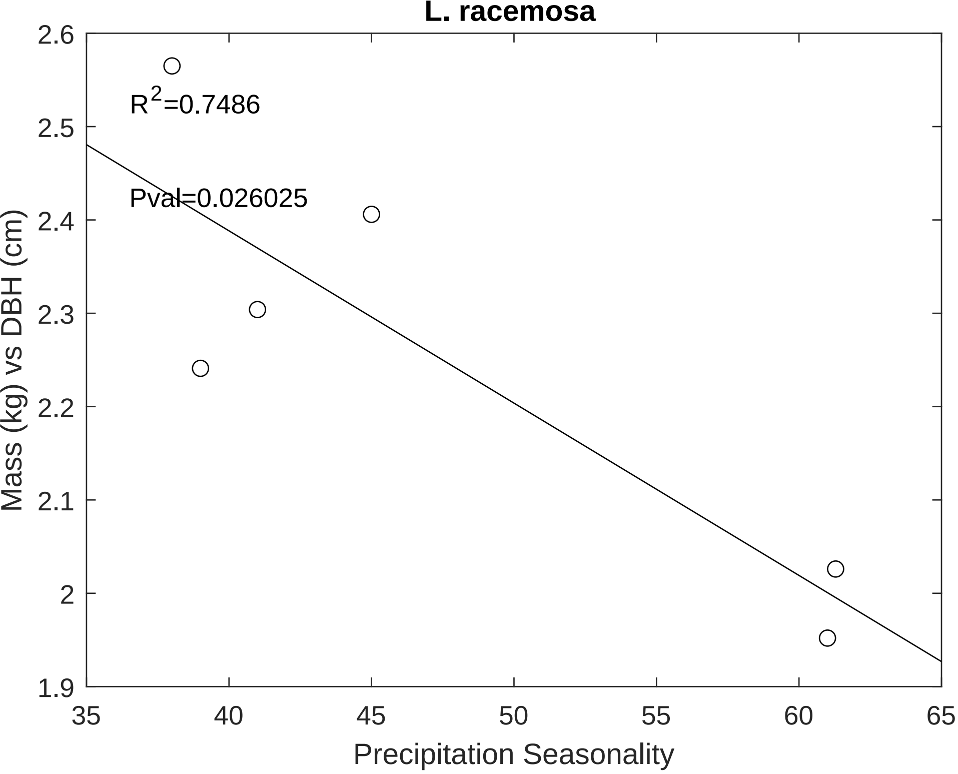
L. racemosa
R2=0.7486
Pval=0.026025
Mass (kg) vs DBH (cm)
Precipitation Seasonality
2.6
2.5
2.4
2.3
2.2
2.1
2
1.9
35
40
45
50
55
60
65

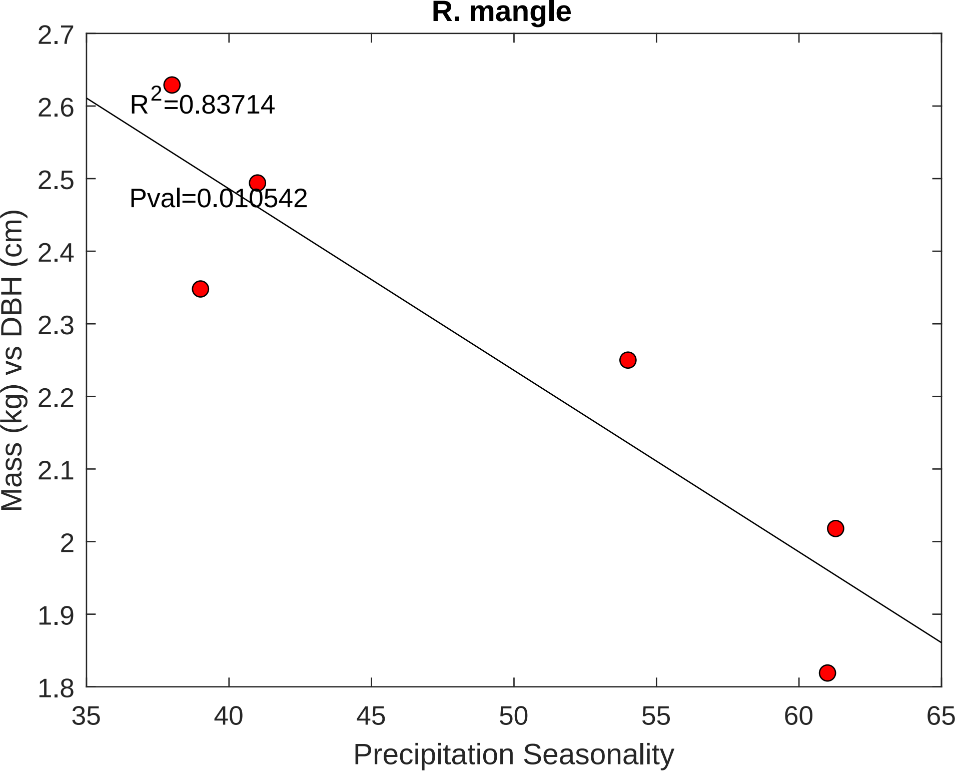
R. mangle
R^2=0.83714
Pval=0.010542
Mass (kg) vs DBH (cm)
Precipitation Seasonality
2.7
2.6
2.5
2.4
2.3
2.2
2.1
2
1.9
1.8
35
40
45
50
55
60
65

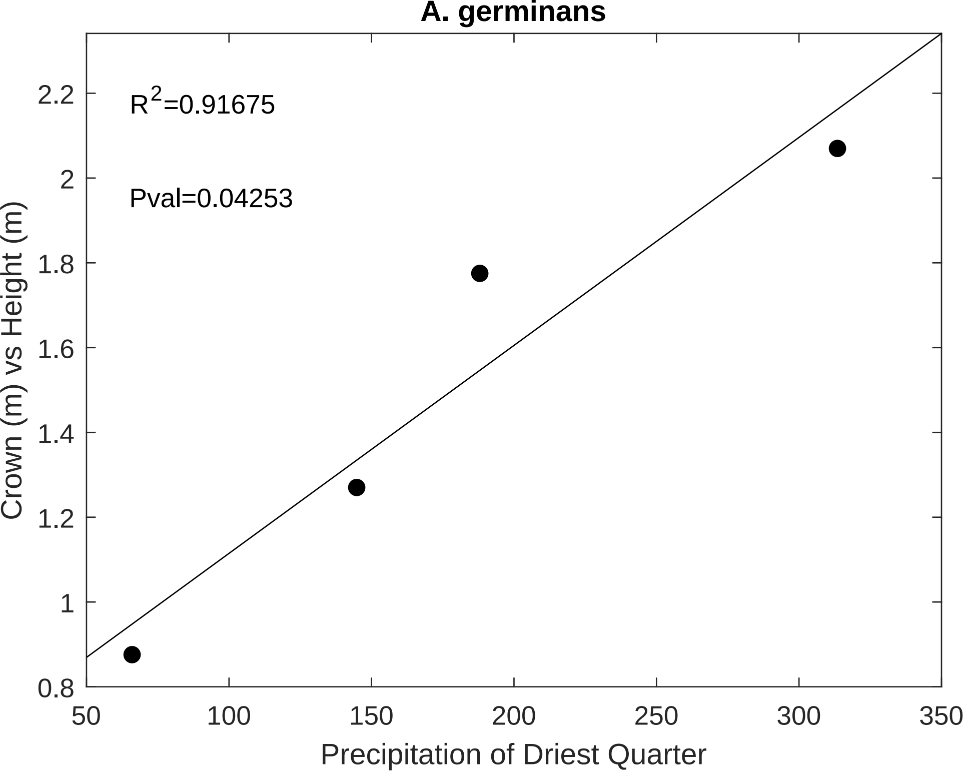
A. germinans
R2=0.91675
Pval=0.04253
Crown (m) vs Height (m)
Precipitation of Driest Quarter
0.8
1
1.2
1.4
1.6
1.8
2
2.2
50
100
150
200
250
300
350

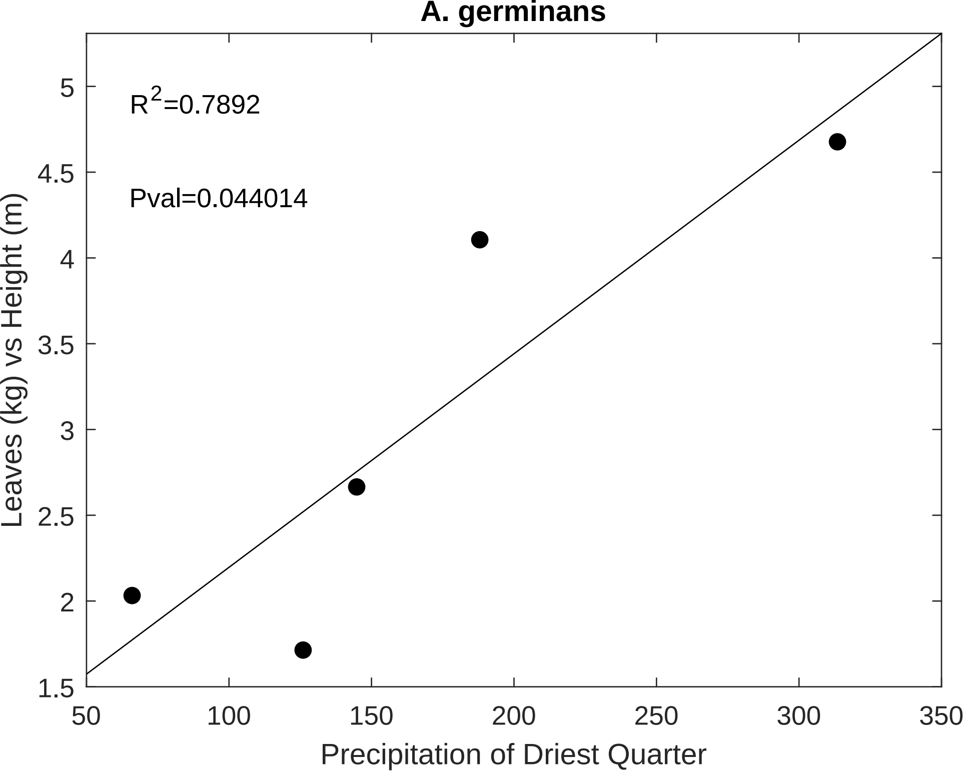
A. germinans
R^2=0.7892
Pval=0.044014
Leaves (kg) vs Height (m)
Precipitation of Driest Quarter
5
4.5
4
3.5
3
2.5
2
1.5
50
100
150
200
250
300
350
