# Supplementary material for: Global Data Compilation Across Climate Gradients Supports the Use of Common Allometric Equations for Three Transatlantic Mangrove Species
Source: Ecol Evol. 2024 Nov 20;14(11):e70577. doi: 10.1002/ece3.70577 (PMC11578852; doi:10.1002/ece3.70577)
Supplement: Supplementary file 1 — Figures S1–S147. [file ECE3-14-e70577-s002.zip › Figs S66_S147 Intercepts.pdf]

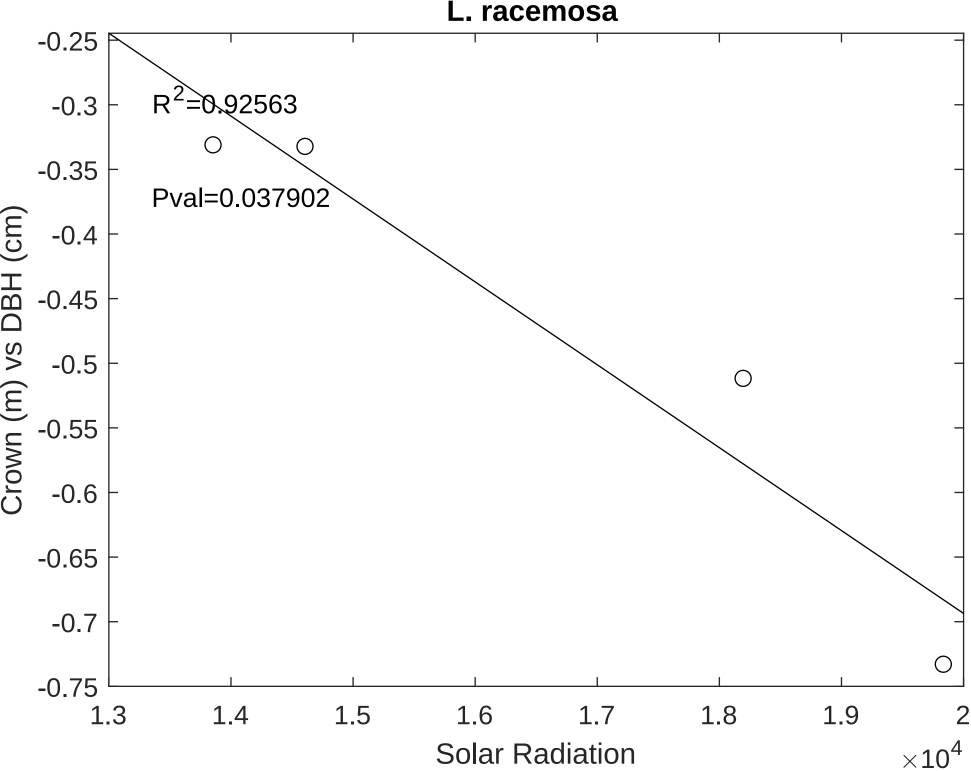
L. racemosa
R^2=0.92563
Pval=0.037902
Crown (m) vs DBH (cm)
Solar Radiation
$\times 10^4$
-0.25
-0.3
-0.35
-0.4
-0.45
-0.5
-0.55
-0.6
-0.65
-0.7
-0.75
1.3
1.4
1.5
1.6
1.7
1.8
1.9
2

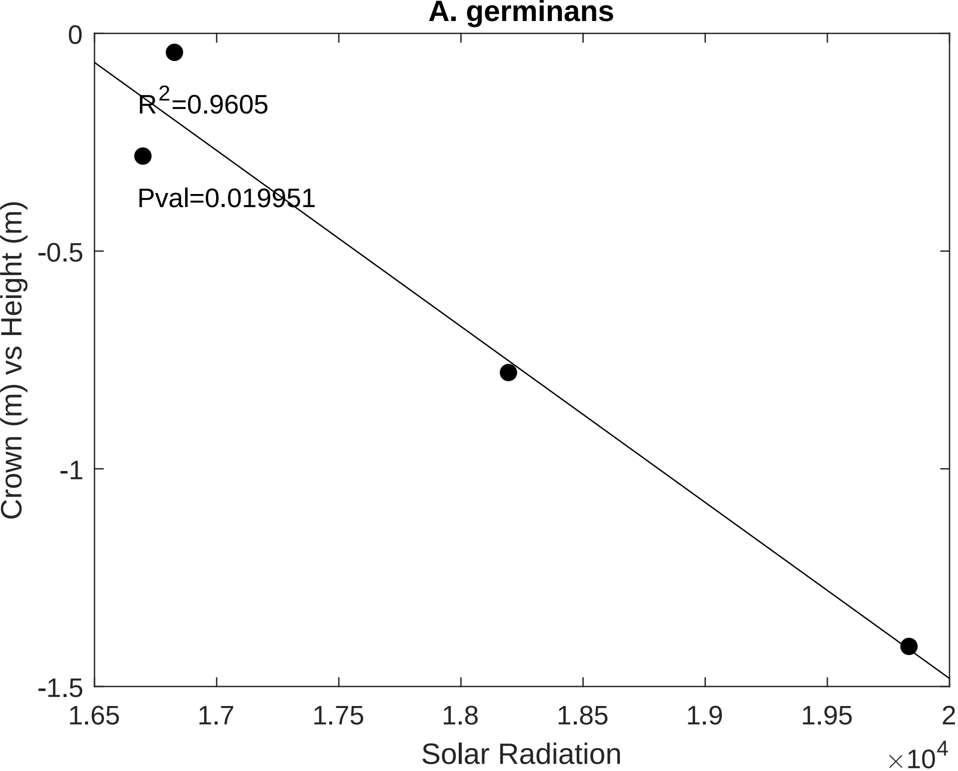

A. germinans
R2=0.9605
Pval=0.019951
Crown (m) vs Height (m)
Solar Radiation
×10^4
0
-0.5
-1
-1.5
1.65
1.7
1.75
1.8
1.85
1.9
1.95
2

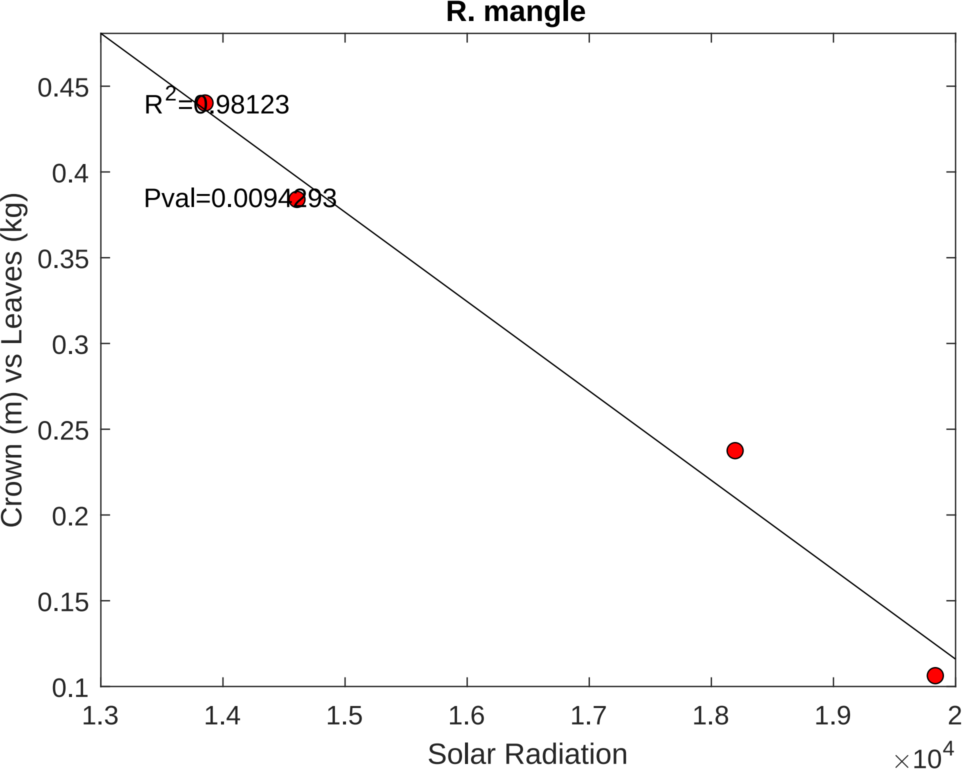

R. mangle
R^2=0.98123
Pval=0.0094293
Crown (m) vs Leaves (kg)
0.45
0.4
0.35
0.3
0.25
0.2
0.15
0.1
1.3
1.4
1.5
1.6
1.7
1.8
1.9
2
Solar Radiation
×10^4

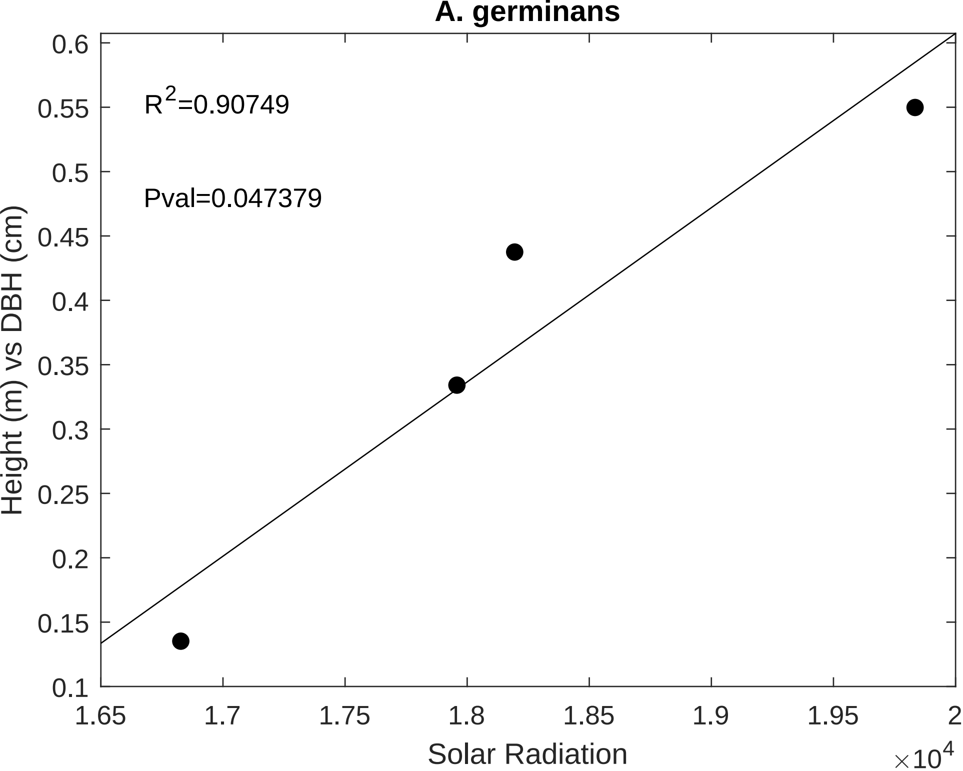

A. germinans
R2=0.90749
Pval=0.047379
Height (m) vs DBH (cm)
0.6
0.55
0.5
0.45
0.4
0.35
0.3
0.25
0.2
0.15
0.1
1.65
1.7
1.75
1.8
1.85
1.9
1.95
2
Solar Radiation
×10^4

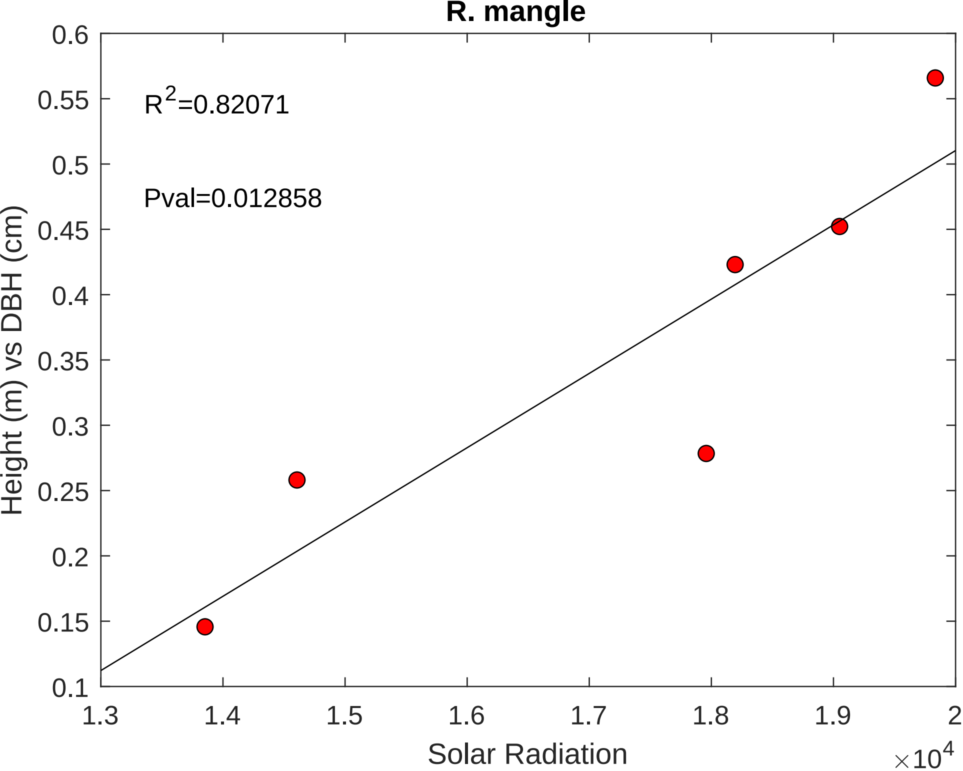
R. mangle
R2=0.82071
Pval=0.012858
Height (m) vs DBH (cm)
0.6
0.55
0.5
0.45
0.4
0.35
0.3
0.25
0.2
0.15
0.1
1.3
1.4
1.5
1.6
1.7
1.8
1.9
2
Solar Radiation
×10^4

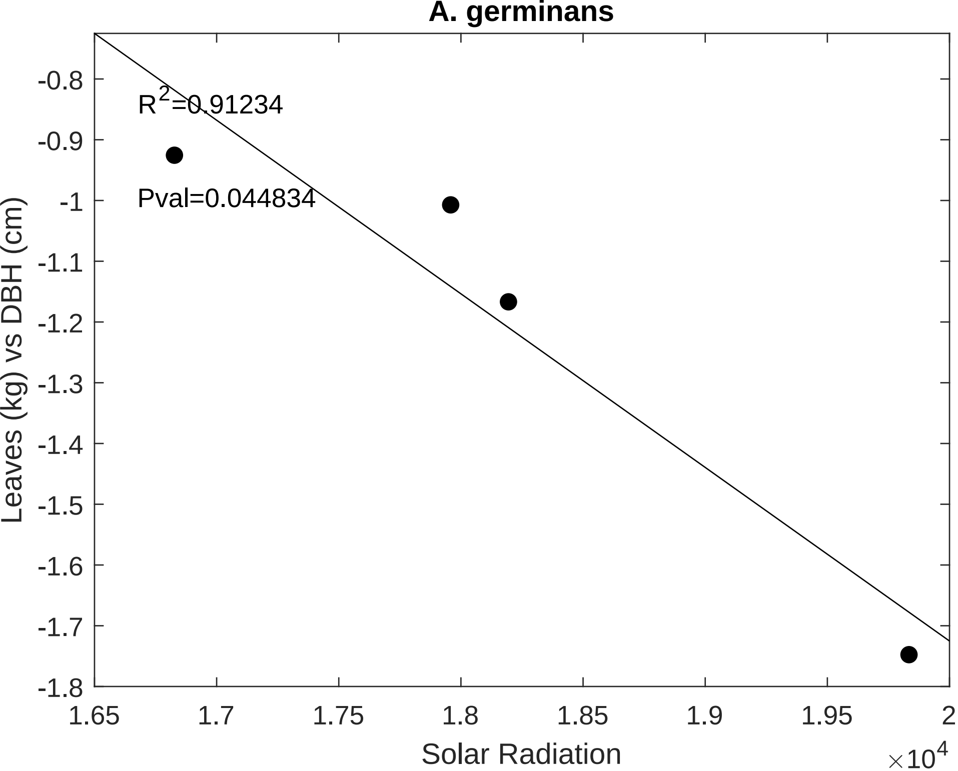

A. germinans
R2=0.91234
Pval=0.044834
Leaves (kg) vs DBH (cm)
-0.8
-0.9
-1
-1.1
-1.2
-1.3
-1.4
-1.5
-1.6
-1.7
-1.8
1.65
1.7
1.75
1.8
1.85
1.9
1.95
2
Solar Radiation
×10^4

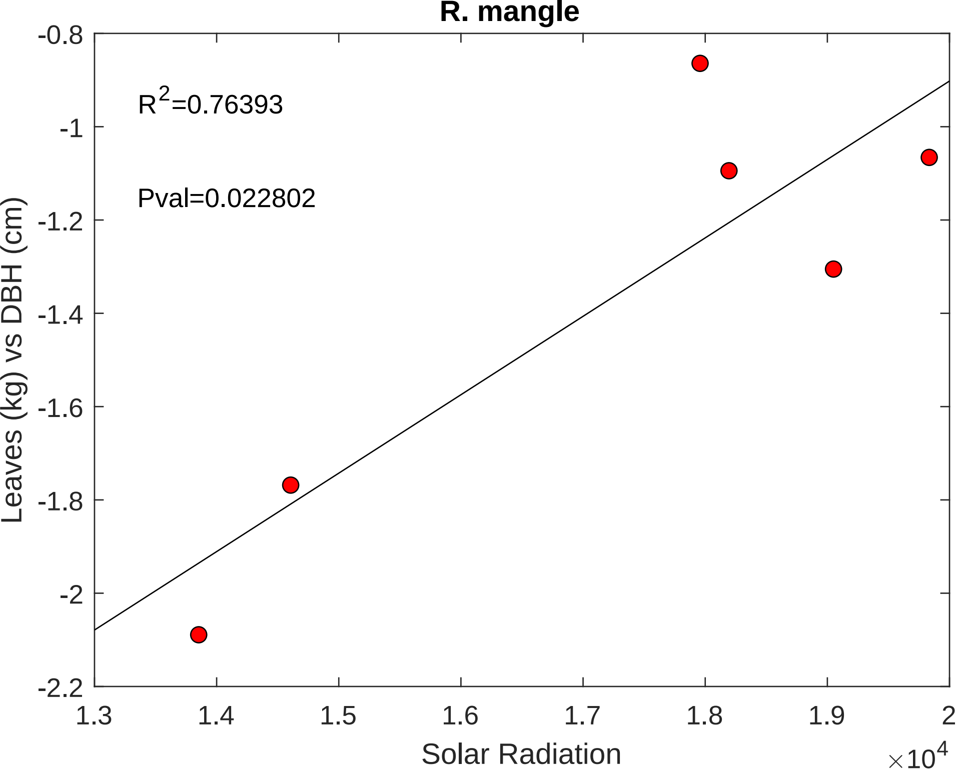

R. mangle
R^2=0.76393
Pval=0.022802
Leaves (kg) vs DBH (cm)
-0.8
-1
-1.2
-1.4
-1.6
-1.8
-2
-2.2
1.3
1.4
1.5
1.6
1.7
1.8
1.9
2
Solar Radiation
$\times10^4$

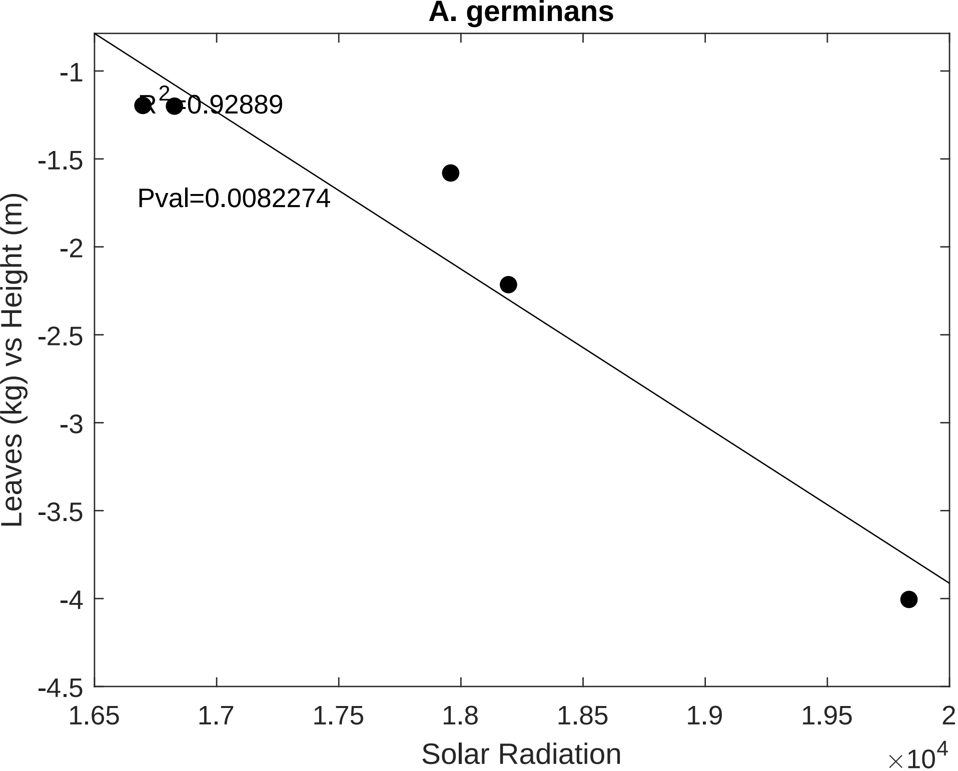

A. germinans
R^2=0.92889
Pval=0.0082274
Leaves (kg) vs Height (m)
-1
-1.5
-2
-2.5
-3
-3.5
-4
-4.5
1.65
1.7
1.75
1.8
1.85
1.9
1.95
2
Solar Radiation
$\times 10^4$

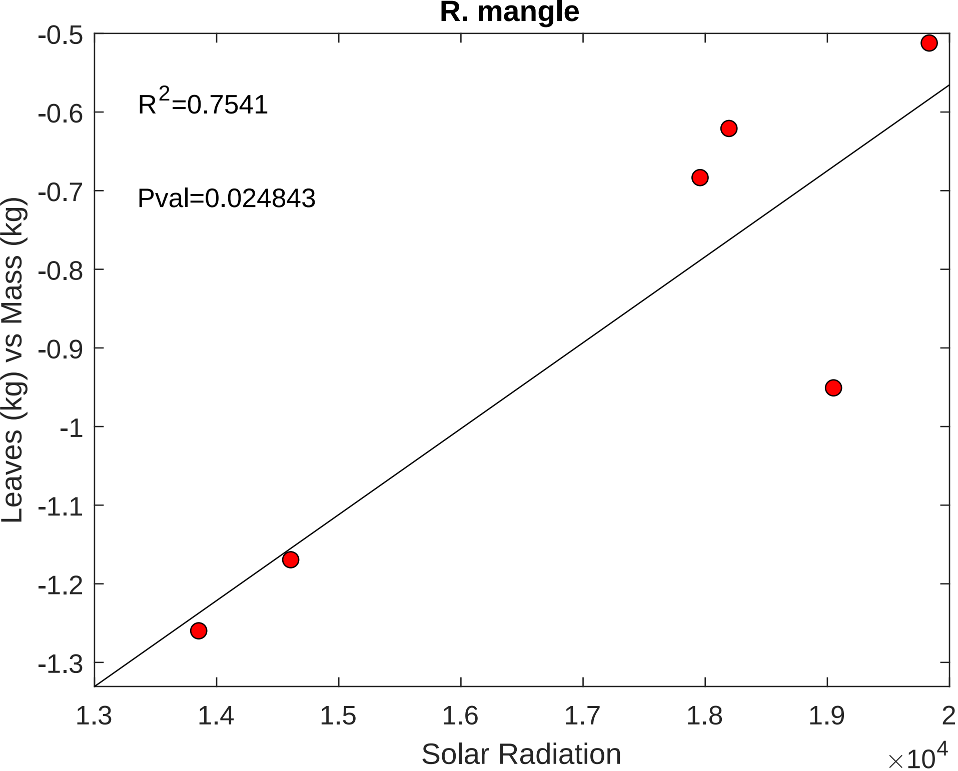

R. mangle
R2=0.7541
Pval=0.024843
Leaves (kg) vs Mass (kg)
-0.5
-0.6
-0.7
-0.8
-0.9
-1
-1.1
-1.2
-1.3
1.3
1.4
1.5
1.6
1.7
1.8
1.9
2
Solar Radiation
×10^4

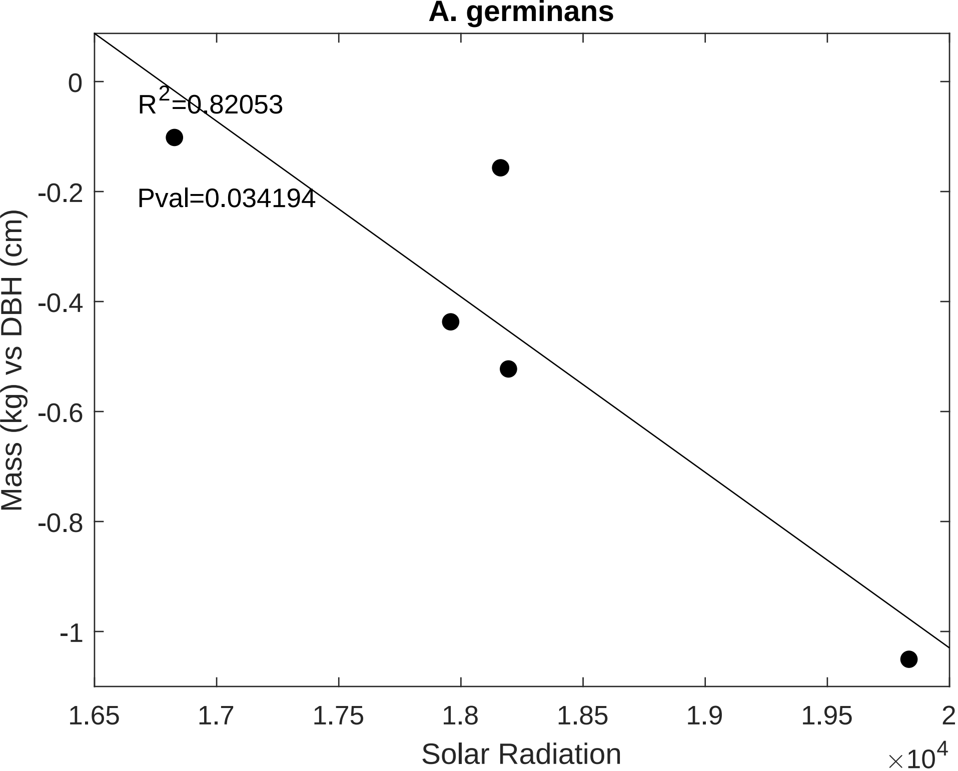
A. germinans
R^2=0.82053
Pval=0.034194
0
-0.2
-0.4
-0.6
-0.8
-1
Mass (kg) vs DBH (cm)
1.65
1.7
1.75
1.8
1.85
1.9
1.95
2
Solar Radiation
$\times10^4$

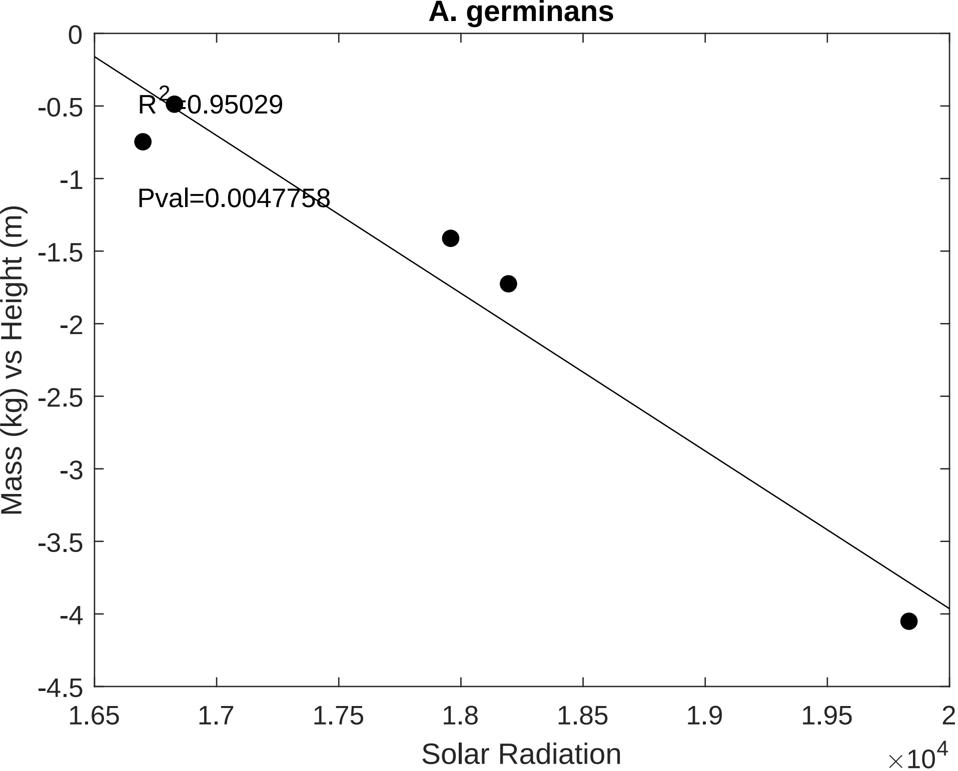

A. germinans
R 2 =0.95029
Pval=0.0047758
Mass (kg) vs Height (m)
0
-0.5
-1
-1.5
-2
-2.5
-3
-3.5
-4
-4.5
1.65
1.7
1.75
1.8
1.85
1.9
1.95
2
Solar Radiation
×10 4

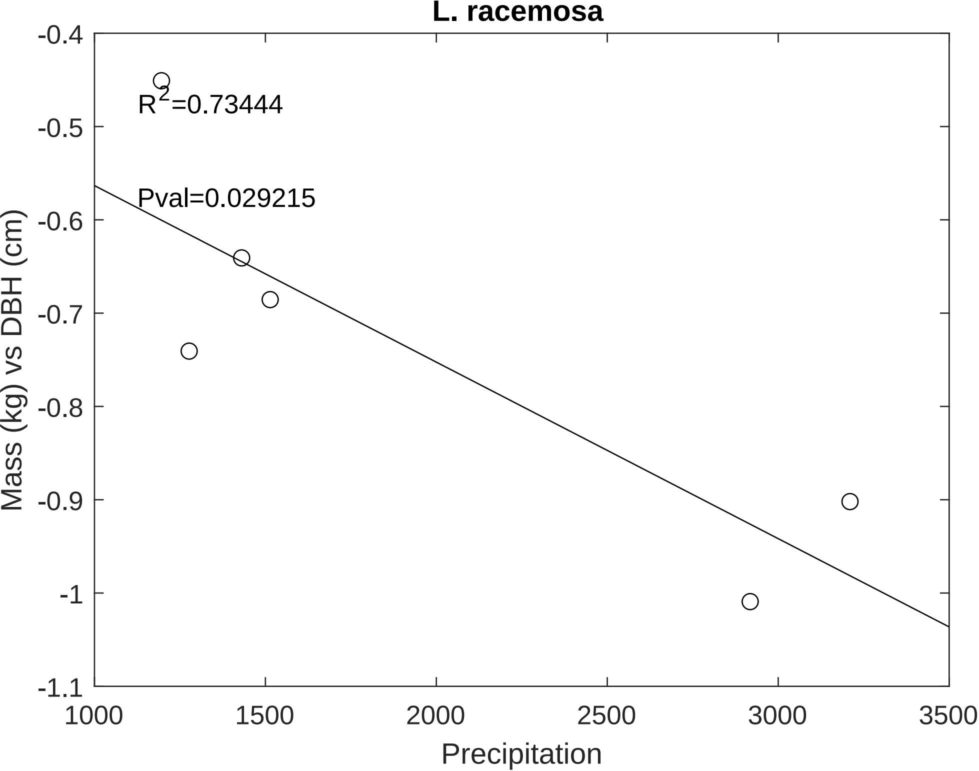

L. racemosa
R^2=0.73444
Pval=0.029215
-0.4
-0.5
-0.6
-0.7
-0.8
-0.9
-1
-1.1
Mass (kg) vs DBH (cm)
1000
1500
2000
2500
3000
3500
Precipitation

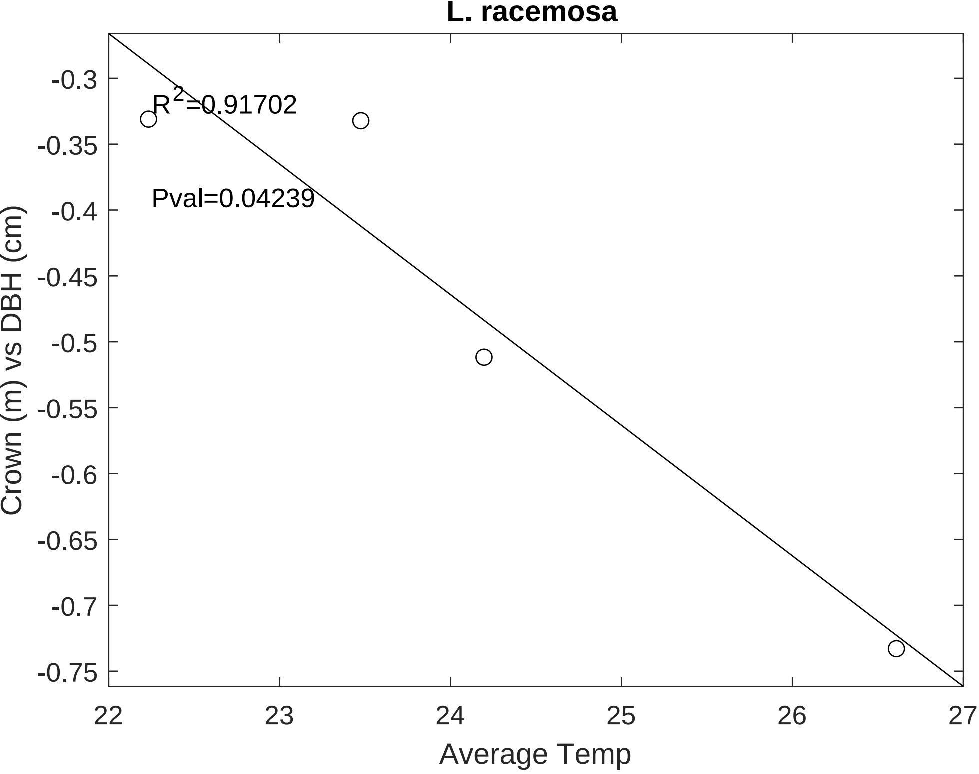

L. racemosa
R^2=0.91702
Pval=0.04239
-0.3
-0.35
-0.4
-0.45
-0.5
-0.55
-0.6
-0.65
-0.7
-0.75
Crown (m) vs DBH (cm)
22
23
24
25
26
27
Average Temp

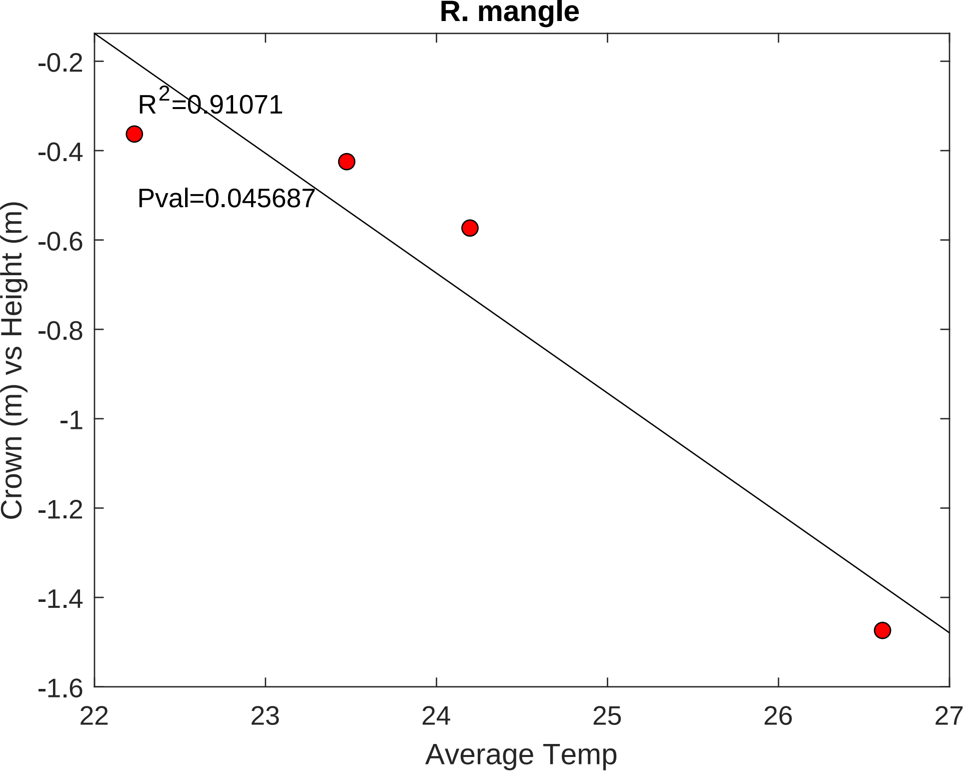

R. mangle
R^2=0.91071
Pval=0.045687
Crown (m) vs Height (m)
-0.2
-0.4
-0.6
-0.8
-1
-1.2
-1.4
-1.6
22
23
24
25
26
27
Average Temp

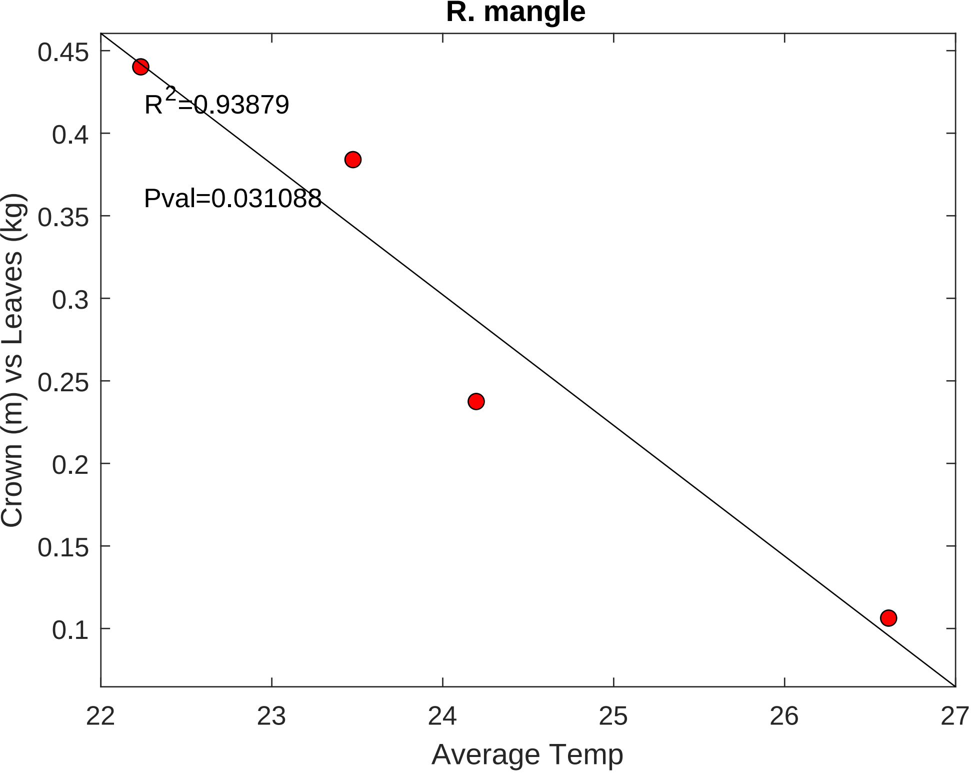
R. mangle
R2=0.93879
Pval=0.031088
Crown (m) vs Leaves (kg)
Average Temp
0.45
0.4
0.35
0.3
0.25
0.2
0.15
0.1
22
23
24
25
26
27

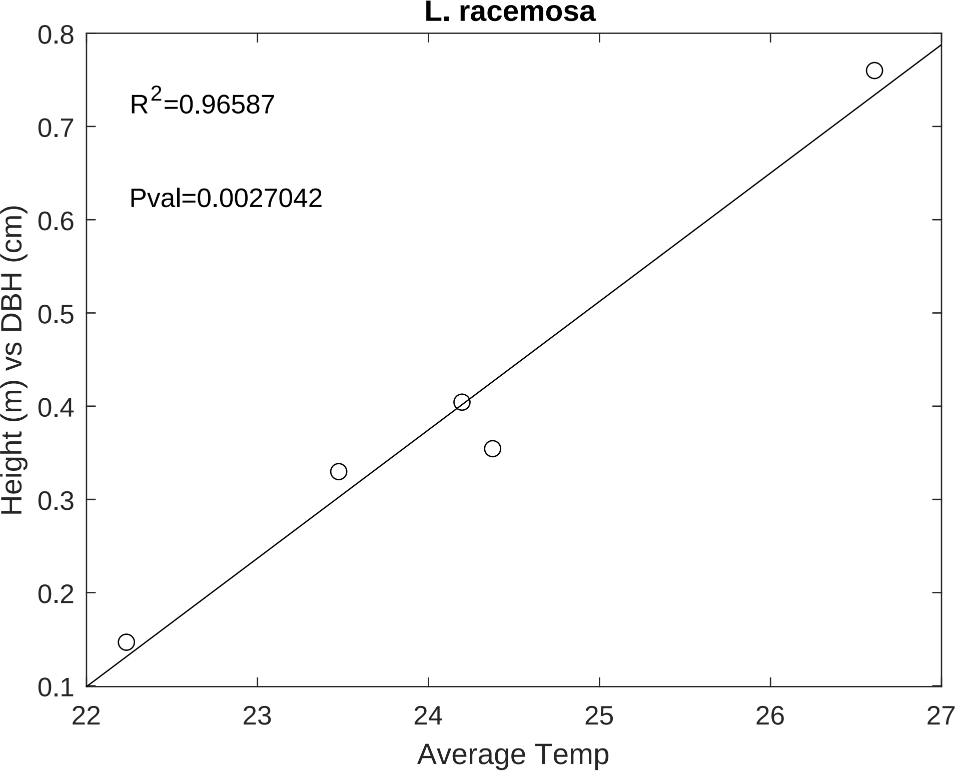

L. racemosa
R2=0.96587
Pval=0.0027042
Height (m) vs DBH (cm)
Average Temp
0.1
0.2
0.3
0.4
0.5
0.6
0.7
0.8
22
23
24
25
26
27

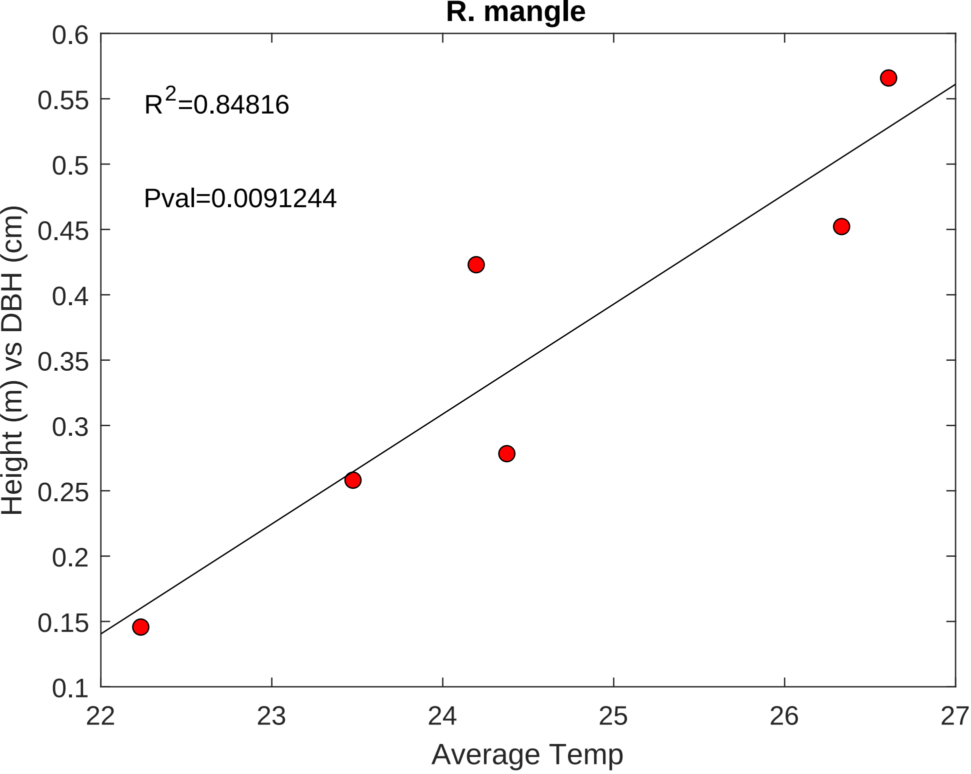
R. mangle
R^2=0.84816
Pval=0.0091244
Height (m) vs DBH (cm)
Average Temp
0.6
0.55
0.5
0.45
0.4
0.35
0.3
0.25
0.2
0.15
0.1
22
23
24
25
26
27

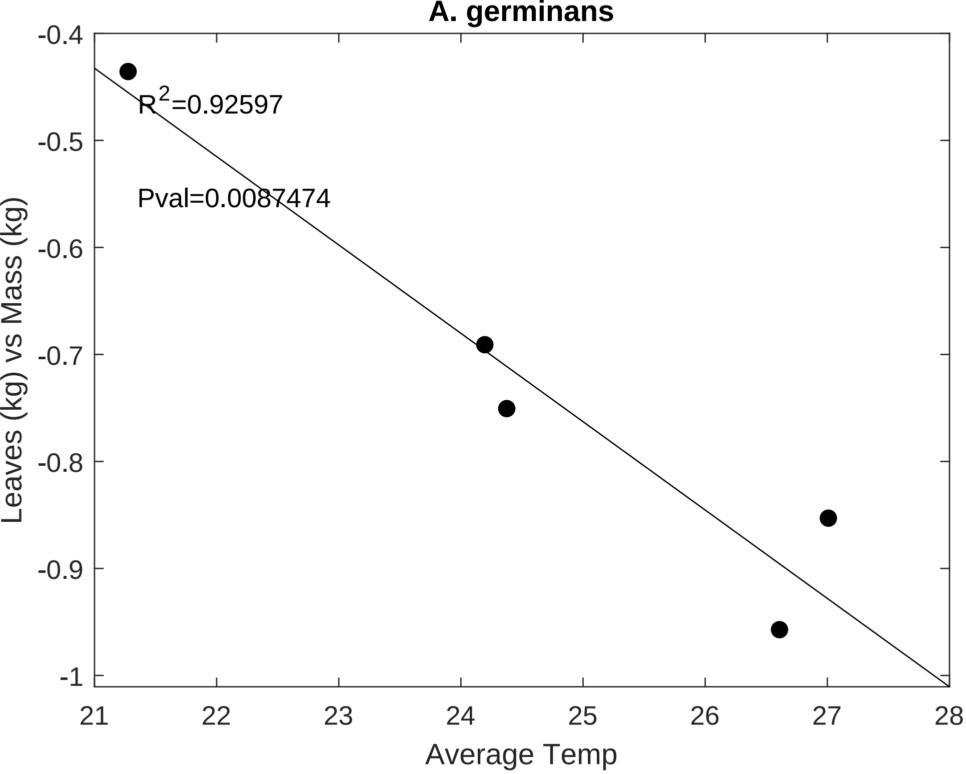

A. germinans
-0.4
-0.5
-0.6
-0.7
-0.8
-0.9
-1
R2=0.92597
Pval=0.0087474
Leaves (kg) vs Mass (kg)
21
22
23
24
25
26
27
28
Average Temp

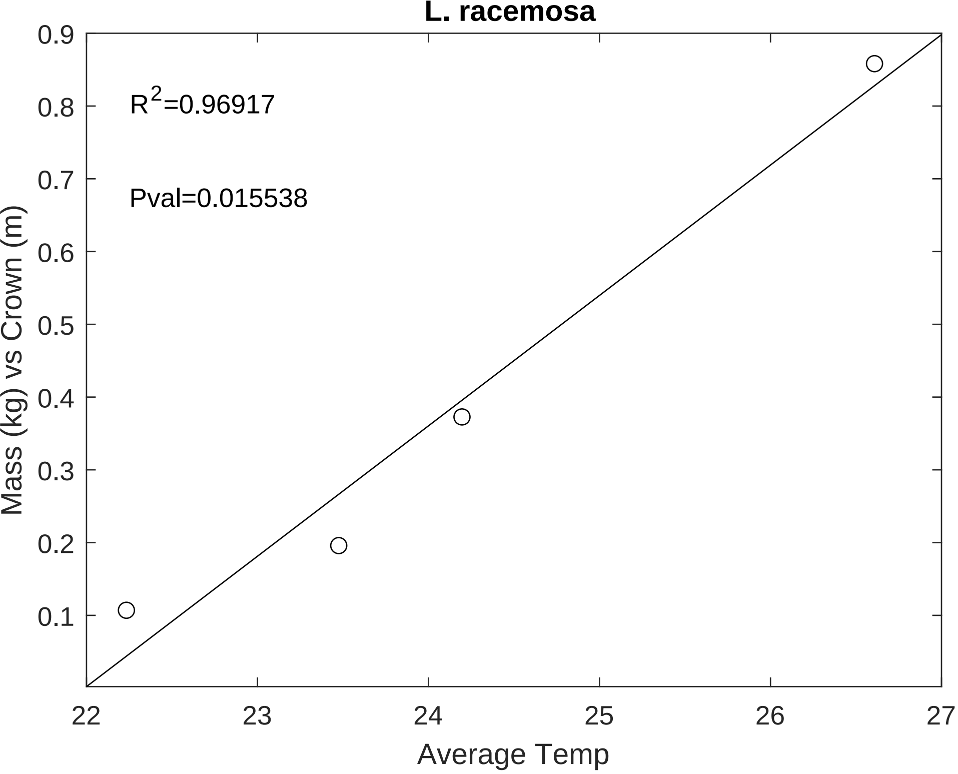

L. racemosa
R^2=0.96917
Pval=0.015538
Mass (kg) vs Crown (m)
Average Temp
0.9
0.8
0.7
0.6
0.5
0.4
0.3
0.2
0.1
22
23
24
25
26
27

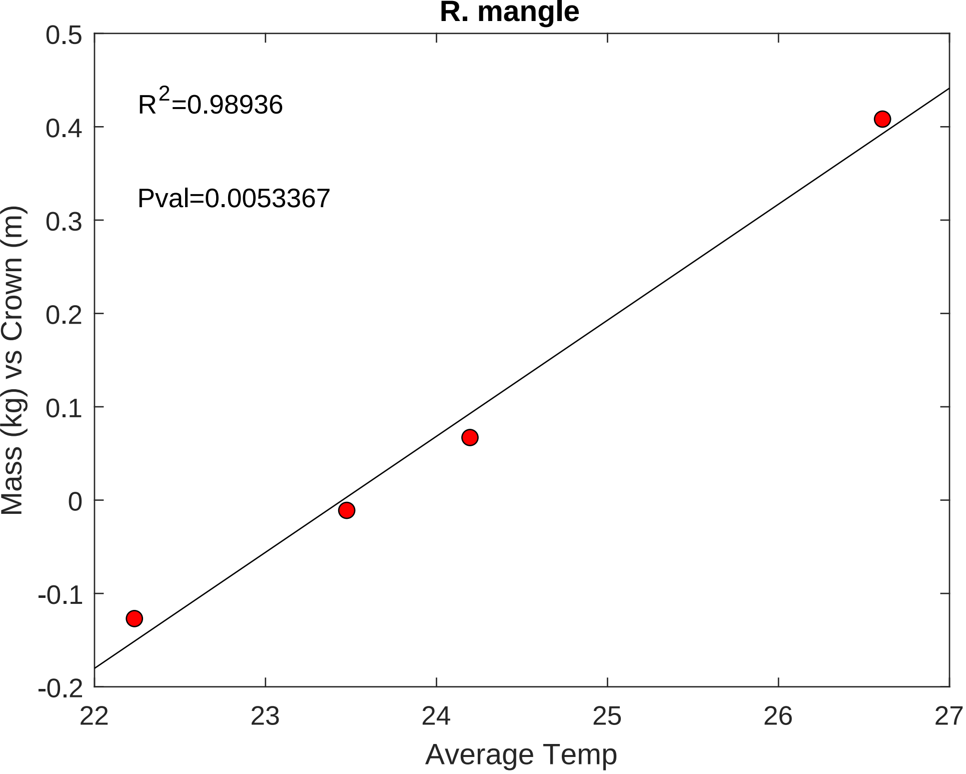

R. mangle
R^2=0.98936
Pval=0.0053367
Mass (kg) vs Crown (m)
Average Temp
0.5
0.4
0.3
0.2
0.1
0
-0.1
-0.2
22
23
24
25
26
27

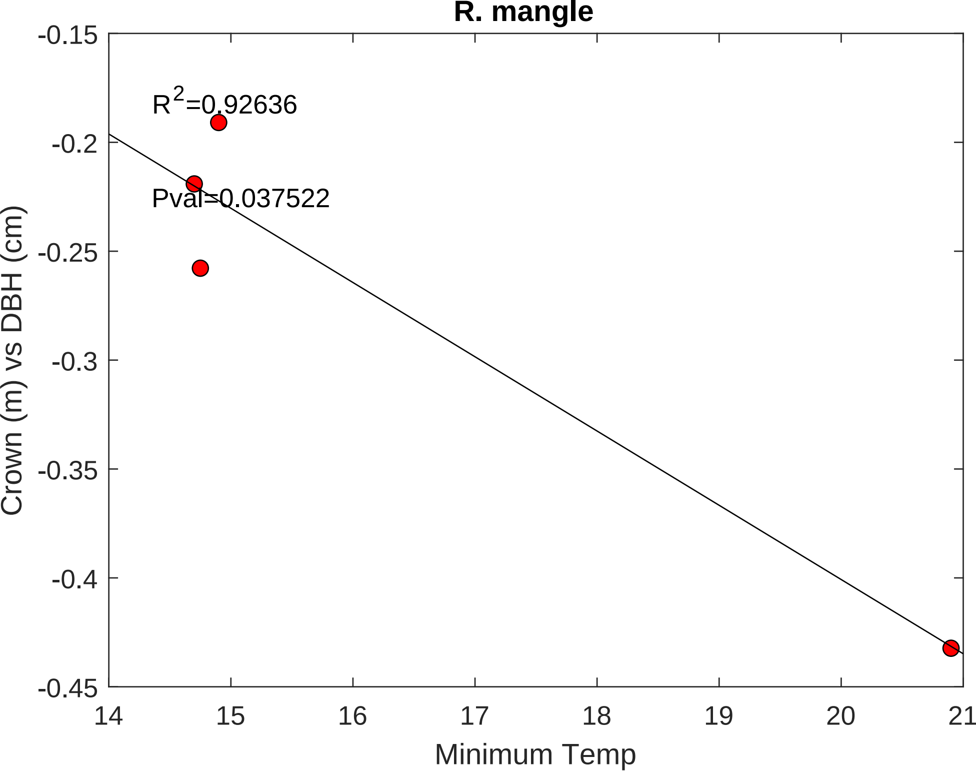
R. mangle
R^2=0.92636
Pval=0.037522
Crown (m) vs DBH (cm)
Minimum Temp
-0.15
-0.2
-0.25
-0.3
-0.35
-0.4
-0.45
14
15
16
17
18
19
20
21

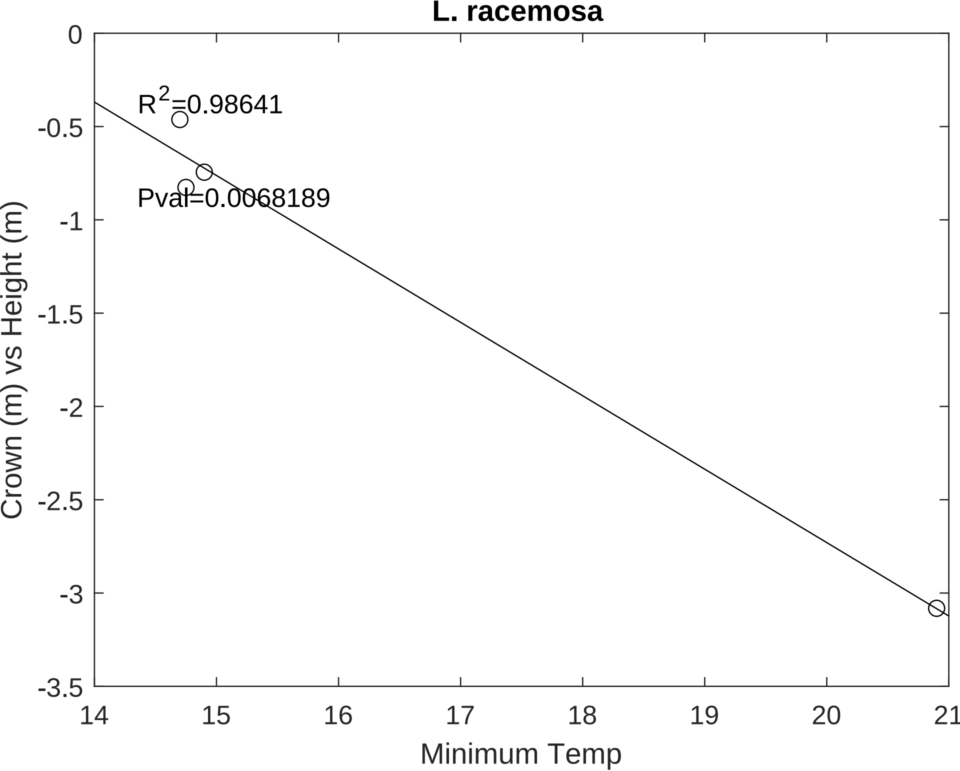

L. racemosa
R2=0.98641
Pval=0.0068189
Crown (m) vs Height (m)
Minimum Temp
0
-0.5
-1
-1.5
-2
-2.5
-3
-3.5
14
15
16
17
18
19
20
21

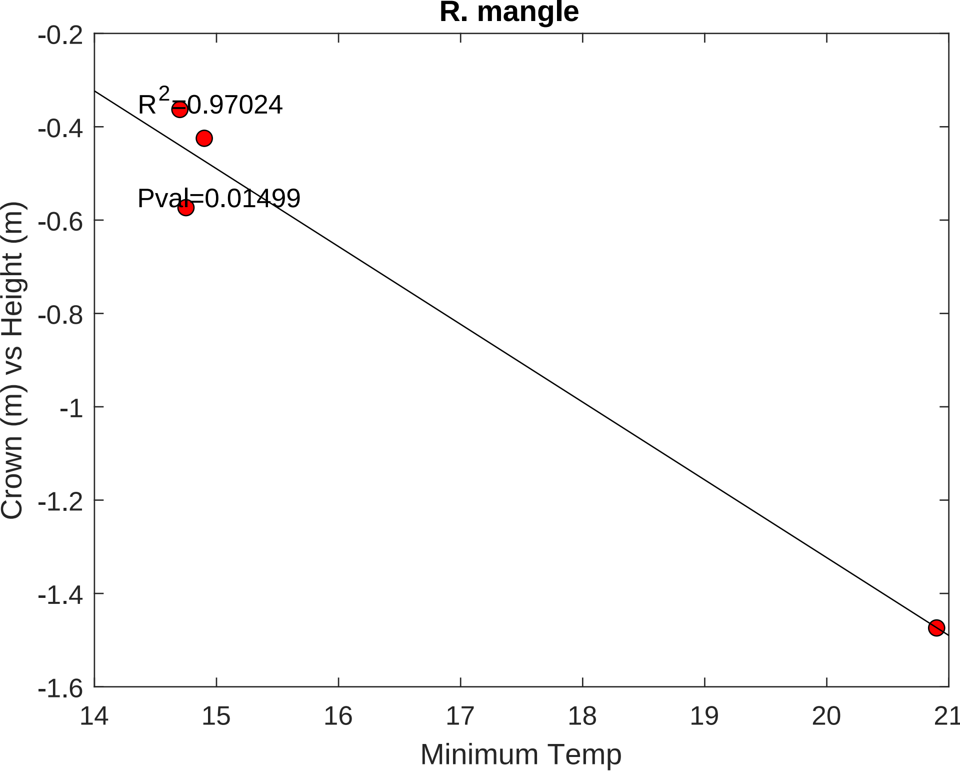
R. mangle
R^2=0.97024
Pval=0.01499
-0.2
-0.4
-0.6
-0.8
-1
-1.2
-1.4
-1.6
14
15
16
17
18
19
20
21
Crown (m) vs Height (m)
Minimum Temp

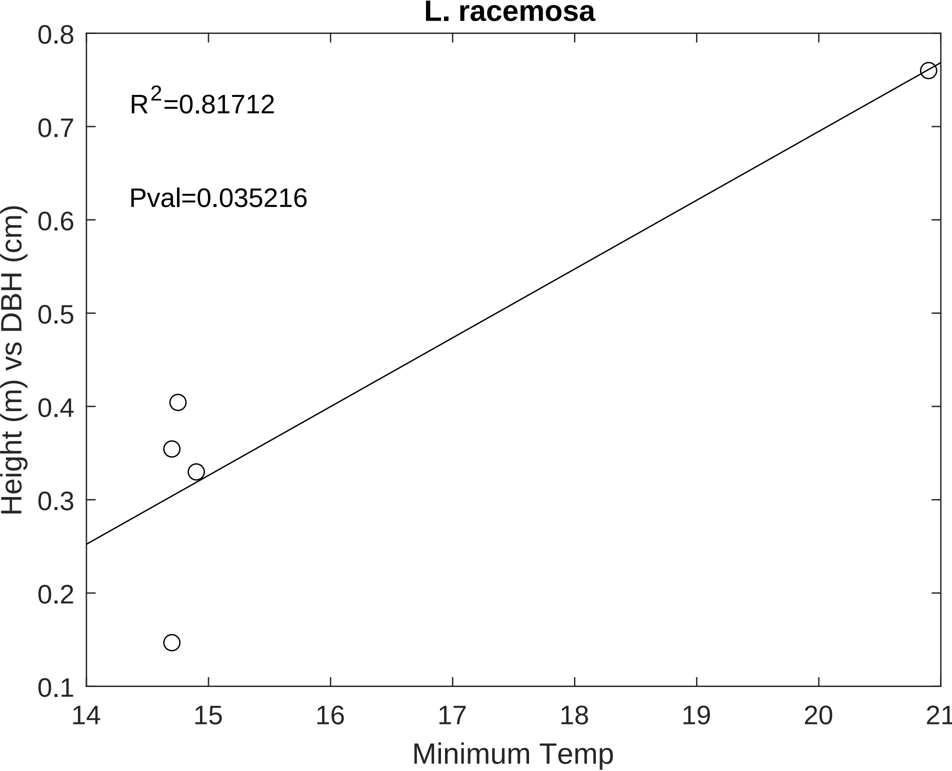

L. racemosa
R2=0.81712
Pval=0.035216
Height (m) vs DBH (cm)
Minimum Temp
0.1
0.2
0.3
0.4
0.5
0.6
0.7
0.8
14
15
16
17
18
19
20
21

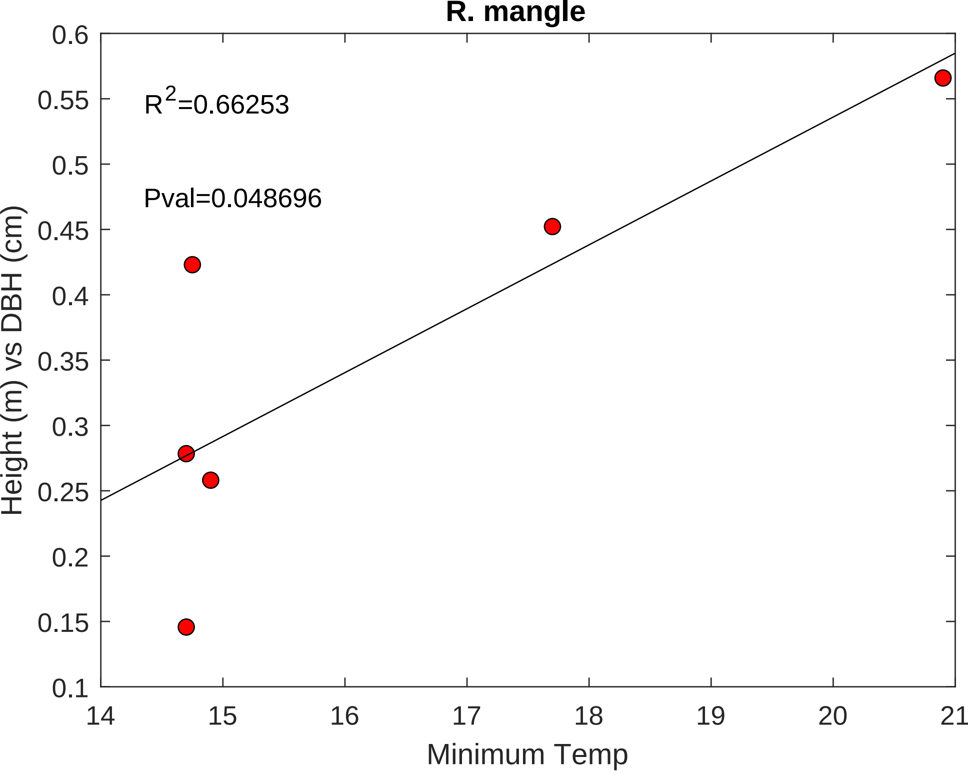
R. mangle
R^2=0.66253
Pval=0.048696
Height (m) vs DBH (cm)
Minimum Temp
0.6
0.55
0.5
0.45
0.4
0.35
0.3
0.25
0.2
0.15
0.1
14
15
16
17
18
19
20
21

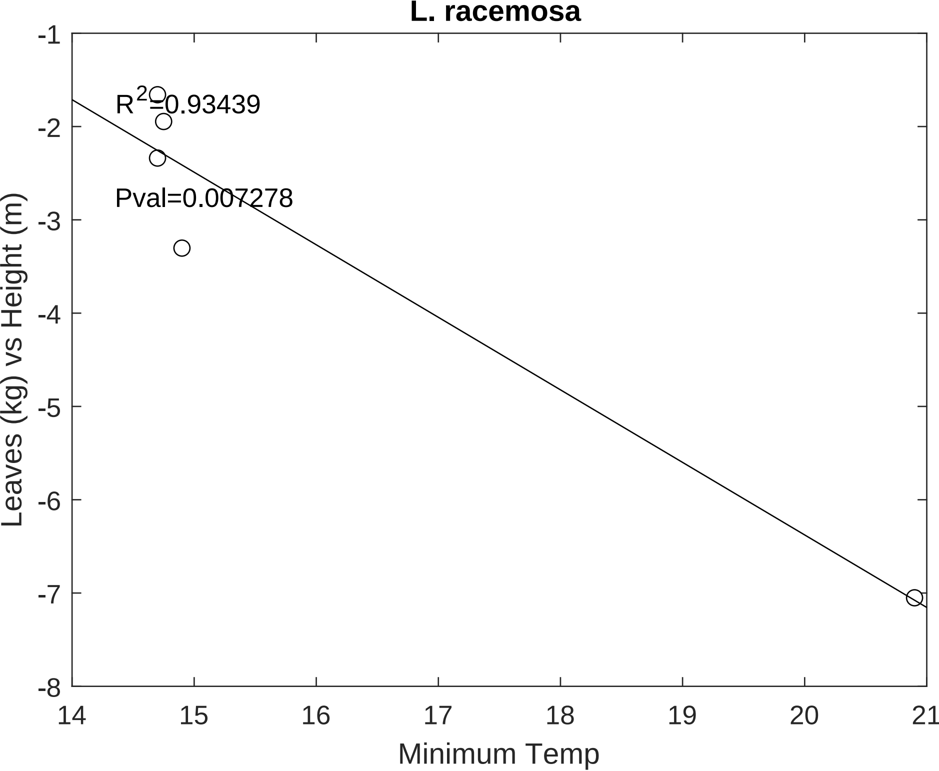

L. racemosa
R2=0.93439
Pval=0.007278
Leaves (kg) vs Height (m)
Minimum Temp
-1
-2
-3
-4
-5
-6
-7
-8
14
15
16
17
18
19
20
21

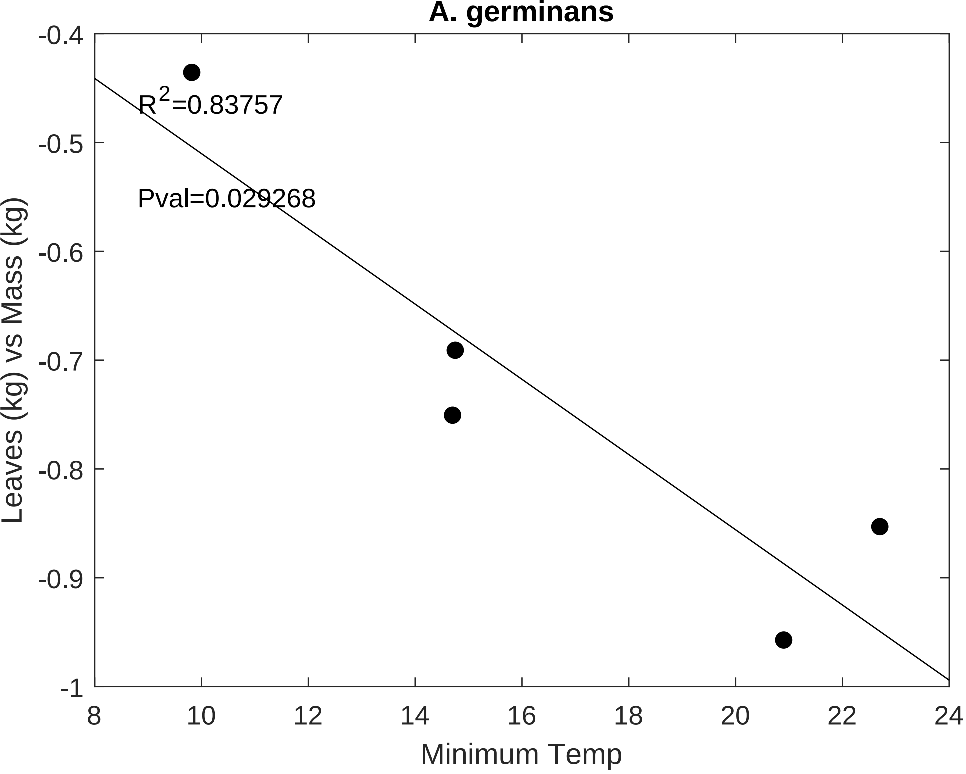

A. germinans
R2=0.83757
Pval=0.029268
-0.4
-0.5
-0.6
-0.7
-0.8
-0.9
-1
8
10
12
14
16
18
20
22
24
Leaves (kg) vs Mass (kg)
Minimum Temp

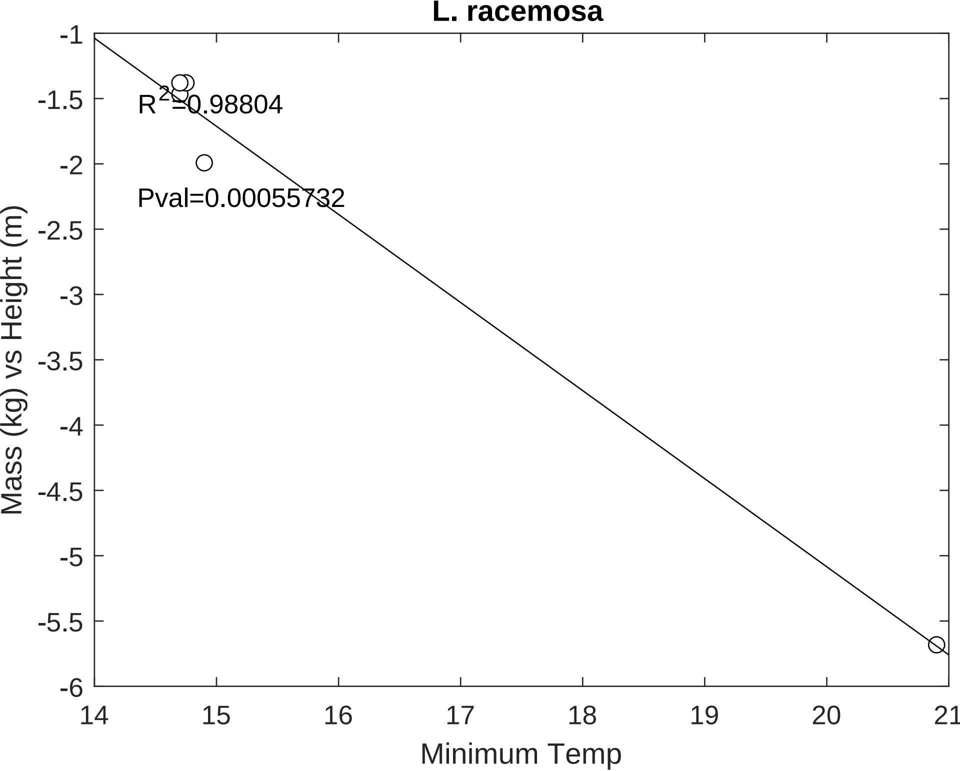

L. racemosa
R^2=0.98804
Pval=0.00055732
Mass (kg) vs Height (m)
Minimum Temp
-1
-1.5
-2
-2.5
-3
-3.5
-4
-4.5
-5
-5.5
-6
14
15
16
17
18
19
20
21

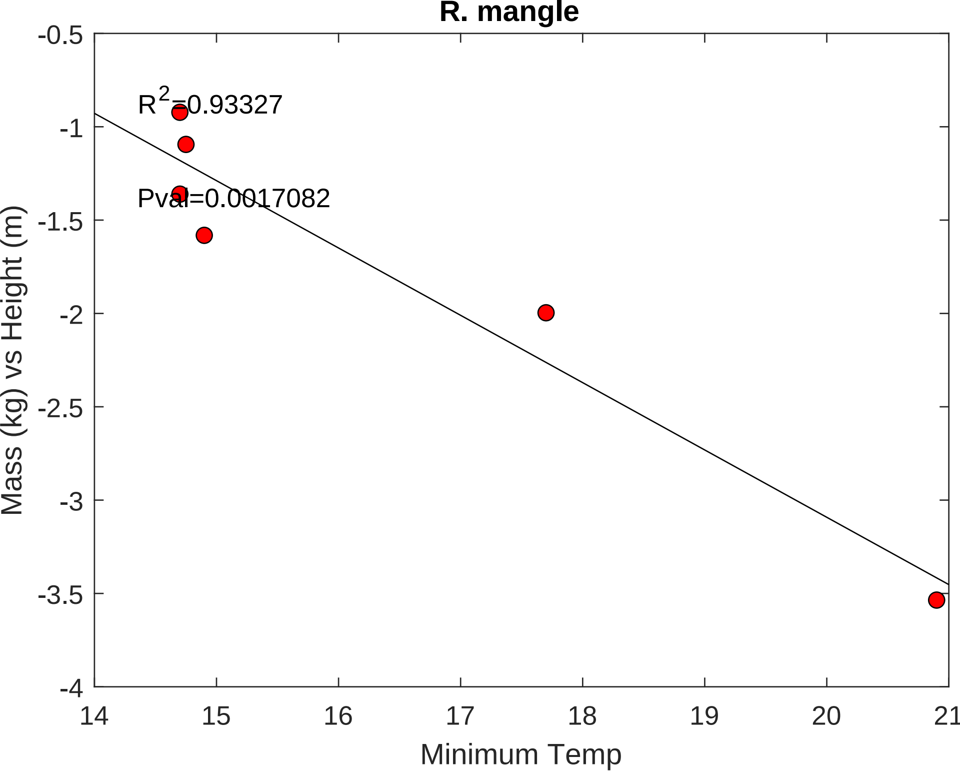

R. mangle
R2=0.93327
Pval=0.0017082
Mass (kg) vs Height (m)
Minimum Temp
-0.5
-1
-1.5
-2
-2.5
-3
-3.5
-4
14
15
16
17
18
19
20
21

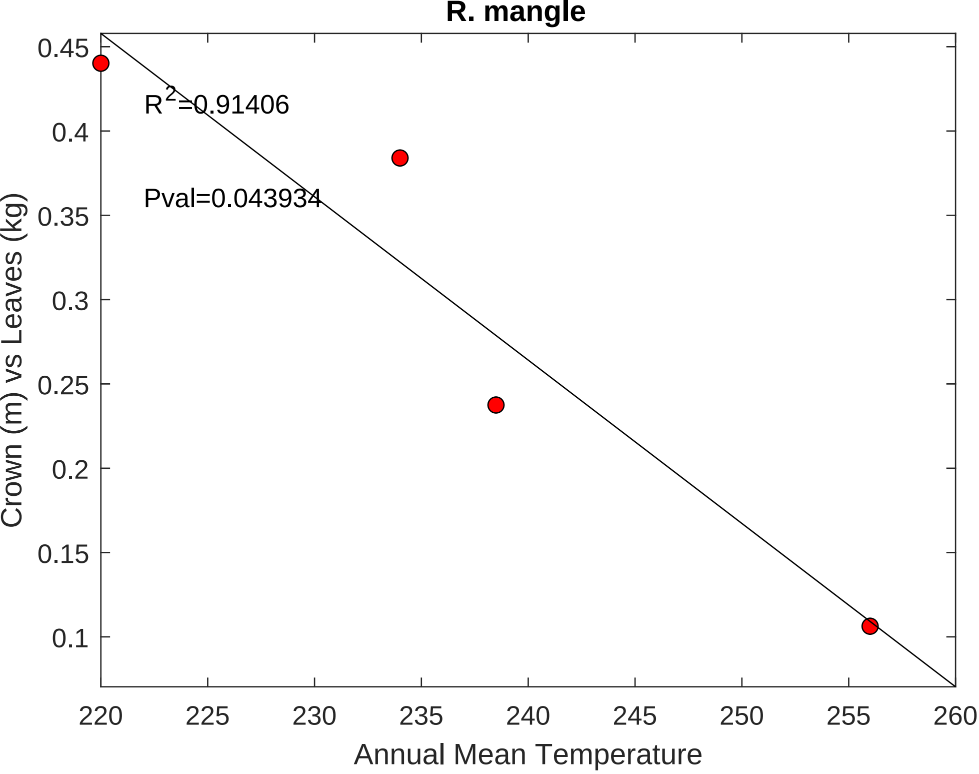

R. mangle
R²=0.91406
Pval=0.043934
Crown (m) vs Leaves (kg)
Annual Mean Temperature
0.45
0.4
0.35
0.3
0.25
0.2
0.15
0.1
220
225
230
235
240
245
250
255
260

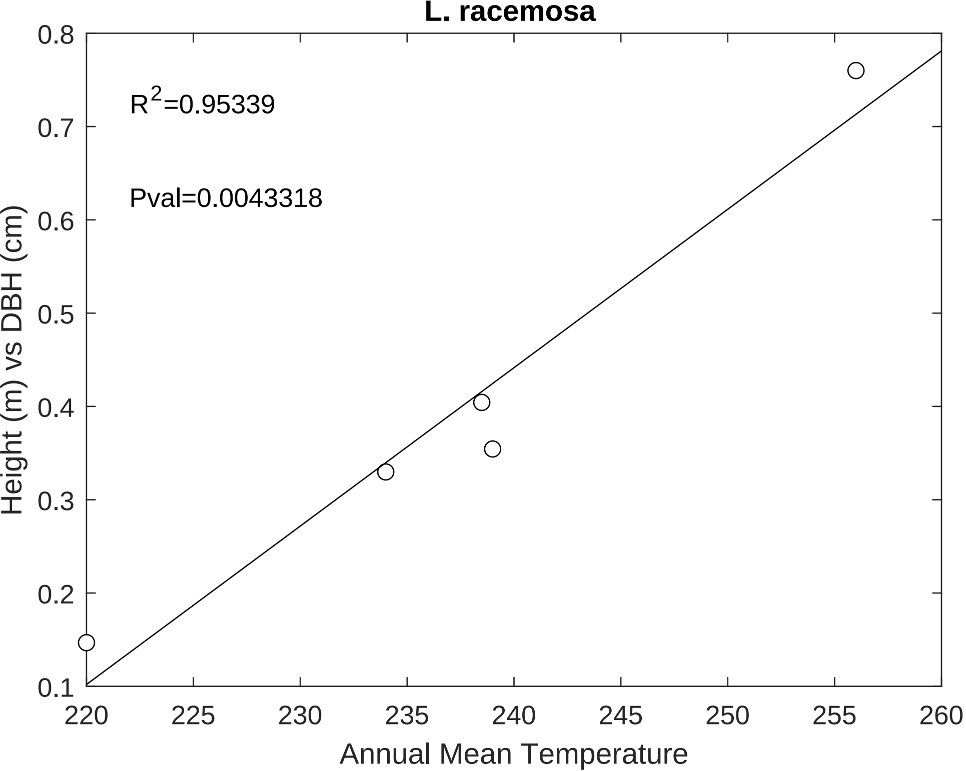
L. racemosa
R2=0.95339
Pval=0.0043318
Height (m) vs DBH (cm)
0.8
0.7
0.6
0.5
0.4
0.3
0.2
0.1
220
225
230
235
240
245
250
255
260
Annual Mean Temperature

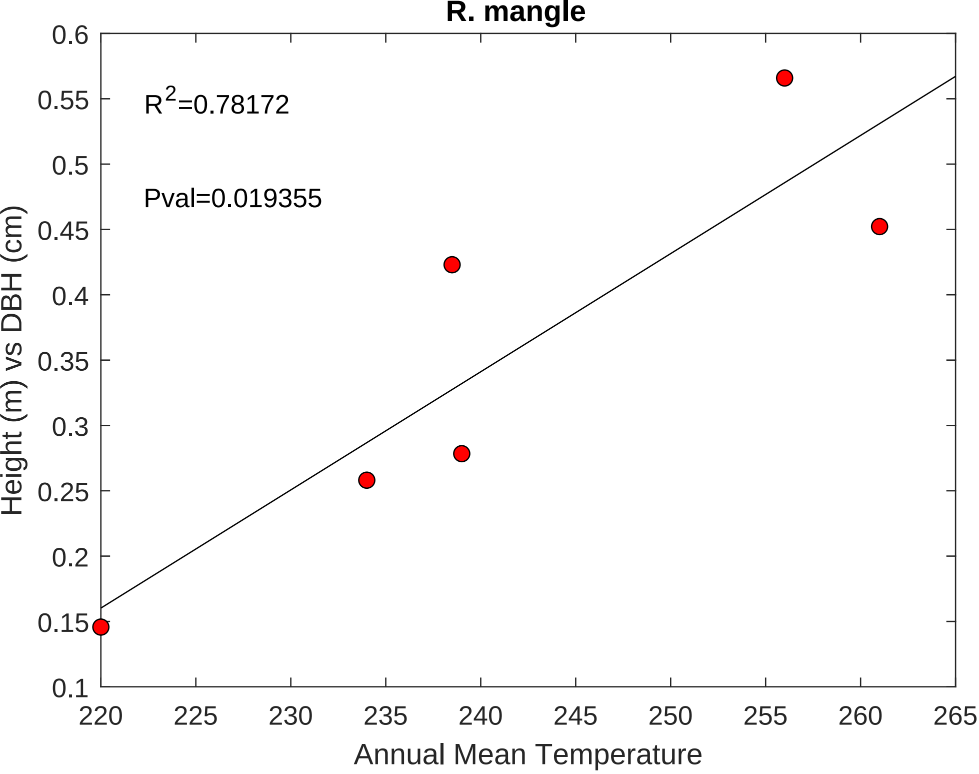

R. mangle
R^2=0.78172
Pval=0.019355
Height (m) vs DBH (cm)
Annual Mean Temperature
0.6
0.55
0.5
0.45
0.4
0.35
0.3
0.25
0.2
0.15
0.1
220
225
230
235
240
245
250
255
260
265

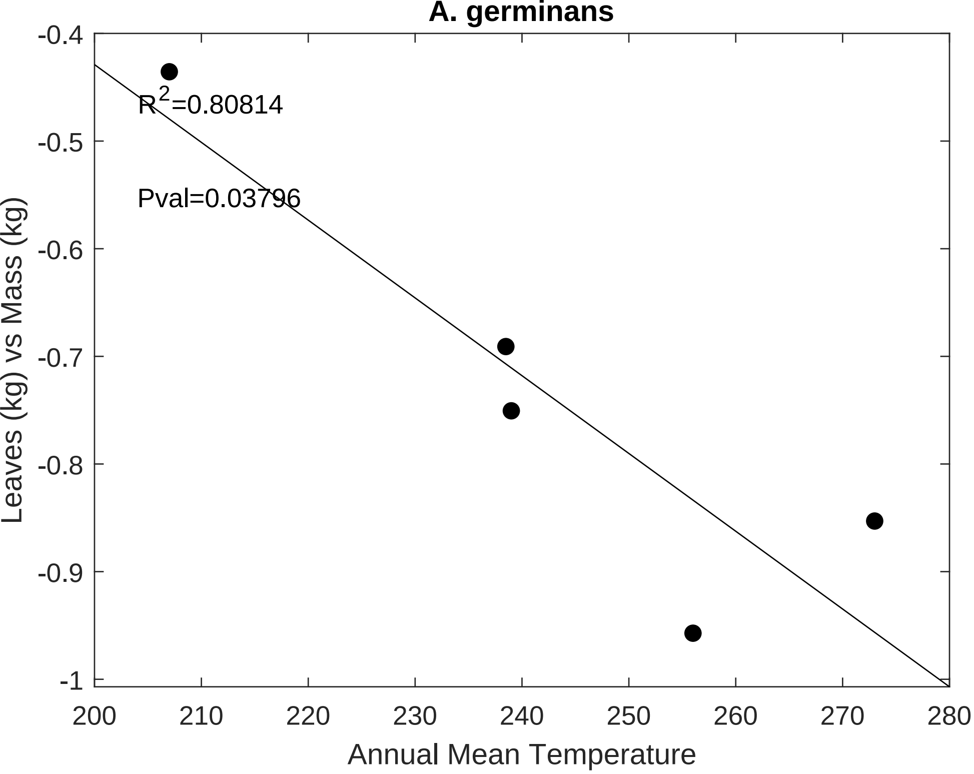

A. germinans
R2=0.80814
Pval=0.03796
-0.4
-0.5
-0.6
-0.7
-0.8
-0.9
-1
200
210
220
230
240
250
260
270
280
Leaves (kg) vs Mass (kg)
Annual Mean Temperature

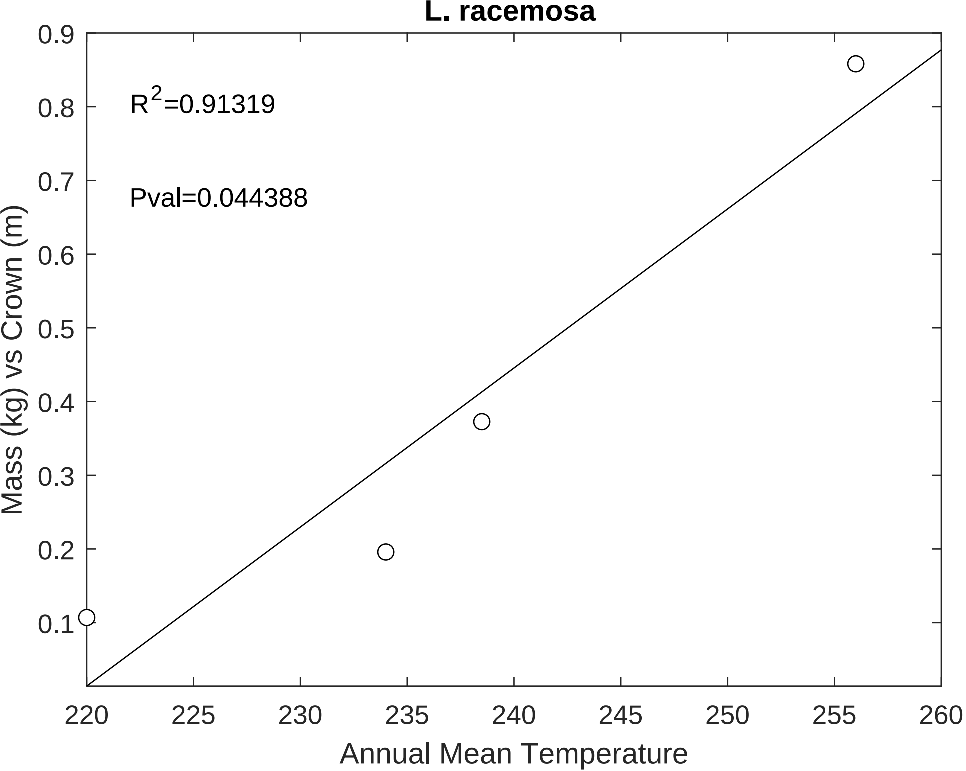

L. racemosa
R2=0.91319
Pval=0.044388
Mass (kg) vs Crown (m)
Annual Mean Temperature
0.9
0.8
0.7
0.6
0.5
0.4
0.3
0.2
0.1
220
225
230
235
240
245
250
255
260

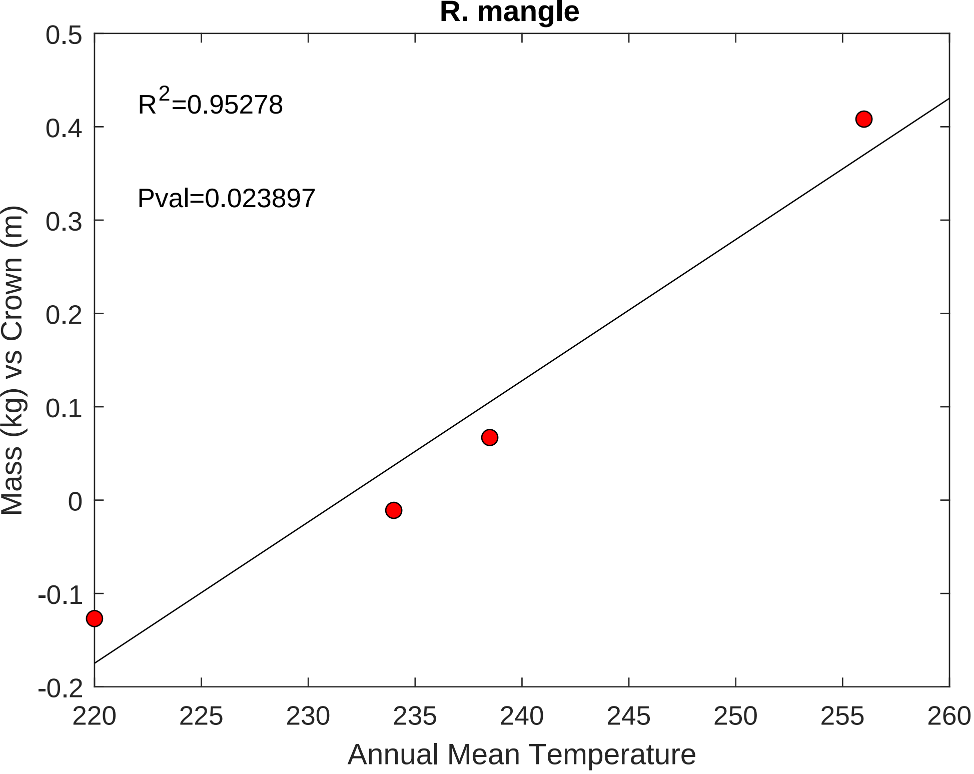

R. mangle
R²=0.95278
Pval=0.023897
Mass (kg) vs Crown (m)
Annual Mean Temperature
0.5
0.4
0.3
0.2
0.1
0
-0.1
-0.2
220
225
230
235
240
245
250
255
260

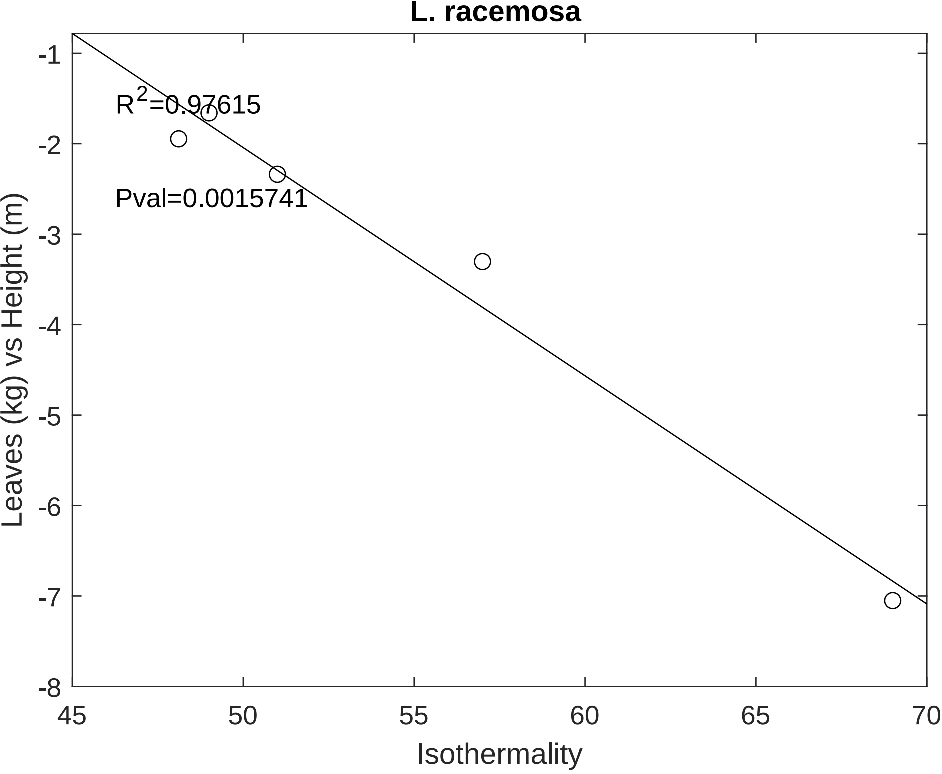
L. racemosa
R2=0.97615
Pval=0.0015741
Leaves (kg) vs Height (m)
-1
-2
-3
-4
-5
-6
-7
-8
45
50
55
60
65
70
Isothermality

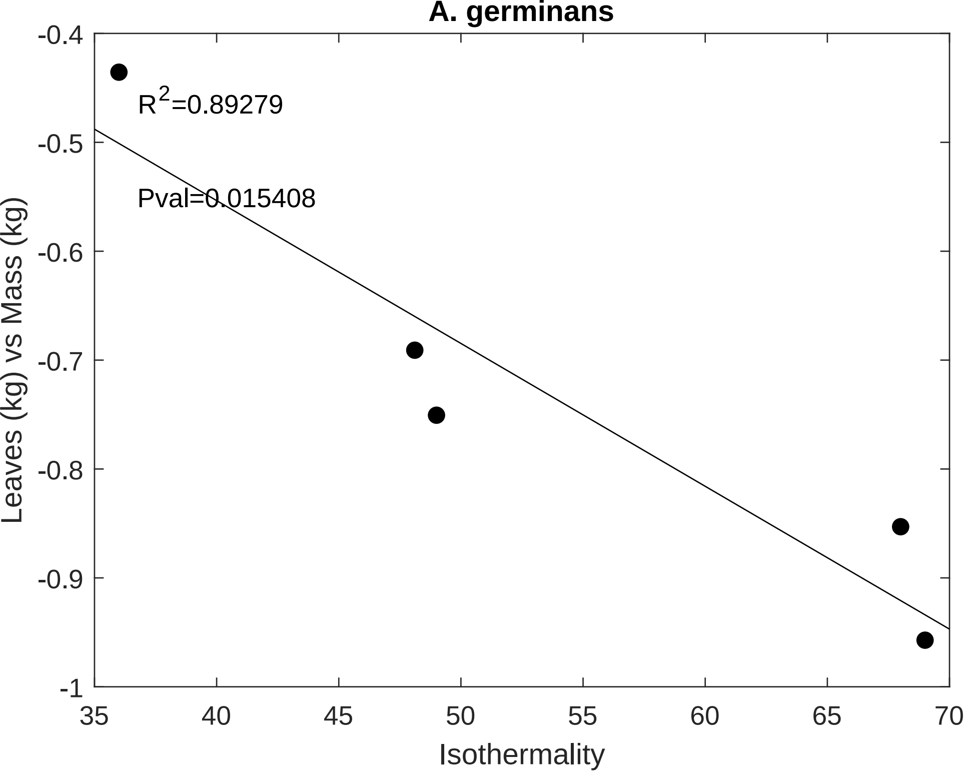

A. germinans
R^2=0.89279
Pval=0.015408
-0.4
-0.5
-0.6
-0.7
-0.8
-0.9
-1
35
40
45
50
55
60
65
70
Isothermality
Leaves (kg) vs Mass (kg)

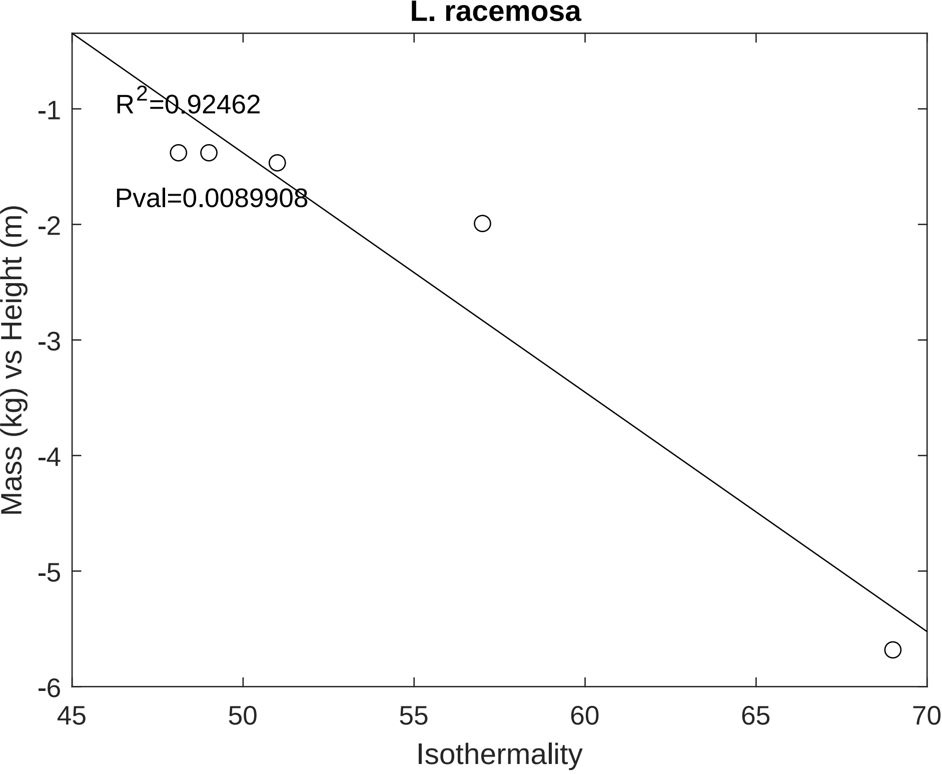
L. racemosa
R^2=0.92462
Pval=0.0089908
Mass (kg) vs Height (m)
Isothermality
-1
-2
-3
-4
-5
-6
45
50
55
60
65
70

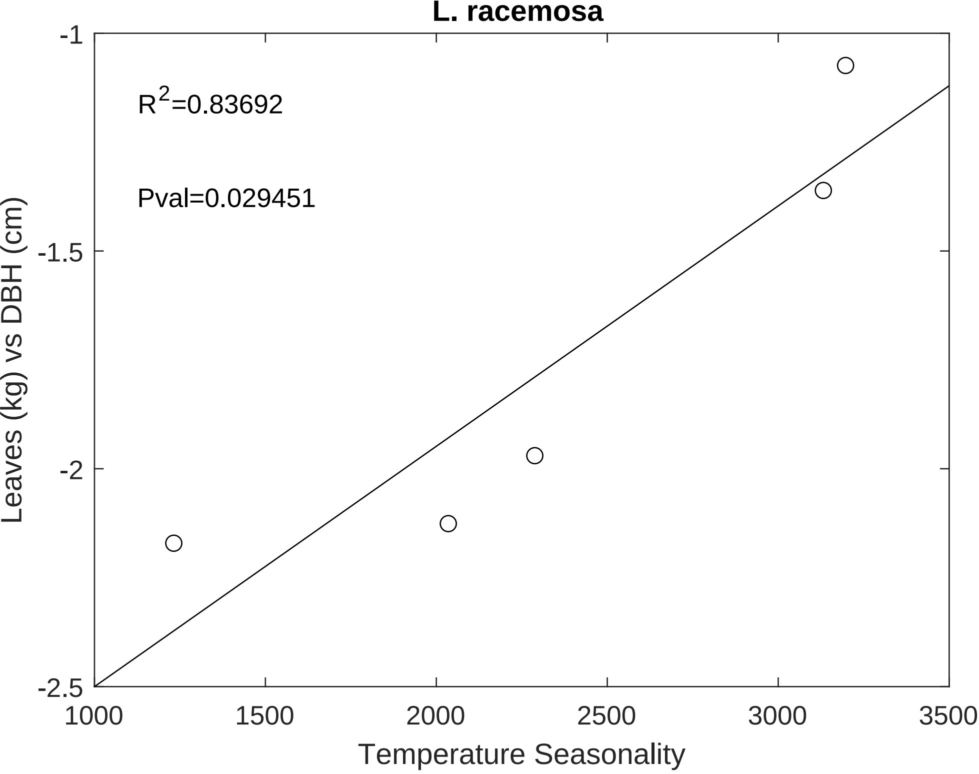
L. racemosa
R2=0.83692
Pval=0.029451
-1
-1.5
-2
-2.5
Leaves (kg) vs DBH (cm)
1000
1500
2000
2500
3000
3500
Temperature Seasonality

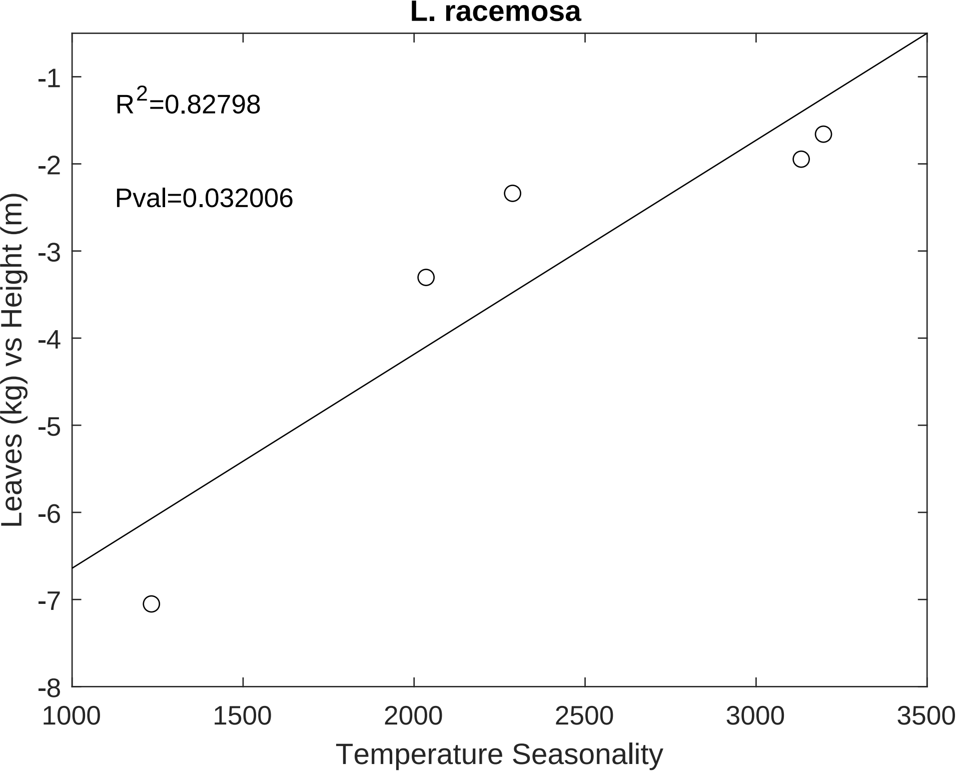

L. racemosa
R^2=0.82798
Pval=0.032006
Leaves (kg) vs Height (m)
-1
-2
-3
-4
-5
-6
-7
-8
1000
1500
2000
2500
3000
3500
Temperature Seasonality

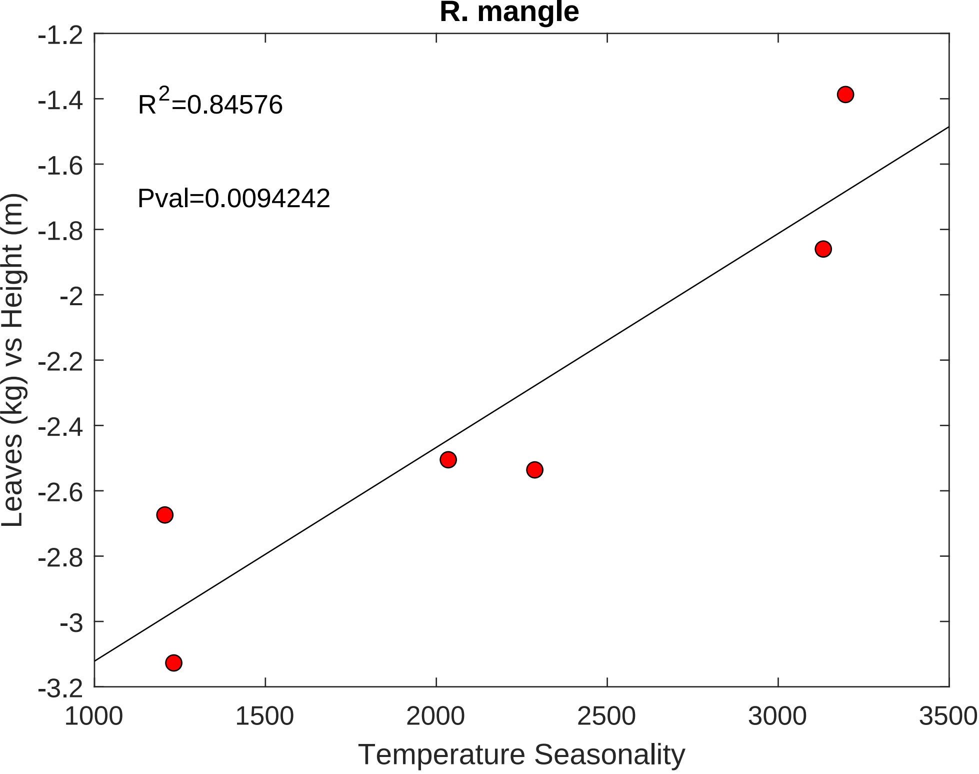
R. mangle
R^2=0.84576
Pval=0.0094242
Leaves (kg) vs Height (m)
Temperature Seasonality
-1.2
-1.4
-1.6
-1.8
-2
-2.2
-2.4
-2.6
-2.8
-3
-3.2
1000
1500
2000
2500
3000
3500

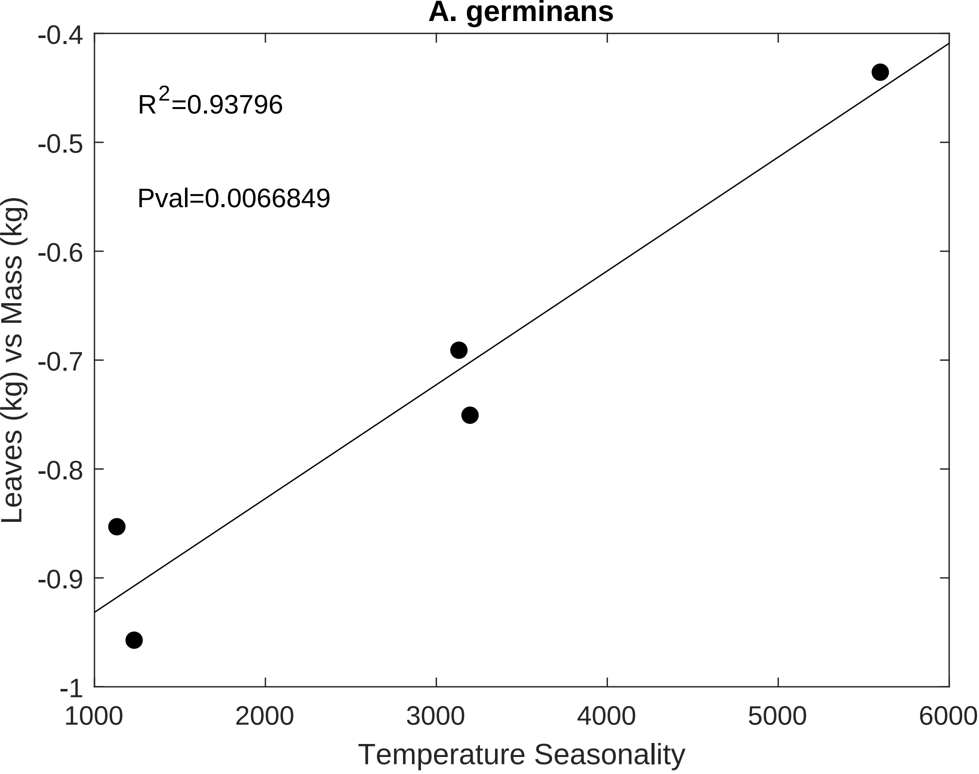
A. germinans
R^2=0.93796
Pval=0.0066849
Leaves (kg) vs Mass (kg)
Temperature Seasonality
-0.4
-0.5
-0.6
-0.7
-0.8
-0.9
-1
1000
2000
3000
4000
5000
6000

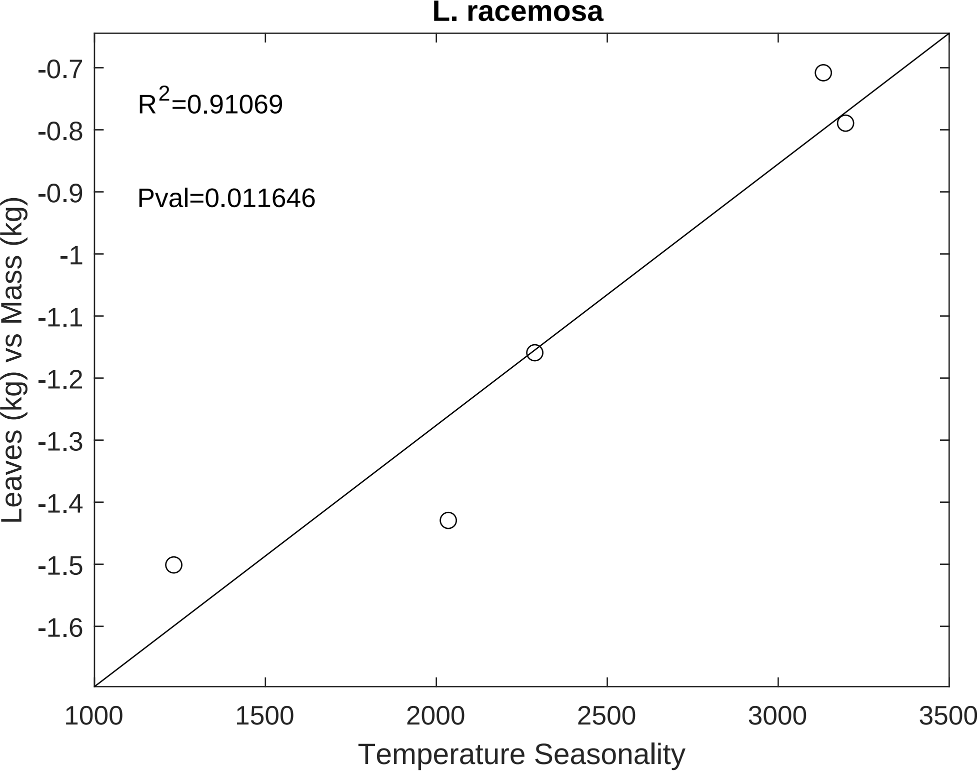

L. racemosa
R2=0.91069
Pval=0.011646
Leaves (kg) vs Mass (kg)
Temperature Seasonality
-0.7
-0.8
-0.9
-1
-1.1
-1.2
-1.3
-1.4
-1.5
-1.6
1000
1500
2000
2500
3000
3500

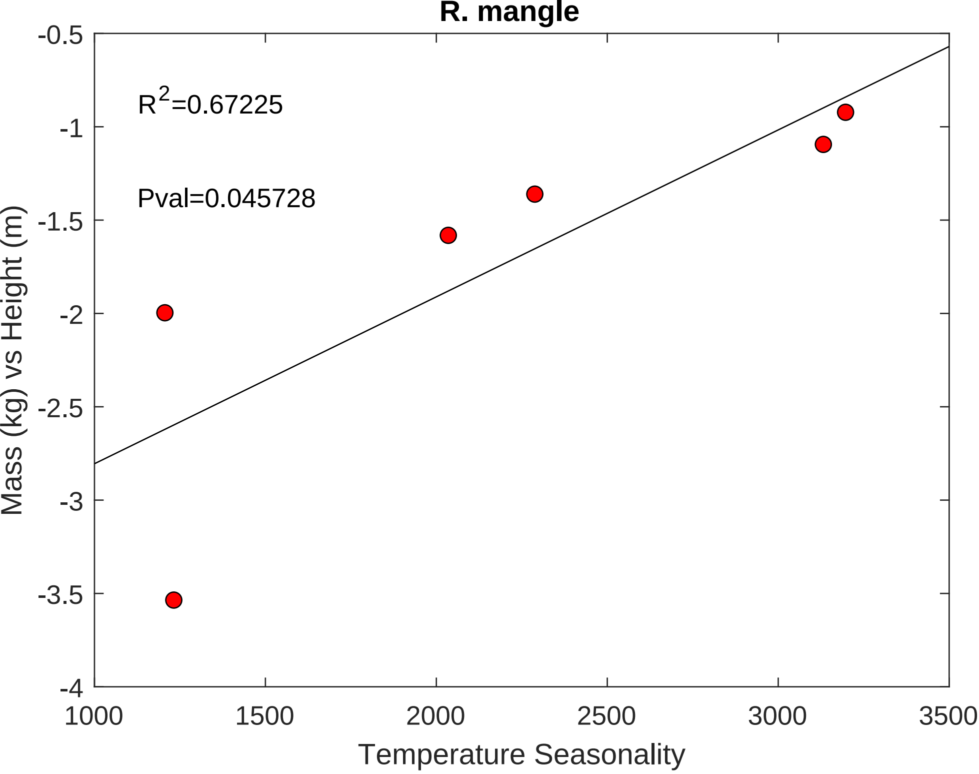

R. mangle
R2=0.67225
Pval=0.045728
Mass (kg) vs Height (m)
Temperature Seasonality
-0.5
-1
-1.5
-2
-2.5
-3
-3.5
-4
1000
1500
2000
2500
3000
3500

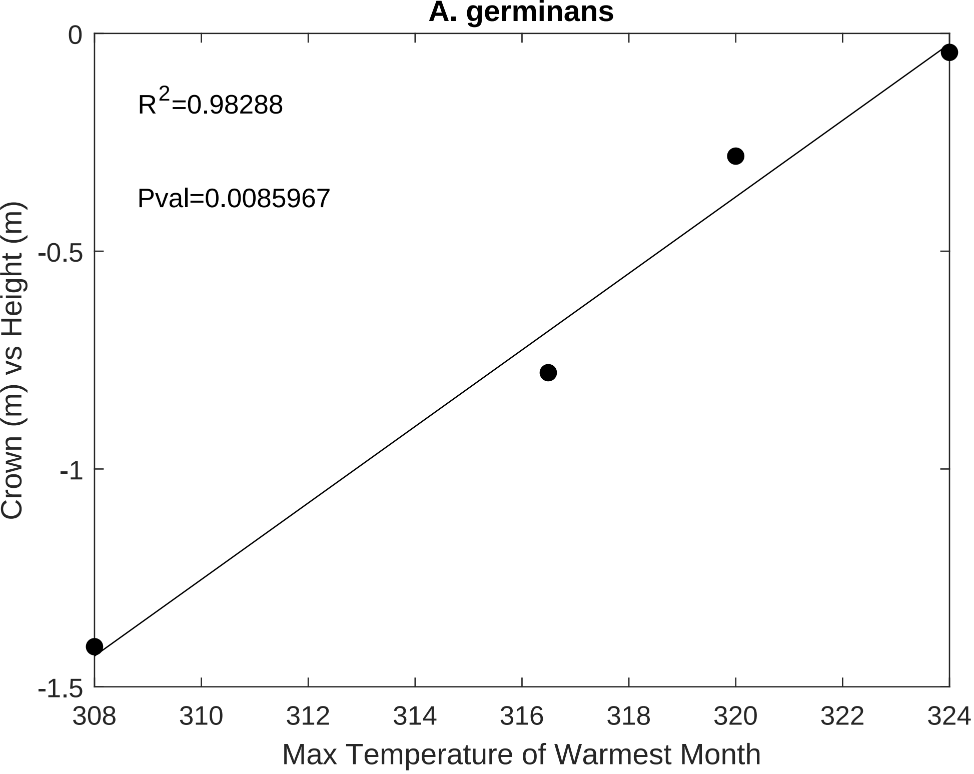
A. germinans
R2=0.98288
Pval=0.0085967
0
-0.5
-1
-1.5
308
310
312
314
316
318
320
322
324
Max Temperature of Warmest Month
Crown (m) vs Height (m)

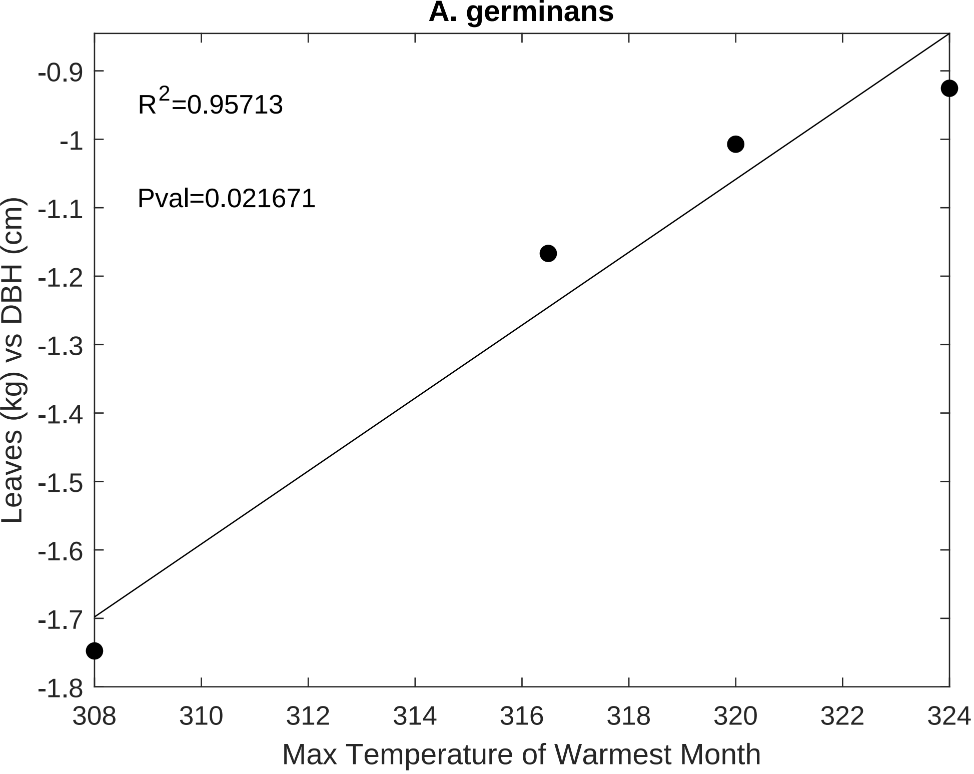
A. germinans
R2=0.95713
Pval=0.021671
Leaves (kg) vs DBH (cm)
-0.9
-1
-1.1
-1.2
-1.3
-1.4
-1.5
-1.6
-1.7
-1.8
308
310
312
314
316
318
320
322
324
Max Temperature of Warmest Month

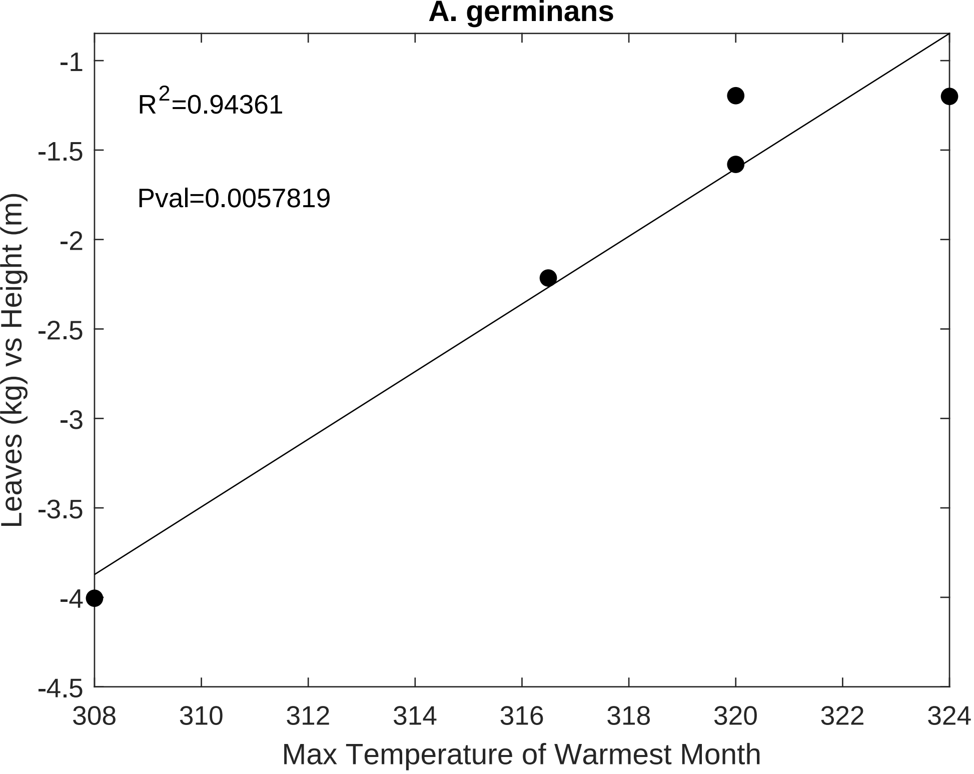
A. germinans
R^2=0.94361
Pval=0.0057819
Leaves (kg) vs Height (m)
-1
-1.5
-2
-2.5
-3
-3.5
-4
-4.5
308
310
312
314
316
318
320
322
324
Max Temperature of Warmest Month

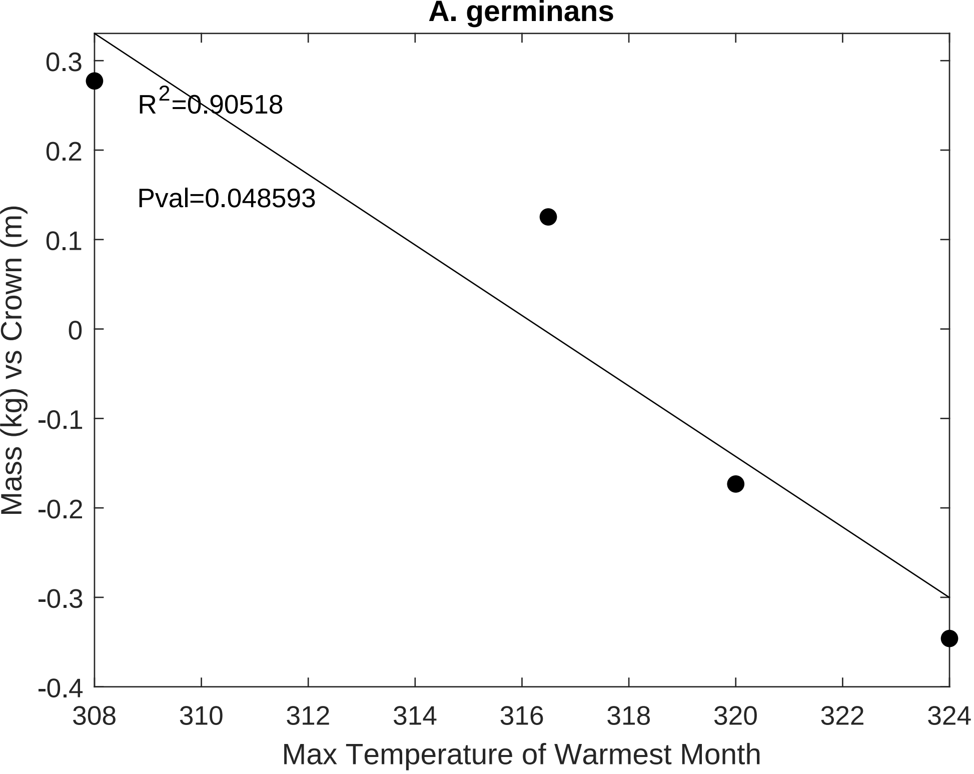
A. germinans
R^2=0.90518
Pval=0.048593
Mass (kg) vs Crown (m)
0.3
0.2
0.1
0
-0.1
-0.2
-0.3
-0.4
308
310
312
314
316
318
320
322
324
Max Temperature of Warmest Month

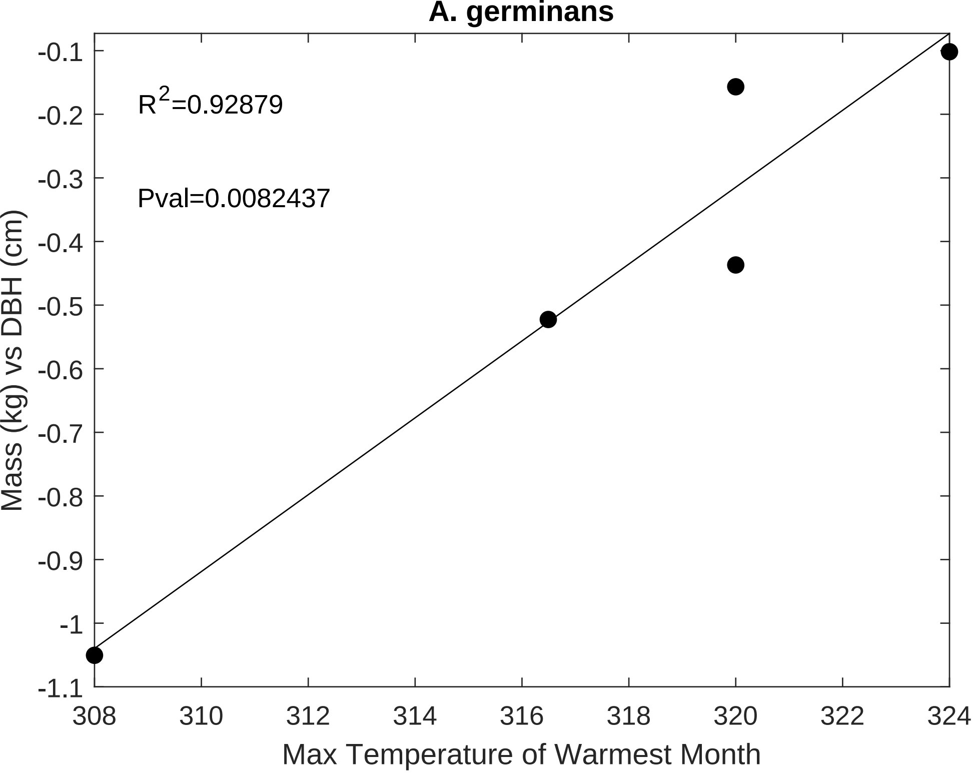
A. germinans
R2=0.92879
Pval=0.0082437
-0.1
-0.2
-0.3
-0.4
-0.5
-0.6
-0.7
-0.8
-0.9
-1
-1.1
308
310
312
314
316
318
320
322
324
Mass (kg) vs DBH (cm)
Max Temperature of Warmest Month

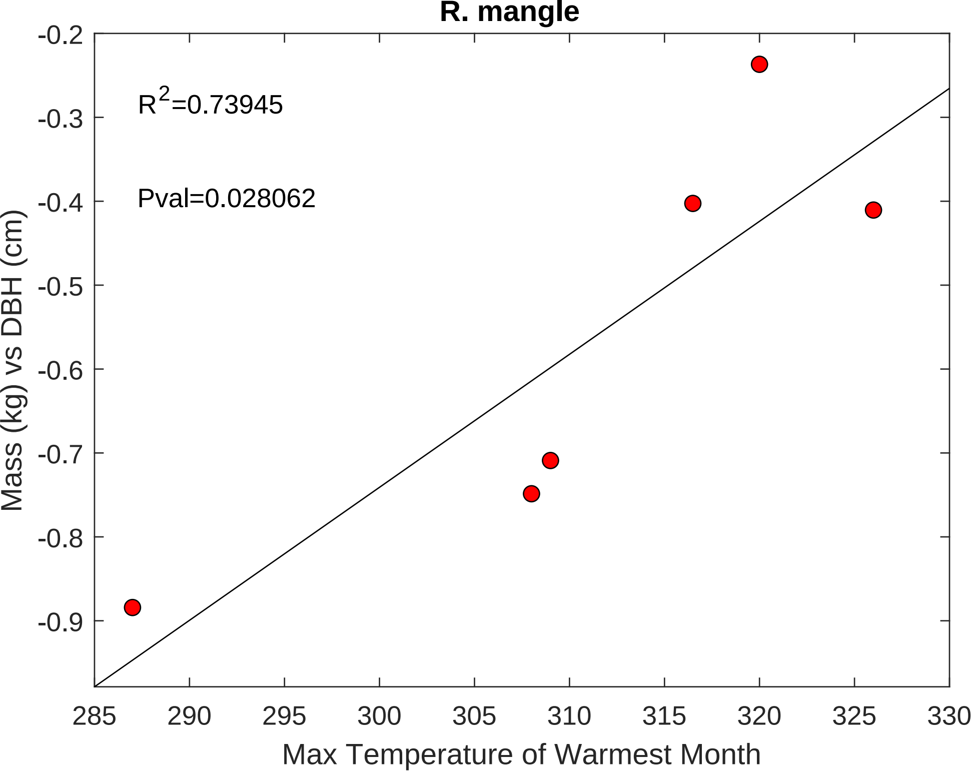
R. mangle
R^2=0.73945
Pval=0.028062
Mass (kg) vs DBH (cm)
Max Temperature of Warmest Month
-0.2
-0.3
-0.4
-0.5
-0.6
-0.7
-0.8
-0.9
285
290
295
300
305
310
315
320
325
330

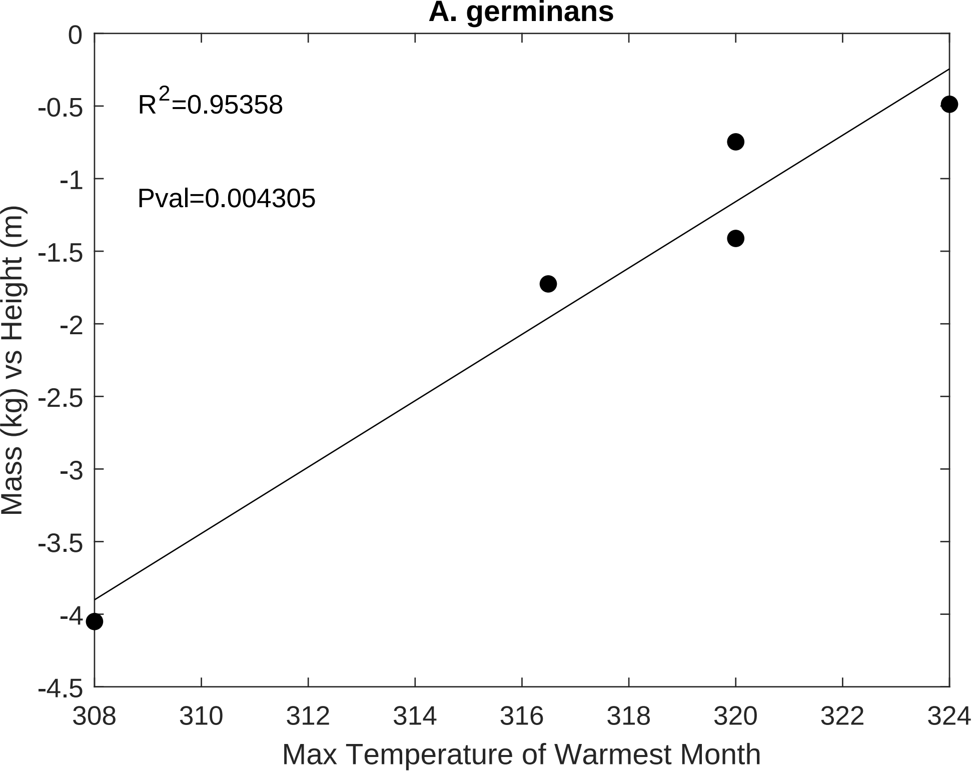
A. germinans
R2=0.95358
Pval=0.004305
Mass (kg) vs Height (m)
Max Temperature of Warmest Month
0
-0.5
-1
-1.5
-2
-2.5
-3
-3.5
-4
-4.5
308
310
312
314
316
318
320
322
324

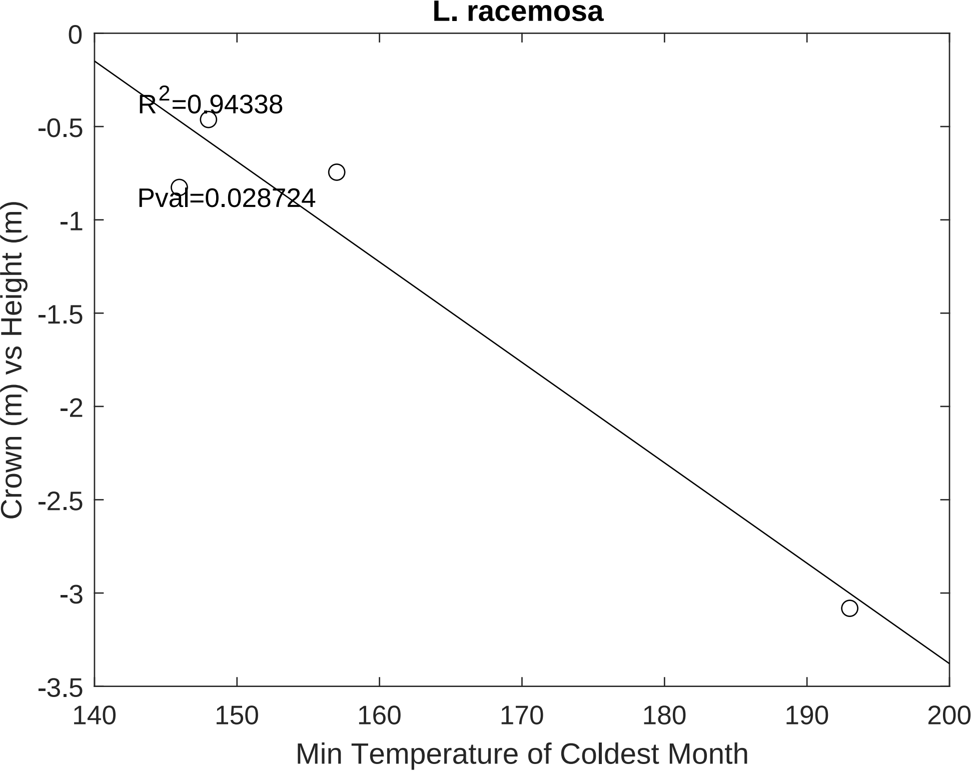

L. racemosa
R2=0.94338
Pval=0.028724
Crown (m) vs Height (m)
Min Temperature of Coldest Month
0
-0.5
-1
-1.5
-2
-2.5
-3
-3.5
140
150
160
170
180
190
200

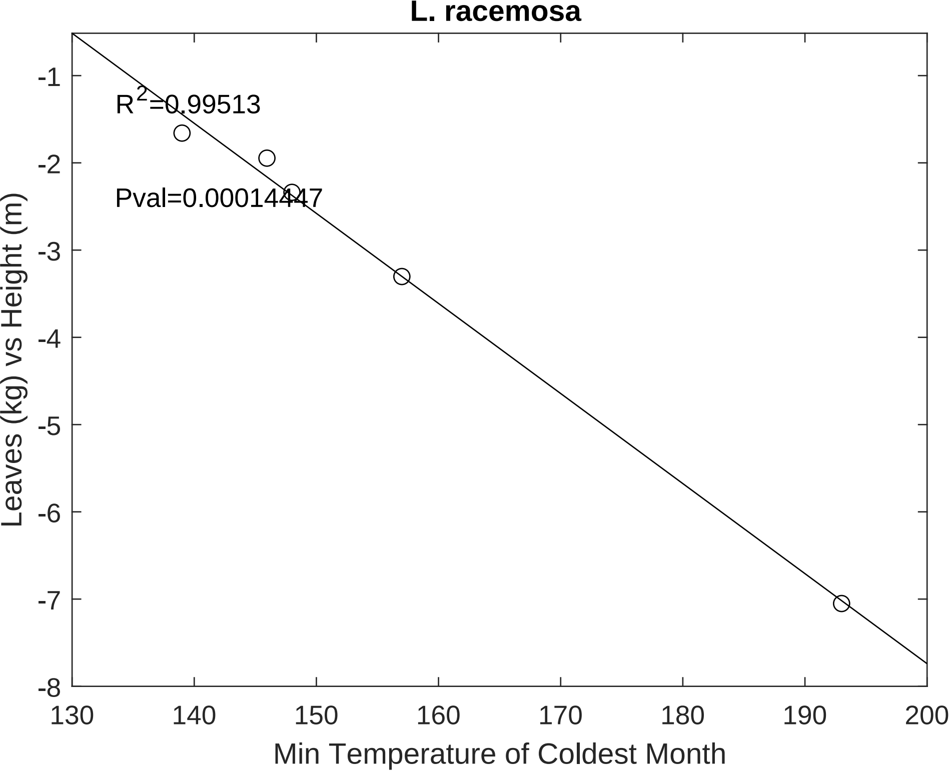

L. racemosa
R2=0.99513
Pval=0.00014447
-1
-2
-3
-4
-5
-6
-7
-8
Leaves (kg) vs Height (m)
130
140
150
160
170
180
190
200
Min Temperature of Coldest Month

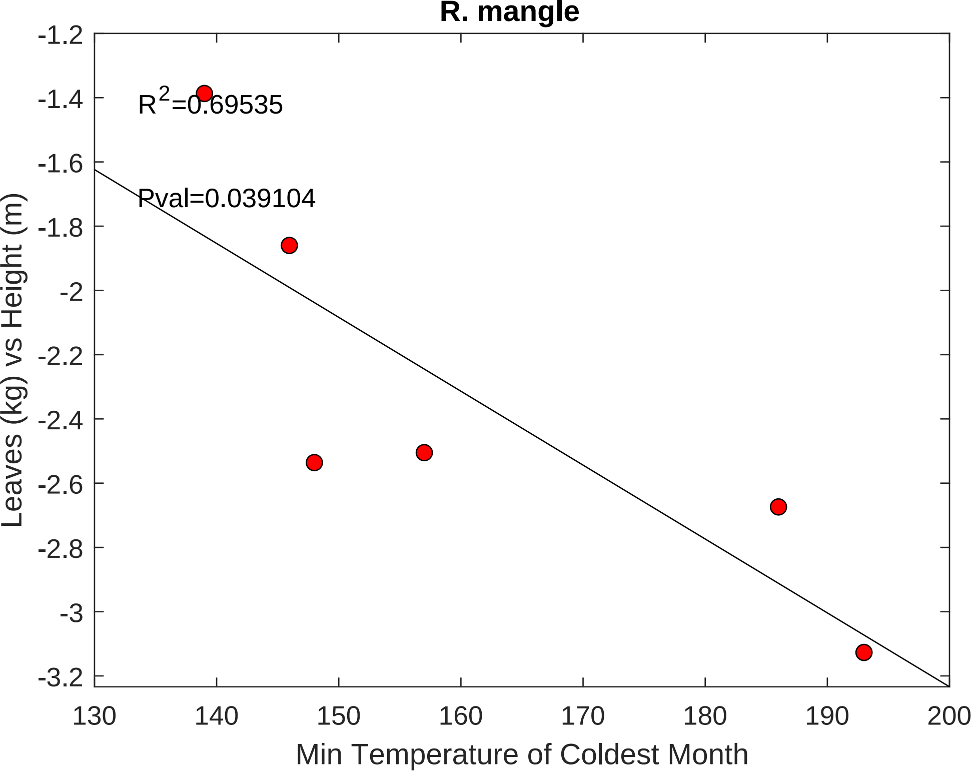
R. mangle
R^2=0.69535
Pval=0.039104
Leaves (kg) vs Height (m)
Min Temperature of Coldest Month
-1.2
-1.4
-1.6
-1.8
-2
-2.2
-2.4
-2.6
-2.8
-3
-3.2
130
140
150
160
170
180
190
200

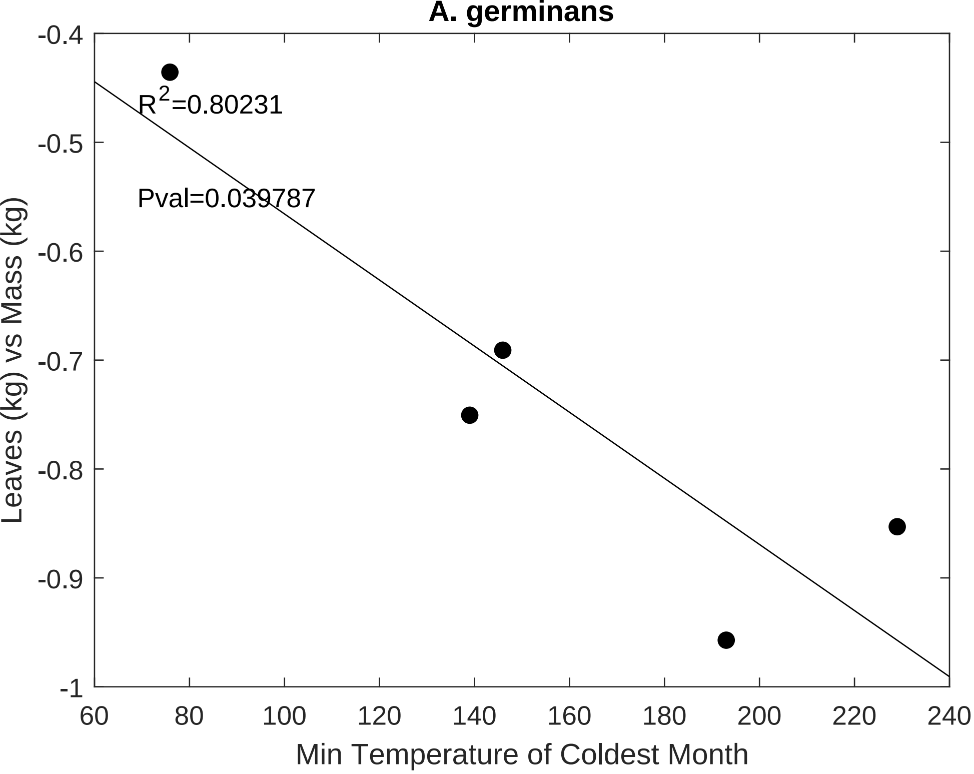

A. germinans
R^2=0.80231
Pval=0.039787
Leaves (kg) vs Mass (kg)
-0.4
-0.5
-0.6
-0.7
-0.8
-0.9
-1
60
80
100
120
140
160
180
200
220
240
Min Temperature of Coldest Month

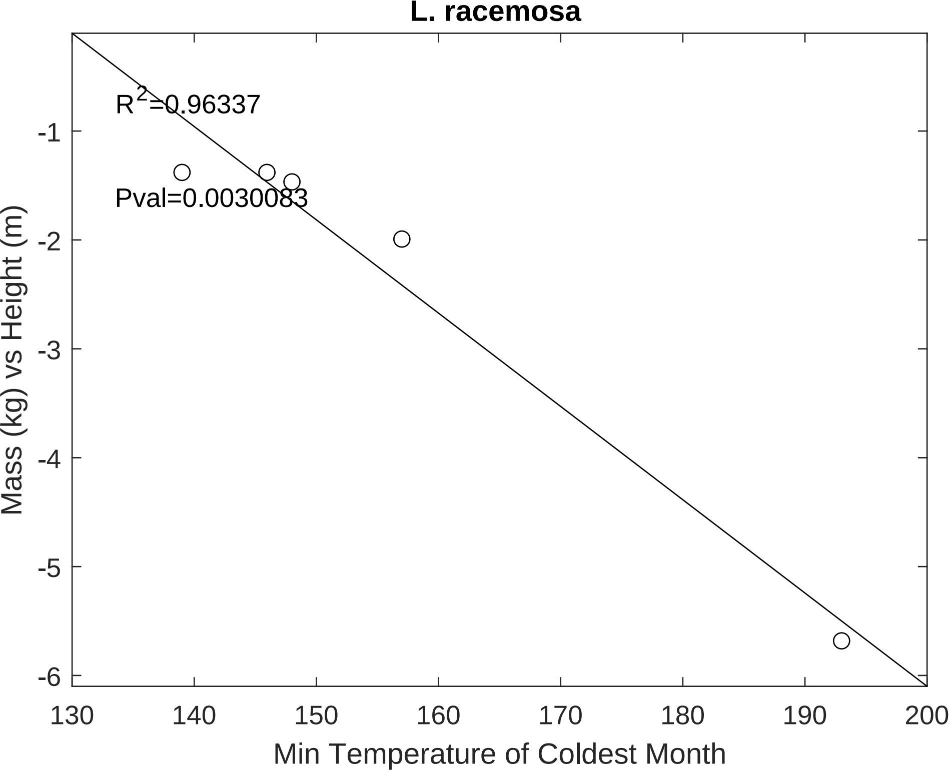

L. racemosa
R^2=0.96337
Pval=0.0030083
Mass (kg) vs Height (m)
-1
-2
-3
-4
-5
-6
130
140
150
160
170
180
190
200
Min Temperature of Coldest Month

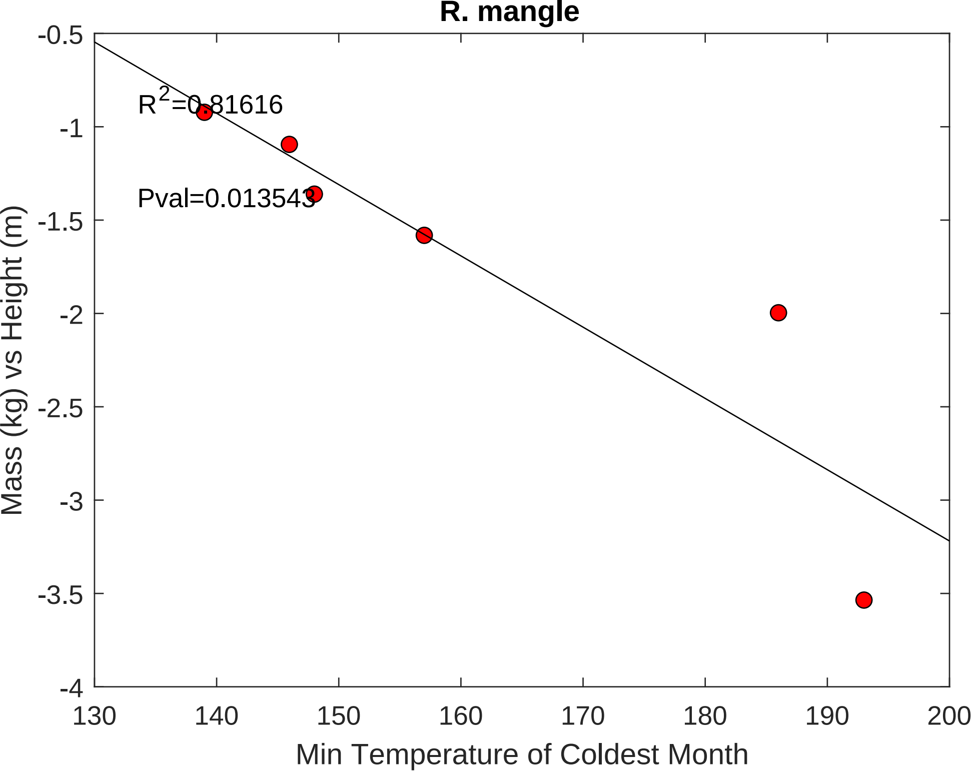
R. mangle
R^2=0.81616
Pval=0.013543
Mass (kg) vs Height (m)
Min Temperature of Coldest Month
-0.5
-1
-1.5
-2
-2.5
-3
-3.5
-4
130
140
150
160
170
180
190
200

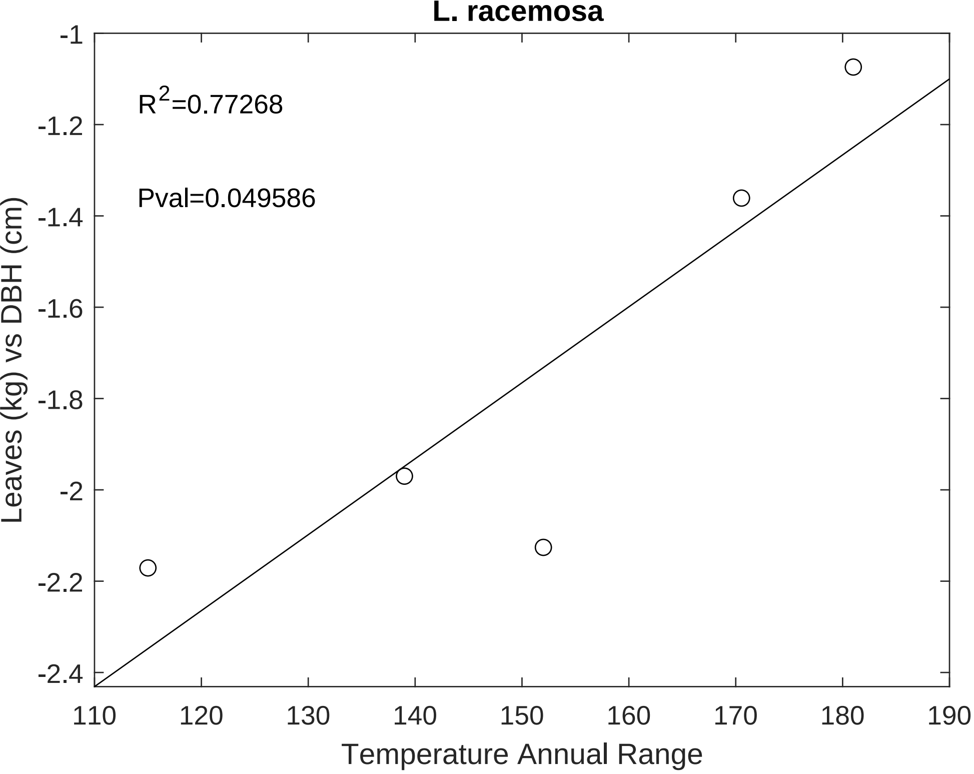

L. racemosa
R2=0.77268
Pval=0.049586
Leaves (kg) vs DBH (cm)
Temperature Annual Range
-1
-1.2
-1.4
-1.6
-1.8
-2
-2.2
-2.4
110
120
130
140
150
160
170
180
190

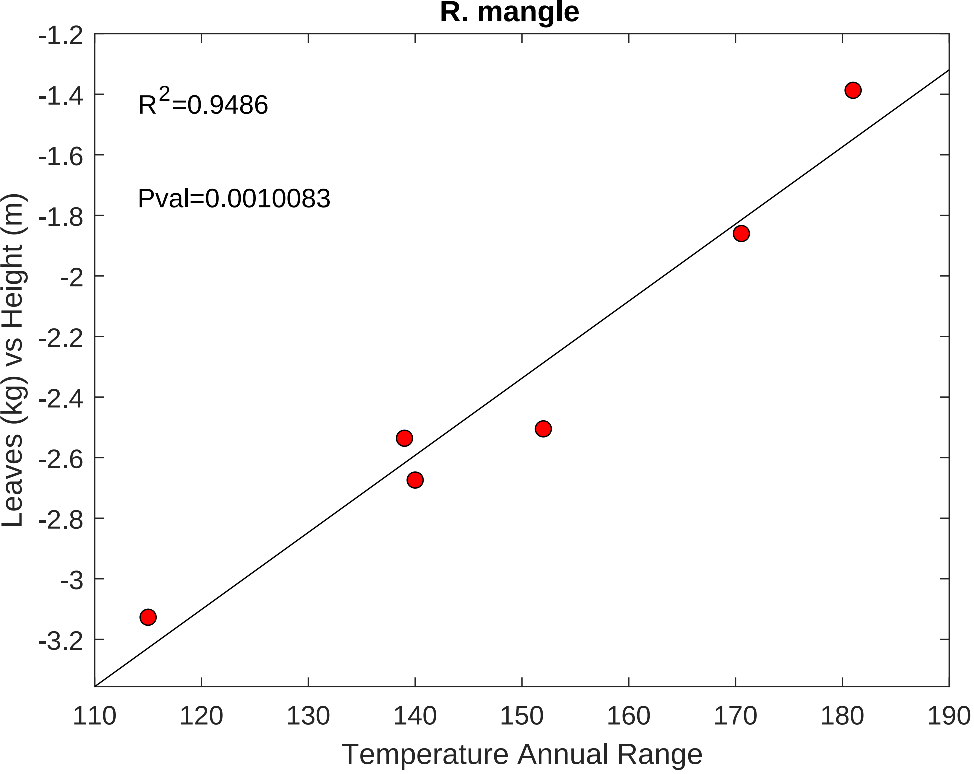

R. mangle
R2=0.9486
Pval=0.0010083
Leaves (kg) vs Height (m)
Temperature Annual Range
-1.2
-1.4
-1.6
-1.8
-2
-2.2
-2.4
-2.6
-2.8
-3
-3.2
110
120
130
140
150
160
170
180
190

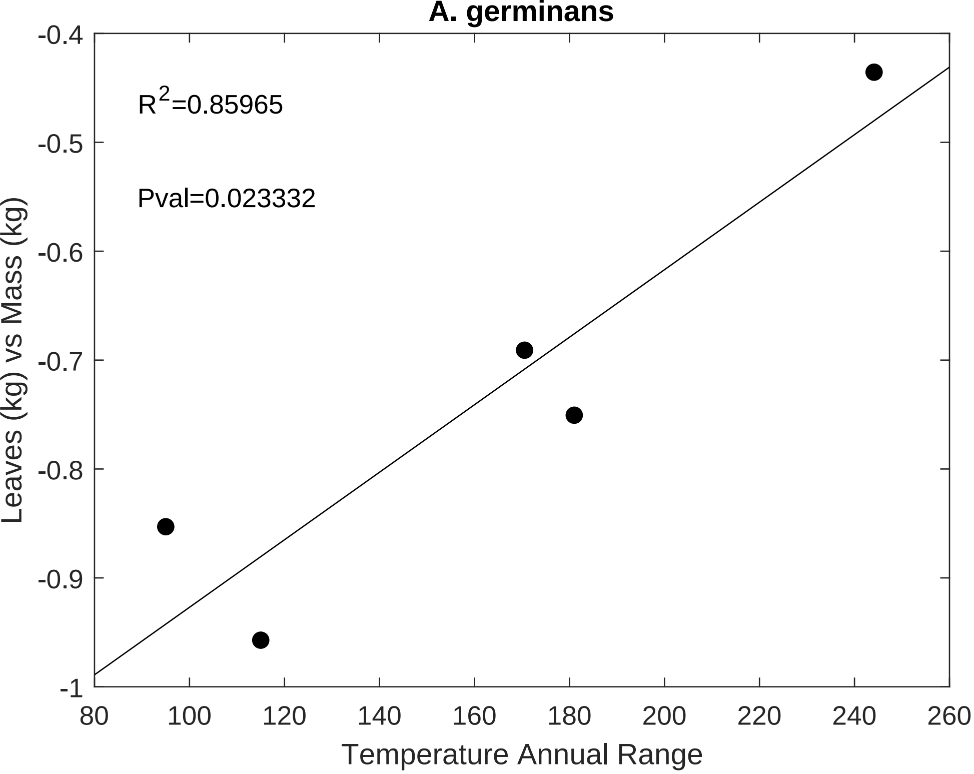
A. germinans
R2=0.85965
Pval=0.023332
Leaves (kg) vs Mass (kg)
Temperature Annual Range
-0.4
-0.5
-0.6
-0.7
-0.8
-0.9
-1
80
100
120
140
160
180
200
220
240
260

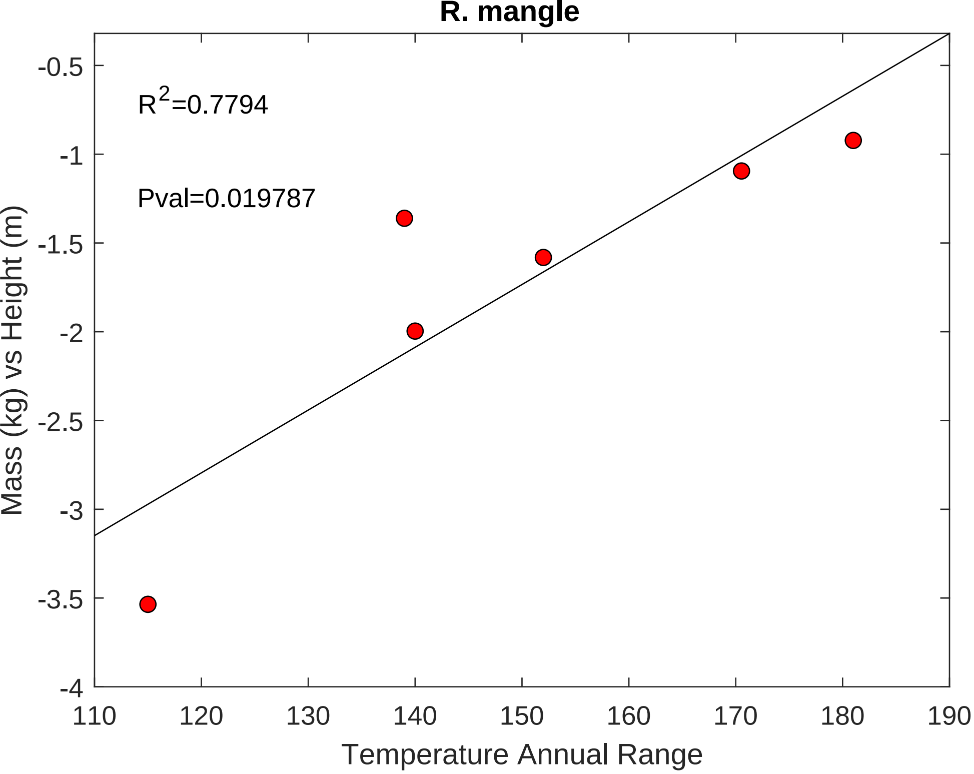
R. mangle
R^2=0.7794
Pval=0.019787
-0.5
-1
-1.5
-2
-2.5
-3
-3.5
-4
110
120
130
140
150
160
170
180
190
Mass (kg) vs Height (m)
Temperature Annual Range

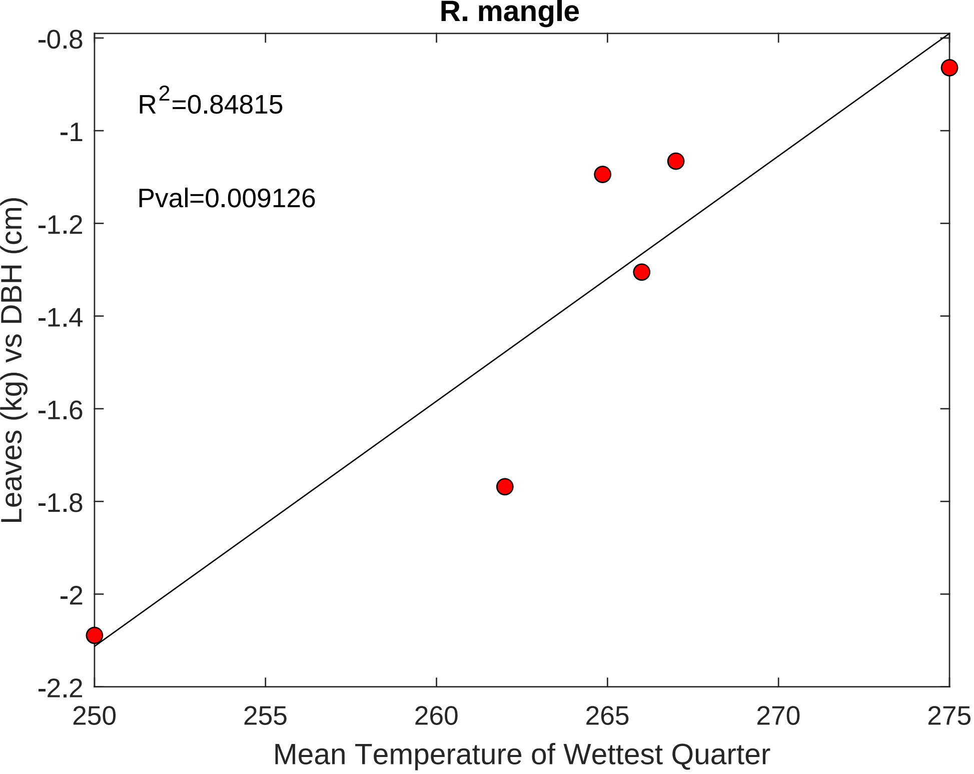
R. mangle
R2=0.84815
Pval=0.009126
Leaves (kg) vs DBH (cm)
Mean Temperature of Wettest Quarter
-0.8
-1
-1.2
-1.4
-1.6
-1.8
-2
-2.2
250
255
260
265
270
275

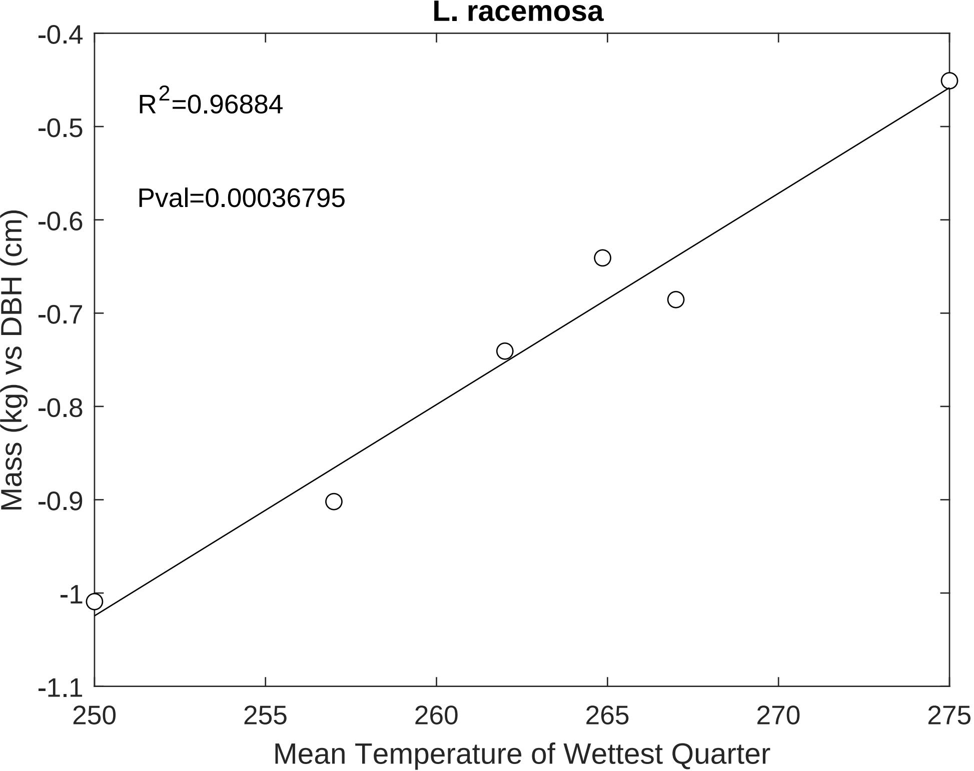

L. racemosa
R2=0.96884
Pval=0.00036795
-0.4
-0.5
-0.6
-0.7
-0.8
-0.9
-1
-1.1
250
255
260
265
270
275
Mass (kg) vs DBH (cm)
Mean Temperature of Wettest Quarter

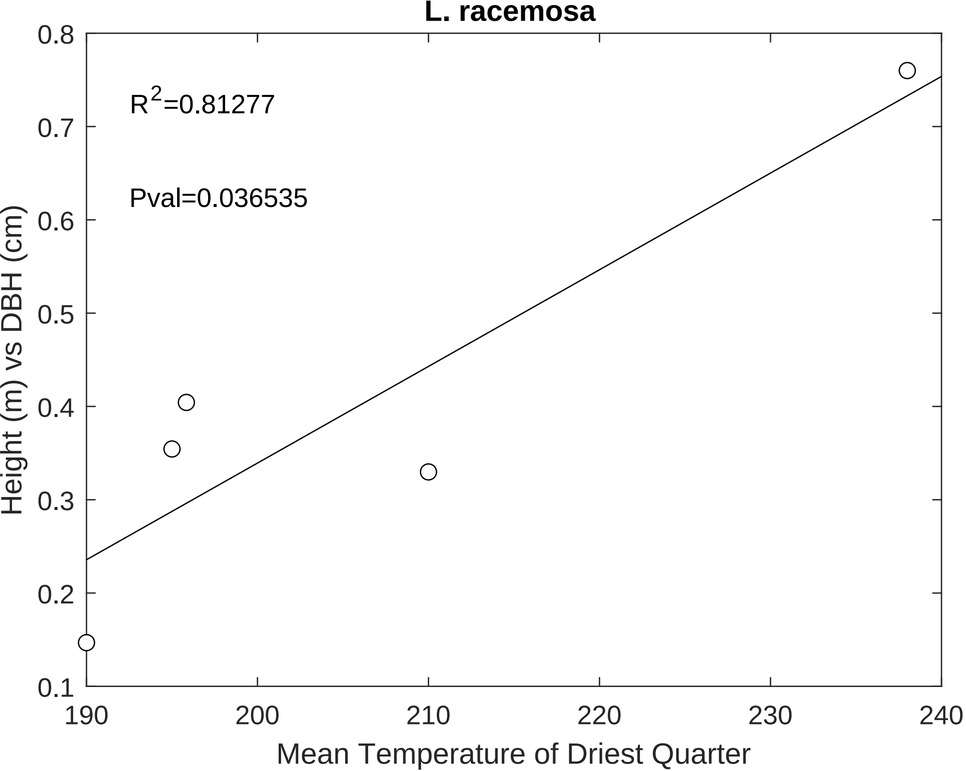
L. racemosa
R^2=0.81277
Pval=0.036535
Height (m) vs DBH (cm)
Mean Temperature of Driest Quarter
0.8
0.7
0.6
0.5
0.4
0.3
0.2
0.1
190
200
210
220
230
240

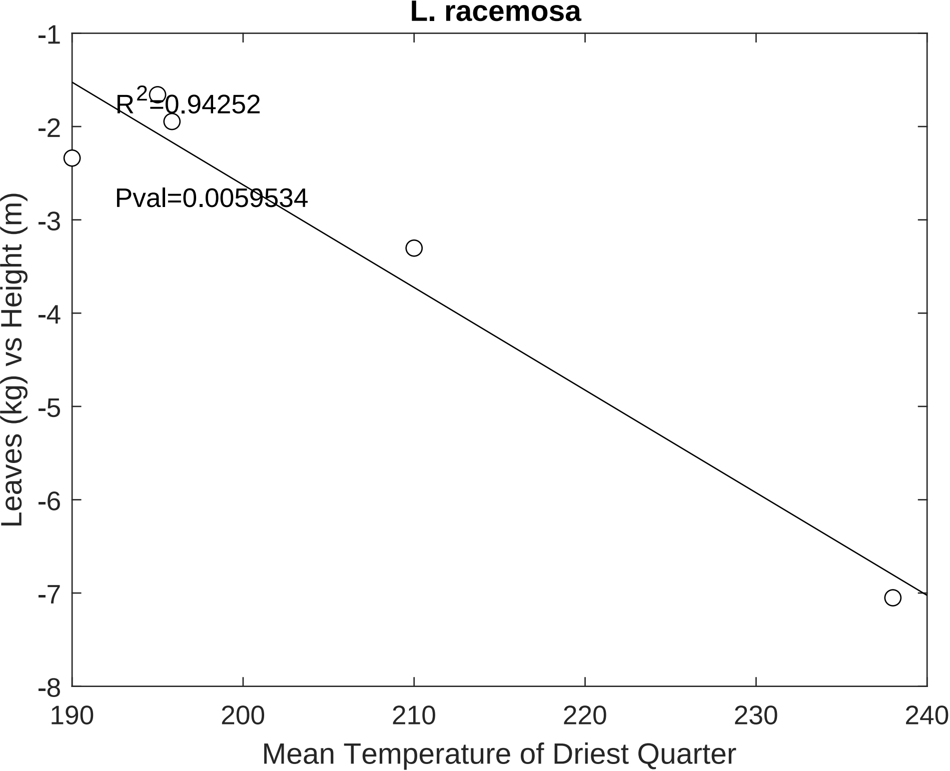
L. racemosa
R^2=0.94252
Pval=0.0059534
Leaves (kg) vs Height (m)
-1
-2
-3
-4
-5
-6
-7
-8
190
200
210
220
230
240
Mean Temperature of Driest Quarter

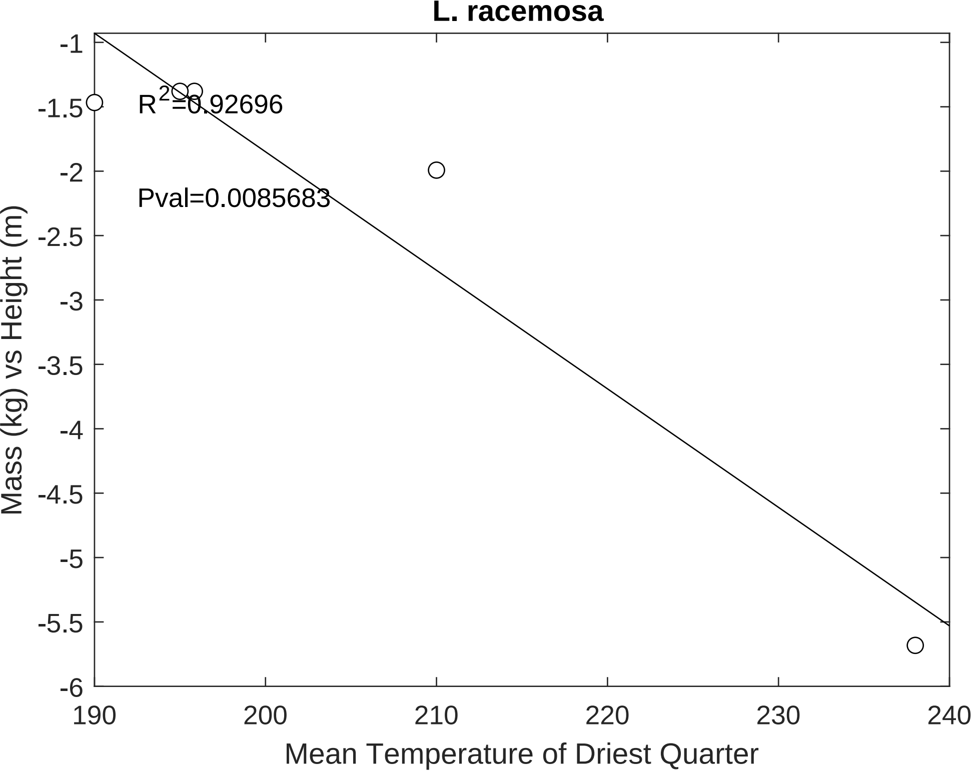
L. racemosa
R^2=0.92696
Pval=0.0085683
Mass (kg) vs Height (m)
-1
-1.5
-2
-2.5
-3
-3.5
-4
-4.5
-5
-5.5
-6
190
200
210
220
230
240
Mean Temperature of Driest Quarter

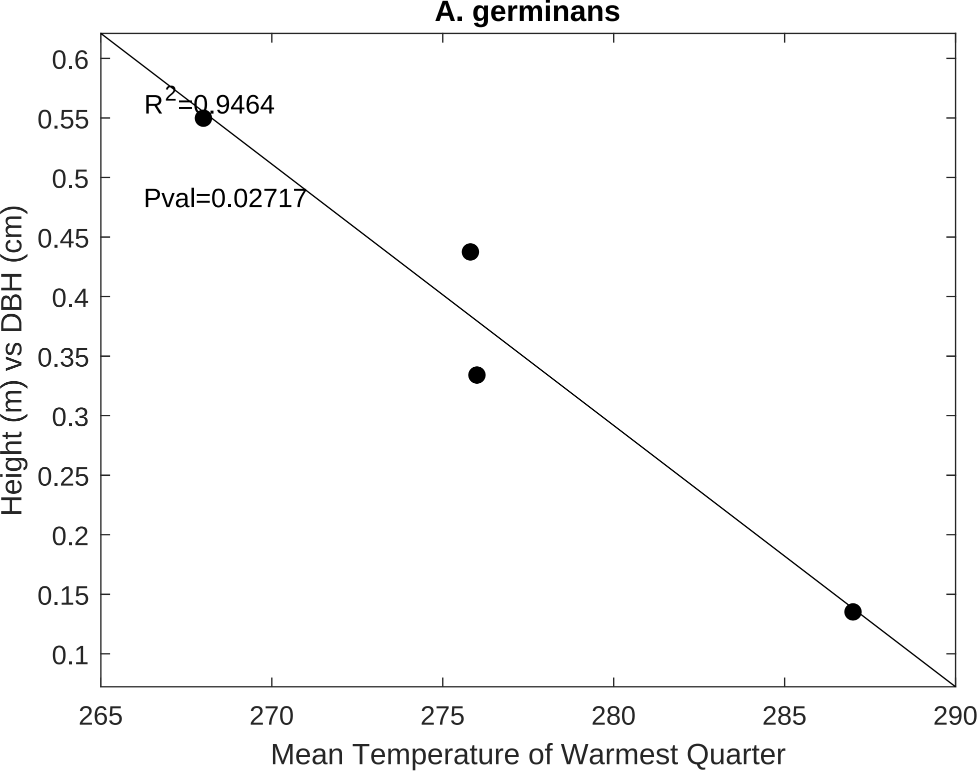
A. germinans
0.6
0.55
0.5
0.45
0.4
0.35
0.3
0.25
0.2
0.15
0.1
Height (m) vs DBH (cm)
R2=0.9464
Pval=0.02717
265
270
275
280
285
290
Mean Temperature of Warmest Quarter

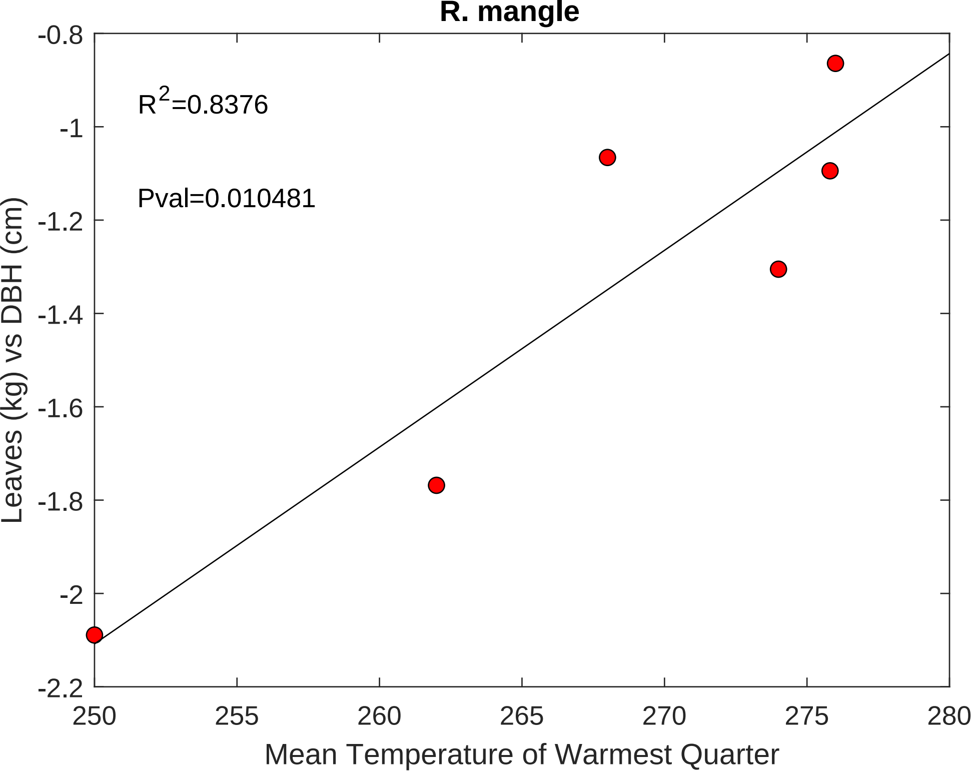
R. mangle
R^2=0.8376
Pval=0.010481
Leaves (kg) vs DBH (cm)
Mean Temperature of Warmest Quarter
-0.8
-1
-1.2
-1.4
-1.6
-1.8
-2
-2.2
250
255
260
265
270
275
280

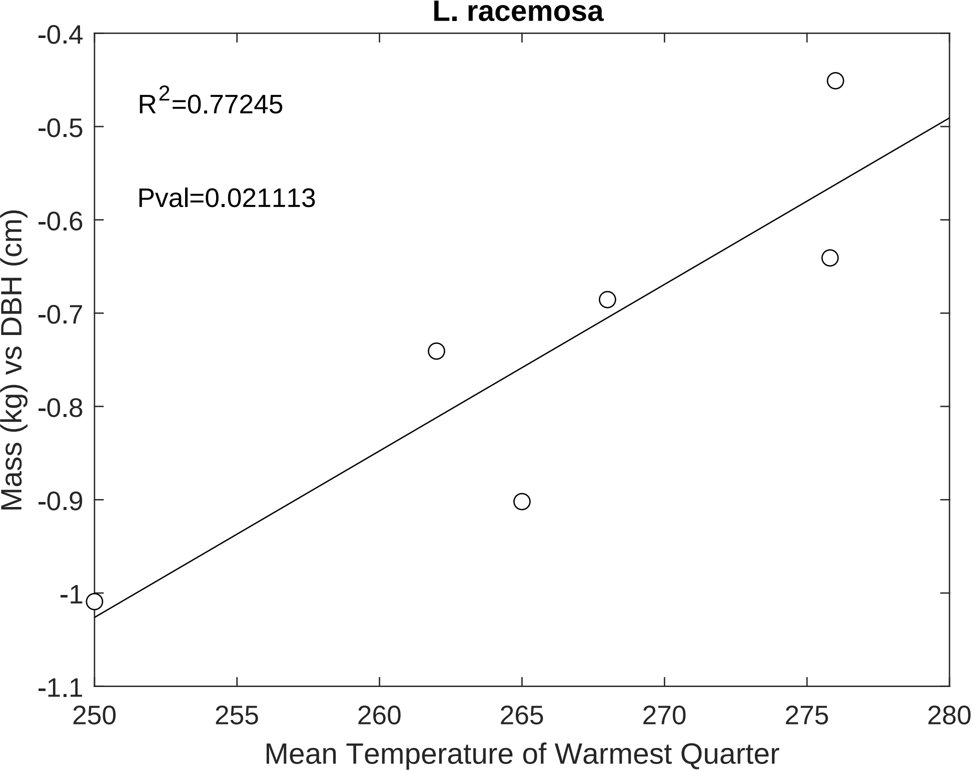
L. racemosa
R2=0.77245
Pval=0.021113
Mass (kg) vs DBH (cm)
Mean Temperature of Warmest Quarter
-0.4
-0.5
-0.6
-0.7
-0.8
-0.9
-1
-1.1
250
255
260
265
270
275
280

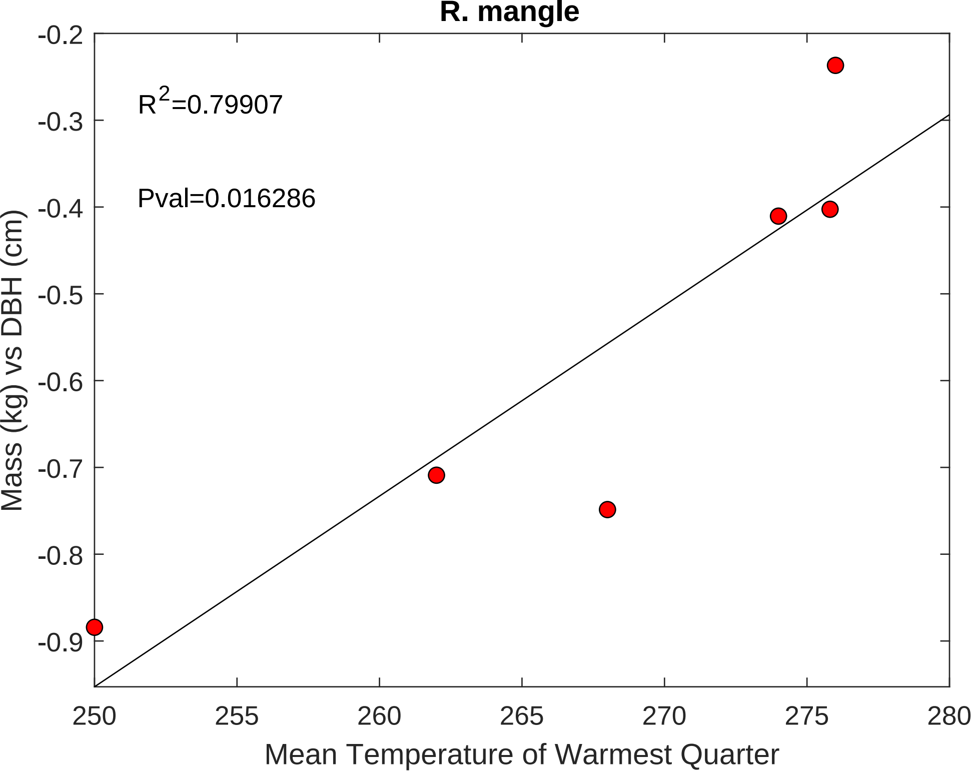
R. mangle
R2=0.79907
Pval=0.016286
Mass (kg) vs DBH (cm)
-0.2
-0.3
-0.4
-0.5
-0.6
-0.7
-0.8
-0.9
250
255
260
265
270
275
280
Mean Temperature of Warmest Quarter

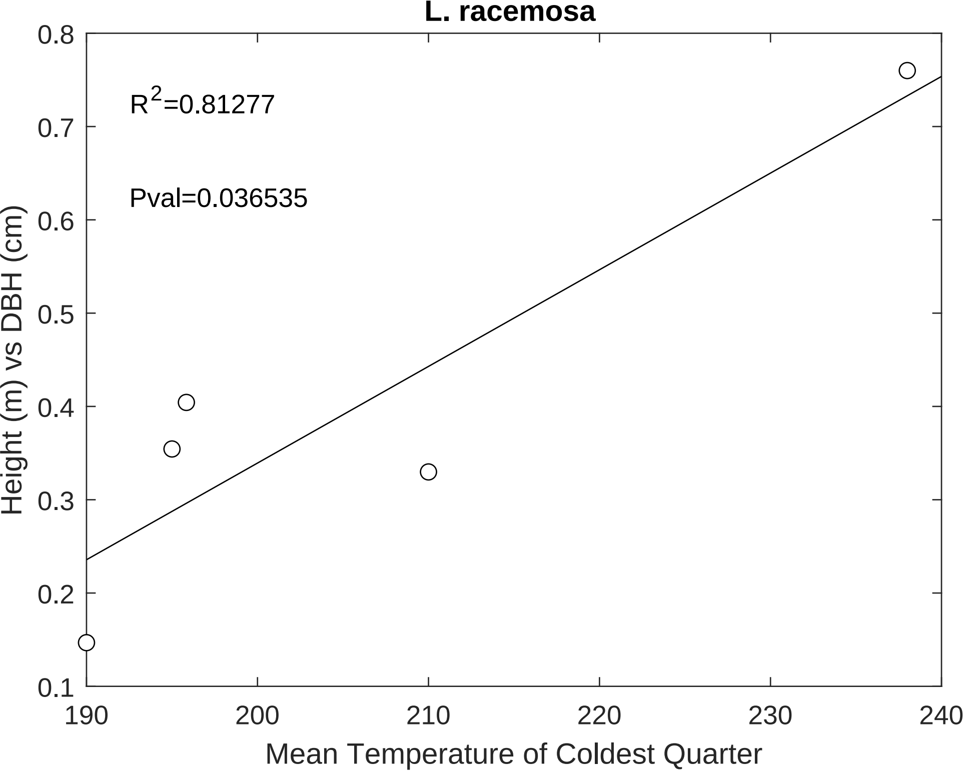

L. racemosa
R^2=0.81277
Pval=0.036535
Height (m) vs DBH (cm)
Mean Temperature of Coldest Quarter
0.8
0.7
0.6
0.5
0.4
0.3
0.2
0.1
190
200
210
220
230
240

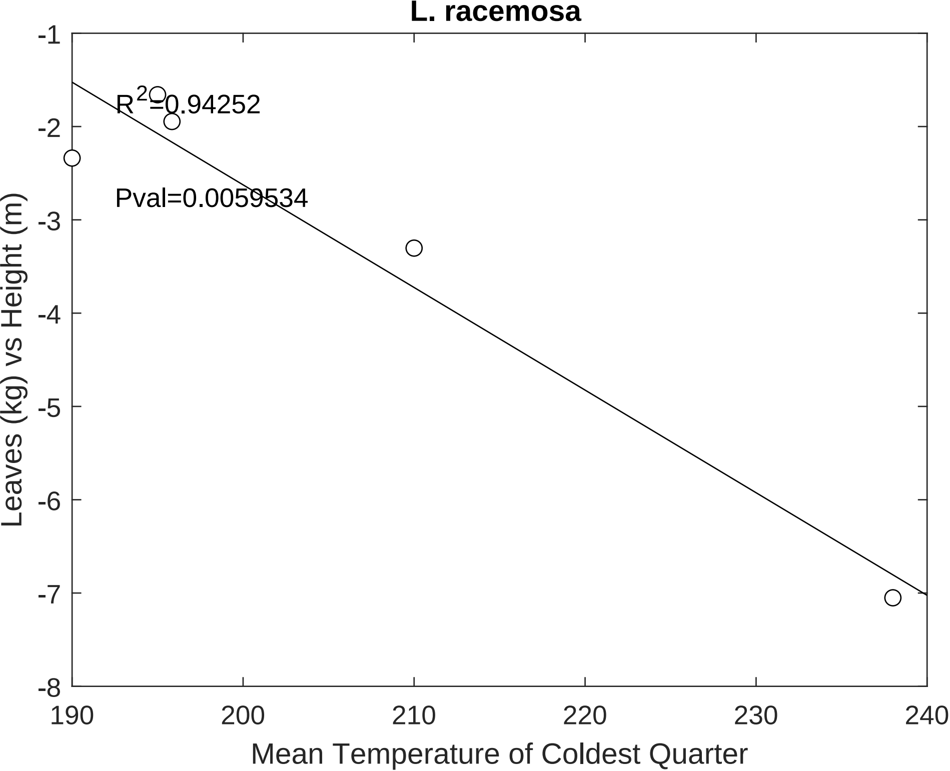
L. racemosa
R^2=0.94252
Pval=0.0059534
Leaves (kg) vs Height (m)
Mean Temperature of Coldest Quarter
-1
-2
-3
-4
-5
-6
-7
-8
190
200
210
220
230
240

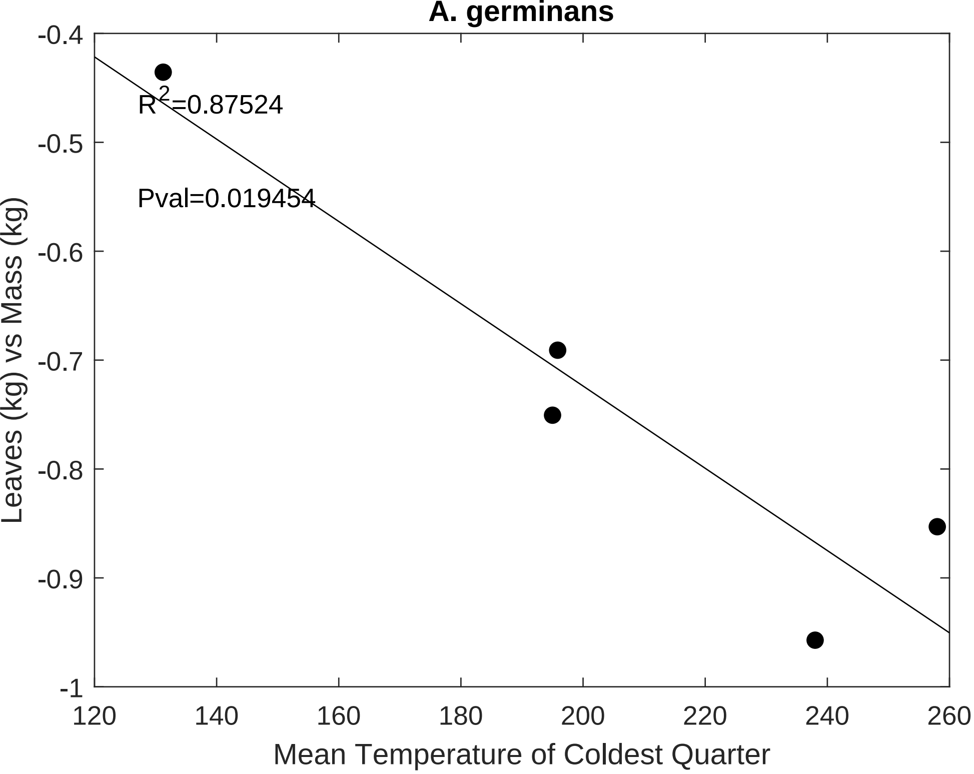
A. germinans
R^2=0.87524
Pval=0.019454
-0.4
-0.5
-0.6
-0.7
-0.8
-0.9
-1
Leaves (kg) vs Mass (kg)
120
140
160
180
200
220
240
260
Mean Temperature of Coldest Quarter

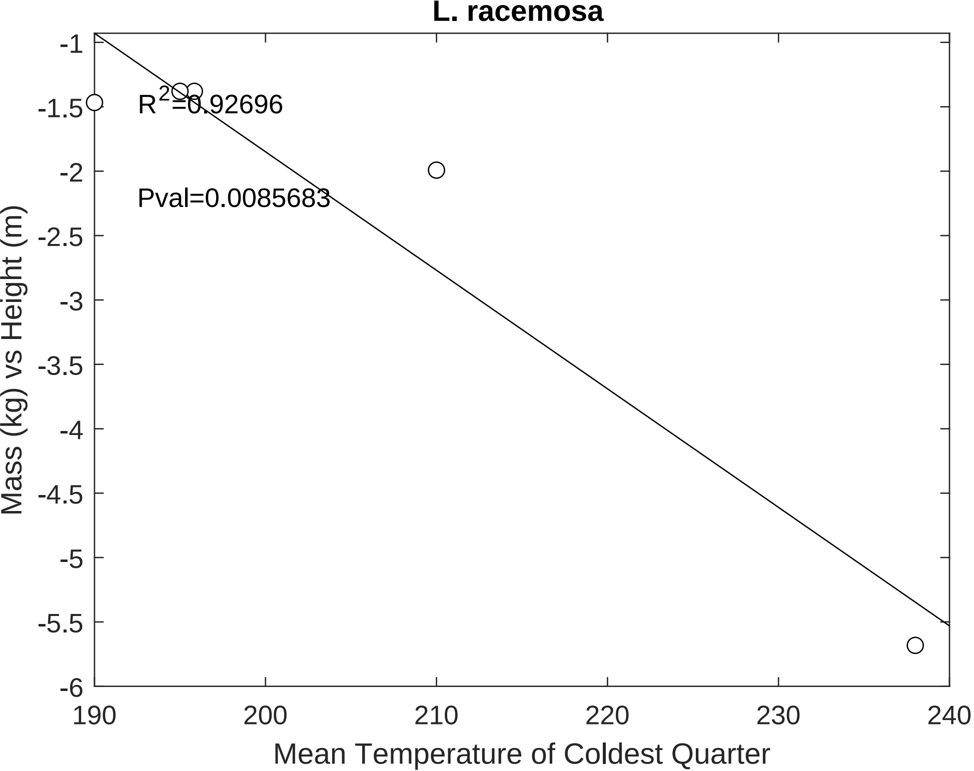
L. racemosa
R^2=0.92696
Pval=0.0085683
Mass (kg) vs Height (m)
Mean Temperature of Coldest Quarter
-1
-1.5
-2
-2.5
-3
-3.5
-4
-4.5
-5
-5.5
-6
190
200
210
220
230
240

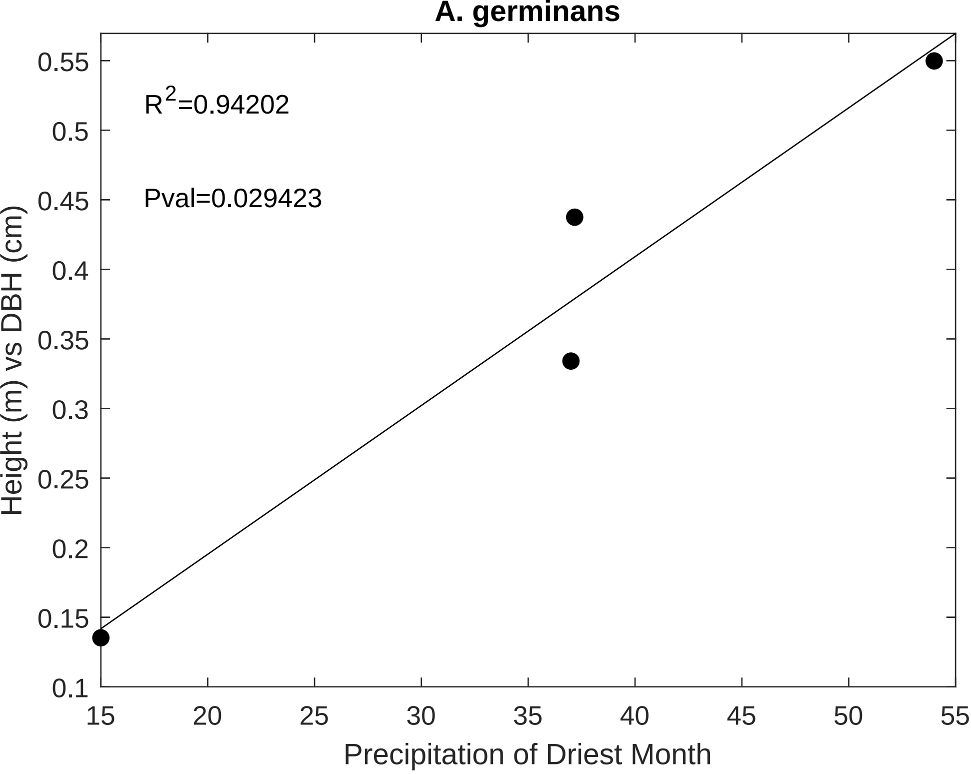

A. germinans
R2=0.94202
Pval=0.029423
Height (m) vs DBH (cm)
Precipitation of Driest Month
0.1
0.15
0.2
0.25
0.3
0.35
0.4
0.45
0.5
0.55
15
20
25
30
35
40
45
50
55

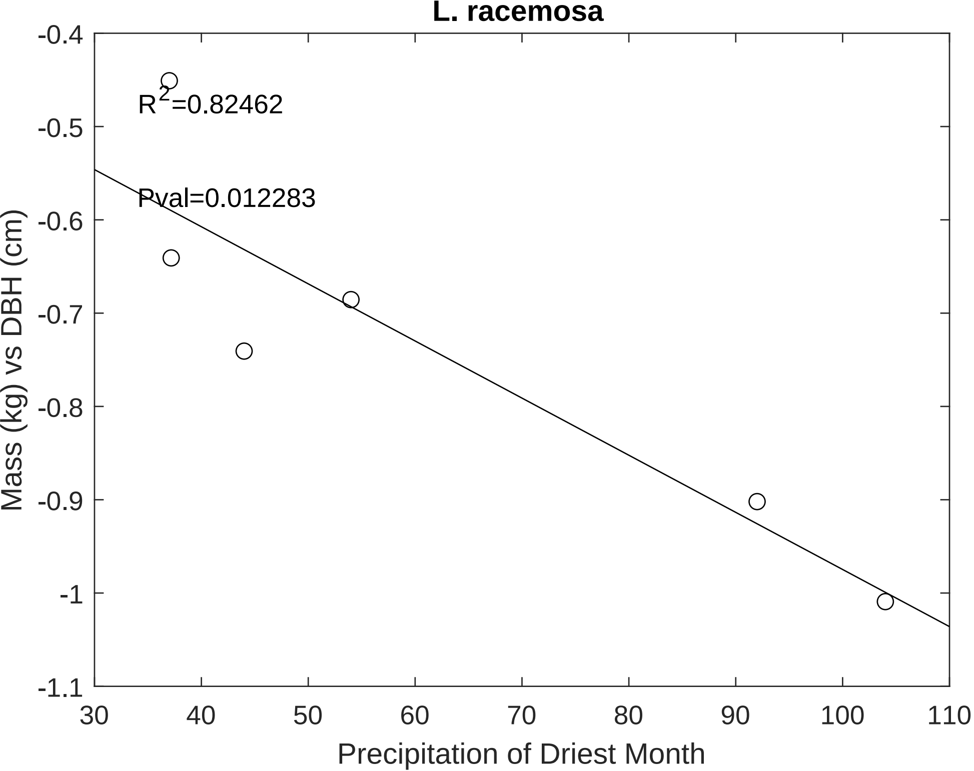
L. racemosa
R^2=0.82462
Pval=0.012283
Mass (kg) vs DBH (cm)
Precipitation of Driest Month
-0.4
-0.5
-0.6
-0.7
-0.8
-0.9
-1
-1.1
30
40
50
60
70
80
90
100
110

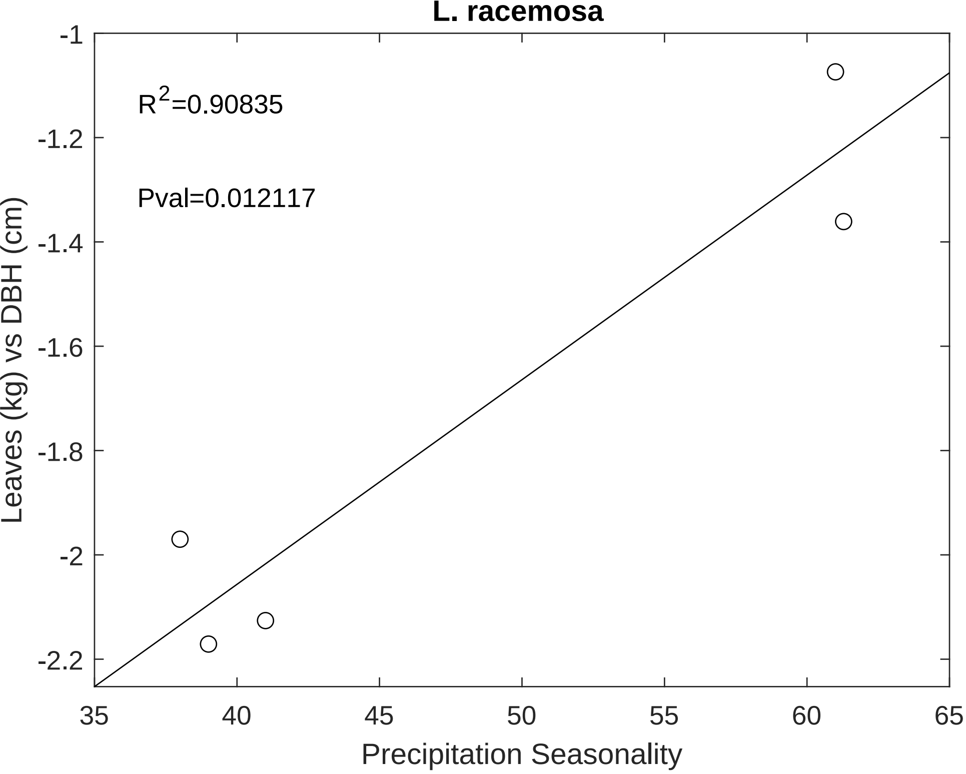
L. racemosa
R2=0.90835
Pval=0.012117
Leaves (kg) vs DBH (cm)
-1
-1.2
-1.4
-1.6
-1.8
-2
-2.2
35
40
45
50
55
60
65
Precipitation Seasonality

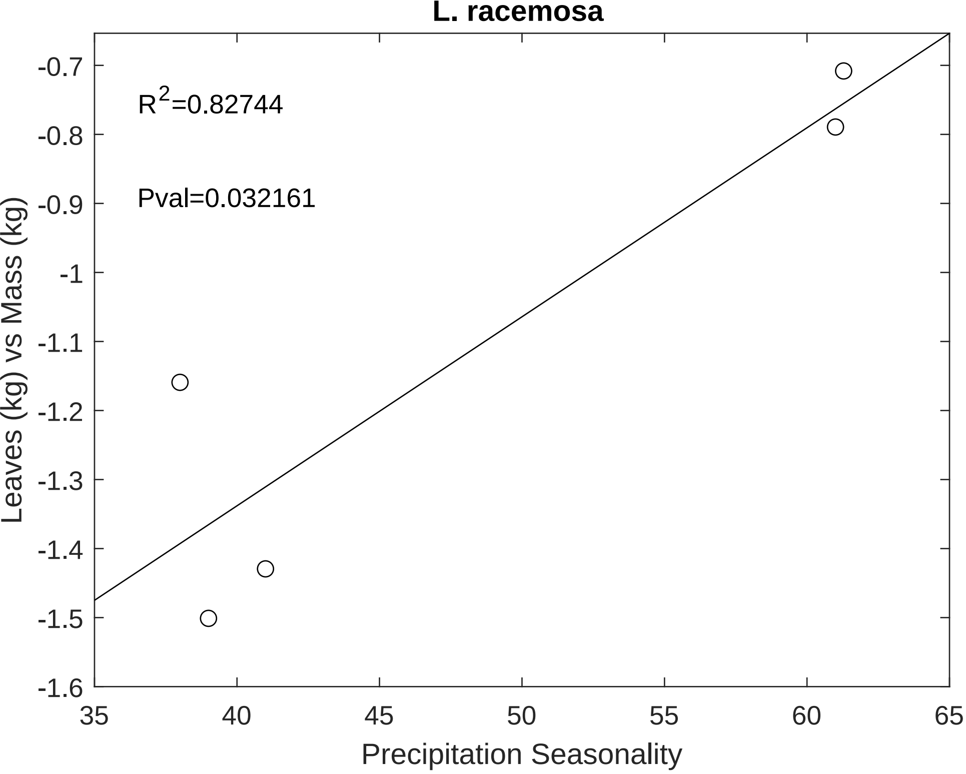
L. racemosa
R2=0.82744
Pval=0.032161
Leaves (kg) vs Mass (kg)
Precipitation Seasonality
-0.7
-0.8
-0.9
-1
-1.1
-1.2
-1.3
-1.4
-1.5
-1.6
35
40
45
50
55
60
65

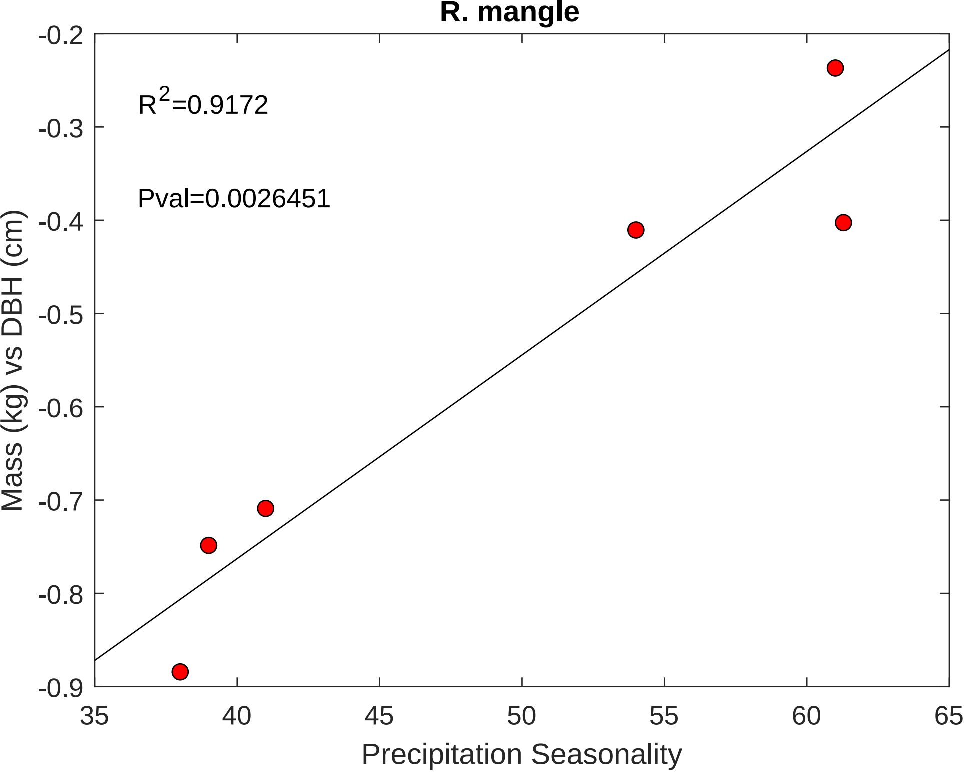
R. mangle
R^2=0.9172
Pval=0.0026451
Mass (kg) vs DBH (cm)
Precipitation Seasonality
-0.2
-0.3
-0.4
-0.5
-0.6
-0.7
-0.8
-0.9
35
40
45
50
55
60
65

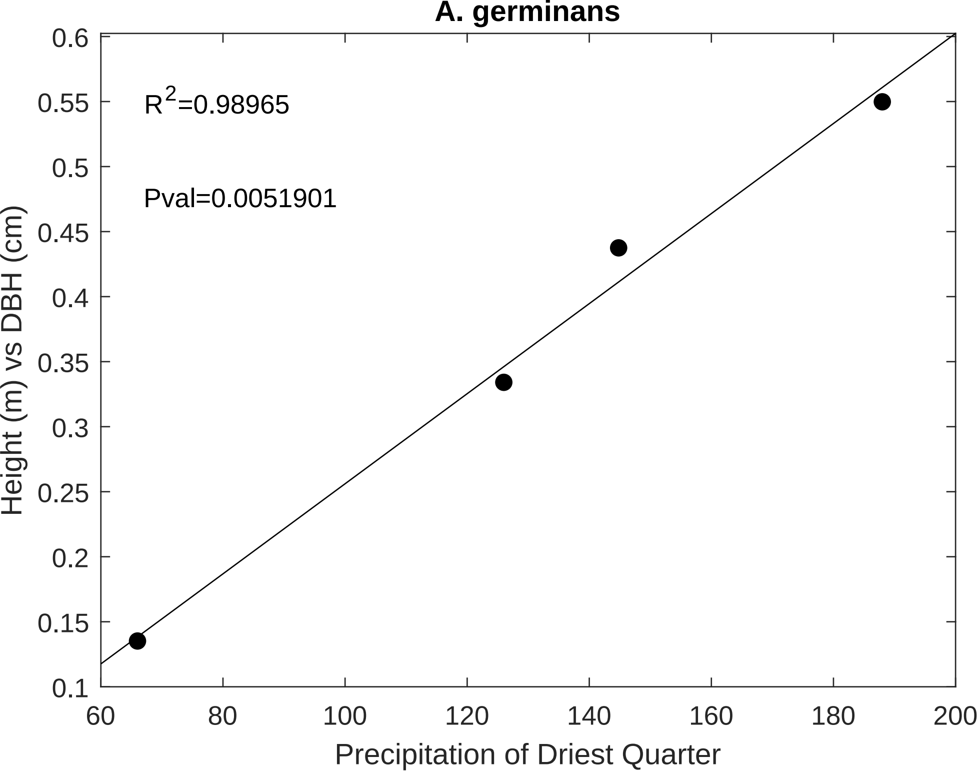

A. germinans
R^2=0.98965
Pval=0.0051901
Height (m) vs DBH (cm)
Precipitation of Driest Quarter
0.1
0.15
0.2
0.25
0.3
0.35
0.4
0.45
0.5
0.55
0.6
60
80
100
120
140
160
180
200

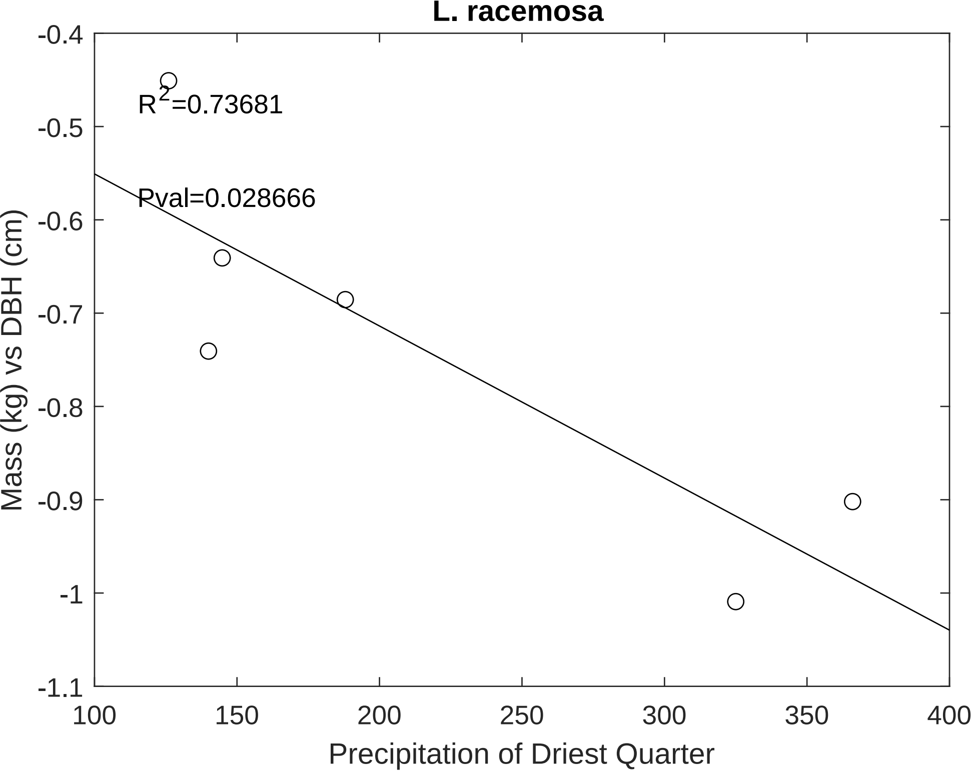

L. racemosa
R^2=0.73681
Pval=0.028666
-0.4
-0.5
-0.6
-0.7
-0.8
-0.9
-1
-1.1
100
150
200
250
300
350
400
Mass (kg) vs DBH (cm)
Precipitation of Driest Quarter

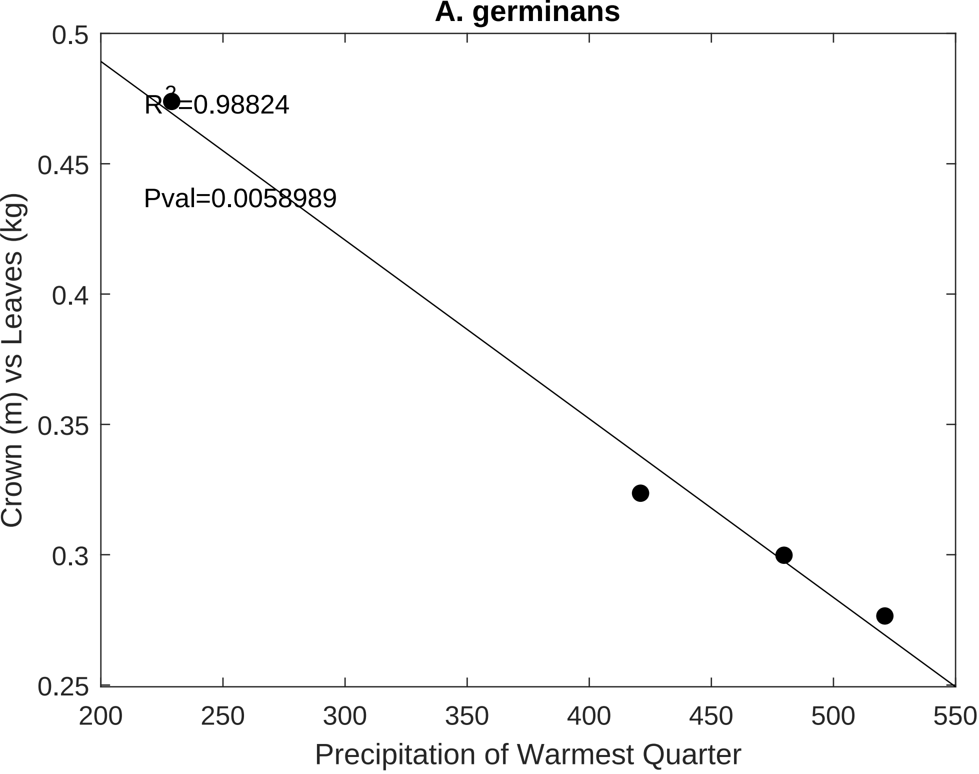
A. germinans
R^2=0.98824
Pval=0.0058989
Crown (m) vs Leaves (kg)
0.5
0.45
0.4
0.35
0.3
0.25
200
250
300
350
400
450
500
550
Precipitation of Warmest Quarter
