# Supplementary figures and images for: Global Data Compilation Across Climate Gradients Supports the Use of Common Allometric Equations for Three Transatlantic Mangrove Species
Source: Ecol Evol. 2024 Nov 20;14(11):e70577. doi: 10.1002/ece3.70577 (PMC11578852; doi:10.1002/ece3.70577)

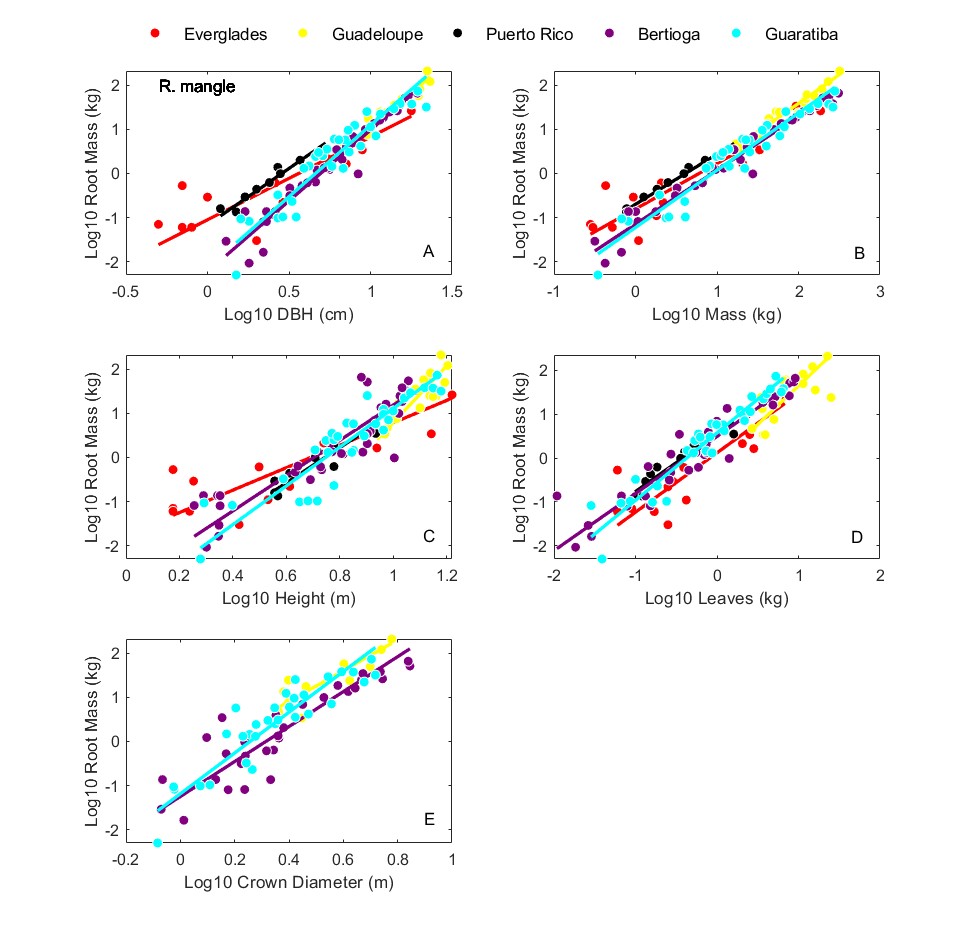

Supplement: Supplementary file 1 — Figures S1–S147. [file ECE3-14-e70577-s002.zip › Fig S1 R mangle Roots.jpg]
